# Pharmaceutical company responses to Canadian opioid advertising restrictions: A framing analysis

**DOI:** 10.1371/journal.pone.0287861

**Published:** 2023-06-29

**Authors:** Daniel Eisenkraft Klein, Joel Lexchin, Abhimanyu Sud, Itai Bavli

**Affiliations:** 1 Dalla Lana School of Public Health, University of Toronto, Toronto, Ontario, Canada; 2 Department of Family and Community Medicine, Faculty of Medicine, University of Toronto, Toronto, Ontario, Canada; 3 Humber River Hospital, Toronto, Ontario, Canada; 4 Department of the History of Science, Faculty of Arts and Sciences, Harvard University, Cambridge, Massachusetts, United States of America; 5 School of Population and Public Health, Faculty of Medicine, The University of British Columbia, Vancouver, British Columbia, Canada; University of the Witwatersrand, SOUTH AFRICA

## Abstract

The pharmaceutical industry’s promotion of opioids in North America has been well-documented. Yet despite the clear consequences of improperly classifying pharmaceutical company messaging and frequently permissive approaches that allow the pharmaceutical industry to self-regulate its own advertising, there has been scarce investigation to date of how pharmaceutical industry stakeholders interpret definitions of “advertising.” This study explores how variations of “marketing” and “advertising” are strategically framed by the different actors involved in the manufacturing and distribution of pharmaceutical opioids. We employed a framing analysis of industry responses to Health Canada’s letter to Canadian manufacturers and distributors of opioids requesting their commitment to voluntarily cease all marketing and advertising of opioids to health care professionals. Our findings highlight companies’ continuing efforts to frame their messaging as “information” and “education” rather than “advertising” in ways that serve their interests. This study also calls attention to the industry’s continual efforts to promote self-regulation and internal codes of conduct within a highly permissive federal regulatory framework with little concern for violations or serious consequences. While this framing often occurring out of public sight, this study highlights the subtle means through which the industry attempts to frame their promotion strategies away from “marketing”. These framing strategies have significant consequences for the pharmaceutical industry’s capacity to influence healthcare professionals, patients, and the general public.

## Introduction

### The prescription opioid crisis and industry culpability

Similar to the United States, opioid-related harms in Canada have continued to escalate over the last half-decade. There were 32,632 apparent opioid toxicity deaths in Canada between January 2016 and June 2022, with the daily death total rising from 8 per day in 2016 to 20 per day in the first half of 2022. Ninety percent of those deaths have occurred in three of the country’s most populous provinces of British Columbia, Alberta, and Ontario. The majority of deaths were among individuals aged 20 to 59, with males accounting for over three quarters. In addition to deaths, there were 2,524 opioid-related hospitalizations in the first half of 2022 [1].

While most current opioid toxicity deaths are related to high potency illicit opioids such as fentanyl and its analogues [2], a combination of misleading marketing campaigns by opioid manufacturers and overall lax federal regulation are recognized as key originating factors of the crisis [3, 4]. This interpretation is highlighted by the extensive research that has documented Purdue Pharma’s [5] and other companies’ efforts to disguise their promotion as information provision and education [2, 6–8].

Purdue Pharma applied various strategies to mislead the medical community about the safety and effectiveness of OxyContin (controlled-release oxycodone)—overstating, for example, its effectiveness for treating chronic pain and downplaying the risk of addiction and extramedical use in its promotion to healthcare stakeholders, including physicians and patient groups [6, 9, 10]. While significant attention has been paid to the industry’s role in the United States (US), many of these same tactics occurred in Canada. For instance, Purdue paid 100 doctors more than $2000 per talk in the early 2000s to speak to Canadian physicians about pain management [11]. Purdue also sponsored and distributed educational materials for Canadian doctors and medical students that downplayed the dangers of OxyContin [12]. The company has recently settled a national class action lawsuit alleging “that opioid manufacturers, distributors and their consultants engaged in deceptive marketing practices with a view to increase sales, resulting in increased rates of addiction and overdose” [13].

### Health Canada’s regulation of advertising and promotion

Health Canada is the national medicines regulator in Canada, responsible for approving new medicines and monitoring the safety and effectiveness of products on the market [14]. Although the Food and Drugs Act and Regulations theoretically give Health Canada control over promotion [15], it has largely taken a hands-off approach. There is no record of Health Canada ever having imposed any penalties for illegal promotion of prescription medications [16]. A Health Canada spokesperson was asked by a reporter from the Toronto Star why several companies prosecuted for illegally marketing medicines for unapproved uses in the US had not been prosecuted in Canada for selling the same products. The response to the reporter was that Health Canada “has not been made aware of any specific similar issue in Canada and has not received complaints concerning these companies promoting off-label uses of their products in Canada” [17], despite significant evidence to the contrary [18, 19].

A number of studies have pointed out Health Canada’s failures to control the promotion of opioids in Canada, allowing Purdue to spread misinformation about OxyContin in particular and opioids in general [20]. Based on little evidence, in 1996 Health Canada approved the drug for the management of moderate pain, ignored the risk of addiction, and allowed the statement in the product monograph that the risk of misuse is low [21, 22]. In addition, the product monograph provided no recommended maximum dose, allowing the drug to be marketed and prescribed with no upper dose limit [23]. It also took Health Canada more than ten years to revise misleading claims that appeared in the OxyContin product monograph [21]. In such cases, companies may continue to promote misleading claims until the product monographs are modified, giving companies such as Purdue a decade of opportunity to misleadingly influence physician prescribing practices [24].

### Health Canada’s distinction between advertising and education

In the policy document, “The Distinction Between Advertising and Other Activities,” Health Canada notes the importance to both the pharmaceutical industry and to the general public of being able to disseminate and access non-promotional information regarding medicines for human use. The document defines “advertising” as “any representation by any means whatsoever for the purpose of promoting directly or indirectly the sale or disposal of any food, drug, cosmetic or device" [25]. Health Canada determines whether a message should be classified as promotional via a number of factors, including the content, its dissemination context, the audience, the provider of the message, the sponsor, the overall influence of the drug manufacturer on the message, and the frequency of the message. Health Canada explicitly notes that, “No one factor in itself will determine whether or not a particular message is advertising. Each message must be evaluated on its own merit and other factors may apply” [25]. This leeway leaves Health Canada with significant discretionary power to determine a message’s classification.

Though Health Canada makes a distinction between advertising and other activities, the extent to which it enforces these distinctions and to which industry follows them is unclear–adherence to restrictions remains largely self-regulated and voluntary [26]. Canadian pharmaceutical companies have a strong incentive to avoid classifying materials as advertising or promotion. When a health product’s messaging is considered promotion, it is subject to advertising provisions within the *Food and Drugs Act* and *Regulations* (FDAR) [15]. The FDAR set out numerous restrictions on advertising, while information provision and educational materials face few federal restrictions. This gap between information and advertising typically allows pharmaceutical companies substantial room to promote their products.

Taken together, opioid companies’ promotional tactics and insufficient action taken by Health Canada to regulate industry activities have contributed to a large increase in opioid prescribing associated with high rates of addiction, extramedical use, and overdose deaths in both Canada.

### Health Canada’s most recent action around opioid promotion

In June 2018, in response to escalating opioid-related harms, Dr. Ginette Petitpas Taylor, the then federal Health Minister with direct responsibility for the activities of Health Canada, issued a letter to Canadian manufacturers and distributors of opioids requesting their commitment to voluntarily cease all marketing and advertising of opioids to health care professionals [27]. Direct-to-consumer pharmaceutical advertising is highly restricted in Canada. Responses by the companies to the letter were voluntary with no sanctions for failing to reply. Despite, in Health Canada’s own words, “recognizing the urgency of the opioid crisis and the role that the marketing and advertising of opioids may be playing” [28], communications between Dr. Petitpas Taylor and pharmaceutical manufacturers provided no rationale for favoring voluntary action by industry over government regulation. In March 2019, Health Canada signaled its intention to further restrict all advertising materials provided to healthcare professionals regarding Class B opioids (opioids that are subject to marketing authorization terms and conditions). Starting in June 2019 only messaging with verbatim statements authorized by Health Canada in the product monograph were allowed [29].

Despite the clear consequences of improperly classifying pharmaceutical company messaging and Health Canada’s permissive approach to allow the pharmaceutical industry to self-regulate its advertising, there has been scarce investigation to date of how pharmaceutical industry stakeholders follow or interpret definitions of “promotion”, “advertising” and “marketing”. This study was undertaken to better understand how pharmaceutical manufacturers and industry associations agreed with, interpreted, or resisted Health Canada’s request to cease all marketing and promotion of opioids. The study aims to explore how variations of the three terms are variously and strategically framed in the context of pharmaceutical opioids by the different actors involved in the manufacturing and distribution of these products.

## Methods

One of the authors (DEK) filed a Freedom of Information Act request requesting all the “final, public facing documents of stakeholder communications in regards to the Marketing and Promotion of Pharmaceutical Opioids initiatives” received by Health Canada from June 1, 2018 to December 31, 2020 in response to the letter that it sent in June 2018 to manufacturers and distributors of opioids.

We conducted a framing analysis of all the material received in response to this request (S1 File), in order to explore the question of “how did industry actors frame forms of promotion, marketing, and advertising?” Framing is premised on the idea that an issue can be viewed from a variety of perspectives, and presented as having implications for various values and considerations [30]. The framing of an issue thus opens particular policy approaches so that the policy process becomes a struggle over ideas, meanings, and competing interpretations [31]. Frames highlight particular aspects of a contested situation while concealing others, so as to define problems, identify causes, make moral judgments, and propose solutions [32]. Framing analysis attempts to analyze precisely which frames are being used, and policy framing analysis seeks to understand the ways that policies are presented and assigned meaning. It is principally concerned with the social construction of problems and their related policy solutions. This form of analysis pays particular attention to the policy positions taken, the terms and “frames” that these positions are couched in, and the evidence used to support them [30].

Our analysis specifically drew on the increasing volume of research that highlights how corporations use framing tactics to further their commercial objectives as part of broader political strategies [33–37]. This analysis consequently aligns with wider research frameworks, including the commercial determinants of health field, where framing analysis is frequently employed as a way to critically examine industry political practices [34]. Despite the prominence of framing analyses in broader policy literature, to the authors’ knowledge this is one of the first framing analyses of the pharmaceutical industry.

The full corpus of documents was first read by each author. An initial set of marketing, promotion, and advertising frames was selected by discussion amongst the members of the group. At this point, the corpus was split in two, with each half reviewed and coded independently in duplicate. Each group of two authors then reviewed and resolved coding amongst themselves. Both groups then discussed their coding together and resolved differences. Minor adjustments in coding were then made by each of the two groups based on this full group discussion. We report only descriptive data and use quotes from responses to illustrate how we constructed the frames.

Companies were defined as brand-name or generic primarily by their membership in either Innovative Medicines Canada (IMC, brand-name) or Canadian Generic Manufacturers Association (generic). When a company was not a member of either organization then its website was consulted and its description of its activities was used to make a classification decision.

All data were publicly available and ethics consent was not required.

## Results

Overall, 103 companies and organizations were contacted by Health Canada. We analyzed 102 pages of response documents from 41 (39.8%) unique responding companies and organizations, with some providing multiple responses, leading to a total of 50 responses. Responses came from 14 brand-name companies, 20 generic companies, 2 company associations, and 5 other organizations. One organization (the Ontario Pain Foundation) provided a response despite there being no record that this organization was a recipient of Health Canada’s letter.

We identified a spectrum of four notable and intersecting frames ranging from a denial that the company engaged in marketing and therefore has nothing to answer to, to a defense that activities did not constitute marketing or were designed to protect the public: 1) *Denial of marketing/promoting/advertising/selling*; 2) *Rules-based approach*; 3) *Information Provision*; and 4) *Redefining marketing* (Table 1).

**Table 1:** Overall results.

Frame	Number of Companies and Organizations using Frame*			Illustrative Example
	Brand-name companies (n = 14)	Generic companies (n = 20)	Others (n = 7)†	
**Denial of Marketing/Promoting/Selling**	12	17	5	*Vita Health Products Inc*. *does not engage in the marketing or advertising of opioid products (either directly or indirectly) to health professionals*–Vita
**Rules-Based Approach**	4	5	4	*Any activities that may be undertaken in the future*, *will fully comply with the terms and conditions on specific opioid products under authority of section C*.*01*.*014*.*21 of the Food and Drug Regulations*.–Sanis Health
**Information Provision**	8	8	4	*We will continue to share helpful educational and scientific information on these products in compliance with the marketing authorizations received from Health Canada*–Pfizer *Member companies of CGPA*, *therefore*, *support efforts to educate physicians on appropriate prescribing and patient education on appropriate use and safe storage to prevent diversion–*Canadian Generic Pharmaceutical Association *We support evidence-based*, *non-branded*, *and independently accredited Continuing Health Education [CHE] programs to improve clinical care and patient outcomes*–Purdue *We remain steadfast in our belief that Canadian prescribers require the most recent information*, *including on the most current guidelines*, *to ensure their patients are treated appropriately*. *Going forward*, *requests for information from our opioid products from healthcare professionals will be addressed reactively through direct communication with the experienced healthcare professionals in our Medical Affairs Department*–Purdue
**Redefining marketing**	2‡	4	1	*Understanding the unique characteristics of evidence-based OUD therapies and their appropriate use becomes critically important as we work to address our national public health crisis*. *In fact*, *when there is a clear public health benefit associated with the adoption and integration of medicines that address important public health concerns*, *such as vaccines*, *industry has been permitted to advertise and promote these medicines even beyond prescribing healthcare professionals*—Sterimax

*Some companies and organizations used more than one frame.

^†^Brand-name and generic associations, patients’ groups, and other types of companies.

^‡^Stated by Innovative Medicines Canada, representing 48 Canadian pharmaceutical companies.

The outlier in the responses that Health Canada received came from the Ontario Pain Foundation, the only respondent that was not part of the pharmaceutical industry. The Foundation asserted that reliable research evidence demonstrates that the causes underlying the current opioid crisis do not lie with legitimate prescriptions for medical uses.

### Denial of marketing/Promoting/Advertising/Selling

Nineteen companies denied promoting or marketing opioids and one company said it would stop marketing in the future, but many of their statements used terms without completely defining them and companies were unclear about whether they actually sold them and what they meant by the term “marketing”. For instance, while Bristol-Myers Squibb specifically clarified that it “does not currently manufacture, distribute, or sell opioids as part of its product portfolio,” Vita Health Products simply responded it “does not engage in the marketing or advertising of opioid products (either directly or indirectly) to health professionals.” Some companies seemed to use the term marketing interchangeably with promotion or advertising, whereas other companies seemed to mean selling. The same lack of precision applied to an additional 5 companies that declared that they had already stopped or would restrict promoting or marketing opioids and one company that said it did not distribute opioids. Six companies said they did not sell opioids.

The Canadian Generic Pharmaceutical Association clarified that they did not market opioids, as did four companies selling over-the-counter products, preparing drugs for intravenous injection, or selling medical equipment—Church & Dwight, Laboratoire Confab, Vita, and ICU Medical.

### Rules-based approaches

“Rules-based approaches” included responses that explicitly stated that respondents were following and/or would continue to follow all marketing rules but did not deny past or future marketing of opioid products. Thirteen companies stated that they had and/or would be careful to continue to market according to the present and future guidelines laid out by Health Canada. Ethylpharm, for instance, noted its commitment to working with Health Canada and stated that it had as such “suspended all its promotional activities pending the result of the Public Consultation.” It did not provide any further information on these activities or which actions it would take after completion of the consultation. These responses also did not necessarily explicitly state that companies would change their marketing practices.

### Information provision

Information provision took a number of different forms with some companies and organizations distinguishing marketing from education, including continuing health education programs, educating physicians on prescribing opioids, and patient education about opioid use and safe storage. Other companies focused more on their responsibility to distribute scientific information to physicians such as the 2017 Canadian Guideline for Opioids for Chronic Non-Cancer Pain, as opposed to organizing events for them.

Continuing Health Professions Education and its equivalent was more likely to be mentioned by brand-name companies–four out of 14 (29%)–as compared to generic companies–three out of 20 (15%). IMC, the primary representative organization of the Canadian brand-name pharmaceutical industry, cited “the value of the biopharmaceutical industry conveying education and scientific information about health products, which has been acknowledged by Health Canada and should not be undermined.” Others noted their “support” for education, while not specifying their own approach to education. Teva, for instance, noted “[w]e support efforts to educate physicians on appropriate prescribing and patient education about opioid use and the safe storage of all medicines.” Indivior positioned itself as placing “a strong emphasis on offering education to reduce the stigma associated with [opioid use disorder] which prevent patients from seeking treatment” and Purdue emphasized its commitment to following the 2017 Canadian Guideline for Opioids for Chronic Non-Cancer Pain [31] and to evidence-based, non-branded, and independently accredited Continuing Health Education (CHE) programs” as being “essential to drive medical progress and improve patient outcomes.”

Generic companies concentrated more narrowly on distributing scientific information. Five out of 20 generic companies emphasized the need to engage in “information provision,” with a particular focus on scientific information as opposed to 2 out of 14 brand-name companies.

Finally, two companies–Pfizer and Purdue Pharma–specifically noted that they would continue to respond to direct requests for information on their products from individual healthcare professionals, in Purdue’s case “through direct communication with the experienced healthcare professionals in [its] Medical Affairs Department.” This engagement is in distinction to pro-active and general marketing activities conducted by these companies.

### Redefining marketing

This frame also took a number of forms. Five companies specifically distinguished marketing from the importance of the industry’s role in reducing the risks of the products and opioids in general, often distinguishing opioids from opioid use disorder therapies such as sublingual buprenorphine. Companies’ responses regarding risk reduction included arguing for the need to advertise opioid use disorder therapies to healthcare professionals and the public, working with Health Canada towards solutions to decrease opioid addiction and misuse, and submitting risk management plans. Indivior’s response, for instance, was *predominantly* focused on opioid use disorder therapies, with little material on opioid analgesics themselves. This focus on risk reduction was reiterated by IMC in its response.

A second way of redefining marketing was to claim that certain activities did not constitute marketing or they were already regulated and therefore no further action was necessary. IMC specifically emphasized that marketing practices did not include "…reimbursement for travel and hospitality expenses to attend-industry sponsored events, and gifts of meals, equipment…" and that IMC’s *internal* Code of Ethical Practices already placed restrictions on them. While the IMC response was coded as a single entry, we believed that it was reasonable to assume that this perspective represented the views of the organization’s 48 member companies.

## Discussion

Overall, there were a multitude of frames in the responses that were largely determined by the respondent’s positionality and stake in opioid manufacturing, distribution, and utilization; some companies manufactured, sold and promoted brand-name opioids, other companies engaged in the same activities for generic opioids and some companies were distributors.

### Avoidance of labeling activities as “promotion” or “advertising”

Many generic companies, and their association, the Canadian Generic Pharmaceutical Association, maintained that while they distributed opioids, they did not market or advertise them, thereby absolving themselves from opioid-related harms. This type of response ignores the fact that these companies had been profiting from opioid promotion by others and have been implicated in class-action lawsuits in Canada and the US. Some generic companies such as AA Pharmaceuticals provided an “information provision” response, stating that it “will provide product monographs upon request” although product monographs had contained misleading statements such as the one for OxyContin (oxycodone) [21].

The frame of information provision was used not just to absolve responsibility with respect to the particular issues of marketing and promotion, but pushed even further to position the importance, to the public good, of industry involvement in the discourse with health professionals about the safety, efficacy, and appropriateness of opioid analgesics. The frame was used by both generic and brand-name companies, although more frequently defined as “education” by brand-name companies, while “information provision” was more common amongst generic companies. By drawing on, for example, the distribution of national clinical practice guidelines for opioid prescribing as part of their educational and information provision activities, the companies implied that these activities were based on scientific evidence and free of commercial influence. However, these responses obscure findings that drug company education of physicians frequently leads to inappropriate prescribing [38]. Several studies have shown that both promotional and educational practices by pharmaceutical companies are associated with lower prescribing quality, higher prescribing frequency of the branded drug, and higher drug costs [39–42].

A smaller subset of responses, focused specifically on opioid use disorder, pushed even further to identify that their activities, whether promotion, marketing, education, information provision or otherwise, were essential to reducing opioid-related harms. The implication, then, is that restrictions on such activities would be opposed to the intended impact of Health Canada’s measures aiming to reduce risks related to opioids. This messaging is consistent with other analyses that have examined specifically how the concept of risks within health crises can legitimize industry involvement in crisis interventions and over-ride other moral imperatives of avoiding industry conflicts of interest [43].

In addition to attempting to reframe their activities as being non-promotional, industry responses also repeatedly attempted to emphasize that their promotional activities were sufficiently regulated by their own internal codes of conduct [44]. The Code is entirely developed and administered by IMC, and the maximum sanction for a fourth violation of the Code in a 12-month calendar year is $100,000. Evaluations of similar codes in other countries have concluded that there is “a discrepancy between the ethical standard codified in industry Codes of Conduct and the actual conduct of the industry” [45].

### Framing in context

These findings highlight the underlying foundation of interpretivist research that emphasizes policy as a social construct that can be communicated in multiple ways to imply different problems and, by extension, different solutions. Framing offers important insights into the *nature* of these debates, which is particularly applicable to research on corporate strategies that come at the expense of public health. Particular industries commonly frame policy issues in ways that protect their profit-seeking strategies and divert attention away from public health harms [46]. These are often done in subtle ways, as made clear in this study. Companies’ framing strategies generally aimed to either absolve themselves of responsibility (primarily generics) or narrow the definition of marketing to allow them to continue their current practices that support their commercial interests (primarily brands). The difference between the two groups of companies likely reflects the fact that brand-name companies deal directly with physicians whereas generic companies typically do not. The brands identified a number of actions which they hoped to distinguish from marketing, which determined their "frames," including information provision and education. This change in framing around the Canadian opioid crisis follows changes in the trajectory in the larger public discourse. For example, a recent critical content analysis of media reporting on opioids in Canada between 2000–2017 identified a progressive move away in the later years of the crisis from any attention on responsibility of the pharmaceutical industry [47].

This study also provides an example of the *discursive* power–i.e., the power to influence via communication, rather than action–of the Canadian pharmaceutical industry. At the same time that the pharmaceutical industry was attempting to influence policymakers via lobbying [48], companies responded to a call to voluntarily cease marketing of opioids by instead attempting to define and re-frame pharmaceutical marketing itself. These extensive framing strategies highlight the room that Health Canada’s definition of “advertising” leaves for pharmaceutical companies and their representative organizations to bend these definitions towards their interests. The vast difference in regulatory oversight between advertising and information provision appears to have led to the industry searching for framings of messaging that avoid "promotion" or “advertising” labeling. Brand-name companies and their representative organization, IMC, also used their letters as opportunities to reiterate their preference for regulatory measures to remain voluntary and self-regulated.

In addition to analyzing the employed frames, an essential component of framing analysis is examining which frames are missing [49]. These findings suggest that by Health Canada narrowly framing industry’s responsibility for the crisis solely around marketing or advertising rather than the broader industry activities that accelerated the crisis, it permitted industry to specifically frame their responses in ways that avoided larger culpability [46]. Moreover, by specifically framing their activities around the *definition* of advertising, industry was able to frame the issues away from the *content* of the advertising. This insight is noteworthy, given the clear evidence of inaccuracy in many opioid advertisements. For instance, a recent analysis of opioid advertisements in general Canadian and American medical journals found that nearly half failed to mention the addictive potential of opioids, and 74% did not disclose the possibility of death from opioid use [50]. In addition, opioid advertisements generally failed to cite high-quality evidence, instead preferring industry-funded studies.

Separately, while not a focus of this study, these responses also provide an opportunity for further study of the ways in which pharmaceutical companies frame the opioid crisis itself. Companies made numerous efforts throughout their letters to shift the blame for the opioid crisis away from the pharmaceutical industry, and towards misuse, diversion, and illicit distribution. For example, IMC’s use of the phrase “drug overdoses and death involving the misuse” and Teva’s reference to “programs aimed at tackling diversion of opioids”. Analysis of these problem attributions would likely provide important insights into pharmaceutical industry strategies to distract from companies’ roles in the crisis by creating a false narrative that they are not responsible for the harm caused to communities throughout Canada.

Our findings are consistent with other studies that show how opioid manufacturers use various tactics to mislead the medical community about their role in the opioid epidemic [6, 9, 12, 16]. This study extends these prior works by showing that framing strategies may have significant consequences for the pharmaceutical industry’s capacity to influence healthcare professionals, patients, and the public, including being able to successfully disguise from the policy and public discourse its significant responsibility in opioid-related harms [47].

#### Policy implications: Moving forward

It appears that the industry’s framing was largely successful in avoiding any further regulatory action. Health Canada continues to rely on industry compliance with IMC’s code for many aspects of the promotion of all health products. While opioid promotion was eventually required to be pre-cleared by Health Canada, this did not occur until 2020, well after the spike in pharmaceutical opioid use [51].

On one hand, these findings highlight the need for Health Canada and policymakers to shift the Canadian pharmaceutical advertising policy model away from a permissive model consisting of voluntary measures and self-regulation, and towards a more proactive model of regulation. Yet Health Canada’s ability to take on this regulatory role remains in question, given their historical lack of willingness to proactively regulate drug promotion and their simultaneous interest in “promoting pharmaceutical innovation” [52]. This emphasis on innovation has previously been highlighted as a key barrier to health protection and promotion [52], and is further emphasized in our findings.

Regulatory efforts involving promotion should ideally be performed by an agency that is independent of government and industry and set up through legislation. Membership in this agency would be defined in the legislation (e.g., consumers, representatives of medical associations, nursing associations, etc.), and people representing the various organizations would need to be free of any conflicts-of-interest as would the organizations themselves. Funding should be stable and independent of direct government control. Rather than awaiting complaints, an independent body’s proactive regulatory approach would mean, at a minimum, monitoring activities of sales representatives and pre-clearance screening of all print and electronic forms of communication with doctors. Pre-clearance is currently performed by the Pharmaceutical Advertising Advisory Board—funded by fees from pharmaceutical companies—and is regulated under a very weak code [53]. There would need to be significant consequences for promotion violations and sufficiently strong disincentives to seek loopholes. There has been extensive writing on how this third-party model could operate, including in its funding [54]. This change in how promotion is regulated could occur in tandem with more significant oversight and regulation from Canadian medical education bodies and professional societies, such as the College of Family Physicians of Canada and the Royal College of Physicians and Surgeons of Canada.

Regardless of whether Health Canada or a third-party body regulates pharmaceutical promotion, this study highlights how industry is likely to gravitate to “education” and “information provision” specifically *because* these are soft spots within the healthcare regulatory landscape. Increased regulation around these frames is thus necessary regardless of the regulatory body involved. These results re-emphasize the need to institute the proper checks and balances to ensure that pharmaceutical promotion is ultimately serving the public interest.

### Limitations

The frames that we identified may have been limited by the relatively low response rate. Only 41 companies and organizations responded to the letter, out of 103 contacted. It is unclear how many of these non-responses were because they did not actually make the product, as opposed to a desire to ignore Health Canada’s request. Although it may be reasonably inferred that the request did not apply to many of the companies, others that were impacted may not have felt compelled to respond given the voluntary nature of the request, further emphasizing the permissive nature of Health Canada’s actions in this area.

## Conclusion

This study highlights companies’ continuing efforts to frame their messaging as “information” and “education” rather than “advertising” or “promotion” in ways that serve their interests. The research also calls attention to the industry’s continual efforts to promote self-regulation and internal codes of conduct. This commercially oriented approach to regulation occurs within a highly permissive federal regulatory framework with little concern for serious consequences or violations. It is expected that companies with a vested interest in labeling their messaging as non-promotional will continue to do so. With few changes to Health Canada’s approach to advertising, and little reason for the industry to change its own efforts to stretch the boundaries of “advertising”, it appears that the same factors leading to the mischaracterization of opioid advertising remain for health products, including all other classes of prescription drugs, at this time. It is in the public interest, however, to ensure that third parties determine these categorizations, rather than industry itself.

## Supporting information

S1 File This file contains all the supporting files. (PDF)
