# Supplementary material for: Pharmaceutical company responses to Canadian opioid advertising restrictions: A framing analysis
Source: PLoS One. 2023 Jun 29;18(6):e0287861. doi: 10.1371/journal.pone.0287861 (PMC10310031; doi:10.1371/journal.pone.0287861)
Supplement: S1 File — (PDF) [file pone.0287861.s001.pdf]

Expert Advisory Group on the Marketing of Opioids
Summary of Meeting #1
August 16, 2018

Members in attendance:
Dr. Irfan Dhalla
Dr. Barbara Mintzes
Dr. Andreas Laupacis

Regrets:
Dr. Trudo Lemmens

Health Canada (HC) officials:
Ed Morgan, Director General (Chair)
Amanda Moir, Director
Lissa Murseli, Manager
Laura Etherden, Senior Policy Analyst
Jessica Faubert, Senior Policy Analyst
Jackie Cote, Policy Analyst

Summary of Discussion:

1. **Welcome and Introductions**
    - Members introduced themselves.
    - HC provided an overview of the signed confidentiality agreements, highlighting that information created or shared as part of the Advisory Group's (AG) work must be kept confidential.
    - A draft forward agenda was presented, noting that it would continue to be updated as the work of the Advisory Group progresses.

2. **Terms of Reference**
    - HC confirmed the scope of the Advisory Group is on the marketing and advertising of opioids.
    - Members approved the Terms of Reference.

3. **Background Information**
    - Health Canada provided an overview of the:
        - **Openness and transparency policy**, identifying that the Terms of Reference, member biographies and summary of affiliations would be posted online.
        - **Drug review process and the current regulatory system**
            - Members expressed interest in further details about the new regulatory requirement for risk management plans for opioids, and compliance enforcement of complaints. HC noted the Marketed Health Products Directorate would be invited to the next meeting.
            - HC clarified the maximum monetary fines for inappropriate advertising under Vanessa's Law is $5M.
            - Members highlighted that while the scope of the AG is related to opioids, non-opioid drugs can also have risks.

- **Marketing and advertising activities** and the types of health professionals exposed to them.
  - Members highlighted the role of new media and how it is changing the way prescribers obtain information about medications.
- **Summary of evidence**
  - Members noted that transparency and disclosure of financial payments or incentives is a valuable initiative, but would be insufficient on its own.

4. Key Points of Discussion

- It was noted that there has been some shift from direct marketing activities to indirect marketing (such as peer-to-peer marketing and funding of organizations).
- Through existing literature and identified gaps, the AG discussed what is known about the link between opioid marketing practices and the current opioid crisis.
- To encourage appropriate prescribing, it was noted that there is a need for drug information that is concise, unbiased, and easily accessible to health professionals.
- It was noted that industry provision of information to clinicians (for example, through continuing medical education and guideline development) presents a conflict of interest. It was suggested that independent third parties would be better placed to provide this information.
- It was noted that imbedding evidence-based practices in clinical workflow can be challenging for health professionals.
- Members indicated that health professionals need information on:
  - How to appropriately taper patients off opioids;
  - Support available beyond gradual dose reduction, including access to psychological support and other professionals;
  - New drugs, which would increase awareness of options;
  - What is known, and what isn't known, about a health product; and
  - How to maximize benefits and minimize harms, without requiring the health professional to consult primary literature.

5. Roundtable and Closing Remarks

- HC thanked members for their participation.
- HC completed administrative items such as proposing the date for the next meeting and requesting members submit biographies for HC to post online.

Groupe consultatif d'experts sur le marketing des opioïdes
Compte rendu de la première réunion
16 août 2018

Membres présents
Dr Irfan Dhalla
Barbara Mintzes, Ph. D.
Dr Andreas Laupacis

Absents
Trudo Lemmens, Ph. D.

Représentants de Santé Canada (SC)
Ed Morgan, directeur général (président)
Amanda Moir, directrice
Lissa Murseli, gestionnaire
Laura Etherden, analyste principale des politiques
Jessica Faubert, analyste principale des politiques
Jackie Cote, analyste des politiques

Résumé des discussions

1. **Mot de bienvenue et présentations**
   - Les membres se présentent à leurs collègues.
   - SC présente un aperçu des ententes de confidentialités signées, soulignant que l'information créée ou partagée dans le cadre du travail du Groupe consultatif doit demeurer confidentielle.
   - Une ébauche d'ordre du jour des prochaines réunions est présentée, en notant qu'il continuera d'être mis à jour au fil de la progression des travaux du Groupe consultatif.

2. **Mandat**
   - SC confirme que les travaux du Groupe consultatif portent sur le marketing des opioïdes et la publicité sur les opioïdes.
   - Les membres approuvent le mandat.

3. **Renseignements généraux**
   - Santé Canada présente un aperçu des points suivants :
     - **Politique d'ouverture et de transparence :** Il est souligné que le mandat, les biographies des membres et le résumé des affiliations seront publiés en ligne.
     - **Processus d'examen des médicaments et système réglementaire actuel**
       - Les membres demandent des précisions sur la nouvelle exigence réglementaire en matière de plans de gestion des risques pour les opioïdes, et sur la conformité et l'application de loi en matière de plaintes. SC souligne que la Direction des produits de santé commercialisés sera invitée à la prochaine réunion.
       - SC précise que l'amende maximale pour publicités inappropriées en vertu de la *Loi de Vanessa* est de 5 millions de dollars.

        - Les membres soulignent que même si les travaux du Groupe consultatif portent sur les opioïdes, les médicaments non opioïdes peuvent également comporter des risques.
    - **Activités de marketing et de publicité** et types de professionnels de la santé qui y sont exposés.
        - Les membres soulignent le rôle des nouveaux médias et comment ceux-ci changent la façon dont les prescripteurs obtiennent des renseignements sur les médicaments.
    - **Résumé des preuves**
        - Les membres soulignent que la transparence et la divulgation de paiements et d'incitatifs financiers sont une initiative utile, mais insuffisante en soi.

4. Principaux points de discussion

- Il est mentionné qu'il y a eu un certain déplacement des activités de marketing direct vers le marketing indirect (p. ex. le marketing entre pairs et le financement d'organisations).
- En se fondant sur la documentation existante et les lacunes connues, le Groupe consultatif discute de ce qui est connu sur le lien entre les pratiques de marketing des opioïdes et la crise des opioïdes actuelle.
- Pour encourager des pratiques de prescription appropriées, il est souligné que les renseignements sur les médicaments doivent être concis, non biaisés et facilement accessibles aux professionnels de la santé.
- Il est mentionné que les renseignements fournis par l'industrie et destinés aux cliniciens (p. ex. au moyen de la formation médicale continue et de l'élaboration de lignes directrices) donnent lieu à un conflit d'intérêts. Il est suggéré que des tiers indépendants seraient mieux placés pour fournir ces renseignements.
- On souligne que l'intégration de pratiques fondées sur des éléments probants dans la pratique clinique peut poser des problèmes aux professionnels de la santé.
- Les membres indiquent que les professionnels de la santé ont besoin de renseignements sur ce qui suit :
    - Comment sevrer des patients des opioïdes de manière appropriée;
    - Le soutien disponible au-delà d'une réduction graduelle de la dose, y compris l'accès à une aide psychologique et d'autres soins professionnels;
    - De nouveaux médicaments, ce qui améliorerait la connaissance des possibilités;
    - Ce qui est connu et qui ne l'est pas à propos d'un produit de santé;
    - Comment maximiser les bienfaits et limiter les préjudices, sans obliger les professionnels de la santé à consulter la documentation spécialisée.

5. Table ronde et mot de la fin

- SC remercie les membres de leur participation.
- SC règle des points administratifs, entre autres pour proposer la date de la prochaine réunion et demander aux membres de soumettre leur biographie pour publication en ligne par SC.

Expert Advisory Group on the Marketing of Opioids
Summary of Meeting #2
August 29, 2018

Members in attendance:
Dr. Irfan Dhalla
Dr. Barbara Mintzes
Dr. Trudo Lemmens

Regrets:
Dr. Andreas Laupacis

Health Canada (HC) officials:
Ed Morgan, Director General (Chair)
Lissa Murseli, Manager
Jessica Faubert, Senior Policy Analyst
Jackie Côté, Policy Analyst
Ashley Zakhem, Policy Analyst
Alain Musende, Manager (presenter)
David Lee, Chief Regulatory Officer (presenter)
Karen Leblanc, Senior Policy Analyst (observer)

Summary of Discussion:

1. **Opening remarks and agenda**
   - HC presented the agenda and forward agenda for comments.
   - Members noted the recent announcement that British Columbia would pursue legal action against opioid manufacturers.

2. **Approval of meeting minutes**
   - Members approved minutes of August 16, 2018.

3. **Q&A on opioid advertising regulations, compliance monitoring and enforcement**
   - HC provided a summary of how advertising complaints are received and handled.
   - For opioids, an advertising complaint is received and investigated. If non-compliance is found, Health Canada notifies the company to immediately cease that activity. If compliance is not achieved wilfully, further compliance actions can include injunction or prosecution.
   - HC also noted that it is taking a more proactive approach to monitoring advertising and marketing.
   - The published "Distinction Between Advertising and Other Activities" document was noted as a relevant policy to clarify the distinction between advertising activities and other activities that are not primarily intended to promote the sale of a drug.

4. **Opioid Symposium consultation**
   - Health Canada shared the presentation planned for the Restricting Marketing and Advertising of Opioids session on September 5 at the Opioid Symposium in Toronto.

5. Key Points of Discussion

- Members discussed pharmaceutical advertising activities, their potential influence and usefulness. Overall, members noted the complexities of these activities and the subtle ways information is portrayed by industry to health professionals. While certain activities may not be viewed as advertisements, they can be considered a kind of marketing/promotion.

a. Advertisements (e.g. journals and on the internet)

- It was noted that while advertisements in journals may not be the main influential promotional activity, they are a clear indication of companies' branding and messaging and they can be influential.

b. Materials generated by industry, specific to a product (e.g. brochures, posters, pamphlets)

- It was noted that material that is approved/pre-cleared and has been determined to be non-promotional could be considered separate from other types of industry-generated materials that are promotional.
- Members noted that an area where information and training should be available to prescribers is related to opioid use disorder treatments (e.g. suboxone; methadone).

c. Material generated by a third party and disseminated by industry (e.g. peer-reviewed articles, research publications, clinical guidelines, textbooks)

- It was noted that distribution of third-party information is a category of materials that is unique from material generated by industry; therefore, it warrants careful consideration.
- It was noted that independent studies are not always unbiased and can shape opinions.

d. Sales representative visits to provide information or materials, including drug samples

- It was noted there is evidence that sales representative visits can change prescriber behaviour; can lead to lower quality prescribing, overprescribing, and more expensive drug expenditures.

e. Industry emails and mail-outs including promotional messages (e.g. press releases, recruitment of clinical trial participants)

- It was noted that while some clinical trial information is useful, at times they can be used as a marketing activity to make doctors accustomed to prescribing a new drug.

6. Roundtable

- HC thanked members for their participation.
- HC completed administrative items such as proposing the date for the next meeting and requesting member biographies.

Groupe consultatif d'experts sur le marketing des opioïdes
Compte rendu de la deuxième réunion
29 août 2018

Membres présents
Dr Irfan Dhalla
Barbara Mintzes, Ph. D.
Trudo Lemmens, Ph. D.

Absents :
Dr Andreas Laupacis

Représentants de Santé Canada (SC)
Ed Morgan, directeur général (président)
Lissa Murseli, gestionnaire
Jessica Faubert, analyste principale des politiques
Jackie Côté, analyste des politiques
Ashley Zakhem, analyste des politiques
Alain Musende, gestionnaire (présentateur)
David K. Lee, chef de la réglementation (présentateur)
Karen Leblanc, analyste principale des politiques (observatrice)

Résumé des discussions

1. **Mot de bienvenue et ordre du jour**
    - SC présente l'ordre du jour et celui des prochaines réunions pour commentaires.
    - Les membres soulignent l'annonce récente que la Colombie-Britannique intentera des poursuites judiciaires contre les fabricants d'opioïdes.

2. **Approbation du procès-verbal**
    - Les membres approuvent le procès-verbal du 16 août 2018.

3. **Questions et réponses sur la réglementation de la publicité sur les opioïdes, la surveillance de la conformité et les contrôles d'application**
    - SC présente un résumé de la manière dont les plaintes liées à la publicité sont reçues et traitées.
    - Dans le cas des opioïdes, la plainte liée à la publicité est reçue et étudiée. Si une non-conformité est constatée, SC avise l'entreprise de cesser l'activité immédiatement. Si la conformité n'est pas atteinte volontairement, d'autres mesures de conformité peuvent comprendre notamment une injonction ou une poursuite.
    - SC précise qu'il aborde la surveillance de la publicité et du marketing de manière proactive.
    - Le document publié sous le nom « Distinction entre les activités publicitaires et les autres activités » est désigné comme une politique pertinente pour préciser la distinction entre les activités publicitaires et d'autres activités qui ne sont pas principalement destinées à promouvoir la vente d'un médicament.

4. **Consultation concernant le Symposium sur les opioïdes**
   - SC présente l'exposé qu'il doit faire sur la restriction du marketing des opioïdes et de la publicité sur les opioïdes, le 5 septembre, dans le cadre du Symposium sur les opioïdes qui se déroulera à Toronto.

5. **Principaux points de discussion**
   - Les membres discutent des activités publicitaires de l'industrie pharmaceutique et de leurs influence et utilité potentielles. Dans l'ensemble, les membres soulignent la complexité de ces activités et la façon subtile dont les renseignements sont transmis par l'industrie aux professionnels de la santé. Bien que certaines activités ne soient pas perçues comme des publicités, on peut les considérer comme une sorte de marketing ou de promotion.

   a. **Publicités (p. ex. revues et pages Web)**
      - Il est noté que même si les publicités dans les revues scientifiques ne constituent sans doute pas la principale activité de promotion qui exerce une influence, elles sont une indication claire de l'image de marque d'une entreprise et des messages qu'elles véhiculent et peuvent exercer une influence.

   b. **Les documents produits par l'industrie portant sur un produit (p. ex. brochures, affiches, dépliants)**
      - Il est souligné que les documents approuvés ou préapprouvés, jugés non promotionnels pourraient être distingués d'autres types de documents produits par l'industrie, qui sont promotionnels.
      - Les membres soulignent qu'un domaine où des renseignements et des formations devraient être disponibles aux prescripteurs concerne les traitements des troubles liés à la consommation d'opioïdes (p. ex. suboxone; méthadone).

   c. **Documents produits par un tiers et diffusés par l'industrie (p. ex. articles évalués par des pairs, publications de recherche, guides de pratique clinique, manuels)**
      - Il est souligné que la distribution de renseignements de tiers constitue une catégorie de documents qui se distinguent de documents produits par l'industrie et méritent donc une attention particulière.
      - Il est noté que les études indépendantes ne sont pas toujours objectives et qu'elles peuvent influencer les opinions.

   d. **Visites de représentants commerciaux pour fournir de l'information ou des documents, notamment des échantillons de médicaments**
      - Il est mentionné que les données montrent que les visites de représentants commerciaux peuvent modifier les comportements des prescripteurs et peuvent entraîner une réduction de la qualité des pratiques de prescription, une prescription excessive et des dépenses en médicaments plus élevées.

   e. **Courriels et envois postaux de l'industrie (p. ex. communiqués de presse, recrutement de participants à des essais cliniques)**
      - Il est souligné que même si certains renseignements sur les essais cliniques sont utiles, ils peuvent parfois servir à des fins de marketing pour habituer les médecins à prescrire un nouveau médicament.

6. Table ronde

- SC remercie les membres de leur participation.
- SC règle des points administratifs, entre autres pour proposer la date de la prochaine réunion et demander aux membres de soumettre leur biographie.

Expert Advisory Group on the Marketing of Opioids
Summary of Meeting #3
September 27, 2018

Members in attendance:
Dr. Andreas Laupacis
Dr. Trudo Lemmens
Dr. Barbara Mintzes

Regrets:
Dr. Irfan Dhalla

Health Canada (HC) Officials:
Ed Morgan, Director General (Chair)
Amanda Moir, Director
Lissa Murseli, Manager
Jackie Côté, Policy Analyst
David Lee, Chief Regulatory Officer (presenter)
Karen Leblanc, Senior Policy Analyst (observer)

Summary of Discussion:

1. **Opening remarks and agenda**
   - HC presented the agenda for comments.

2. **Approval of meeting minutes**
   - Members approved the minutes of August 29, 2018.

3. **Openness and transparency: web content**
   - HC proposed that high level summaries of Advisory Group meetings would be posted online as part of Advisory Group's approach to openness and transparency. Members were supportive of sharing the summaries online.

4. **Summary of Comments from Notice of Intent and Opioid Symposium**
   - HC gave a summary of the feedback received for the Notice of Intent that was open for comment in June and July. HC noted that responses were received from over 40 organizations, companies, and individuals as part of these consultations. Stakeholders were generally supportive of restricting marketing and advertising of opioids, but noted there should be exemptions or considerations for educational materials and opioid use disorder medications. A What We Heard report will be published in the fall.
   - HC gave a summary of the Opioid Symposium session. Comments from stakeholders included suggestions to address marketing and advertising of prescription drugs broadly, the conflict of interest presented by industry's role in medical education, and concerns that marketing restrictions could lead to prescriber reluctance to prescribe opioids.
   - HC will continue to engage the medical and educational communities to gather further comments.

5. **Federal regulatory and legislative authorities**
   - HC provided background on the current legislative framework in the *Food and Drugs Act* and the *Controlled Drugs and Substances Act*'s *Narcotic Control Regulations*, the roles

of federal vs. provincial and territorial governments, and new authorities under Vanessa's Law which allow the Minister to apply terms and conditions to the sale of a specific drug by a specific company.

6. **Key Points of Discussion**
   - Members discussed international models aimed at increasing transparency of pharmaceutical industry's interaction with health professionals, including potential effectiveness at reducing industry influence on prescribing.
   - Members noted the importance of considering that scope and level of detail of activities, such as clearly defining who are payors and/or recipients.
   - Members noted that transparency requirements make data on payments available so that researchers can analyse impacts. In addition, these requirements can encourage industry to reassess and modify their practices.
   - Members suggested that successful models do the following:
     - Account for the complexity of drug marketing;
     - Include transparency measures that require industry to disclose the product that is being marketed, in addition to the value amount transferred;
     - Proactively review marketing materials before they are used in promotions;
     - Make public any non-compliances and compliance and enforcement activities; and
     - Make data available in a user-friendly way so that researchers can analyse results. This data should be broad enough to capture financial relationships with all relevant institutions, including patient organizations and research institutions.

7. **Roundtable and closing remarks**
   - HC completed administrative items such as proposing the timing of the next meeting.

Groupe consultatif d'experts sur le marketing des opioïdes
Compte rendu de la troisième réunion
27 septembre 2018

Membres présents
Dr Andreas Laupacis
Trudo Lemmens, Ph. D.
Barbara Mintzes, Ph. D.

Absents :
Dr Irfan Dhalla

Représentants de Santé Canada (SC)
Ed Morgan, directeur général (président)
Amanda Moir, directrice
Lissa Murseli, gestionnaire
Jackie Côté, analyste des politiques
David K. Lee, chef de la réglementation (présentateur)
Karen Leblanc, analyste principale des politiques (observatrice)

Résumé des discussions

1. **Mot de bienvenue et ordre du jour**
    - SC présente l'ordre du jour pour commentaires.

2. **Approbation du procès-verbal**
    - Les membres approuvent le procès-verbal du 29 août 2018.

3. **Ouverture et transparence : contenu Web**
    - SC propose que des résumés de haut niveau de réunions du Groupe consultatif soient affichés en ligne dans une optique d'ouverture et de transparence. Les membres appuient la diffusion de résumés en ligne.

4. **Résumé des commentaires sur l'avis d'intention et le Symposium sur les opioïdes**
    - SC présente un résumé des commentaires reçus concernant l'avis d'intention qu'on avait la possibilité de commenter en juin et juillet. SC remarque que des réponses ont été reçues de plus de 40 organisations, entreprises et individus dans le cadre de ces consultations. Les intervenants appuyaient généralement les restrictions sur le marketing et la publicité à l'égard des opioïdes, mais ils soulignaient qu'il devrait y avoir des dispenses ou des considérations pour le matériel éducatif et les médicaments pour traiter les troubles liés à la consommation d'opioïdes. Un rapport Ce que nous avons entendu sera publié à l'automne.
    - SC présente un résumé du Symposium sur les opioïdes. Les intervenants formulent des commentaires, parmi lesquels des suggestions pour résoudre les questions du marketing et de la publicité à l'égard des médicaments d'ordonnance de façon élargie, du conflit d'intérêts sous-jacent au rôle de l'industrie dans l'éducation médicale et des préoccupations à l'idée que les restrictions sur le marketing pourraient entraîner une réticence de la part des prescripteurs à prescrire des opioïdes.
    - SC continuera de consulter les milieux de la santé et de l'éducation médicale pour obtenir d'autres commentaires.

5. **Pouvoirs réglementaires et législatifs du gouvernement fédéral**
   - SC présente le contexte du cadre législatif actuel de la *Loi sur les aliments et drogues* et du *Règlement sur les stupéfiants* de la *Loi réglementant certaines drogues et autres substances*, le rôle du gouvernement fédéral par rapport à celui des gouvernements provinciaux et territoriaux et les nouveaux pouvoirs en vertu de la *Loi de Vanessa* qui permettent au ministre d'appliquer des modalités et conditions à la vente d'un médicament particulier par une entreprise particulière.

6. **Principaux points de discussion**
   - Les membres discutent de modèles internationaux visant à accroître la transparence dans l'interaction de l'industrie pharmaceutique avec les professionnels de la santé, y compris l'efficacité éventuelle d'une réduction de l'influence de l'industrie sur les pratiques de prescription.
   - Les membres soulignent l'importance de considérer la portée et le degré de détail des activités, par exemple en définissant clairement qui sont les payeurs et les bénéficiaires.
   - Les membres soulignent que les exigences de transparence rendent les données sur les paiements disponibles pour que les chercheurs puissent analyser l'incidence. De plus, ces exigences peuvent inciter l'industrie à réévaluer et à modifier ses pratiques.
   - Les membres suggèrent que les modèles retenus effectuent ce qui suit :
     - Tenir compte de la complexité du marketing des médicaments;
     - Inclure des mesures de transparence qui exigent que l'industrie divulgue le produit qui fait l'objet de marketing, en plus du montant transféré;
     - Examiner de façon proactive les documents de marketing avant qu'ils soient utilisés dans des promotions;
     - Rendre publiques toutes non-conformités et activités de conformité et d'application de la loi;
     - Rendre les données disponibles d'une façon conviviale, pour que les chercheurs puissent analyser les résultats. La portée de ces données devrait être suffisamment élargie pour rendre compte des relations financières avec toutes les institutions pertinentes, y compris les organisations de patients et les établissements de recherche.

7. **Table ronde et mot de la fin**
   - SC règle des points administratifs, entre autres pour proposer la date de la prochaine réunion.

Summary of Findings and Advice: Expert Advisory Group on the Marketing of Opioids

Context

The opioid crisis is one of Canada's most serious public health crises in recent memory, with a growing number of opioid-related overdoses and deaths. Recent data confirms that over the last three years there were over 10,300 documented apparent opioid-related deaths in Canada.

The factors contributing to the opioid crisis are complex, touching on a range of often interrelated health, social and economic issues. Addressing the crisis will involve concerted efforts across the country from governments, stakeholders and others. For its part, the federal government is working with provinces, territories and other partners on numerous fronts to take action in areas related to prevention, treatment, harm reduction, and enforcement supported by a strong evidence base.

While the factors contributing to the opioid crisis are complex, prescriptions written by health care professionals are a common source of opioids in Canada. Prescription opioids can help Canadians who need them manage pain. However, there is growing consensus that the over-prescribing of opioids has contributed to Canada's opioid crisis.

Health care professionals receive information from a variety of sources to inform their prescribing decisions and advice to patients, including from the pharmaceutical industry. While there may be value in the pharmaceutical industry conveying educational and scientific information about a health product, pharmaceutical companies are not neutral marketing information providers, as companies have an interest in product sales and market share. The opioid crisis confirms the need to carefully look at the role industry marketing and advertising of opioids may play in unduly influencing health care professionals and instituting changes to minimize inappropriate industry influence on prescribing.

In this context, in June 2018 Health Canada announced its intent to restrict the marketing and advertising of opioids to health care professionals. The Minister of Health further called on Canadian manufacturers and distributors of opioids to immediately cease the marketing and advertising of opioids to health care professionals, on a voluntary basis, until new restrictions were put in place.

Consistent with these actions, Health Canada has consulted with stakeholders including health care professionals, patients, academics and the pharmaceutical industry on its intent to restrict opioid marketing and advertising and engaged experts to understand the impact of marketing practices on the medical community.

As part of this work, Health Canada established an Expert Advisory Group on the Marketing of Opioids in August 2018. The mandate of the Expert Advisory Group was to

provide timely information and advice to Health Canada on the evidence regarding the pervasiveness, value and influence of pharmaceutical industry marketing of opioids.

The Expert Advisory Group consisted of four members with a mix of expertise in areas related to pharmaceutical marketing and promotion, the governance of pharmaceuticals, evidence-based health care, opioids, and prescribing behaviour. Member backgrounds included health and legal professionals and academic researchers.

From August 2018 to May 2019 the Expert Advisory Group met by teleconference six times to provide Health Canada with information, assess evidence, review and discuss materials, and provide broad strategic advice on issues related to the marketing of opioids. This included discussion of:

- the scope and range of pharmaceutical industry marketing of opioids to health care professionals;
- the influence of industry marketing on prescribing behaviour and patient health; and,
- the effectiveness of measures for oversight of industry marketing and advertising, including measures for increased transparency of industry marketing and advertising to health professionals.

Summary of Discussions

Scope and range of industry marketing practices

The pharmaceutical industry interacts with health care professionals in numerous ways to make them aware of their products, provide prescribing and other medical product information, and promote their products. Industry marketing and promotional techniques can include a range of direct and indirect activities, such as advertisements in medical journals, promotional materials in print and electronic format; the production and distribution of scientific publications (journal articles and text books), some of which are potentially ghost-written by industry-paid authors; industry-funded presentations at conferences and within accredited and non-accredited education programs, and personal visits from sales representatives. Financial incentives and transfers of value through industry sponsorship of events, fees for research, consulting and speaking, reimbursement for travel and hospitality expenses, and gifts of meals, equipment and materials may also be used.

Reports[1] have also documented the influence on research results and medical practice of industry's sponsorship and shaping of research and treatment guidelines, as well as the frequent financial links between companies and members of guideline panels. While

[1] For example: Lundh, A. et al. 2017. Industry sponsorship and research outcome (Review). Cochrane Database of Systematic Reviews, Issue 2.; Ross, Joseph et al. 2012. Promoting Transparency in Pharmaceutical Industry-Sponsored Research. American Journal of Public Health. Vol 102, No.1.; Shnier, Adrienne et al. 2016. Reporting of financial conflicts of interest in clinical practice guidelines: a case study analysis of guidelines from the Canadian Medical Association Infobase. BMC Health Services Research, 16 (383).

there are varying views on industry's role in these activities, some have argued that such activities can also serve as a form of promotion, contributing to marketing strategies.

Throughout the course of their work, members discussed the complexities of marketing activities and the subtle ways information can be portrayed for promotional ends. Advertising and marketing activities can be directed at health professionals such as physicians, nurses and pharmacists, including those in training, as well as organizations such as health professional bodies, patient advocacy groups and continuing professional development organizations.

Specific to opioids, a number of reports[2] have documented manufacturers' marketing practices in the United States. Marketing activities have also been described in documents made public in legal cases[3].

The extent and scope of industry marketing of opioids to health professionals in Canada is not as well documented when compared to the United States. There is, however, evidence to show that payments by opioid manufacturers to doctors in Canada have occasionally been higher per capita than in the United States[4]. Additional case studies also raise concerns about the influence of industry in medical education[5].

Overall, the Expert Advisory Group advised that marketing practices in Canada are likely to have been comparable to those that occurred in the United States. Further, many of the marketing activities, for example those at conferences and professional meetings and in scientific publications, often occur across jurisdictions and target health professionals in different countries.

Influence of marketing on prescribing behaviour

The Expert Advisory Group noted that while health care professionals require accurate information on opioids to support them in clinical decision-making, the broader literature has demonstrated that pharmaceutical companies can use promotional tactics to shape

---

[2] For example: Van Zee, A. 2009. The promotion and marketing of oxycontin: commercial triumph, public health tragedy. Am J. Public Health. 99; and United States General Accounting Office. December 2003. Report to Congressional Requesters. Prescription Drugs: OxyContin Abuse and Diversion and Efforts to Address the Problem. GAO-04-110.

[3] For example: Haffajee, Rebecca L. et al. 2017. Drug Companies' Liability for the Opioid Epidemic. NEJM 377;24. December 14, 2017; 'A blizzard of prescriptions': Documents reveal new details about Purdue's marketing of OxyContin Jan 15, 2019 https://www.statnews.com/2019/01/15/massachusetts-purdue-lawsuit-new-details/; Secret trove reveals bold 'crusade' to make OxyContin a blockbuster. September 22, 2016. https://www.statnews.com/2016/09/22/abbott-oxycontin-crusade/

[4] https://www.thestar.com/news/investigations/2018/05/03/why-did-the-maker-of-oxycontin-pay-canadian-doctors-nearly-three-and-a-half-times-more-money-per-capita-than-it-doled-out-to-us-prescribers.html

[5] Persaud, Navindra 2013 "Questionable content of an industry-supported medical school lecture series: a case study" Journal of Medical Ethics, 40:6, 2014.

health providers' knowledge and influence their attitudes and decision-making (generally leading to more prescribing).

The Expert Advisory Group discussed how evidence demonstrates that industry's marketing of pharmaceuticals tends to influence health care professionals, and that health care professionals are often unaware of the full extent of industry influence and its impact. For example, one study[6] found that although physicians believed themselves to be reliant on scientific evidence, their knowledge of the properties of two drugs was more consistent with messages in promotional information than the scientific literature. Other studies have documented that marketing and promotional information is often used as a source of information about new drugs[7]. Members also noted evidence demonstrates that when health care professionals receive information directly from pharmaceutical companies, they are less likely to follow independent guidelines, prescribe more frequently, and are more likely to prescribe expensive medications[8].

Members also discussed the presentation of promotional information during visits by pharmaceutical sales representatives, citing a study[9] that included a random sample of 129 general practitioners in Vancouver and Montreal and reported on over 800 presentations. Information on benefits was more prominent than information on harms: nearly 40 percent of the time, the doctors reported having received no oral or written information on harms. Nonetheless, most physicians participating in this study judged the quality of the scientific information provided to them by sales representatives to be good.

Recent studies[10] in the United States linking payments to doctors reported under the *Physician Payments Sunshine Act* to prescribing data have found consistent associations between receipt of payments and prescribing patterns, including more prescribing of brand-name drugs, higher cost prescribing, and higher proportions of prescriptions for the promoted brand within a drug class. These patterns existed not only for large scale payments but also for receipt of smaller amounts (e.g., inexpensive meals). These studies have added considerably to the research evidence on the effects

---

[6] Avorn, J. et al. 1982. "Scientific Versus Commercial Sources of Influence on the Prescribing Behavior of Physicians", American Journal of Medicine 73, no.1 (1982); 4-8; Study was referenced in Robertson, Christopher et al. 2012. Effect of Financial Relationships on the Behaviours of Health Care Professionals: A Review of the Evidence. Journal of Law, Medicine & Ethics.

[7] Norris, Pauline et al. 2004. Drug Promotion: what we know, what we have yet to learn. Reviews of materials in the WHO/HAI database on drug promotion. WHO/EDM/PAR/2004.3

[8] Spurling, G. et al 2010. "Information from Pharmaceutical Companies and the Quality, Quantity, and Cost of Physicians' Prescribing: A Systematic Review." PLOS Medicine 7

[9] Mintzes, B. et al. "Pharmaceutical Sales Representatives and Patient Safety: A Comparative Prospective Study of Information Quality in Canada, France and the United States." Journal of General Internal Medicine, 28 (10) October 2013.

[10] For example: Dejong C. et al Pharmaceutical industry-sponsored meals and physician prescribing patterns for Medicare beneficiaries. JAMA Intern Med. 2016:176 (8):114-10; Steinbrook, Robert Physicians, Industry Payments for Food and Beverages, and Drug Prescribing. JAMA May 2, 2017. Volume 317, Number 17; and Yeh, James S et al. (2016) Association of Industry Payments to Physicians With the Prescribing of Brand-name Statins in Massachusetts. JAMA Vol176, No 6.

of drug promotion on prescribing, as they include follow up of many thousands of physicians and hundreds of thousands of payments.

The Expert Advisory Group also examined studies on the influence of payments to health professionals specific to opioids, including emerging research and reports from the United States that found an association between industry marketing of opioids to physicians and increased opioid prescribing[11]. A number of reports[12] have documented the role of opioid manufacturers' marketing practices to encourage physicians to prescribe opioids. This includes an intensive promotional campaign by Purdue Pharmaceuticals linked to the launch of OxyContin™, which has been associated with a significant increase in the prescription of opioids in primary care and encouraging its use for less severe levels of pain that had not previously been treated with opioids, including non-cancer chronic pain conditions. These messages were also provided in manufacturer-sponsored continuing medical education. Marketing activities described in documents made public in legal cases also raise concerns with the role and influence of industry marketing[13].

While there are no similar studies from Canada, members felt that the findings from such studies could reasonably be considered applicable to the Canadian context. Overall, the Expert Advisory Group advised that the research evidence does support an association between both health professionals' reliance on promotional information and industry payments to health professionals and prescribing decisions.

Measures for oversight and increasing transparency of industry marketing

In the course of their work, the Expert Advisory Group discussed the current federal legislative and regulatory framework for industry advertising of opioids to health care professionals, as well as the roles of industry, professional associations, and provincial and territorial governments. This included discussion of:

- The *Food and Drugs Act*, the *Controlled Drugs and Substances Act* and associated regulations, with a focus on advertising definition and rules.
    - Recent requirement for mandatory preclearance of opioid-related materials provided to health care professionals by an external advertising

---

[11] Hadland, Scott et al. 2017. Industry Payments to Physicians for Opioid Products, 2013-2015. AJPH. Vol 107 (9); Hadland, Scott et al. 2018. Association of Pharmaceutical Industry Marketing of Opioid Products to Physicians with Subsequent Opioid Prescribing. JAMA; and Hadland, Scott et al. 2019. Association of Pharmaceutical Industry Marketing of Opioid Products with Mortality from Opioid-Related Overdoses. JAMA 2019; 2(1)

[12] For example: Van Zee, A. 2009. The promotion and marketing of oxycontin: commercial triumph, public health tragedy. Am J. Public Health. 99; and United States General Accounting Office. December 2003. Report to Congressional Requesters. Prescription Drugs: OxyContin Abuse and Diversion and Efforts to Address the Problem. GAO-04-110.

[13] For example: Haffajee, Rebecca L. et al. 2017. Drug Companies' Liability for the Opioid Epidemic. NEJM 377;24. December 14, 2017; 'A blizzard of prescriptions': Documents reveal new details about Purdue's marketing of OxyContin Jan 15, 2019 https://www.statnews.com/2019/01/15/massachusetts-purdue-lawsuit-new-details/; Secret trove reveals bold 'crusade' to make OxyContin a blockbuster. September 22, 2016. https://www.statnews.com/2016/09/22/abbott-oxycontin-crusade/

preclearance agency to ensure promotional material complies with advertising rules.
- Health Canada's approach to compliance and enforcement of advertising rules, including investigation of complaints and measures to prevent and address non-compliant advertising
- Other federal actions undertaken, including updates related to the Minister of Health's call on opioid manufacturers and distributors to voluntarily cease marketing and advertising of opioids and related consultations.
- Initiatives undertaken by industry, professional associations and others to provide guidance and ethical standards for interactions between industry and health professionals, including industry funding and support to health professionals and measures for enhanced transparency

These discussions largely served as context for members' deliberations on the extent, characteristics, value and impact of the marketing and advertising of opioids on health care professionals. However, members raised issues related to the difficulties of monitoring the advertising environment and interpreting compliance with advertising rules. Concerns with the pharmaceutical industry's potential influence upon external bodies tasked with the preclearance of advertising materials were also noted. Members also noted potential limitations to the definition and interpretation of advertising within current legislation to capture the full scope of marketing and promotional activities undertaken by the pharmaceutical industry.

The Expert Advisory Group also reviewed international models aimed at increasing transparency of industry marketing practices, including their effectiveness, merits and challenges. This included consideration of:
- France's *Loi anti-Cadeaux* and *Loi Bertrand* that regulate financial relationships and transparency requirements between industry and health professionals and organizations[14];
- United States' *Physician Payments Sunshine Act*[15] and associated regulations which require industry disclosure of payments and transfer of value given to physicians and teaching hospitals through a national disclosure program;
- Australia's self-regulatory model under Medicine Australia's Code of Conduct, with regulatory oversight from the Australian Competition and Consumer Council, which requires member companies to disclose payments to individual health professionals, patient/consumer groups, and organizations involved in educational meetings and symposia[16];

[14] Loi anti-Cadeaux ("Anti-Gift Law"): article 4113-6 and "The strengthening of health protection for medicinal and health products" Act, also known as *Loi Bertrand.*
[15] The *Physician Payments Sunshine Act* is established as section 6002 of the Patient Protection and Affordable Care Act, and through regulations (Final Rule).
[16] See: Medicines Australia Code of Conduct; and Australian Competition & Consumer Commission's April 24, 2015 Decision

- Measures in the Netherlands[17] that regulate and promote transparency of financial relationships in advertising of medicinal products between industry and health professionals; and,
- Other initiatives undertaken in Europe, such as industry-led disclosure policies, and EU policy directives which recommend limits to industry transfers of value[18].

Members advised that successful models need to:
- account for the complexity of drug marketing activities;
- provide more detailed information clearly identifying the product and intended audience for marketing;
- make data available in a user-friendly way to allow all those interested to analyze the results
- make public any non-compliances, and compliance and enforcement activities; and,
- be implemented in addition to measures that proactively review industry marketing materials before they are disseminated to health professionals.

The Expert Advisory Group also advised that despite the limitations to some international transparency models, they are valuable in making marketing and advertising data publicly available and highly visible, as well as enabling civic society and governmental agencies to further evaluate marketing practices.

Summary of Advice

Members were cognizant of the scale and complexity of the current opioid crisis. They stressed that while industry's marketing of opioids played a role in contributing to the crisis, the marketing of opioids is currently only one part of a large crisis with many different factors.

At the same time, members stressed that the opioid crisis illuminates the role aggressive, diverse and sustained industry marketing activities can play in influencing prescribing behaviour. In particular, members advised that marketing and promotion practices and their impact is not an issue limited to opioids. Issues beyond those pertaining to opioids should be considered to ensure the appropriateness of industry marketing and advertising, and sufficient oversight of drug promotion.

Drawing on the available evidence, the Expert Advisory Group underscored the appropriateness of restricting industry's marketing and advertising activities to health care professionals on opioids.

---

[17] Includes statutory rules contained in the Dutch *Medicines Act*, and a voluntary national transparency register: https://www.transparantieregister.nl/home

[18] See: the European Federation of Pharmaceutical Industries and Associations (EFPIA) Disclosure Code; and the European Union policy directive https://www.ema.europa.eu/en/documents/regulatory-procedural-guideline/directive-2001/83/ec-european-parliament-council-6-november-2001-community-code-relating-medicinal-products-human-use_en.pdf

In particular, the Expert Advisory Group advised that:

- There is a need for drug information that is concise, unbiased, and easily accessible to health care professionals, and such information is an essential support for appropriate prescribing. Independent third parties are best-placed to provide such information.
- Further restricting industry marketing and advertising of opioids would not create an information gap.
- Marketing and advertising should present an accurate and balanced perspective of benefits and harms.
- The temporary call on opioid manufacturers and distributors to voluntarily cease marketing and advertising of opioids should be made permanent.
- Payments and other transfers of value to health professionals should be made public, and some such non-research-related transfers (e.g., gifts of food and drink, subsidized travel for conference attendees, speaker fees and marketing-related consultancies), should be stopped entirely.
- Education, guidance and clinical practice guidelines should be developed in a manner that minimizes industry influence.
- International models exist and are informative, in particular in relation to information on transfers of value to health professionals and organizations.
- Making marketing information publicly available is an important measure that enables further study and better understanding of marketing practices.
- Even though transparency is a crucial step, it alone is insufficient to curb the potential negative impact of marketing.

Résumé des conclusions et des conseils du
Groupe consultatif d'experts sur le marketing des opioïdes

Contexte

La crise des opioïdes constitue l'une des plus graves crises de santé publique qu'ait connue le Canada dans l'histoire récente en raison du nombre croissant de surdoses et de décès attribuables aux opioïdes. Des données récentes confirment qu'au cours des trois dernières années, plus de 10 300 décès apparemment liés aux opioïdes ont été constatés au Canada.

Les facteurs contribuant à la crise des opioïdes sont complexes puisqu'ils découlent d'une gamme de problèmes d'ordre sanitaire, social et économique souvent interdépendants. Les efforts concertés des gouvernements, des intervenants et d'autres parties concernées seront nécessaires pour faire face à la crise. Pour sa part, le gouvernement fédéral travaille sur plusieurs fronts avec les provinces, les territoires et d'autres partenaires pour prendre les mesures qui s'imposent dans les domaines liés à la prévention, au traitement, à la réduction des méfaits et à l'application de la loi en se fondant sur des données probantes solides.

Malgré le fait que les facteurs contribuant à la crise des opioïdes sont complexes, les ordonnances rédigées par les professionnels de la santé sont une source courante d'opioïdes au Canada. Les opioïdes sur ordonnance peuvent aider les Canadiens souffrants à soulager leurs douleurs. Toutefois, un consensus se dégage à l'effet que la surprescription d'opioïdes a contribué à la crise des opioïdes au Canada.

Pour éclairer leurs décisions portant sur le traitement à prescrire et les conseils à donner aux patients, les professionnels de la santé utilisent des renseignements provenant de diverses sources, notamment de l'industrie pharmaceutique. Bien qu'il y ait un intérêt à ce que l'industrie pharmaceutique transmette des renseignements éducatifs et scientifiques sur un produit de santé, les entreprises pharmaceutiques ne sont pas des fournisseurs d'information de marketing neutre puisqu'elles sont intéressées à vendre leurs produits et à acquérir des parts de marché. La crise des opioïdes confirme la nécessité d'examiner soigneusement le rôle que peuvent jouer le marketing et la publicité des opioïdes en influençant indûment les professionnels de la santé, et d'apporter des changements visant à réduire au minimum cette influence de l'industrie sur les prescriptions.

Dans ce contexte, en juin 2018, Santé Canada a annoncé son intention de restreindre le marketing et la publicité des opioïdes à l'intention des professionnels de la santé. La ministre de la Santé a ensuite invité tous les fabricants et distributeurs canadiens d'opioïdes à cesser immédiatement et volontairement leurs activités de marketing et de publicité des opioïdes auprès des professionnels de la santé, jusqu'à ce que de nouvelles restrictions soient mises en place.

Pour donner suite à ces mesures, Santé Canada a mené des consultations auprès d'intervenants, dont des professionnels de la santé, des patients, des universitaires et des représentants de l'industrie pharmaceutique, sur son intention de restreindre le marketing et la publicité des opioïdes, et a engagé des experts pour comprendre l'impact des pratiques de marketing sur la communauté médicale.

Dans le cadre de ces travaux, Santé Canada a mis sur pied un Groupe consultatif d'experts sur le marketing des opioïdes en août 2018. Le mandat du Groupe consultatif d'experts consistait à fournir de l'information et des conseils opportuns à Santé Canada sur la pénétration, l'utilité et l'influence du marketing des opioïdes par l'industrie pharmaceutique.

Le Groupe consultatif d'experts était formé de quatre membres représentant une combinaison d'expertise et d'expérience dans des domaines tels que le marketing et la promotion des produits pharmaceutiques, la gouvernance des produits pharmaceutiques, les soins de santé fondés sur des données probantes, les opioïdes et les pratiques de prescription. Le Groupe comprenait des professionnels de la santé, des juristes et des chercheurs universitaires.

D'août 2018 à mai 2019, le Groupe consultatif d'experts s'est réuni six fois par téléconférence pour transmettre des renseignements à Santé Canada, évaluer les preuves, examiner le matériel et en discuter, et fournir des conseils stratégiques généraux sur des questions liées au marketing des opioïdes. Les discussions ont porté, entre autres, sur :

- L'ampleur et la portée du marketing des opioïdes pratiqué par l'industrie pharmaceutique auprès des professionnels de la santé;
- L'influence du marketing sur les pratiques de prescription et sur la santé des patients;
- L'efficacité des mesures visant à surveiller les activités de marketing et de publicité de l'industrie, y compris les mesures destinées à accroître la transparence du marketing et de la publicité de l'industrie auprès des professionnels de la santé.

Résumé des discussions

Ampleur et portée des pratiques de marketing de l'industrie

L'industrie pharmaceutique interagit de nombreuses façons avec les professionnels de la santé afin de les renseigner sur ses produits et d'en faire la promotion, et de leur fournir de l'information posologique et sur les produits médicaux. Les techniques de marketing et de promotion de l'industrie peuvent inclure tout un éventail d'activités directes et indirectes telles que des annonces publicitaires dans des revues médicales; du matériel promotionnel imprimé et en format électronique; la production et la distribution de publications scientifiques (articles publiés dans des revues et livres de

cours, dont certains sont probablement écrits par des rédacteurs anonymes rémunérés par l'industrie); des présentations commanditées par l'industrie lors de conférences et dans le cadre de programmes d'éducation agréés et non agréés; et des visites individuelles de représentants des ventes. La commandite d'événements; des honoraires pour la recherche, la consultation et les conférences; le remboursement des frais de déplacement et d'hospitalité; et des dons de repas, d'équipement et de matériel sont d'autres incitatifs financiers et transferts de valeur auxquels l'industrie a recours.

Des rapports[1] ont également démontré l'influence exercée sur les résultats de recherche et la pratique médicale par les commandites de l'industrie et par l'orientation qu'elle donne aux directives en matière de recherche et de traitement, ainsi que les nombreux liens financiers qui existent entre les entreprises et les membres des groupes d'experts pour l'élaboration des directives. Alors que les opinions diffèrent quant au rôle de l'industrie dans ces activités, certains ont fait valoir que de telles activités peuvent également servir d'outils promotionnels contribuant aux stratégies de marketing.

Tout au long de leurs travaux, les membres ont discuté des complexités des activités de marketing et des façons subtiles dont l'information peut être présentée à des fins promotionnelles. Les activités de publicité et de marketing peuvent être destinées aux professionnels de la santé tels que médecins, infirmières et pharmaciens, y compris ceux qui sont en formation, ainsi qu'à des organisations telles qu'associations de professionnels de la santé, groupes de défense des intérêts des patients et organismes de perfectionnement professionnel continu.

Quant aux opioïdes en particulier, de nombreux rapports[2] ont décrit les pratiques de marketing des fabricants aux États-Unis. Des activités de marketing ont également été décrites dans des documents rendus publics dans le cadre d'affaires juridiques[3].

L'étendue et la portée des activités de marketing des opioïdes menées par l'industrie auprès des professionnels de la santé au Canada ne sont pas aussi bien documentées que celles pratiquées aux États-Unis. Cependant, des preuves existent à l'effet que les sommes versées par des fabricants d'opioïdes à des médecins du Canada ont parfois

[1] Par exemple : LUNDH, A., et autres. « Le soutien industriel et les résultats des recherches » (revue), *Base des revues systématiques Cochrane*, 2e éd., 2017; ROSS, Joseph, et autres. « Promoting Transparency in Pharmaceutical Industry-Sponsored Research », *American Journal of Public Health*, 2012, vol. 102, no 1; SHNIER, Adrienne, et autres. « Reporting of financial conflicts of interest in clinical practice guidelines: a case study analysis of guidelines from the Canadian Medical Association Infobase », *BMC Health Services Research*, 2016, 16 (383).

[2] Par exemple : VAN ZEE, A. « The promotion and marketing of oxycontin: commercial triumph, public health tragedy », *Am J. Public Health*, 2009, 99; et United States General Accounting Office, « Report to Congressional Requesters. Prescription Drugs: OxyContin Abuse and Diversion and Efforts to Address the Problem », GAO-04-110, déc. 2003.

[3] Par exemple : HAFFAJEE, Rebecca L., et autres. « Drug Companies' Liability for the Opioid Epidemic », NEJM 377;24, 14 déc. 2017; « 'A blizzard of prescriptions': Documents reveal new details about Purdue's marketing of OxyContin », 15 janv. 2019, https://www.statnews.com/2019/01/15/massachusetts-purdue-lawsuit-new-details/; « Secret trove reveals bold 'crusade' to make OxyContin a blockbuster », 22 sept. 2016, https://www.statnews.com/2016/09/22/abbott-oxycontin-crusade/ .

été plus élevées, par personne, que celles versées aux États-Unis[4]. D'autres études de cas suscitent des inquiétudes quant à l'influence exercée par l'industrie sur la formation médicale[5].

De façon générale, le Groupe consultatif d'experts a indiqué qu'il est probable que les pratiques de marketing observées au Canada aient été comparables à celles ayant eu lieu aux États-Unis. En outre, plusieurs activités de marketing, par exemple celles menées lors de conférences et réunions professionnelles ainsi que dans les publications scientifiques, ont souvent lieu dans l'ensemble des provinces et territoires et ciblent les professionnels de la santé de différents pays.

Influence du marketing sur les pratiques de prescription

Le Groupe consultatif d'experts a constaté qu'alors que les professionnels de la santé ont besoin de renseignements précis sur les opioïdes pour les appuyer dans leur prise de décisions cliniques, la documentation plus général a démontré que les entreprises pharmaceutiques peuvent avoir recours à des tactiques promotionnelles pour façonner les connaissances des intervenants en matière de santé et exercer une influence sur leurs attitudes et leur prise de décisions (ce qui les mène généralement à prescrire davantage).

Le Groupe consultatif d'experts a discuté de la façon dont les éléments probants démontrent que le marketing des produits pharmaceutiques pratiqué par l'industrie a tendance à influencer les professionnels de la santé et que ces derniers n'ont souvent pas conscience de l'ampleur de l'influence exercée par l'industrie ni de ses répercussions. Par exemple, une étude[6] a conclu que même si les médecins croyaient se fier aux preuves scientifiques, leur connaissance des propriétés de deux médicaments correspondait davantage aux messages contenus dans le matériel promotionnel qu'à ce qu'on pouvait lire dans les ouvrages scientifiques. D'autres études ont permis d'établir que le matériel de marketing et de promotion est souvent utilisé comme source d'information sur les nouveaux médicaments[7]. Les membres ont également constaté que les éléments probants démontrent que lorsque les professionnels de la santé reçoivent l'information directement des entreprises pharmaceutiques, ils sont moins enclins à suivre les directives indépendantes,

---

[4] https://www.thestar.com/news/investigations/2018/05/03/why-did-the-maker-of-oxycontin-pay-canadian-doctors-nearly-three-and-a-half-times-more-money-per-capita-than-it-doled-out-to-us-prescribers.html

[5] PERSAUD, Navindra. « Questionable content of an industry-supported medical school lecture series: a case study », *Journal of Medical Ethics*, 40:6, 2014.

[6] AVORN, J., et autres. « Scientific Versus Commercial Sources of Influence on the Prescribing Behavior of Physicians", *American Journal of Medicine*, 73, n° 1, 1982, 4-8. Cette étude a été mentionnée dans ROBERTSON, Christopher, et autres. « Effect of Financial Relationships on the Behaviours of Health Care Professionals: A Review of the Evidence », *Journal of Law, Medicine & Ethics*, 2012.

[7] NORRIS, Pauline, et autres. « Drug Promotion: what we know, what we have yet to learn. Reviews of materials in the WHO/HAI database on drug promotion », WHO/EDM/PAR/2004.3, 2004.

prescrivent plus fréquemment, et sont plus susceptibles de prescrire des médicaments coûteux[8].

Les membres ont également discuté de la présentation du matériel promotionnel lors des visites réalisées par les représentants des ventes des entreprises pharmaceutiques, citant une étude[9] qui comportait un échantillon aléatoire de 129 médecins généralistes de Vancouver et de Montréal et qui faisait état de 800 présentations. Les renseignements portant sur les bienfaits étaient plus nombreux que ceux concernant les effets néfastes : près de 40 pour cent du temps, les médecins indiquaient n'avoir reçu aucune information orale ou écrite sur les méfaits des médicaments. Néanmoins, la plupart des médecins ayant participé à cette étude ont déclaré avoir été satisfaits de la qualité de l'information scientifique que leur ont transmise les représentants pharmaceutiques.

Des études récentes[10] menées aux États-Unis reliant les sommes versées aux médecins signalées en vertu du *Physician Payments Sunshine Act* aux données sur les prescriptions ont permis d'établir qu'il existe des liens systématiques entre la réception de paiements et les tendances de prescription, y compris davantage de prescriptions de médicaments de la marque, des prescriptions plus coûteuses, et des proportions plus élevées de prescriptions de la marque ayant fait l'objet d'une promotion au sein d'une classe de médicaments. Ces tendances étaient observables non seulement lorsque de généreuses sommes avaient été versées, mais également lorsque les paiements étaient moins élevés (p. ex. repas peu coûteux). Ces études ont considérablement contribué à établir des éléments probants de recherche sur les effets de la promotion des médicaments sur les prescriptions puisqu'elles comportent le suivi de milliers de médecins et de centaines de milliers de paiements.

Le Groupe consultatif d'experts a également examiné des études portant sur l'influence des paiements faits aux professionnels de la santé et liés particulièrement aux opioïdes, dont des recherches émergentes et des rapports des États-Unis indiquant le lien qui existe entre le marketing des opioïdes auprès des médecins et la hausse du nombre de prescriptions d'opioïdes[11]. De nombreux rapports[12] ont documenté le rôle des pratiques

---

[8] SPURLING, G., et autres. « Information from Pharmaceutical Companies and the Quality, Quantity, and Cost of Physicians' Prescribing: A Systematic Review », *PLOS Medicine* 7, 2010.

[9] MINTZES, B., et autres. « Pharmaceutical Sales Representatives and Patient Safety: A Comparative Prospective Study of Information Quality in Canada, France and the United States », *Journal of General Internal Medicine*, 28 (10) octobre 2013.

[10] Par exemple : DEJONG, C., et autres. « Pharmaceutical industry-sponsored meals and physician prescribing patterns for Medicare beneficiaries », *JAMA Intern Med*, 2016:176 (8):114-10; STEINBROOK, Robert. « Physicians, Industry Payments for Food and Beverages, and Drug Prescribing », *JAMA*, 2 mai 2 2017, vol. 317, n° 17; et YEH, James S., et autres. « Association of Industry Payments to Physicians With the Prescribing of Brand-name Statins in Massachusetts », *JAMA*, 2016, vol. 176, n° 6.

[11] HADLAND, Scott, et autres. « Industry Payments to Physicians for Opioid Products, 2013-2015 », *AJPH*, 2017, vol. 107 (9); HADLAND, Scott, et autres. « Association of Pharmaceutical Industry Marketing of Opioid Products to Physicians with Subsequent Opioid Prescribing », *JAMA*, 2018; et HADLAND, Scott, et autres. « Association of Pharmaceutical Industry Marketing of Opioid Products with Mortality from Opioid-Related Overdoses », *JAMA*, 2019, 2(1).

de marketing des fabricants d'opioïdes pour inciter les médecins à prescrire des opioïdes. Ces pratiques incluent une vaste campagne promotionnelle organisée par Purdue Pharmaceuticals et liée au lancement de l'OxyContin^MC, qui a été associé à une augmentation considérable du nombre de prescriptions d'opioïdes en soins de base et dont l'emploi a été encouragé pour des douleurs moins intenses qui n'avaient jamais été traitées avec des opioïdes auparavant, y compris les douleurs chroniques non cancéreuses. Ces messages étaient également transmis dans le cadre de la formation médicale continue commanditée par les fabricants. Les activités de marketing décrites dans les documents rendus publics dans le contexte d'affaires juridiques ont également suscité des inquiétudes quant au rôle et à l'influence du marketing de l'industrie[13].

Même s'il n'existe pas d'études semblables en provenance du Canada, les membres ont estimé que les conclusions de telles études pouvaient raisonnablement être considérées applicables au contexte canadien. Dans l'ensemble, le Groupe consultatif d'experts a constaté que les données des recherches concluent à la fois à un lien entre le fait que les professionnels de la santé ont recours à l'information promotionnelle qui leur est fournie par l'industrie et les sommes qu'elle leur verse, et les décisions prises en matière de prescriptions.

Mesures relatives à la surveillance et à l'amélioration de la transparence du marketing de l'industrie

Au cours de ses travaux, le Groupe consultatif d'experts a discuté du cadre législatif et réglementaire fédéral actuel concernant la publicité sur les opioïdes faite par l'industrie auprès des professionnels de la santé, ainsi que des rôles de l'industrie, des associations professionnelles, et des gouvernements des provinces et territoires. Les discussions ont porté, entre autres, sur :

- La *Loi sur les aliments et les drogues*, la *Loi réglementant certaines drogues et autres substances* et les règlements connexes, en mettant l'accent sur la définition de la publicité et les règles qui la régissent;
    - Les récentes exigences relatives à la pré-approbation du matériel lié aux opioïdes fourni aux professionnels de la santé par un organisme de pré-approbation de la publicité externe en vue de s'assurer que le matériel promotionnel est conforme aux règles de publicité;

---

[12] Par exemple : VAN ZEE, A. « The promotion and marketing of oxycontin: commercial triumph, public health tragedy », *Am J. Public Health*, 99; et United States General Accounting Office, « Report to Congressional Requesters. Prescription Drugs: OxyContin Abuse and Diversion and Efforts to Address the Problem », GAO-04-110, déc. 2013.

[13] Par exemple : HAFFAJEE, Rebecca L., et autres. « Drug Companies' Liability for the Opioid Epidemic », *NEJM*, 377;24, 14 déc. 2017; « A blizzard of prescriptions: Documents reveal new details about Purdue's marketing of OxyContin », 15 janv. 2019. https://www.statnews.com/2019/01/15/massachusetts-purdue-lawsuit-new-details/; « Secret trove reveals bold 'crusade' to make OxyContin a blockbuster », 22 sept. 2016. https://www.statnews.com/2016/09/22/abbott-oxycontin-crusade/

- L’approche adoptée par Santé Canada en matière de conformité et d’application de la loi régissant la publicité, y compris l’examen des plaintes et des mesures prises pour prévenir la publicité non conforme et y remédier;
- Les autres mesures adoptées par le gouvernement fédéral, y compris des mises à jour relatives à l’appel de la ministre de la Santé aux fabricants et distributeurs d’opioïdes leur demandant de cesser volontairement leurs activités de marketing et de publicité et autres consultations connexes;
- Les initiatives prises par l’industrie, les associations professionnelles et autres pour fournir une orientation et établir des normes d’éthique encadrant les interactions entre l’industrie et les professionnels de la santé, y compris le financement de l’industrie et l’appui accordé aux professionnels de la santé, ainsi que les mesures adoptées pour améliorer la transparence.

Ces discussions ont servi de cadre aux délibérations des membres sur l’étendue, les caractéristiques, la valeur et les répercussions du marketing et de la publicité des opioïdes auprès des professionnels de la santé. Toutefois, les membres ont soulevé des enjeux liés aux difficultés de suivi de l’environnement publicitaire et à l’interprétation de la conformité aux règles régissant la publicité. Des préoccupations ont été exprimées quant à l’influence potentielle que l’industrie pharmaceutique peut exercer sur des organismes externes qui sont chargés de la pré-approbation du matériel publicitaire. Les membres ont également constaté des limites éventuelles en ce qui a trait à la définition et à l’interprétation de la publicité dans la législation en vigueur pour saisir toute la portée des activités de marketing et de publicité menées par l’industrie pharmaceutique.

Le Groupe consultatif d’experts a également examiné les modèles internationaux visant à améliorer la transparence des pratiques de marketing de l’industrie, y compris leur efficacité, leur mérite et leurs défis. Les modèles examinés comprenaient :

- La *Loi anti-cadeaux* et la *Loi Bertrand* en vigueur en France, qui réglementent les relations financières et les exigences en matière de transparence entre l’industrie et les organisations et professionnels de la santé[14];
- Le *Physician Payments Sunshine Act*[15] des États-Unis et les règlements connexes, qui exigent de l’industrie de divulguer, dans le cadre d’un programme national de divulgation, les paiements et transferts de valeur qu’elle octroie aux médecins et aux hôpitaux universitaires;
- Le modèle d’autoréglementation de l’Australie en vertu du *Medicines Australia Code of Conduct*, avec la surveillance réglementaire de l’Australian Competition and Consumer Council, qui exige que les entreprises membres divulguent les paiements octroyés aux professionnels de la santé, groupes de

[14] Loi anti-cadeaux (*Anti-Gift Law*), art. 4113-6, et *The strengthening of health protection for medicinal and health products Act*, également appelé *Loi Bertrand.*

[15] Le *Physician Payments Sunshine Act* est constitué de la section 6002 du « Patient Protection and Affordable Care Act » et de la régulation.

patients/consommateurs et organisations participant à des conférences et symposiums éducatifs[16];

- Les mesures prises par les Pays-Bas[17] réglementant et favorisant la transparence des relations financières entre l'industrie et les professionnels de la santé en matière de publicité des produits médicinaux;
- D'autre initiatives prises en Europe, telles que les politiques de divulgation établies par l'industrie, et les directives en matière de politique de l'UE qui recommandent l'imposition de limites en ce qui a trait aux transferts de valeur pratiqués par l'industrie[18].

Les membres ont conclu que les modèles efficaces doivent :

- Tenir compte de la complexité des activités de marketing des médicaments;
- Fournir des renseignements plus détaillés identifiant clairement le produit et le public visé par les activités de marketing;
- Rendre les données disponible de façon conviviale afin que les personnes intéressées puissent analyser les résultats;
- Rendre publics tous les cas de non-conformité, ainsi que toutes les activités relatives à la conformité et à l'application de la loi;
- Être mis en œuvre en complément des mesures prises pour examiner de façon proactive le matériel de marketing de l'industrie avant qu'il ne soit distribué aux professionnels de la santé.

Le Groupe consultatif d'experts a également indiqué qu'en dépit des limites de certains modèles de transparence internationaux, ces instruments sont de précieux outils pour rendre les données sur le marketing et la publicité accessibles au public et leur donner plus de visibilité, et pour permettre à la société civique et aux organismes gouvernementaux de mieux évaluer les pratiques de marketing.

Résumé des avis

Les membres étaient conscients de l'ampleur et de la complexité de la crise des opioïdes qui sévit actuellement. Ils ont souligné que même si les activités de marketing des opioïdes menées par l'industrie ont contribué à la crise, le marketing des opioïdes ne représente actuellement qu'une partie d'une crise plus large provoquée par différents facteurs.

---

[16] Voir *Medicines Australia Code of Conduct* et la décision de l'Australian Competition & Consumer Commission du 24 avril 2015.

[17] Comprend des règles légales contenues dans le *Medicines Act* des Pays-Bas et un registre national volontaire en matière de transparence : https://www.transparantieregister.nl/home

[18] Voir le Code sur la publication (Disclosure Code) de la Fédération européenne des associations et industries pharmaceutiques (EFPIA); et la directive en matière de politique de l'Union européenne https://www.ema.europa.eu/en/documents/regulatory-procedural-guideline/directive-2001/83/ec-european-parliament-council-6-november-2001-community-code-relating-medicinal-products-human-use_en.pdf

En même temps, les membres ont indiqué que la crise des opioïdes a mis en lumière le rôle que peuvent jouer les activités de marketing persuasives, diverses et soutenues menées par l'industrie sur l'influence des pratiques de prescription. En particulier, les membres sont d'avis que les pratiques de marketing et de promotion ainsi que leurs répercussions constituent un problème ne se limitant pas aux opioïdes. Des questions autres que celles liées aux opioïdes devraient être examinées pour faire en sorte que les activités de marketing et de publicité pratiquées par l'industrie soient appropriées et que la surveillance de la promotion des médicaments soit suffisante.

S'appuyant sur les données probantes disponibles, le Groupe consultatif d'experts a fait valoir la pertinence de restreindre les activités de marketing et de publicité de l'industrie auprès des professionnels de la santé en ce qui a trait aux opioïdes.

En particulier, le Groupe consultatif d'experts conclut que :

- Il y a un besoin pour des renseignements sur les médicaments qui sont concis, impartiaux et facilement accessible aux professionnels de la santé, de plus ces renseignements constituent un soutien essentiel à une pratique de prescription appropriée;
- Des restrictions supplémentaires en matière de marketing et de publicité des opioïdes imposées à l'industrie ne généreraient pas un manque d'information;
- Le marketing et la publicité devraient présenter tous les bienfaits et méfaits des produits, et ce, de façon exacte et équilibrée;
- L'invitation provisoire faite aux fabricants et distributeurs à cesser volontairement leurs activités de marketing et de publicité des opioïdes devrait devenir permanente;
- Les paiements et autres transferts de valeur aux professionnels de la santé devraient être rendus publics, et certains de ces transferts non liés à la recherche (p. ex. dons de nourriture et de boisson, frais de déplacement subventionnés pour les participants à des conférences, honoraires des conférenciers et consultants en marketing) devraient cesser complètement;
- Des lignes directrices sur l'éducation, l'orientation et la pratique clinique devraient être élaborées de façon à réduire au minimum l'influence exercée par l'industrie;
- Des modèles internationaux existent et ils sont instructifs, particulièrement en ce qui a trait aux transferts de valeur aux organisations et professionnels de la santé;
- Rendre publics l'information de marketing représente une mesure importante permettant une étude plus approfondie et une meilleure compréhension des pratiques de marketing;
- Bien que la transparence constitue une étape essentielle, elle ne suffira pas à elle seule à atténuer l'éventuel impact négatif du marketing.

Expert Advisory Group on the Marketing of Opioids
Summary of Meeting #4
February 12, 2019

Members in attendance:
Dr. Irfan Dhalla
Dr. Andreas Laupacis
Dr. Barbara Mintzes
Dr. Trudo Lemmens

Health Canada (HC) Officials:
Amanda Moir, Director (acting Chair)
Lissa Murseli, Manager
Jessica Faubert, Senior Policy Analyst
David Lee, Chief Regulatory Officer (presenter)
Kathy Soltys, Director (presenter)
Lisa Lange, Director (presenter)
Alain Musende, Manager (presenter)

Regrets:
Ed Morgan, Director General (Chair)

Summary of Discussion:

1. **Opening remarks and agenda**
    - Health Canada (HC) presented the agenda for comments.

2. **Approval of meeting minutes**
    - Members approved the minutes of the September 27, 2018 meeting.

3. **Federal Updates**
    - HC reminded members that information on the Expert Advisory Group was made publicly available in December.
    - HC updated members that the June 2018 Notice of Intent What We Heard report, and a summary of the consultation session at the September 2018 Opioid Symposium have been posted online.
    - HC provided background on the current legislative authorities under the *Food and Drugs Act* as it relates to the advertising of health products.
    - HC provided an update on the implementation of Canadian Specific Opioid Targeted Risk Management Plans (CSO-tRMPs), as well as opioid specific labeling changes, mandatory stickers and patient handouts.
        - HC communicated preliminary results from the first wave of Canadian Specific Opioid Targeted Risk Management Plans.
        - HC indicated the intent to publish the products that have CSO-tRMPs and a summary of HC's review.
        - Members noted that publishing the contents of these plans might increase the effectiveness and transparency of RMP requirements.
    - HC provided an update on HC's Proactive Monitoring of the Canadian health product advertising environment announced by the Minister in June 2018. HC highlighted these activities take a risk-based approach with prioritization on opioid-related advertising.

- Members noted the importance that Continued Medical Education and professional development activities be independent from industry. HC indicated these events will be closely monitored through HC's Proactive Monitoring efforts.

4. **Considerations in potential further restrictions to opioid advertising**
   - HC outlined a proposed approach to further restrict the marketing and advertising of opioids.
     - Members indicated the proposed approach would encourage provision of accurate and balanced information for health care professionals.
     - Members noted the importance for advertising to present a balanced perspective of benefit and harm.
     - Members mentioned that promotional activities, play a role in influencing physicians and other health care professionals, and that promotional activities have shifted from direct advertising (e.g. print ads and pamphlets) to a broader array of indirect approaches (e.g. social media, honorariums, etc.) over time.

5. **Planned Stakeholder Engagement**
   - HC updated members on proposed stakeholder engagement plans to inform stakeholders of HC action, and seek targeted feedback related to federal action, views on increased transparency of opioid marketing, and information on marketing and opioid-related harms.
     - Members noted the importance of consulting a range of stakeholder groups to seek balanced views, and the need for conflicts of interest and affiliations to be disclosed.

6. **Recent Research**
   - HC highlighted recently published research related to opioid marketing, prescribing and opioid-related harms in the United States.
     - Members indicated a lack of comparable marketing data in Canada exists.
     - Members noted U.S. findings could reasonably be considered applicable in the Canadian context.

7. **Roundtable and closing remarks**
   - Members asked about Health Canada's broad approach to addressing the opioid crisis, beyond the scope of this Expert Advisory Group. HC indicated the Department is taking comprehensive actions and committed to inviting HC colleagues to provide more information on the comprehensive response to the opioid crisis at the next meeting.

Groupe consultatif d'experts sur le marketing des opioïdes
Compte rendu de la quatrième réunion
12 février 2019

Membres présents
Dr Irfan Dhalla
Dr Andreas Laupacis
Barbara Mintzes, Ph. D.
Trudo Lemmens, Ph. D.

Représentants de Santé Canada
Amanda Moir, directrice (présidente intérimaire)
Lissa Murseli, gestionnaire
Jessica Faubert, analyste principale des politiques
David K. Lee, chef de la réglementation (présentateur)
Kathy Soltys, directrice (présentatrice)
Lisa Lange, directrice (présentatrice)
Alain Musende, gestionnaire (présentateur)

Absents
Ed Morgan, directeur général (président)

Résumé des discussions

1. **Mot de bienvenue et ordre du jour**
    - Santé Canada (SC) présente l'ordre du jour pour commentaires.

2. **Approbation du procès-verbal**
    - Les membres approuvent le procès-verbal de la réunion du 27 septembre 2018.

3. **Nouvelles du gouvernement fédéral**
    - SC rappelle aux membres que l'information sur le Groupe consultatif d'experts a été rendue publique en décembre.
    - SC informe les membres que le rapport *Ce que nous avons entendu* lié à l'avis d'intention de juin 2018 et que la séance de consultation tenue lors du Symposium sur les opioïdes en septembre 2018 ont été publiés en ligne.
    - SC donne un aperçu général des actuels pouvoirs législatifs prévus par la *Loi sur les aliments et drogues* concernant la publicité des produits de santé.
    - SC fait le point sur la mise en œuvre des plans canadiens ciblant la gestion des risques spécifiques aux opioïdes (PCcGRSO), de même que des modifications d'étiquetage propres aux opioïdes, des autocollants obligatoires et des feuillets de renseignements à l'intention des patients.
        - SC communique les résultats préliminaires de la première vague de plans canadiens ciblant la gestion des risques spécifiques aux opioïdes.
        - SC mentionne l'intention de publier les produits qui sont assortis de PCcGRSO et un résumé de l'examen de SC.
        - Les membres signalent que la publication du contenu de ces plans pourrait augmenter l'efficacité et la transparence des exigences liées aux plans de gestion des risques.

- SC fait le point sur la surveillance proactive du cadre de la publicité sur les produits de santé par SC, annoncée par la ministre en juin 2018. SC souligne que ces activités comportent une approche fondée sur les risques qui met en priorité la publicité sur les opioïdes.
    - Les membres font remarquer qu'il importe que les activités continues de formation médicale et de perfectionnement professionnel soient indépendantes par rapport à l'industrie. SC mentionne que ces activités seront surveillées de près dans le cadre des démarches de surveillance proactive de SC.

4. **Facteurs à prendre en considération dans les éventuelles restrictions supplémentaires visant la publicité sur les opioïdes**
    - SC décrit brièvement une approche proposée pour restreindre davantage le marketing et la publicité sur les opioïdes.
        - Les membres mentionnent que l'approche proposée favoriserait la transmission d'information exacte et nuancée aux professionnels de la santé.
        - Les membres font remarquer qu'il importe que la publicité présente un point de vue objectif des bienfaits et des effets nocifs.
        - Les membres mentionnent que les activités promotionnelles influent sur les médecins et les autres professionnels de la santé, et que ces activités sont passées de la publicité directe (p. ex. publicités imprimées et dépliants) à un éventail plus vaste d'approches indirectes (p. ex. médias sociaux, rétributions) au fil du temps.

5. **Mobilisation des intervenants prévue**
    - SC met les membres au courant des plans de mobilisation des intervenants proposés pour informer les intervenants des mesures prises par SC et veiller à obtenir des commentaires ciblés concernant les mesures prises par le gouvernement fédéral, les points de vue au sujet de la transparence accrue du marketing des opioïdes, de même que de l'information sur le marketing et les effets nocifs des opioïdes.
        - Les membres signalent l'importance de consulter de nombreux groupes d'intervenants pour obtenir des points de vue nuancés, en plus du besoin de divulguer les conflits d'intérêts et les affiliations.

6. **Étude récente**
    - SC souligne une étude récemment publiée aux États-Unis au sujet du marketing, de la prescription et des effets nocifs des opioïdes.
        - Les membres mentionnent le manque de données de marketing comparables au Canada.
        - Les membres font remarquer que les constatations américaines pourraient raisonnablement s'appliquer dans le contexte canadien.

7. **Table ronde et mot de la fin**
    - Les membres s'enquièrent de l'approche globale de SC pour régler la crise des opioïdes, au-delà des limites de ce Groupe consultatif d'experts. SC mentionne que le Ministère prend des mesures complètes et tient à inviter les collègues de SC à fournir plus d'information sur la réponse globale à la crise des opioïdes à la prochaine réunion.

Expert Advisory Group on the Marketing of Opioids
Summary of Meeting #5
April 4, 2019

Members in attendance:
Dr. Irfan Dhalla
Dr. Andreas Laupacis
Dr. Barbara Mintzes
Dr. Trudo Lemmens

Health Canada (HC) Officials:
Ed Morgan, Director General (Chair)
Amanda Moir, Director
Roxanne Lewis, Manager
Heather Parsons, Senior Policy Analyst
Charlie Barnett, Policy Analyst
Lisa Lange, Director (presenter)
Alain Musende, Manager (presenter)
Jennifer Novak, Executive Director (presenter)

Summary of Discussion:

1. **Opening remarks and agenda**
    - Health Canada (HC) presented the agenda for comments.

2. **Approval of meeting minutes**
    - Members approved the summary of the February 12, 2019 meeting in advance.

3. **Federal Updates**
    - Health Canada updated members on the March 11, 2019 announcement proposing further restrictions to the marketing and advertising of opioids through terms and conditions. HC explained that manufacturer had a 15 day period to comment that ended April 1, 2019.
        - Health Canada outlined that it would be considering the comments received and, following final decisions, the department will notify individual Market Authorization Holders of any additional terms and conditions by end of April 2019 with the expectation that all new advertising materials developed as of June 2019 would be required to comply with the new terms and conditions.
        - Members noted the importance of determining how advertising is being defined and the role of pre-clearance agencies.
    - Health Canada updated members on the launch of the Stop Illegal Marketing of Drugs and Devices website aimed at health care professionals to provide information on health product advertising.

4. **Federal Actions on Opioids**
    - Health Canada provided members with an overview of the Canadian opioid crisis and federal actions taken to date, including the $150 million emergency treatment fund announced in Budget 2018.

- Health Canada updated members on the Budget 2019 funding announcement aimed at expanding access to safe supply of prescription opioids.
- Health Canada informed members on emerging priorities including addressing stigma, persistent gaps in harm reduction and treatment, and chronic pain and opioid crisis linkages.
    - Members appreciated the opportunity to learn about comprehensive and ongoing federal actions, and noted the importance of increasing the public's awareness of HC's response to the crisis, and suggested having a well-known figure as champion. Members also noted that a champion could encourage provinces to improve access to Suboxone.
    - Members inquired about the existence of Canadian data differentiating prescription and illicit opioid use, similar to the data produced by the Centers for Disease Control and Prevention in the United States. Health Canada noted that currently such data does not exist for Canada.

5. **Summary of EAG members' input**
    - Health Canada proposed that members provide written comments on the summary of the committee's work and advice.

6. **Recent Research**
    - Members shared two recent studies, one showing an increase in mortality among osteoarthritis patients using Frenado and the second indicating that industry funded continuing medical education (CME) differs in terms of pain management information compared to independent CME.

7. **Roundtable and closing remarks**
    - Health Canada completed administrative items.

Groupe consultatif d'experts sur le marketing des opioïdes
Compte rendu de la cinquième réunion
4 avril 2019

Membres présents
Dr Irfan Dhalla
Dr Andreas Laupacis
Mme Barbara Mintzes
M. Trudo Lemmens

Représentants de Santé Canada (SC)
Ed Morgan, directeur général (président)
Amanda Moir, directrice
Roxanne Lewis, gestionnaire
Heather Parsons, analyste principale des politiques
Charlie Barnett, analyste des politiques
Lisa Lange, directrice (présentatrice)
Alain Musende, gestionnaire (présentateur)
Jennifer Novak, directrice exécutive (présentatrice)

Résumé des discussions

1. **Mot de bienvenue et ordre du jour**
   - Santé Canada (SC) présente l'ordre du jour pour recueillir des commentaires.

2. **Approbation du procès-verbal**
   - Les membres ont approuvé en avance le résumé de la réunion du 12 février 2019.

3. **Mises à jour du gouvernement fédéral**
   - Santé Canada fait le point sur l'annonce du 11 mars 2019 proposant d'autres restrictions au marketing et à la publicité des opioïdes en imposant certaines conditions. SC explique que les fabricants disposaient d'une période de 15 jours pour formuler des commentaires. Cette période a pris fin le 1er avril 2019.
     - Santé Canada indique qu'on tiendrait compte des commentaires reçus et qu'à la suite des décisions finales, le Ministère avisera les titulaires d'une autorisation de mise en marché de toute condition supplémentaire d'ici la fin d'avril 2019, en s'attendant à ce que tous les nouveaux documents de publicité élaborés à partir de juin 2019 soient conformes aux nouvelles conditions.
     - Les membres soulignent l'importance de déterminer comment la publicité est définie et de préciser le rôle des agences de contrôle préalable.
   - Santé Canada fait le point sur le lancement du site Web Mettons fin au marketing illégal des médicaments et des instruments médicaux, qui vise à informer les professionnels de la santé sur la publicité des produits de santé.

4. **Mesures fédérales relatives aux opioïdes**
   - Santé Canada présente un aperçu de la crise des opioïdes au Canada et des mesures fédérales prises à ce jour, y compris le Fonds d'urgence pour le traitement de 150 millions de dollars annoncé dans le budget de 2018.

- Santé Canada fait le point sur l’annonce de financement du budget de 2019 visant à élargir l’accès à un approvisionnement sécuritaire en opioïdes d’ordonnance.
- Santé Canada informe les membres des nouvelles priorités, y compris la lutte contre la stigmatisation, les lacunes persistantes en matière de réduction des effets négatifs et de traitement, ainsi que les liens entre la douleur chronique et la crise des opioïdes.
    - Les membres apprécient l’occasion d’en apprendre davantage sur les mesures fédérales en cours. Ils soulignent l’importance de sensibiliser davantage le public à la réponse de SC à la crise, et suggèrent de trouver une personnalité bien connue comme champion. Les membres font également remarquer qu’un champion pourrait encourager les provinces à améliorer l’accès au Suboxone.
    - Les membres posent des questions sur l’existence de données canadiennes différenciant l’utilisation d’opioïdes d’ordonnance et illicites, semblables aux données produites par le Centers for Disease Control and Prevention aux États-Unis. Santé Canada fait remarquer qu’à l’heure actuelle, de telles données n’existent pas pour le Canada.

5. **Résumé des commentaires des membres du GCE**
    - Santé Canada propose que les membres fournissent des commentaires écrits sur le résumé des travaux et des conseils du comité.

6. **Recherche récente**
    - Les membres prennent connaissance de deux études récentes, l’une montrant une augmentation de la mortalité chez les patients atteints d’arthrose qui utilisent du Frenado et l’autre indiquant que l’éducation médicale continue (EMC) financée par l’industrie diffère de l’EMC indépendante en ce qui concerne l’information sur la gestion de la douleur.

7. **Tour de table et mot de la fin**
    - Santé Canada a complété des tâches administratives.

Expert Advisory Group on the Marketing of Opioids
Summary of Meeting #6
May 23, 2019

Members in attendance:
Dr. Irfan Dhalla
Dr. Andreas Laupacis
Dr. Barbara Mintzes
Dr. Trudo Lemmens

Health Canada (HC) Officials:
Ed Morgan, Director General (Chair)
Amanda Moir, Director
Roxanne Lewis, Manager
Heather Parsons, Senior Policy Analyst
Charlie Barnett, Policy Analyst
Ahmadreza Mohammadi, Policy Analyst
Alain Musende, Manager (presenter)

Summary of Discussion:

1. **Opening remarks and agenda**
    - Health Canada (HC) presented the agenda for comments.

2. **Approval of meeting minutes**
    - Members approved the summary of the April 4, 2019 meeting.

3. **Federal Updates**
    - Health Canada updated members on the changes to the terms and conditions to further restrict the marketing and advertising of opioids.
        - Comments received from the industry were minimal.
        - Health Canada sent letters to the industry at the beginning of May as a reminder that the new restrictions will come in force in June. Eighty percent of the recipients have acknowledged the receipt of this letter and have no objections or comments.
    - Health Canada updated members on the launch of the Stop Illegal Marketing of Drugs and Devices website aimed at health care professionals to provide information on health product advertising.
        - Members inquired if the complaints received would be made public and what happens following a complaint. HC confirmed that they will be posted quarterly in “the table of advertising incident” on its website. HC also explained that actions following a complaint can include: wilful compliance, stop a sale or seizure of the material, and suspending a licence.
    - Health Canada informed members on recent roundtables with Professional Regulatory Bodies and Health Care Professional Associations on marketing and advertising of opioids. A summary of the discussions will be made available for order on Health Canada’s website.

4. **Summary of EAG members' input**
   - Members discussed outstanding comments and agreed to approve the final advice by email.

5. **Recent Research**
   - Members shared two recent studies, one showing an increase in mortality among osteoarthritis patients using Frenado and the second indicating that industry funded continuing medical education (CME) differs in terms of pain management information compared to independent CME.

6. **Roundtable and closing remarks**
   - Health Canada thanked the members for their contribution to the working group and noted that the work of the group is now complete.

Groupe consultatif d'experts sur le marketing des opioïdes
Compte rendu de la sixième réunion
23 mai 2019

Membres présents :
Dr Irfan Dhalla
Dr Andreas Laupacis
Mme Barbara Mintzes, Ph. D.
M. Trudo Lemmens, Ph. D.

Représentants de Santé Canada (SC) :
Ed Morgan, directeur général (président)
Amanda Moir, directrice
Roxanne Lewis, gestionnaire
Heather Parsons, analyste principale des politiques
Charlie Barnett, analyste des politiques
Ahmadreza Mohammad, analyste des politiques
Alain Musende, gestionnaire (présentateur)

Résumé des discussions :

1. **Mot de bienvenue et ordre du jour**
    - Santé Canada (SC) présente l'ordre du jour pour recueillir des commentaires.

2. **Approbation du procès-verbal**
    - Les membres approuvent le résumé de la réunion du 4 avril 2019.

3. **Mises à jour du gouvernement fédéral**
    - Santé Canada fait le point sur les changements apportés aux conditions visant à restreindre davantage le marketing et la publicité des opioïdes.
        - Les commentaires reçus de la part de l'industrie étaient minimes.
        - Santé Canada a envoyé des lettres à l'industrie au début de mai pour leur rappeler que les nouvelles restrictions entreront en vigueur en juin. Quatre-vingts pour cent des destinataires ont accusé réception de cette lettre et n'avaient aucune objection ou commentaire.
    - Santé Canada fait le point sur le lancement du site Web Mettons fin au marketing illégal des médicaments et des instruments médicaux, qui vise à informer les professionnels de la santé sur la publicité des produits de santé.
        - Les membres demandent si les plaintes reçues seront rendues publiques et ce qui se produira suite à une plainte. SC confirme qu'elles seront affichées une fois par trimestre dans la section « tableau des incidents en matière de marketing » sur son site Web. SC explique également que les mesures prises à la suite d'une plainte peuvent inclure les suivantes : conformité volontaire, cessation d'une vente ou saisie du matériel, et suspension d'une licence.
    - Santé Canada fait le point sur les récentes tables rondes sur le marketing et la publicité des opioïdes avec des organismes de réglementation professionnels et des associations de professionnels de la santé. Un résumé des discussions sera disponible sur commande sur le site Web de Santé Canada.

4. **Résumé des commentaires des membres du GCE**
    - Les membres discutent des commentaires formulés et conviennent d'approuver l'avis final par courriel.

5. **Recherche récente**
    - Les membres ont partagé deux études récentes, l'une montrant une augmentation de la mortalité chez les patients atteints d'arthrose qui utilisent du Frenado et l'autre indiquant que la formation médicale continue (FMC) financée par l'industrie diffère de l'FMC indépendante en ce qui concerne l'information sur la gestion de la douleur.

6. **Tour de table et mot de la fin**
    - Santé Canada remercie les membres de leur contribution au groupe de travail et souligne que le travail du groupe est maintenant terminé.

Aralez Pharmaceuticals
Breathing New Life Into Pharmaceuticals™

Global Headquarters
7100 West Credit Avenue
Mississauga, Ontario L5N 0E4

U.S. Headquarters
400 Alexander Park Drive
Princeton, NJ 08540

Ireland Headquarters
47-49 St. Stephen's Green
Dublin 2, Ireland

June 19, 2018

The Honourable Ginette Petitpas Taylor
Minister of Health
Ottawa, ON K1A 0K9

RE: OPIOID CRISIS

Dear Ms. Petitpas Taylor,

Thank you for your letter regarding the opioid crisis dated June 19, 2018. We too are very concerned about this issue in Canada, and are very encouraged to see the government taking decisive action.

Please be advised that Aralez Pharmaceuticals Canada Inc. does not promote opioid products, nor do we have plans on doing so in the future.

Yours sincerely,

[redacted]

James Hall,
General Manager
ARALEZ PHARMACEUTICALS CANADA INC.

Dear Company X,

I am writing to you today to seek your commitment in support of Canada's collective response to the opioid crisis by immediately suspending any and all marketing and advertising of opioids to healthcare professionals.

Our Government is deeply concerned about this urgent public health issue which is affecting Canadians across all regions and from all segments of society. Although there are many factors that have led to this situation, the high level of opioids historically prescribed in Canada has certainly contributed to what is now a national public health crisis.

In February 2018, Purdue Pharma in the United States voluntarily ceased promoting prescription opioid products as their contribution to limiting the potential harms of opioid medications. This commitment serves as a strong example of how your industry can support "re-centering the pendulum" on opioid prescribing practices. I would welcome similar actions from Canadian pharmaceutical companies.

My officials are exploring options at the federal level to achieve the kind of results we need and increase the overall transparency of pharmaceutical marketing in Canada. The Government intends to restrict the marketing and advertising of prescription opioids in Canada. Health Canada is launching a public consultation which will be open until July 18, 2018. Stakeholders are invited to share information and views on the benefits and risks of opioid marketing and advertising, and potential restrictions.

I call on industry to show leadership by immediately suspending marketing and advertising of opioids. I welcome your prompt acknowledgment and action on this request. As you know, the Government of Canada is committed to openness and transparency. As such, Health Canada will publish all responses received. I look forward to publicly recognizing those pharmaceutical companies that respond to this call to action.

I look forward to your response.

Yours sincerely,

The Honourable Ginette Petitpas Taylor
Minister of Health

Chère compagnie,

Je vous écris aujourd'hui pour solliciter votre engagement à l'appui de la réponse collective du Canada à la crise des opioïdes en suspendant immédiatement toute commercialisation et toute publicité d'opioïdes auprès des professionnels de la santé.

Notre gouvernement est profondément préoccupé par cet enjeu urgent de santé publique qui touche les Canadiens de toutes les régions et de tous les segments de la société. Bien que de nombreux facteurs aient mené à cette situation, le niveau historiquement élevé d'opioïdes prescrit au Canada a certainement contribué à ce qui est maintenant une crise nationale de santé publique.

En février 2018, *Purdue Pharma* aux États-Unis a volontairement cessé de promouvoir les produits opioïdes sur ordonnance comme contribution afin de limiter les méfaits potentiels des médicaments opioïdes. Cet engagement est un bon exemple de la façon dont votre industrie peut «remettre les pendules à l'heure» en matière de pratiques de prescription d'opioïdes. Je serais heureuse de voir des actions similaires de la part des compagnies pharmaceutiques canadiennes.

Mes fonctionnaires explorent des options au niveau fédéral pour obtenir le genre de résultats dont nous avons besoin et accroître la transparence globale en matière de commercialisation pharmaceutique au Canada. Le gouvernement a l'intention de restreindre la commercialisation et la publicité sur les opioïdes sur ordonnance au Canada. Santé Canada lance une consultation publique qui sera ouverte jusqu'au 18 juillet 2018. Les intervenants sont invités à nous transmettre des renseignements et leur point de vue sur les avantages et les risques associés au marketing et à la publicité sur les opioïdes et aux éventuelles restrictions à cet égard.

J'appelle l'industrie à faire preuve de leadership en suspendant immédiatement le marketing et la publicité sur les opioïdes. J'accueille favorablement votre confirmation et votre action rapide sur cette demande. Comme vous le savez, le gouvernement du Canada est déterminé à faire preuve d'ouverture et de transparence. À ce titre, Santé Canada publiera toutes les réponses reçues. Je suis impatiente de reconnaître publiquement les sociétés pharmaceutiques qui répondent à cet appel à l'action.

J'attends avec impatience votre réponse.

Je vous prie d’agréer l’expression de mes sentiments les meilleurs

L'honorable Ginette Petitpas Taylor
ministre de la Santé

Recipients of Minister of Health's Letter June 19, 2018	Destinataires de la lettre de la Ministre de la santé le 19 juin 2018
1. AA Pharma Inc.<br>2. AbbVie Corporation<br>3. Accord Healthcare Inc.<br>4. Adapt Pharma Canada Limited<br>5. Allergan Inc.<br>6. Apotex Inc.<br>7. Aralez Pharmaceuticals Canada Inc.<br>8. Aspen Pharmacare Canada Inc.<br>9. Astrazeneca Canada Inc.<br>10. Auro Pharma Inc.<br>11. Baxter Corporation<br>12. Biogen Canada Inc.<br>13. Biomed Pharma<br>14. Biosyent Pharma Inc.<br>15. Boehringer Ingelheim (Canada) Limited<br>16. Bristol-Myers Squibb Canada<br>17. Buzzz Pharmaceuticals Ltd. (Dublin)<br>18. Canada's Medical Device Technology Companies (Medec)<br>19. Canadian Generic Pharmaceuticals Association<br>20. Church & Dwight Canada Corporation<br>21. Cipher Pharmaceuticals Inc.<br>22. Collegium Pharmaceutical, Inc.<br>23. Dentsply Canada Limited<br>24. DRA Pharmedev Canada Inc.<br>25. Eli Lilly Canada Inc.<br>26. EMD Serono, A Division Of EMD Inc., Canada<br>27. Endo Ventures Inc.<br>28. ERFA Canada 2012 Inc.<br>29. Eisai Limited<br>30. Ethypharm<br>31. Euro-Pharm International Canada Inc.<br>32. Fresenius-Kabi Canada Limited<br>33. Generic Medical Partners Inc.<br>34. GlaxoSmithKline Inc.<br>35. Groupement Provincial de l'industrie du médicament (GPIM)<br>36. GW Pharmaceuticals<br>37. Hansamed Limited<br>38. HLS Therapeutics Inc.<br>39. Hoffmann-La Roche Limited<br>40. INDIVIOR Canada Ltd<br>41. Innovative Medicines Canada<br>42. Intrinsik Corp.	1. AA Pharma Inc.<br>2. Accord Healthcare Inc.<br>3. Adapt Pharma Canada Limited<br>4. Allergan Inc.<br>5. Apotex Inc.<br>6. Aralez Pharmaceuticals Canada Inc.<br>7. Aspen Pharmacare Canada Inc.<br>8. Association canadienne du médicament générique<br>9. AstraZeneca Canada Inc.<br>10. Auro Pharma Inc.<br>11. Biogen Canada Inc.<br>12. Biomed Pharma<br>13. BioSyent Pharma Inc.<br>14. Boehringer Ingelheim (Canada) Limitée<br>15. Bristol-Myers Squibb Canada<br>16. Buzzz Pharmaceuticals Ltd. (Dublin)<br>17. Church & Dwight Canada Corporation<br>18. Cipher Pharmaceuticals Inc.<br>19. Collegium Pharmaceutical, Inc.<br>20. Corporation AbbVie<br>21. Dentsply Canada Limited<br>22. DRA Pharmedev Canada Inc.<br>23. Eisai Co. Limited<br>24. Eli Lilly Canada Inc.<br>25. Emd Serono, une division d'EMD Canada Inc.<br>26. Endo Ventures Inc.<br>27. ERFA Canada 2012 Inc.<br>28. Ethypharm<br>29. EURO-PHARM International Canada inc.<br>30. Fresenius-Kabi Canada Limitée<br>31. Generic Medical Partners Inc.<br>32. GlaxoSmithKline Inc.<br>33. Groupement Provincial de l'industrie du médicament (GPIM)<br>34. GW Pharmaceuticals<br>35. HANSAmed Limited<br>36. HLS Therapeutics Inc.<br>37. Hoffmann-La Roche Limitée<br>38. INDIVIOR Canada Ltd.<br>39. Intrinsik Corp.<br>40. Ionis Pharmaceuticals, Inc.<br>41. JAMP Pharma Corporation<br>42. Janssen Inc.<br>43. Knight Therapeutics Inc.

43. Ionis Pharmaceuticals, Inc.<br>44. JAMP Pharma Corporation<br>45. Janssen Inc.<br>46. Knight Therapeutics Inc.<br>47. Laboratoire Atlas<br>48. Laboratoire Riva Inc.<br>49. Lundbeck Canada Inc.<br>50. Lupin Pharma Canada<br>51. Mallinckrodt Canada ULC<br>52. Mantra Pharma Inc.<br>53. Mapi Group<br>54. Marcan Pharmaceuticals Inc.<br>55. Merck Canada Inc.<br>56. Mint Pharmaceuticals Inc.<br>57. Mitsubishi Tanabe Pharmaceutical Development America, Inc.<br>58. Mylan Pharmaceuticals ULC (BGP Pharma ULC)<br>59. Novartis Pharmaceuticals Canada Inc.<br>60. Odan Laboratories Ltd.<br>61. Omega Laboratories Ltd.<br>62. Otsuka Canada Pharmaceutical Inc.<br>63. Paladin Labs Inc.<br>64. Peptigroupe Inc.<br>65. Phebra Pty Ltd<br>66. Pfizer Canada Inc.<br>67. Pharmapar Inc.<br>68. Pharmascience Inc.<br>69. Pharmazeutische Fabrik Montavit Ges M.B.H.<br>70. Pro Doc Ltée<br>71. Purdue Pharma<br>72. Ranbaxy Pharmaceuticals Canada Inc. a SUN PHARMA Company<br>73. Regulatory Solutions Inc.<br>74. Sandoz Canada Inc.<br>75. Sanis Health Inc.<br>76. Sanofi-Aventis Canada Inc.<br>77. Shire Pharma Canada ULC<br>78. Sivem Pharmaceuticals ULC<br>79. Sterimax Inc.<br>80. Sterinova Inc.<br>81. Sunovion Pharmaceuticals Canada Inc.<br>82. Taro Pharmaceuticals Inc.<br>83. Teligent<br>84. Teva Canada Limited.<br>85. Therapeutic Products, Inc.<br>86. UCB Canada Inc.	44. Laboratoire Atlas<br>45. Laboratoire Riva Inc.<br>46. Lundbeck Canada Inc.<br>47. Lupin Pharma Canada<br>48. Mallinckrodt Canada ULC<br>49. Mantra Pharma Inc.<br>50. Mapi Group<br>51. Marcan Pharmaceuticals Inc.<br>52. Médicaments novateurs Canada<br>53. Merck Canada Inc.<br>54. Mint Pharmaceuticals Inc.<br>55. Mitsubishi Tanabe Pharmaceutical Development America, Inc.<br>56. Mylan Pharmaceuticals ULC (BGP Pharma ULC)<br>57. Novartis Pharmaceuticals Canada Inc.<br>58. Odan Laboratories Ltd.<br>59. Omega Laboratories Ltd.<br>60. Otsuka Canada Pharmaceutical Inc.<br>61. Paladin Labs Inc.<br>62. Peptigroupe Inc.<br>63. Pfizer Canada Inc.<br>64. Pharmapar Inc.<br>65. Pharmascience Inc.<br>66. Pharmazeutische Fabrik Montavit Ges M.B.H.<br>67. Phebra Pty Ltd<br>68. Pro Doc Ltée<br>69. Purdue Pharma<br>70. Ranbaxy Pharmaceuticals Canada Inc., un compagnie de SUN PHARMA<br>71. Regulatory Solutions Inc.<br>72. Sandoz Canada Inc.<br>73. Sanis Health Inc. (membre du réseau Pharmaprix)<br>74. Sanofi-Aventis Canada Inc.<br>75. Shire Pharma Canada ULC<br>76. Sivem Pharmaceuticals ULC<br>77. Société Baxter<br>78. Les sociétés canadiennes de technologies médicales (MEDEC)<br>79. SteriMax Inc.<br>80. Sterinova Inc.<br>81. Sunovion Pharmaceuticals Canada Inc.<br>82. Taro Pharmaceuticals Inc.<br>83. Teligent<br>84. Teva Canada Limitée<br>85. Therapeutic Products Inc.

87. Valeant Canada LP<br>88. Xediton Pharmaceuticals Inc.	86. UCB Canada Inc.<br>87. Valeant Canada LP<br>88. Xediton Pharmaceuticals Inc.
**Recipients of Health Canada's Letter August 17, 2018**	**Destinataires de la lettre de Santé Canada le 17 août 2018**
Version A	Version A
1. Actavis Pharma Company<br>2. Pendopharm a Divisions of de Pharmascience Inc.<br>3. Pfizer Consumer Healthcare a Division of Pfizer Canada Inc.	1. Actavis Pharma Company<br>2. Pendopharm, une division de Pharmascience Inc.<br>3. Pfizer Consumer Healthcare, une division de Pfizer Canada Inc
Version B	Version B
1. Actavis Group PTC ehf.<br>2. Bard Pharmaceuticals (1990) Inc.<br>3. Hospira Healthcare Corporation<br>4. Confab Laboratories Inc.<br>5. Laboratoires Trianon Inc.<br>6. Patriot a Division of Janssen Inc.<br>7. Tanta Pharmaceuticals Inc<br>8. Viva Pharmaceutical Inc.	1. Actavis Group PTC ehf.<br>2. Bard Pharmaceuticals (1990) Inc.<br>3. Corporation Hospira Healthcare<br>4. Laboratoires Confab Inc.<br>5. Laboratoires Trianon Inc.<br>6. Patriot, une division de Janssen Inc<br>7. Tanta Pharmaceuticals Inc.<br>8. Viva Pharmaceuticals Inc.
Version C	Version C
1. ICU Medical Canada<br>2. Pharmetics (2011) Inc.<br>3. Vita Health Products Inc.	1. ICU Medical Canada<br>2. Pharmetics (2011) Inc.<br>3. Vita Health Products Inc.

Petitpas Taylor, Minister / Ministre Ginette (HC/SC)

From: Hugues Gélinas <[redacted]@biomed-pharma.ca>
Sent: 2018-06-20 6:01 PM
To: Petitpas Taylor, Minister / Ministre Ginette (HC/SC)
Subject: RE: Lettre de la ministre de la Santé à l'Industrie Pharmaceutique : commercialisation et publicité des opioïdes

Bonjour,

Nous ne distribuons pas d'opioïdes présentent dans notre portfolio de molécules et nous sommes d'accord pour ne pas en faire la promotion après des professionnels de la santé.

Par contre, nous avons un partenariat avec un producteur de Naloxone (pre-filed injection) et Naltrexone (Implant) pour lesquels nous avons entamé des discussions avec Santé Canada dans le but de faire approuver les produits. Nous croyons sincèrement que l'approbation de ces produits aideraient les autorités Canadienne à faire face à cette crise de santé public.

ECD Health Canada
DCHG Santé Canada
Rec'd
Reçu JUL 04 2018
#18-004185-706

Cordialement,

Hugues Gélinas
Président Biomed Pharma

De : Petitpas Taylor, Minister / Ministre Ginette (HC/SC) <hcminister.ministresc@canada.ca>
Envoyé : 19 juin 2018 11:04
À : Hugues Gélinas
Objet : Lettre de la ministre de la Santé à l'Industrie Pharmaceutique : commercialisation et publicité des opioïdes

Veuillez trouver ci-joint, une lettre de la ministre de la Santé.

Merci
Health Canada - Santé Canada

MINT PHARMACEUTICALS INC.
1093 MEYERSIDE DRIVE, UNIT #1
MISSISSAUGA, ONTARIO, CANADA L5T 1J6
TEL 905.795.9437
FAX 905.271.9696
TF 877.398.9696
mintpharmaceuticals.com

June 21th, 2018

Ginette Petitpas Taylor
P.C, M.P.
Minister of Health Canada
Ginette.PetitpasTaylor@parl.gc.ca

Dear Honourable Ginette Petitpas Taylor,

At Mint Pharmaceuticals ("Mint"), we take seriously the state of the Canadian Healthcare system. We also understand our key role in improving the health of Canadians across the country by providing access to high quality and affordable pharmaceutical products.

We stand in solidarity with this great and difficult initiative that our government is undertaking. Mint will effective immediately, suspend and eliminate all marketing and advertising of opioids in Canada.

Mint is committed to improving the health and well-being of all Canadians across the country and we hope that our contribution and participation in this initiative will help the current and tragic opioid crisis.

Sincerely,

[redacted]

Jaiveer Singh
CEO, Mint Pharmaceuticals

Ranbaxy Pharmaceuticals Canada Inc.
(A SUN PHARMA company)
126 East Drive, Brampton, ON L6T 1C1 Canada
Tel: +1 905-790-5170
Fax: + +1 905-790-5198

To,
The Honorable Ginette Petitpas Taylor, PC MP
Minister of Health,
Govt. of Canada
Ottawa, K1A 0K9

Ref: You letter dated June 19, 2018 regarding your request to cease promoting and marketing of Opoids

Dear Ms. Taylor,

We have received your letter dated June 19, 2018 addressed to Mr. Abhay Gandhi, CEO, of North America at Sun Pharmaceuticals, which requests industry to immediately suspend marketing and advertising of opioids in Canada.

We can confirm that we do not advertise opioids in Canada. Sun Pharmaceuticals does not market or promote any opioid products to prescribers and therefore has no influence over the prescribing of opioids in Canada.

We are committed to contribute to well-being of Canadian citizens.

Please do not hesitate to contact me if you need further information in this regards.

Thank you,

[redacted]

Rakesh Upadhyaya
Ph: [redacted]
[redacted]@sunpharma.com

VIA EMAIL: Ginette.PetitpasTaylor@parl.gc.ca

June 22nd, 2018

The Honorable Ginette Petitpas Taylor P.C., M.P.
Minister of Health
Ottawa, Canada
K1A 0K9

Subject: Minister of Health Letter to Pharmaceutical Industry: Marketing and Advertising of Opioids

Dear Minister Petitpas Taylor,

Thank you for reaching out to us with regards to our industry's role in supporting the collective response to the current opioid crisis. As a global leader in the development of pharmacotherapy for opioid use disorder (OUD), Indivior stands with the government in its resolve to address the opioid crisis, which is arguably the most significant public health issue in Canada.

Indivior has actively engaged with the Opioid Response Team in 2017 and with the team responsible for the consultation on Risk Management Plans for opioid-containing products in March 2018. Both were very productive engagements.

During our discussions, we highlighted the need for a distinction to be made between opioid-containing products prescribed for pain and others, such as Suboxone, prescribed for the treatment of OUD. In response to the government's plans to implement regulations that might restrict the marketing and advertising of opioid containing products, Indivior welcomes the opportunity to once again distinguish OUD therapies from opioids that are prescribed for pain and other uses. Understanding the unique characteristics of evidence-based OUD therapies and their appropriate use becomes critically important as we work to address our national public health crisis. In fact, when there is a clear public health benefit associated with the adoption and integration of medicines that address important public health concerns, such as vaccines, industry has been permitted to advertise and promote these medicines even beyond prescribing healthcare professionals.

Indivior is committed to ethical and responsible marketing and promotion. The focus of our organization has always been to help remove barriers for patients to access treatment. Indivior has placed a strong emphasis on offering education to reduce the stigma associated with OUD which can prevent patients from seeking treatment. Indivior has offered free educational programs to healthcare professionals (HCPs) since 2007 on the disease of OUD and Suboxone as one of the treatment options. The Suboxone Training Program, for example, has received extremely positive feedback and has been accessed by more than 4,000 HCPs.

Indivior Canada Ltd., 2600 Blvd Alfred Nobel, Suite 200, Ville Saint-Laurent, QC, H4S 0A9
Tel (514) 331-1114, Fax (514) 332-1118

In this context, Indivior welcomes the opportunity to participate in the Health Canada public consultation. We will submit our input by the July 18th deadline. Our submission will provide Indivior's view of ongoing issues and challenges related to combating drug addiction and the opioid crisis in Canada, including recommendations on policies aimed at preventing addiction, accelerating evidence-based treatment and enabling long-term recovery for patients. Indivior is committed to addressing the opioid crisis together with all stakeholders.

We would welcome an opportunity to meet with you or colleagues in your office to discuss our therapies and recommendations related to a regulatory approach. We will be in contact with you in the coming weeks to schedule this meeting.

If you have any questions in the meantime, please do not hesitate to contact me.

Sincerely,

Christine LaFave
Country Director
INDIVIOR Canada Ltd.
T:[redacted]
F: 514-332-1118
www.indivior.com

c.c. Ed Morgan, Director General, Policy, Planning & International Affairs Directorate (PPIAD), Health Canada, ed.morgan@canada.ca

Petitpas Taylor, Minister / Ministre Ginette (HC/SC)

From: Sylvain Desjeans <[redacted]@ca.aspenpharma.com>
Sent: 2018-06-26 6:27 PM
To: Petitpas Taylor, Minister / Ministre Ginette (HC/SC)
Subject: RE: Minister of Health Letter to Pharmaceutical Industry: Marketing and Advertising of Opioids

Dear Minister Petitpas Taylor

Although we do not have opioid products, we are behind Canada's initiatives and will support if put to task

Respectfully

Sylvain

ECD Health Canada
DCHG Santé Canada
Rec'd
Reçu JUL 04 2018
18-004180-244

Sylvain Desjeans
Country Manager - Canada
Aspen Pharmacare Canada Inc
111 Queen Street East, Suite 450, Toronto, Ontario, M5C 1S2
Direct Tel: [redacted]
Mobile: [redacted]
www.aspenpharma.com

aspen Healthcare. We Care.

This email is solely for the named addressee. Any unauthorized use or interception of this email, or the review, retransmission, dissemination or other use of, or taking of any action in reliance upon the contents of this email, by persons or entities other than the intended recipient, is prohibited. If you are not the named addressee please notify us immediately by way of a reply e-mail, and also delete this email and any attached files.

Disclaimer: You must scan this email and any attached files for viruses and/or any other defects. Aspen accepts no liability for any loss, damages or consequence whether direct, indirect, consequential or economic, however caused, and whether by negligence or otherwise, which may result directly or indirectly from this communication or of any attached files.

From: Petitpas Taylor, Minister / Ministre Ginette (HC/SC) [mailto:hcminister.ministresc@canada.ca]
Sent: June 19, 2018 11:20 AM
To: Sylvain Desjeans <[redacted]@ca.aspenpharma.com>
Subject: Minister of Health Letter to Pharmaceutical Industry: Marketing and Advertising of Opioids

Please find attached correspondence from the Minister of Health Canada.

Thank you / Merci
Health Canada - Santé Canada

Shire Pharma Canada ULC
Bay Adelaide Centre
22 Adelaide Street West, Suite 3800
Toronto, ON
M5H 4E3 CANADA
Tel. +1 647-798-2200
Fax +1 647-798-2490
shirecanada.com

Shire

June 25, 2018

The Honourable Ginette Petitpas Taylor, P.C., M.P.
Minister of Health
House of Commons
Ottawa, Ontario
K1A 0A6

Dear Minister:

On behalf of Shire Pharma Canada ULC, thank you for consulting directly with pharmaceutical manufacturers on issues of concern to Canadians. We appreciate the collaboration and look forward to further dialogue in other areas of national health policy.

Shire Canada supports your commitment to address the serious issues surrounding opioid use in Canada. In response to your request, we do not manufacture market nor sell opioids in Canada, and can say with confidence that we will not market nor advertise opioids to health care professionals. In addition, we currently do not have any opioids in our pipeline and as a result, have no prospect of marketing or advertising opioids in future.

Thank you for the opportunity to comment on this important issue.

Kind regards,

[redacted]

Eric Tse
General Manager
Shire Canada

teva

June 26, 2018

The Honourable Ginette Petitpas Taylor
Minister of Health
Government of Canada
House of Commons
Ottawa, Canada
K1A 0K9
Delivered by email

Dear Madame Minister,

It was a great pleasure to meet you in April as part of a delegation from the Canadian Generic Pharmaceutical Association (CGPA) to talk about opportunities to work with your government to improve the health of Canadians. Teva is one of Canada's largest pharmaceutical companies making innovative and generic medicines available to Canadian patients and we are proud to be part of this important industry in Canada.

I have recently received a letter from you concerning your Ministry's intent to limit the marketing and advertising of opioids to health care professionals. You specifically have asked for industry to show leadership by suspending any marketing or advertising of opioids.

I am pleased to inform you that Teva does not promote or advertise any opioid products to prescribers and we support the government's efforts to restrict the marketing of prescription opioids to physicians.

Our security and quality control processes are extremely vigilant and we support our supply chain partners in programs aimed at tackling diversion of opioids. For example, Teva's "Patch-for-Patch" pharmacy program ensures that patients can receive a refill only when a used patch is returned or can safely dispose old patches.

One of the often cited causes of the public health issues around opioid use is over-prescribing. It is important to point out that education on the appropriate prescribing of opioids is critical. We support efforts to educate physicians on appropriate prescribing and patient education about opioid use and the safe storage of all medicines. As members of the CGPA, we are aligned in encouraging Health Canada to focus on a strategy that involves physician and patient education.

We appreciate the efforts your government is taking to combat this public health crisis and find solutions to avoid further tragedy. We are committed to working with you to make sure that Canadians who live with pain have access to these important medicines while also ensuring that these products are not abused or misused.

Please let me know if there is any more we can do to help.

Sincerely,

[redacted]

Christine Poulin
Senior Vice President and General Manager
Teva Canada Limited

ECD Health Canada
DCHG Santé Canada
Rec'd Reçu JUL 05 2018
18-004207.174

AstraZeneca Canada· Suite 5000, 1004 Middlegate Road · Mississauga, Ontario· L4Y 1MA
T: 1-888-669-2987 · F: 1-888-697-9886

AstraZeneca

The Hon. Ginette Petitpas Taylor, P.C., M.P.
Minister of Health
Health Canada
Brooke Claxton Building, Tunney's Pasture
Postal Locator: 0906C
Ottawa, Ontario
K1A 0K9

June 27, 2018

Dear Minister Petitpas Taylor:

Thank you for your recent letter regarding the commitment by the Government of Canada to address the opioid crisis.

The growing number of Canadian deaths caused by opioids is a public health emergency, and AstraZeneca Canada stands willing to assist Health Canada in response to this national health crisis.

Please note that AstraZeneca Canada does not distribute, market or promote any opioids to health care professionals in Canada.

On behalf of AstraZeneca Canada, we appreciate your outreach on this urgent public health matter and hope that we may be of assistance in the future.

Sincerely,

[redacted]

Dr. Jamie Freedman
President
AstraZeneca Canada Inc.

paladin
une compagnie de endo international
an endo international company

The Honourable Ginette Petitpas Taylor, P.C., M.P., Minister of Health
Health Canada
Brooke Claxton Building, Tunney's Pasture
Postal Locator: 0906C
Ottawa, Ontario K1A 0K9

June 27, 2018

Dear Ms. Petitpas Taylor:

I am writing to inform you of the decision by Paladin Labs Inc. (Paladin) to cease the advertising and promotion of opioid products in Canada. Among other things, effective immediately, Paladin will no longer distribute promotional materials to Canadian healthcare professionals regarding opioid products. Our U.S. affiliate, Endo Pharmaceuticals Inc., previously took similar steps in the United States.

As a company with a longstanding commitment to improve patients' lives, we remain mindful of the legitimate access needs of the millions of patients who suffer from acute or chronic pain who rely on opioid medications. At the same time, we share Health Canada's concern about the public health challenges that have arisen as the result of opioid abuse. As your recent letter to Paladin recognizes, many factors contributed to the current situation, and a comprehensive solution will require collaboration among multiple stakeholders within and beyond the healthcare system. We hope our actions today demonstrate our commitment to help achieve that goal.

We welcome further dialogue with Health Canada about the opioid abuse crisis in Canada and additional ways we might help. In the interim, please feel free to contact me with any questions you may have.

Sincerely,

[redacted]

Livio Di Francesco
Vice President and General Manager
Paladin Labs

Laboratoires Paladin Inc.
Paladin Labs Inc.
100 Boul. Alexis Nihon,
Bureau 600, Montréal
Québec H4M 2P2
1.888.376.7830
514.344.4675 fax
paladinlabs.com

Purdue Pharma
575 Granite Court
Pickering, Ontario
CANADA
L1W 3W8

Wednesday, June 27, 2018

Hon. Ginette Petitpas Taylor, P.C., M.P
Minister of Health
Health Canada
Brooke Claxton Building, Tunney's Pasture
Ottawa ON K1A 0K9

Dear Minister,

Thank you for your letter dated June 19th and we want to assure you that Purdue Pharma (Canada) is also deeply concerned about the opioids crisis. In response to your call for action to support Canada's collective response to the opioids crisis, effective June 20, 2018, **Purdue Pharma (Canada) suspended all promotional and advertising activities relating to our prescription opioids,** pending the outcome of your consultation and the implementation of new regulations.

While we have taken this action, we remain steadfast in our belief that Canadian prescribers require the most recent information, including on the most current guidelines, to ensure their patients are treated appropriately. Going forward, requests for information about our opioid products from healthcare professionals will be addressed reactively through direct communication with the experienced healthcare professionals in our Medical Affairs department.

Pain Management and Putting Patients First
Purdue Pharma (Canada) is a leader in pain management in Canada. Our company was physician-founded, with a 60-year history of supporting physicians and pain patients with Health Canada approved innovative products, independently accredited education for healthcare professionals, and ethical interactions by our employees across a range of health system stakeholders.

The opioids crisis is a complex and multifaceted public health issue that involves both prescription opioids and, increasingly, illegally produced and consumed opioids, as indicated in Health Canada's latest quarterly monitoring report. Nonetheless, all stakeholders, including the pharmaceutical industry, have a role to play in providing practical and sustainable solutions.

According to the Canadian Pain Society, 1 in 5 Canadians suffer from chronic pain and pain is the most common reason for seeking healthcare in Canada. Purdue Pharma (Canada) supports a research-driven approach to managing pain that may or may not include available prescription products, in concert with other effective pharmacological and non-pharmacological treatments.

As government policy with respect to the marketing of prescription opioid medications evolves, neither pain patients nor their physicians should be subject to stigmatization, either for seeking treatment for their conditions, or for treating patients according to their best professional and clinical judgment.

Pain medications, when appropriately prescribed in accordance with established guidelines, offer effective pain relief to patients in a number of areas, including surgery, oncology, chronic non-cancer pain and palliative care.

Continually Adhering to Government Regulatory Requirements
Purdue Pharma (Canada) has always marketed its products in line with the Health Canada approved product monograph and in compliance with all relevant rules, regulations and codes, including the *Food and Drugs Act* and the Pharmaceutical Advertising Advisory Board (PAAB) Code. Moreover, our company has always adhered to all Health Canada and international regulations, including pharmacovigilance reporting obligations. Additionally, as a member of Innovative Medicines Canada, we also adhere to its Code of Ethical Practices.

We endorse relevant clinical practice guidelines and, in fact, Purdue representatives have engaged with healthcare professionals (HCPs) to distribute and inform them of the 2017 Canadian Guideline for Opioids for Chronic Non-Cancer Pain, which we fully support. In November 2017, we had a positive meeting with the Health Canada opioids response leadership team in Ottawa to advise them of these efforts and provided the exact materials and tools we use with prescribers that are evidence-based and follow current clinical practice guidelines, such as the Patient Agreement and Opioid Risk Tool.

Evidence-Based Education is Essential
We support evidence-based, non-branded, and independently accredited Continuing Health Education (CHE) programs to improve clinical care and patient outcomes. These programs transfer knowledge of the latest advances in science and clinical practice between qualified HCPs and are subject to rigorous review by respected organizations, including the Royal College of Physicians and Surgeons of Canada, the College of Family Physicians of Canada and the Fédération des médecins omnipraticiens du Québec. We believe in the value of accredited CHE Programs that are currently offered to healthcare professionals through Purdue Pharma (Canada) which include:

- Optimizing Pain Relief and Function with Non-Pharmacological Therapies;
- Managing Chronic Pain: When and How to Discontinue Opioids;
- Urine Drug Testing (UDT) in Pain Management; and
- Approaches to Responsible Opioid Use: Maximizing Your Role in Improving Outcomes and Reducing Risk.

We have suspended these programs which we regard as education not promotion. However, we are concerned as to how HCPs will have access to similar information about chronic pain management, pharmacological and non-pharmacological pain therapies, and current opioid use guidelines that could be of benefit to clinicians and patients.

Peer-reviewed, scientific exchange between industry, researchers and clinicians is essential to drive medical progress and improve patient outcomes. Accordingly, we ask that you give thought to further approaches to facilitate ongoing educational support to healthcare professionals as new regulations are implemented.

Our Belief in Tamper-Resistant Opioid Medications

While doing our part to further education in pain management, Purdue Pharma (Canada) and independent associated companies have worked for more than 15 years on the development of abuse-deterrent formulation (ADF) / tamper-resistant (TR) opioid medicines to make the misuse, abuse and diversion of prescription opioids more difficult. Health Canada has recognized the TR features of both OxyNEO® and Targin®, the only two opioid products recognized as such in Canada.

Purdue's commitment to ADF and TR technologies is driven by the abundance of published evidence supporting the positive impact these technologies have on public health. Presently, Canadian prescribers, pharmacists and patients have limited access to these technologies. We continue to believe that governments – both federal and provincial – as well as private payers should consider accelerating their efforts to enact appropriate regulatory (where applicable) and reimbursement policies to increase the availability of ADF and TR opioid medications.

These technologies are one component of the broader solution to address the misuse, abuse and diversion of prescription opioid medicines and a host of national organizations including healthcare professionals, law enforcement and patient groups endorse this position. In addition, we continue to respond to law enforcement agencies to help them combat the illegal elements of this complex crisis.

Partnering for Results

Purdue Pharma (Canada) is making ongoing contributions to public health including:

- Support of CIHR's Strategy for Patient Oriented Research (SPOR) through a significant donation to the Michael G. DeGroote Institute for Pain Research ***Chronic Pain Network*** which was announced by your predecessor, Hon. Dr. Jane Philpott, on March 31, 2016;

- Funding programs for safe and effective collection and disposal of prescription medications through the Health Products Stewardship Association across Canada;

- Supporting Drug Free Kids Canada since its inception; and

- Educating law enforcement officers, first responders and healthcare providers with a tool to assist in identification of prescription opioid analgesics/stimulants marketed in Canada through the development of a product identification pamphlet, in concert with the Canadian Pharmacists Association, which is used daily by the aforementioned groups nation-wide.

To conclude, we firmly believe that our ethical engagement with HCPs and stakeholders, adherence to regulatory requirements, support for independently accredited CHE, investing in tamper-resistant opioids and partnering with national and regional organizations across industry, the academic and not-for-profit sectors were positive actions and part of the solution to the opioids crisis.

We look forward to contributing to your consultation process and should you have further questions, please do not hesitate to contact me directly at [redacted] or by email at [redacted]@purdue.ca. It would by my pleasure to meet with you as your schedule permits.

Sincerely,

[redacted]

David A. Pidduck
President and CEO

Petitpas Taylor, Minister / Ministre Ginette (HC/SC)

From: Antoine Poncy <[redacted]@ethypharm.com>
Sent: 2018-06-29 9:20 AM
To: Petitpas Taylor, Minister / Ministre Ginette (HC/SC)
Subject: RE: Minister of Health Letter to Pharmaceutical Industry: Marketing and Advertising of Opioids

Dear Honourable Ginette Petitpas Taylor,

I acknowledge receipt of your letter dated June 19.

Ethypharm Inc is committed to work with Health Canada to address the Opioid Crisis and as such has suspended all its promotional activities pending the result of the Public Consultation.

ECD Health Canada
DCHG Santé Canada
Rec'd Reçu JUL 04 2018
#18-04184-282

Yours Sincerely,
Antoine Poncy

Ethypharm

Antoine PONCY
Directeur des Opérations
Director of Operations

1000, rue de la Gauchetière Ouest, Suite 2400
Montreal, QC H3B 4W5
Phone : +[redacted]
Cell : +[redacted]
[redacted]@ethypharm.com

Visit our website at http://www.ethypharm.com/

[illegible]

From: Petitpas Taylor, Minister / Ministre Ginette (HC/SC) <hcminister.ministresc@canada.ca>
Sent: June 19, 2018 11:18 AM
To: Antoine Poncy <[redacted]@ethypharm.com>
Subject: Minister of Health Letter to Pharmaceutical Industry: Marketing and Advertising of Opioids

Please find attached correspondence from the Minister of Health Canada.

Thank you / Merci
Health Canada - Santé Canada

This message and any attachment contains confidential and/or privileged information and is intended only for the individual named. If you are not the named addressee, you should not disseminate, distribute, disclose or copy this email and/or attachment. Please notify the sender immediately by email if you have received this

email by mistake, please notify the sender immediately and delete this email and any attachment from your system. Email transmission cannot be guaranteed to be secure or error-free, as information could be intercepted, corrupted, lost, destroyed, arrive late or incomplete, or contain viruses. The sender, therefore, does not accept liability for any errors or omissions in the contents of this message which arise as a result of email transmission. If verification is required, please request a hard-copy version

SANDOZ A Novartis Division

Sandoz Canada Inc.
110 de Lauzon St.
Boucherville, QC
J4B 1E6
450 641 4903

June 28, 2018

The Honourable Ginette Petitpas Taylor
Minister of Health
Health Canada
Ottawa, Ontario K1A 0K9
hcminister.ministresc@canada.ca

Subject: **Request for immediate voluntary suspension of marketing and advertising activities associated with opioids**

Dear Mrs. Petitpas Taylor,

Thank you for your letter regarding the opioid crisis issue. Sandoz Canada completely shares your concern about the public health and safety matters and agrees that significant efforts are required at all levels of government and with all stakeholders to properly address this crisis. Sandoz Canada is committed to providing leadership on this important issue.

Sandoz Canada has never been involved in the advertising or marketing of opioids to prescribing physicians and we hereby commit to not promote or encourage, directly or indirectly, the prescription of any opioid product.

In addition, in the past years, Sandoz Canada has devoted considerable effort in mitigating the opioid crisis and we will continue to do so to ensure their safe and appropriate use. In 2016 following a request by Health Canada and after considerable effort at the Federal and Provincial levels, we increased access to naloxone, a life-saving drug that could reverse the effects of opioid overdoses, by launching the first non-prescription naloxone in Canada. We also participated in the distribution of thousands of naloxone injection kits as well as organized multiple educational sessions with pharmacists in order for them to better help patients.

In 2017, we actively participated in the Pharmacy Opioid Summit organized by the Canadian Pharmacists Association and identified further opportunities to tackle Canada's opioid crisis[1]. We also implemented our Patch-for-Patch Fentanyl Return Program across the country to limit its misuse.

Furthermore, we would like to highlight the following facts:

- Sandoz Canada's focus, as a generic manufacturer of opioids, is to **ensure safe and reliable supply** to hospitals and community pharmacies and
- Sandoz Canada sells the vast majority of its Part B - opioids in settings where **administration of the drug is under the supervision of a health care professional,** namely hospitals.

Sandoz Canada will make every effort to address misuse and abuse of opioids while also developing harm reduction strategies. You can count on Sandoz Canada as your ally in working towards solutions to ensure prevention and decrease prescription opioid-related harm to Canadians.

Sincerely,

[redacted]

Michel Robidoux
President and General Manager

[1] Some opportunities identified can be found by following this link:
https://www.pharmacists.ca/cpha-ca/assets/File/cpha-on-the-issues/Pharmacy%20Opioid%20Summit.pdf

www.ontariopainfoundation.ca

The Honourable Ginette Petitpas Taylor, M.P.
Minister of Health
Brooke Claxton Building, Tunney's Pasture
Ottawa, Ontario K1A 0K9

Minister_Ministre@hc-sc.gc.ca
Ginette.PetitpasTaylor@parl.gc.ca
Karina.Gould@parl.gc.ca

Dear Minister Petitpas Taylor:

I am writing to you concerning your letter of the 19th of June to drug companies regarding marketing and advertising activities associated with opioids.

I believe that people who live with chronic debilitating pain are being done a disservice by government policy.

Statistics show that while the medical use of prescription opioids is decreasing, the incidence of opioid use overdose deaths is increasing, yet the finger seems to be pointing at legitimate use as your letter to drug companies would imply.

While I share concerns about the growing number of opioid-related overdose deaths in Canada, I would ask you to remain cognisant of the two main causes being cited, one being the use of illicit fentanyl and related opioids, the other being the overuse of prescription opioids.

Evidence indicates that overuse of prescription opioids does not pose a threat to Canadians.

In the Federal report, entitled "*Pan-Canadian Trends in the Prescribing of Opioids and Benzodiazepines, 2012 to 2017*", found at https://www.cihi.ca/sites/default/files/document/opioid-prescribing-june2018-en-web.pdf, it is stated that **dispensing of prescription defined daily doses for opioids declined** by 10.1% between 2016 and 2017, and was consistent with the trend between 2012 and 2016.

In Ontario the **number of new opioid prescriptions decreased** by 2% in 2016 compared to those in 2013 (http://www.hqontario.ca/System-Performance/Specialized-Reports/Starting-on-Opioids-in-Ontario).

In 2013-2014 there were 8,705 opioid prescriptions filed for all uses and in 2015-2016 there were 1,939,924, representing a decrease in the number of people filling an opioid prescription over the time that the population grew from 13,677,687 to 13,959,890 over that period. That is a **decrease in the number of people filling an opioid prescription** from 14.1% to 13.9%. These data are obtained from https://www.fin.gov.on.ca/en/economy/demographics/projections/table1.html and from http://www.hqontario.ca/portals/0/Documents/system-performance/9-million-prescriptions-en.pdf.

www.ontariopainfoundation.ca

Thus, while the opioid crisis shows escalating numbers of opioid overdose deaths government data show a decrease in prescription opioid use.

In Canada, noted pain and addiction experts, Drs. Juurlink and Dhalla, in the *Journal of Medical Toxicology, 8: 393-399, 2012*, state that "… patients can suffer serious opioid-related harm, including death, despite never purposefully misusing and opioid", and "However, such instances are relatively uncommon, and **most serious opioid-related harm accrues to patients who abuse opioids or become addicted to them**".

These national statistics do not point to medical use of opioids as being a major contributor to the current opioid crisis.

I have been unable to find statistics of overdoses of prescription opioids in Canada. However, the *Centers for Disease Control and Prevention* in the USA indicate in their *Morbidity and Mortality Weekly Report* of 1 November, 2011, indicate that while **opioid treatment admission rates** per 10,000 and **opioid prescription sales rates** per 10,000 have each **increased significantly** by many factors over the period 1999 to 2009, the **rates of opioid overdose deaths** have **increased only marginally** over this same period.

The **link between prescription opioid use and opioid use disorder** appears to be untenable. For example, in a paper by Cooper, Willis, Fuller et al., *Prevalence and incidence trends for diagnosed prescription opioid use disorders in the United Kingdom, Pain Therapy 6: 73-84, 2017*, the authors conclude, "Our study demonstrates that despite the marked increase in overall opioid prescribing in the UK in the past decade, **there has not been an increase in the incidence of physician-diagnosed opioid use disorders**."

Prescription opioid use is for treatment of a medical disorder.

In the "*National report: Apparent opioid-related deaths in Canada (released March 2018)*", found at https://www.canada.ca/en/public-health/services/publications/healthy-living/national-report-apparent-opioid-related-deaths-released-march-2018.html, it is stated that from January to September 2017, there were at lease 2,923 apparent opioid-related deaths; **92% of opioid-related deaths were accidental.**

In a paper by Shield, Jones, Rehm and Fischer, of the Centre for Addiction and Mental Health at the University of Toronto (*Use and nonmedical use of prescription opioid analgesics in the general population of Canada and correlations with dispensing levels in 2009. Pain Res Management 18: 69-74, 2013*) it is stated that across Canada in 2009 the prevalence of prescription opioid analgesic use was 19%. However, the **prevalence of prescription opioid analgesic use that would fit the criteria of 'nonmedical' use was 5%.** The use of pain relievers to 'get high' was 0.4%

This study suggests that prescription opioid use is not a major contributor to the opioid crisis.

THE ONTARIO PAIN FOUNDATION
P.O. BOX 71103
501 PLAINS ROAD EAST
BURLINGTON ON L7T 2E2
905-592-1516
theontariopainfoundation@gmail.com

www.ontariopainfoundation.ca

This conclusion is borne out by a European team (*Minozzi S, Amato L, Davoli M. Development of dependence following treatment with opioid analgesics for pain relief: a systematic review. Addiction. 108: 688-698, 2013*) The data were derived from 17 studies, representing 88,235 participants. The authors concluded, "The available evidence suggests that opioid analgesics for chronic pain conditions are **not associated with a major risk for developing dependence**".

On the other hand, illicit use of opioids appears to impact negatively on the statistics regarding prescription opioid use. For example, a paper by Cicero, Ellis and Kasper (*Cicero TJ, Ellis MS, Kasper ZA. Psychoactive substance use prior to the development of iatrogenic opioid abuse: a descriptive analysis of treatment-seeking opioid abusers. Addict Behav. 65: 242-244, 2017*) states, "Our results indicate that pain patients who developed a substance use disorder were **rarely drug naïve prior to receiving their first opioid prescription**. Rather, most have an extensive history of psychoactive drug use". The same authors, in a different publication state "Our results suggest that **self-treatment of co-morbid psychiatric disturbances is a powerful motivating force to initiate and sustain abuse of opioids and that the initial source of drugs**, a prescription or experimentation, is largely irrelevant in the progression to a substance use disorder" (*Cicero TJ, Ellis MS. Understanding the demand side of the prescription opioid epidemic: does the initial source of opioids matter? Drug Alcohol Depend. 173 Suppl 1: S4-S10, 2017*).

The tenet of your letter to the drug companies suggests that the result will have negative impact on the lives of those who live with chronic pain and for whom prescription opioids allow them to live as functional lives as their conditions allow. I believe that people who live with chronic debilitating pain are being done a disservice by government policy. Reliable research evidence demonstrates that the causes underlying the current opioid crisis do not lie with legitimate prescriptions for medical uses.

I urge you to avoid government policy and directives that will have a negative impact on those Canadians whose lives are improved through the legitimate use of prescribed opioids.

Yours truly, [redacted]

James L. Henry, Ph.D.
President
The Ontario Pain Foundation
P.O. Box 71103
501 Plains Road East
Burlington ON L7T 2E2
905-592-1516

The Ontario Pain Foundation is an independent not-for-profit organization incorporated in the Province of Ontario, with the purpose to advance education and awareness for the benefit of people who live with debilitating pain. The Foundation supports the local Halton/Hamilton Chronic Pain Support Group, provides support for similar groups in Guelph and St. Catharines, and holds training courses in pain management best practices for local healthcare professionals. These activities are carried out as a community service to the 20% of our population that lives with chronic debilitating pain.

THE ONTARIO PAIN FOUNDATION
P.O. BOX 71103
501 PLAINS ROAD EAST
BURLINGTON ON L7T 2E2
905-592-1516
theontariopainfoundation@gmail.com

INNOVATIVE MEDICINES CANADA
MÉDICAMENTS NOVATEURS CANADA

AGENDA

MEETING WITH HEALTH CANADA

Attendees:

<u>Health Canada</u>:
Rhonda Kropp, Director General, Marketed Health Products Directorate
Ed Morgan, Director General, Policy, Planning and International Affairs Directorate
Lisa Lange, Director, Marketed Health Products Directorate
Amanda Moir, Director, Policy, Planning and International Affairs Directorate
Alain Musende, Manager, Marketed Health Products Directorate
Lissa Murseli, Manager, Policy, Planning and International Affairs Directorate
Kelly De Cecco, Senior Advisor to the Director General, Marketed Health Products Directorate
Christophe Roy, Senior Advisor, Marketed Health Products Directorate
Rim Lejmi Mrad, Senior Advisor, Marketed Health Products Directorate

<u>IMC</u>:
Declan Hamill, VP, Legal, Regulatory and Compliance (IMC)
Lama Abi Khaled, Executive Director, Ethics, Legal and Regulatory (IMC)
Keith McIntosh, Executive Director, Scientific and Regulatory Affairs (IMC)
Annie Bourgault, Ethics & Compliance Officer (GSK)
Crawford Wright, Chief Compliance and Privacy Officer (AZC)

Date: 10:00 am -11:00 am, Thursday July 5, 2018

Venue: Offices of Health Canada (200 Eglantine Driveway, Tunney's Pasture, Ottawa)

<u>Topics for discussion:</u>

- "The Distinction between Advertising and other Activities" Policy
    - potential changes, timeline and process

- Proactive monitoring of advertising by Health Canada
    - process and timeline
    - will there be any consultation with industry before implementation?

- Transparency of opioid marketing and advertising
    - initiatives at the Federal level
        - update and timeline
        - Transfer of Value
    - progress from IMC members

Stakeholder Meeting Report
Innovative Medicines Canada
July 5, 2018

<table>
<tr><td>Date: July 5, 2018</td><td>Meeting sheet completed by:
Policy, Planning and International Affairs Directorate</td></tr>
<tr><td>Subject :
Innovative Medicines Canada meeting with Health Canada</td><td>Stakeholder attendees:
• Declan Hamill, VP, Legal, Regulatory and Compliance (IMC)
• Lama Abi Khaled, Executive Director, Ethics, Legal and Regulatory (IMC)
• Annie Bourgault, Ethics & Compliance Officer (GSK)
• Crawford Wright, Chief Compliance and Privacy Officer (AZC)

Health Canada attendees:
• Rhonda Kropp, Director General, Marketed Health Products Directorate
• Ed Morgan, Director General, Policy, Planning and International Affairs Directorate
• Lisa Lange, Director, Marketed Health Products Directorate
• Amanda Moir, Director, Policy, Planning and International Affairs Directorate
• Alain Musende, Manager, Marketed Health Products Directorate
• Lissa Murseli, Manager, Policy, Planning and International Affairs Directorate
• Kelly De Cecco, Senior Advisor to the Director General, Marketed Health Products Directorate
• Christophe Roy, Senior Advisor, Marketed Health Products Directorate
• Rim Lejmi Mrad, Senior Advisor, Marketed Health Products Directorate</td></tr>
<tr><td colspan="2">■ Privacy notice was read and acknowledged

“We would like to remind you that this meeting is subject to disclosure as per the Health Products and Food Branch Policy on Open Decision-Making. Therefore, nothing shared during this meeting is considered confidential and personal information may be disclosed without your further consent.”</td></tr>
<tr><td colspan="2">Purpose and Agenda:
To obtain an update on Health Canada activities related to opioid marketing and advertising, such as:
• The Distinction Between Advertising and Other Activities Policy</td></tr>
</table>

- Proactive monitoring of advertising by Health Canada
- Transparency of marketing and advertising

Documents Shared by HC or stakeholders:

1. Agenda
2. Stakeholder Meeting Report

Notes:

The topics of discussion are outlined in the meeting agenda. A summary of key points is as follows:

<u>Distinction Policy:</u>
Health Canada provided an update that next steps include a consultation in the fall. IMC expressed interest in being consulted in person/verbally.

<u>Proactive monitoring of advertising by Health Canada:</u>
Health Canada indicated its is moving to a proactive approach to monitoring compliance, in addition to following up on complaints received. This approach could include, for example, Health Canada attending relevant conferences and monitoring meeting agendas for compliance. Health Canada clarified that the intent of these activities is to prevent, and proactively respond to, any non-compliances. IMC noted that it is important to reinforce positive action from industry as well.

<u>Transparency of marketing and advertising:</u>
Health Canada encouraged IMC to provide comments via the Notice of Intent, open for comments from all stakeholders until July 18, 2018. Health Canada reiterated that information submitted in response to the Notice of Intent would help inform next steps for federal action.
It was mentioned by attendees that some provinces have expressed interest in health sector transparency. IMC indicated that a national approach to transparency may be preferred to a province-by-province system. IMC provided an update on their voluntary disclosure program, with 10 companies disclosing data at an aggregated level.

Compte rendu de la réunion avec les intervenants
Médicaments novateurs Canada
Le 5 juillet 2018

<table>
<tr>
<td>Date : le
5 juillet 2018</td>
<td>Fiche de compte rendu de réunion préparée par :
Direction des politiques, de la planification et des affaires internationales</td>
</tr>
<tr>
<td>Objet :
Réunion de Médicaments novateurs Canada avec Santé Canada</td>
<td>Participants des intervenants
• Declan Hamill, vice-président, Affaires juridiques et réglementaires, et Conformité (MNC)
• Lama Abi Khaled, directrice exécutive, Éthique et Affaires juridiques et réglementaires (MNC)
• Annie Bourgault, responsable, Éthique et conformité (GSK)
• Crawford Wright, responsable, Conformité et protection des renseignements personnels (AZC)

Participants de Santé Canada
• Rhonda Kropp, directrice générale, Direction des produits de santé commercialisés
• Ed Morgan, directeur général, Direction des politiques, de la planification et des affaires internationales
• Lisa Lange, directrice, Direction des produits de santé commercialisés
• Amanda Moir, directrice générale, Direction des politiques, de la planification et des affaires internationales
• Alain Musende, gestionnaire, Direction des produits de santé commercialisés
• Lissa Murseli, gestionnaire, Direction des politiques, de la planification et des affaires internationales
• Kelly De Cecco, conseillère principale à la directrice générale, Direction des produits de santé commercialisés
• Christophe Roy, conseiller principal, Direction des produits de santé commercialisés
• Rim Lejmi Mrad, conseillère principale, Direction des produits de santé commercialisés</td>
</tr>
<tr>
<td colspan="2">■ Lecture et acceptation de l'avis de confidentialité

« Nous souhaitons vous rappeler que le contenu de la réunion pourrait être divulgué conformément à la Politique de prise de décisions ouverte de la Direction générale des produits de santé et des aliments. Par conséquent, aucun élément de la réunion n'est jugé confidentiel et tout renseignement personnel pourrait être divulgué sans votre consentement. » [Traduction]</td>
</tr>
</table>

Objectif et ordre du jour
Faire le point sur les activités de Santé Canada relatives au marketing et à la publicité sur les opioïdes, notamment :

- la Politique *Distinction entre les activités publicitaires et les autres activités*;
- la surveillance proactive de la publicité par Santé Canada;
- la transparence du marketing et de la publicité.

Documents partagés par SC ou les intervenants

1. Ordre du jour
2. Compte rendu de la réunion avec les intervenants

Notes

Les sujets de discussion sont présentés à l'ordre du jour de la réunion. Les principaux points à l'ordre du jour sont résumés ci-après.

<u>Politique Distinction</u>
Santé Canada a fait le point sur cette politique et précisé que les prochaines étapes engloberont le lancement d'une consultation à l'automne. MNC a exprimé le désir de participer à cette consultation en personne ou verbalement.

<u>Surveillance proactive de la publicité par Santé Canada</u>
Santé Canada a précisé passer d'une surveillance proactive à une surveillance de la conformité, en plus de continuer de traiter les plaintes reçues. Cette approche pourrait signifier, par exemple, que Santé Canada assistera aux conférences pertinentes et vérifiera la conformité des ordres du jour des réunions.
Santé Canada a expliqué que l'objectif de ces activités est de prévenir les non-conformités et de les régler rapidement. MNC a soulevé qu'il est également important de renforcer les mesures positives prises dans le secteur.

<u>Transparence du marketing et de la publicité</u>
Santé Canada a invité MNC à envoyer ses commentaires au sujet de l'Avis d'intention, que tous les intervenants peuvent commenter jusqu'au 14 juillet 2018. Santé Canada a rappelé que tous les commentaires formulés au sujet de l'Avis d'intention permettront de déterminer les prochaines interventions au nieau fédéral.
Les participants ont mentionné que certaines provinces ont manifesté de l'intérêt à l'endroit de la transparence du secteur de la santé. MNC a précisé qu'une approche nationale en matière de transparence pourrait être préférable à un système par province. MNC a fait le point sur son programme de divulgation volontaire, qui permet de divulguer les données regroupées de dix sociétés.

Pro Doc

Le 6 juillet 2018 **PAR COURRIEL**

L'honorable Ginette Petitpas Taylor
Ministre de la Santé du Canada
Ottawa, Ontario
K1A 0K9

Sujet : Avis d'intention de restreindre le marketing et la publicité des opioïdes

Madame la Ministre,

Pour donner suite à votre lettre du 19 juin dernier, nous vous confirmons notre engagement et notre soutien concernant la crise des opioïdes.

Nous tenons à vous informer que Pro Doc s'engage à restreindre le marketing et la publicité sur les opioïdes durant la période de consultation publique et la mise en place de nouvelles réglementations sur le sujet.

Bien que nous ayons pris cette mesure, nous demeurons fermement convaincus que les professionnels de la santé canadiens qui prescrivent ces médicaments ont besoin des renseignements les plus récents, pour s'assurer que leurs patients sont traités de façon appropriée. De ce fait, les demandes d'information sur nos produits opioïdes par des professionnels de la santé seront traitées par une communication directe par nos professionnels de la santé expérimentés de notre département de pharmacovigilance.

Soyez assurée, Madame la Ministre, que Pro Doc respectera ses engagements et que vous pouvez compter sur notre support concernant tout enjeu lié à la santé publique.

Veuillez agréer, Madame la Ministre, l'expression de mon profond respect.

[redacted]

Marcel A. Raymond, CPA, CA
Président

ECD Health Canada
DCHG Santé Canada
Rec'd Reçu JUL 17 2018
18-004383-642

Petitpas Taylor, Minister / Ministre Ginette (HC/SC)

From: Mark Mantel [redacted]@teligent.com>
Sent: 2018-07-06 1:17 PM
To: Petitpas Taylor, Minister / Ministre Ginette (HC/SC)
Cc: Anneli Simm; Erin Anderson; [redacted]@trademarkcreative.ca; Bianca Marchione; Christine Kenney; Jason Walman; Josey Hobbs; Kayla Kolacz; Kevin Houlihan; Lisa Sorensen; Mariam Benyamin; Maya Raman; Sandeep Shrivastava
Subject: RE: Minister of Health Letter to Pharmaceutical Industry: Marketing and Advertising of Opioids
Attachments: Products Brochure Teligent.pdf

Good day Minister,

Please take this as confirmation that Teligent Canada will not publish any marketing materials that are related to opioid based products.

Our immediate action will be to remove the Fentanyl product page from our website and remove it from our web based product list.
We will also remove fentanyl from our public catalogue that is on our website.

We do ask your opinion on keeping it in our catalogue for hospital based use. We would continue to carry this in our product catalogue as its used as a reference for ordering in the hospital and critical care setting. Please see our catalogue attached for your review and feedback.

It is our long term intent **to NOT manufacture fentanyl** should other analgesic products come available for the hospital and critical care setting. That being said our product is currently in need for hospitals across the country and we are one of only two manufacturers that make 2ml ampules.

We are also a manufacture of Naloxone and currently have partnerships across the country where we make Naloxone kits for emergency settings.

We are pleased to see your initiatives and will work with you in any way we can to bring better outcomes to Canadians and end the Opioid crisis.

Our team is at your disposal and please do not hesitate to provide feedback on above or call with any questions you may have.

Regards,

Mark Mantel / General Manager

Teligent
Suite 1500, 10 King Street East
Toronto, Ontario, Canada
M5C 1C3

T 1 365 886 1000
F 1 800 656 0794
www.teligent.com

This message is private and confidential. If you have received this message in error, please notify us and remove it from your system.

L'information apparaissait dans ce message électronique est de nature confidentielle et privilégiée . Si ce message vous est parvenu par erreur, vous êtes donc prié de nous en informer et de détruire ce message.

From: Petitpas Taylor, Minister / Ministre Ginette (HC/SC) <hcminister.ministresc@canada.ca>
Sent: Tuesday, June 19, 2018 11:07 AM
To: Mark Mantel <[redacted]@teligent.com>
Subject: Minister of Health Letter to Pharmaceutical Industry: Marketing and Advertising of Opioids

Please find attached correspondence from the Minister of Health Canada.

Thank you / Merci
Health Canada - Santé Canada

Teligent

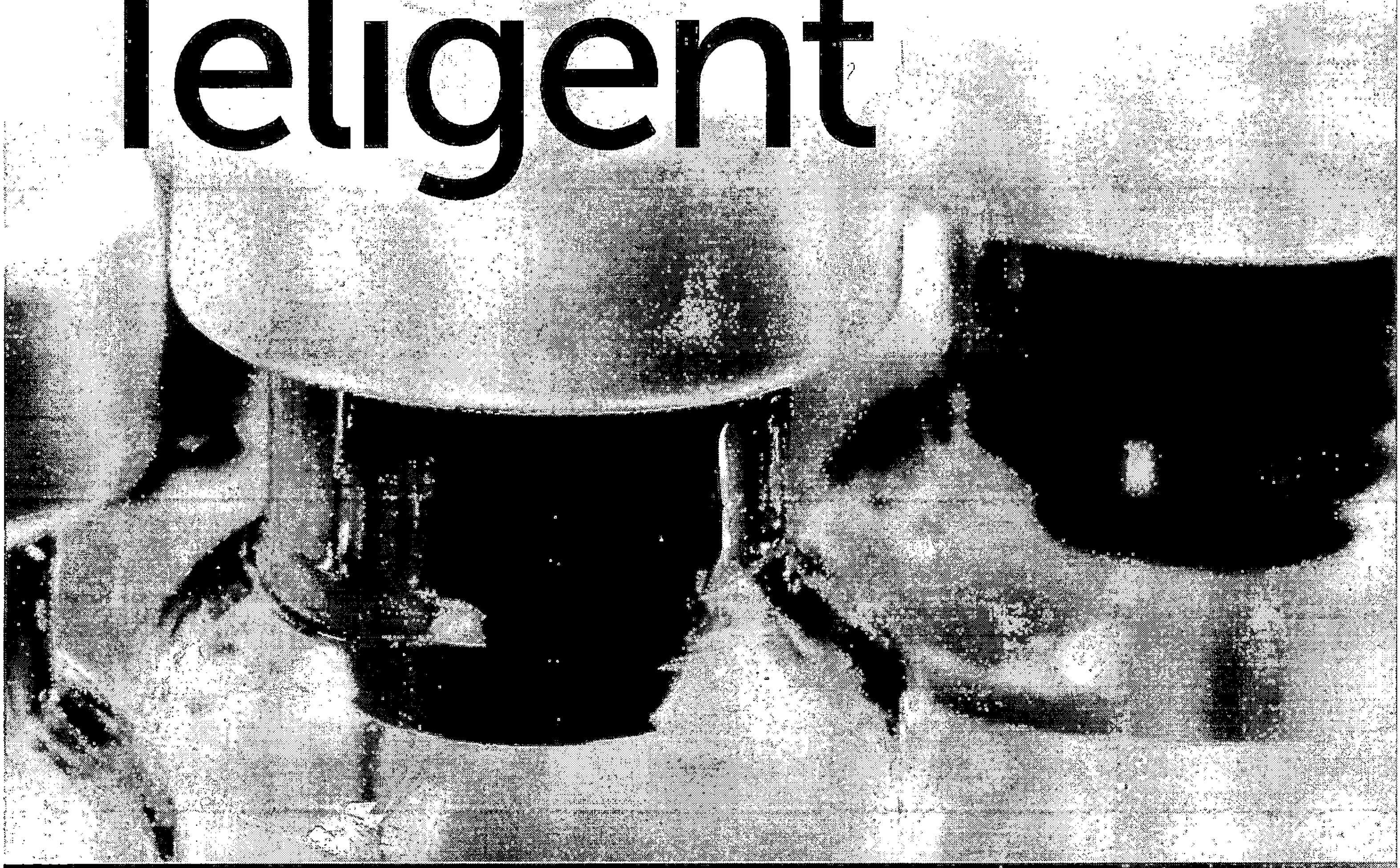

PRODUCT CATALOGUE

Teligent

OUR MISSION IS TO BECOME A LEADING MEMBER OF THE GLOBAL SPECIALTY GENERIC INDUSTRY BY 2020.

We develop, manufacture and market pharmaceutical products for use by doctors and patients in the United States and Canada.

We are a dynamic group of about 200 employees based in New Jersey, Toronto, Montreal and Tallinn, Estonia. Our Product Development and Manufacturing complex is in Buena, New Jersey, approximately 45 minutes from Philadelphia. In 2015 we acquired Alveda Pharmaceuticals Inc., a Toronto-based pharmaceutical company focused on the Canadian injectable pharmaceutical market. In 2016 we hired our first European colleagues in Tallinn who support our supply chain and quality operations.

Our Quality, Regulatory Affairs and Operations colleagues work closely with our Product Development team to achieve regulatory approval in the countries where we operate. Our Commercial team distributes our portfolio of topical medicines and hospital-based sterile injectable products.

We are energized about what we are going to accomplish at Teligent today. We believe in taking ownership, driving execution, and making smart, quick decisions.

We are passionate about our science, and are committed to building a diverse pipeline of topical, injectable, complex and ophthalmic projects.

PRODUCT LIST

ACETYLCYSTEINE SOLUTION USP

ATROPINE INJECTION BP

BACLOFEN INJECTION

CYANOCOBALAMIN INJECTION USP

DIAZEPAM INJECTION USP

DIMENHYDRINATE INJECTION USP

DIMENHYDRINATE INJECTION USP WITH PRESERVATIVE

DOBUTAMINE INJECTION USP

EPINEPHRINE INJECTION

ERGONOVINE MALEATE INJECTION

EUFLEXXA – 1% SODIUM HYALURONATE SYRINGES

FENTANYL CITRATE INJECTION USP

FUROSEMIDE INJECTION USP

GENTAMICIN INJECTION USP

LIDOCAINE HCl INJECTION (1%, 2%)

LIDOCAINE HCl INJECTION WITH PRESERVATIVE (1%, 2%)

LIDOCAINE 2% AND EPINEPHRINE 1:100,000 INJECTION

NALOXONE HYDROCHLORIDE INJECTION USP

NALOXONE INJECTABLE (NON-Rx)

PIPERACILLIN AND TAZOBACTAM FOR INJECTION

SODIUM CHLORIDE INJECTION USP 0.9%

STERILE WATER FOR INJECTION USP

SUCCINYLCHOLINE CHLORIDE INJECTION

Teligent

PRODUCT CATALOGUE

ACETYLCYSTEINE SOLUTION USP

No.	Description	Strength	Concentration	Fill Volume	Units Per Pack	DIN	Pack Bar Code	Unit of Use Bar Code	McKesson Catalogue Number	Kohl and Frisch Item Number
0015AF01	Clear Glass Vial	2 G	200 mg/mL	10 mL	10	02459906	837641000256	(01) 00837641010255	424572	131147
0015AI01	Clear Glass Vial	6 G	200 mg/mL	30 mL	10	02459906	837641000263	(01) 00837641010262	694133	137387

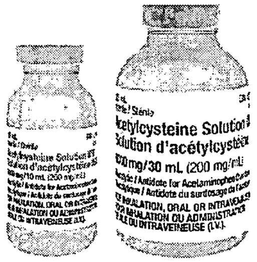

ATROPINE INJECTION BP

No.	Description	Strength	Concentration	Fill Volume	Units Per Pack	DIN	Pack Bar Code	Unit of Use Bar Code	McKesson Catalogue Number	Kohl and Frisch Item Number
0010AA01	Clear Glass Ampoule	0.4 mg	0.4 mg/mL	1 mL	10	02432188	837641000300	(01) 00837641010309	749598	131149
0011AA01	Clear Glass Ampoule	0.6 mg	0.6 mg/mL	1 mL	10	02432196	837641000317	(01) 00837641010316	749580	159291

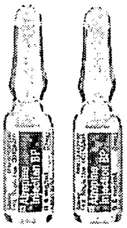

BACLOFEN INJECTION

No.	Description	Strength	Concentration	Fill Volume	Units Per Pack	DIN	Pack Bar Code	Unit of Use Bar Code	McKesson Catalogue Number	Kohl and Frisch Item Number
0020AA01	Clear Glass Ampoule	0.05 mg	0.05 mg/mL	1 mL	10	02457059	837641000799	(01) 00837641010798	TBA	TBA
0022AG01	Clear Glass Ampoule	10 mg	0.5 mg/mL	20 mL	1	02457067	837641000805	(01) 00837641010804	TBA	TBA
0024AD01	Clear Glass Ampoule	10 mg	2 mg/mL	5 mL	10	02457075	837641000812	(01) 00837641010811	TBA	TBA
0024AG01	Clear Glass Ampoule	40 mg	2 mg/mL	20 mL	1	02457075	837641000829	(01) 00837641010828	TBA	TBA

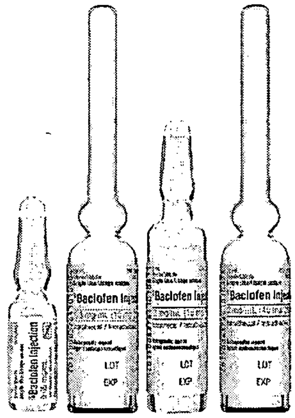

Teligent

ALL PRODUCTS ARE LATEX FREE UNLESS OTHERWISE NOTED

CYANOCOBALAMIN INJECTION USP

No.	Description	Strength	Concentration	Fill Volume	Units Per Pack	DIN	Pack Bar Code	Unit of Use Bar Code	McKesson Catalogue Number	Kohl and Frisch Item Number
0034AA01	Amber Glass Ampoule	1 mg	1 mg/mL	1 mL	10	02463393	837641000508	(01) 00837641010507	TBA	TBA
0034AF01	Amber Glass Multidose Vial	10 mg	1 mg/mL	10 mL	1	02465507	837641000126	(01) 00837641010125	TBA	TBA

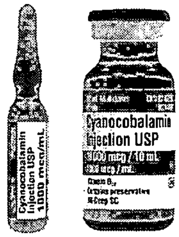

DIAZEPAM INJECTION USP

No.	Description	Strength	Concentration	Fill Volume	Units Per Pack	DIN	Pack Bar Code	Unit of Use Bar Code	McKesson Catalogue Number	Kohl and Frisch Item Number
0042AB01	Amber Glass Ampoule	10 mg	5 mg/mL	2 mL	10	02385392	837641000522	(01) 00837641010521	111981	TBA

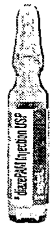

DIMENHYDRINATE INJECTION USP – PRESERVATIVE FREE

No.	Description	Strength	Concentration	Fill Volume	Units Per Pack	DIN	Pack Bar Code	Unit of Use Bar Code	McKesson Catalogue Number	Kohl and Frisch Item Number
0040AA01	Clear Glass Ampoule	50 mg	50 mg/mL	1 mL	10	02428954	837641000225	(01) 00837641010224	447979	131153

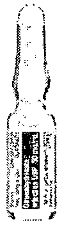

PRODUCT CATALOGUE

DIMENHYDRINATE INJECTION USP WITH PRESERVATIVE

No.	Description	Strength	Concentration	Fill Volume	Units Per Pack	DIN	Pack Bar Code	Unit of Use Bar Code	McKesson Catalogue Number	Kohl and Frisch Item Number
0040AD01	Clear Glass Multidose Vial	250 mg	50 mg/mL	5 mL	10	02435241	837641000218	(01) 00837641010217	189290	134533

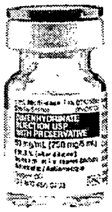

DOBUTAMINE INJECTION USP

No.	Description	Strength	Concentration	Fill Volume	Units Per Pack	DIN	Pack Bar Code	Unit of Use Bar Code	McKesson Catalogue Number	Kohl and Frisch Item Number
0043AG01	Clear Glass Single Use Vial	250 mg	12.5 mg/mL	20 mL	10	02462729	837641000843	(01) 00837641010842	TBA	TBA

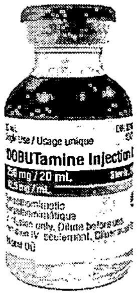

EPINEPHRINE INJECTION

No.	Description	Strength	Concentration	Fill Volume	Units Per Pack	DIN	Pack Bar Code	Unit of Use Bar Code	McKesson Catalogue Number	Kohl and Frisch Item Number
1293AA01	Amber Glass Ampoule	1 mg	1 mg/mL	1 mL	10	02435810	837641000454	(01) 00837641010453	981845	153389

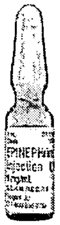

PRODUCT CATALOGUE

ERGONOVINE MALEATE INJECTION

No.	Description	Strength	Concentration	Fill Volume	Units Per Pack	DIN	Pack Bar Code	Unit of Use Bar Code	McKesson Catalogue Number	Kohl and Frisch Item Number
0050AA01	Amber Glass Ampoule	0.25 mg	0.25 mg/mL	1 mL	5	02441241	837641000010	(01) 00837641010019	607226	131155

** Non-returnable / Ref. 2 - 8 °C*

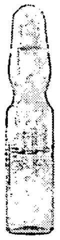

EUFLEXXA – 1% SODIUM HYALURONATE SYRINGES

No.	Description	Strength	Concentration	Fill Volume	Units Per Pack	DIN	Pack Bar Code	Unit of Use Bar Code	McKesson Catalogue Number	Kohl and Frisch Item Number
0138AB01	Syringe	20mg	10mg/mL	2 mL	3	N/A	779170144202	N/A	260992	93807

** Parts of the syringe contain natural rubber latex which may cause allergic reactions in latex-senstive individuals / Non-returnable*

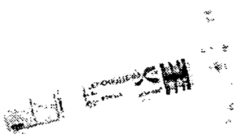

Ⓝ FENTANYL CITRATE INJECTION USP

No.	Description	Strength	Concentration	Fill Volume	Units Per Pack	DIN	Pack Bar Code	Unit of Use Bar Code	McKesson Catalogue Number	Kohl and Frisch Item Number
0062AB01	Clear Glass Ampoule	100 mcg	50 mcg/mL	2 mL	10	02385406	837641000546	(01) 00837641010545	095486	TBA

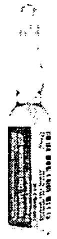

FUROSEMIDE INJECTION USP

No.	Description	Strength	Concentration	Fill Volume	Units Per Pack	DIN	Pack Bar Code	Unit of Use Bar Code	McKesson Catalogue Number	Kohl and Frisch Item Number
0060AB01	Amber Glass Ampoule	20 mg	10 mg/mL	2 mL	10	02384094	837641000584	(01) 00837641010583	49063	TBA

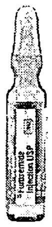

PRODUCT CATALOGUE

GENTAMICIN INJECTION USP

No.	Description	Strength	Concentration	Fill Volume	Units Per Pack	DIN	Pack Bar Code	Unit of Use Bar Code	McKesson Catalogue Number	Kohl and Frisch Item Number
0072AB01	Clear Glass Ampoule	20 mg	10 mg/mL	2 mL	10	02470462	837641001062	(01) 00837641011061	TBA	TBA
0073AB01	Clear Glass Ampoule	80 mg	40 mg/mL	2 mL	10	02457008	837641000782	(01) 00837641010781	105559	TBA

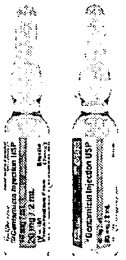

LIDOCAINE HCl INJECTION (1% PRESERVATIVE FREE)

No.	Description	Strength	Concentration	Fill Volume	Units Per Pack	DIN	Pack Bar Code	Unit of Use Bar Code	McKesson Catalogue Number	Kohl and Frisch Item Number
0121AD01	Clear Polyampoule	50 mg	10 mg/mL	5 mL	20	02421984	837641000034	(01) 00837641010033	701938	155537
0121AF01	Clear Polyampoule	100 mg	10 mg/mL	10 mL	20	02421984	837641000041	(01) 00837641010040	701987	131158
1177AD01	Clear Glass Ampoule	50 mg	10 mg/mL	5 mL	25	02421984	837641001055	(01) 00837641011054	TBA	136628

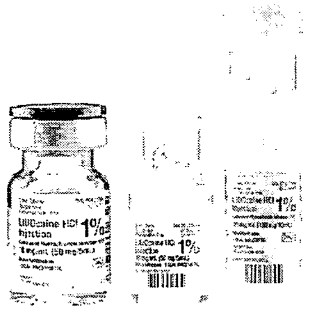

LIDOCAINE HCl INJECTION WITH PRESERVATIVE (1%)

No.	Description	Strength	Concentration	Fill Volume	Units Per Pack	DIN	Pack Bar Code	Unit of Use Bar Code	McKesson Catalogue Number	Kohl and Frisch Item Number
0122AG01	Clear Glass Multidose Vial	200 mg	10 mg/mL	20 mL	10	02422018	837641000058	(01) 00837641010057	702068	131156
0122AJ01	Clear Glass Multidose Vial	500 mg	10 mg/mL	50 mL	10	02422018	837641000065	(01) 00837641010064	706473	131157

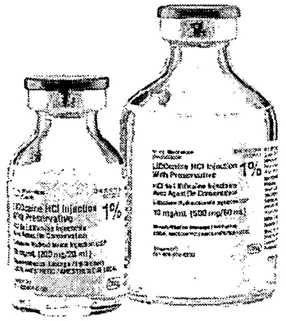

Teligent

ALL PRODUCTS ARE LATEX FREE UNLESS OTHERWISE NOTED

PRODUCT CATALOGUE

LIDOCAINE HCl INJECTION (2% PRESERVATIVE FREE)

No.	Description	Strength	Concentration	Fill Volume	Units Per Pack	DIN	Pack Bar Code	Unit of Use Bar Code	McKesson Catalogue Number	Kohl and Frisch Item Number
0126AD01	Clear Polyampoule	100 mg	20 mg/mL	5 mL	20	02421992	837641000089	(01) 00837641010088	713289	157472
0126AF01	Clear Polyampoule	200 mg	20 mg/mL	10 mL	20	02421992	837641000096	(01) 00837641010095	714386	131163

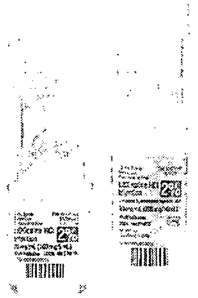

LIDOCAINE HCl INJECTION WITH PRESERVATIVE (2%)

No.	Description	Strength	Concentration	Fill Volume	Units Per Pack	DIN	Pack Bar Code	Unit of Use Bar Code	McKesson Catalogue Number	Kohl and Frisch Item Number
0127AG01	Clear Glass Multidose Vial	400 mg	20 mg/mL	20 mL	10	02422026	837641000102	(01) 00837641010101	715482	157588
0127AJ01	Clear Glass Multidose Vial	1 G	20 mg/mL	50 mL	10	02422026	837641000119	(01) 00837641010118	720482	TBA

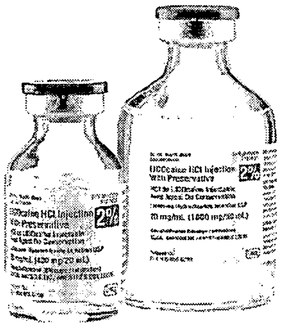

LIDOCAINE 2% AND EPINEPHRINE 1:100,000 INJECTION

No.	Description	Strength	Concentration	Fill Volume	Units Per Pack	DIN	Pack Bar Code	Unit of Use Bar Code	McKesson Catalogue Number	Kohl and Frisch Item Number
0128AG01	Amber Glass Multidose Vial	400 mg & 0.2 mg	20 mg/mL & 0.01 mg/mL	20 mL	1	02436221	837641000140	(01) 00837641010149	720573	155538
0128AJ01	Amber Glass Multidose Vial	1 G & 0.5 mg	20 mg/mL & 0.01 mg/mL	50 mL	1	02436221	837641000157	(01) 00837641010156	720631	131167

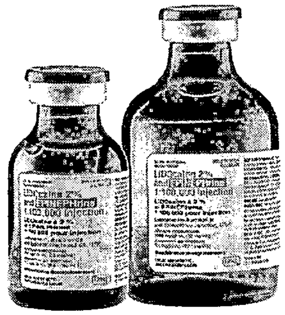

NALOXONE HYDROCHLORIDE INJECTION USP

No.	Description	Strength	Concentration	Fill Volume	Units Per Pack	DIN	Pack Bar Code	Unit of Use Bar Code	McKesson Catalogue Number	Kohl and Frisch Item Number
0140AA01	Amber Glass Ampoule	0.4 mg	0.4 mg/mL	1 mL	10	02382482	837641000607	(01) 00837641010606	069322	TBA

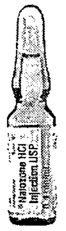

NALOXONE INJECTABLE (NON-PRESCRIPTION)

No.	Description	Strength	Concentration	Fill Volume	Units Per Pack	DIN	Pack Bar Code	Unit of Use Bar Code	McKesson Catalogue Number	Kohl and Frisch Item Number
0141AA01	Amber Glass Ampoule	0.4 mg	0.4mg/mL	1 mL	10	02458578	837641001048	(01) 00837641011047	TBA	TBA

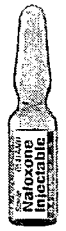

PIPERACILLIN AND TAZOBACTAM FOR INJECTION

No.	Description	Strength	Concentration	Fill Volume	Units Per Pack	DIN	Pack Bar Code	Unit of Use Bar Code	McKesson Catalogue Number	Kohl and Frisch Item Number
0160AF01	Clear Glass Vial	2 g & 0.25 g	n/a	2.25 g	10	02401312	837641000751	(01) 00837641010750	079735	TBA
0160AG01	Clear Glass Vial	3 g & 0.375 g	n/a	3.375 g	10	02401320	837641000768	(01) 00837641010767	079734	TBA
0160AH01	Clear Glass Vial	4 g & 0.5 g	n/a	4.5 g	10	02401339	837641000775	(01) 00837641010774	079736	TBA

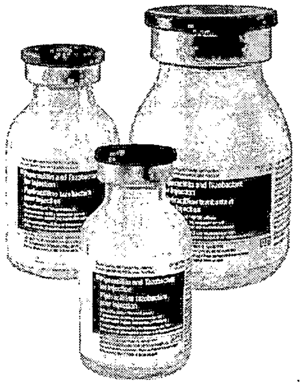

PRODUCT CATALOGUE

SODIUM CHLORIDE INJECTION USP 0.9%

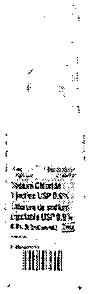

No.	Description	Strength	Concentration	Fill Volume	Units Per Pack	DIN	Pack Bar Code	Unit of Use Bar Code	McKesson Catalogue Number	Kohl and Frisch Item Number
0195AF01	Clear Polyampoule	90 mg	9 mg/mL	10 mL	20	02304341	837641000430	(01) 00837641010439	941955	131169

STERILE WATER FOR INJECTION USP

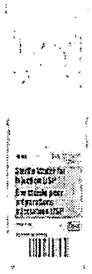

No.	Description	Strength	Concentration	Fill Volume	Units Per Pack	DIN	Pack Bar Code	Unit of Use Bar Code	McKesson Catalogue Number	Kohl and Frisch Item Number
0230AF01	Clear Polyampoule	100%	N/A	10 mL	20	02299186	837641000447	(01) 00837641010446	336644	131332

SUCCINYLCHOLINE CHLORIDE INJECTION

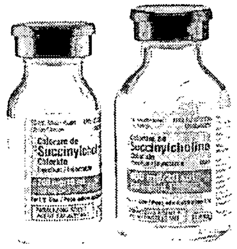

No.	Description	Strength	Concentration	Fill Volume	Units Per Pack	DIN	Pack Bar Code	Unit of Use Bar Code	McKesson Catalogue Number	Kohl and Frisch Item Number
0190AF01	Clear Glass Multidose Vial	200 mg	20 mg/mL	10 mL	10	02422336	837641000287	(01) 00837641010286	030142	134310
0190AG01	Clear Glass Multidose Vial	400 mg	20 mg/mL	20 mL	10	02422336	837641000294	(01) 00837641010293	030143	TBA

** Non-returnable / Ref. 2 - 8 °C*

Teligent

ALL PRODUCTS ARE LATEX FREE UNLESS OTHERWISE NOTED

For terms of sale and return goods information, please visit www.teligent.ca

Teligent Canada Inc.
10 King Street East, Suite 1500
Toronto, ON M5C 1C3

1-800-656-0793
canada_customerservice@teligent.com
communications@teligent.com

www.teligent.ca

Version 5.0.6

INNOVATIVE MEDICINES CANADA | MÉDICAMENTS NOVATEURS CANADA

President's Office | Bureau du Président

July 9, 2018

The Honourable Ginette Petitpas Taylor, PC, MP
Minister of Health
House of Commons
Ottawa, ON K1A 0A6
Ginette.PetitpasTaylor@parl.gc.ca

Dear Minister Petitpas Taylor:

On behalf of Innovative Medicines Canada, thank you for your letter of June 19th, 2018, regarding Canada's collective response to the opioid crisis. Innovative Medicines Canada and our members are deeply concerned about this issue and, of course, support your goal to "re-centre the pendulum" on opioid prescribing.

It is our firm belief that a collaborative approach provides both industry and legislators with the opportunity to successfully deliver innovative solutions that can reduce or prevent risk and harm to Canadians. I have shared your letter with our membership and discussions will continue with our Board of Directors. As you know, one of our members, Purdue Pharma (Canada), has responded positively in response to your call for action and has suspended all promotional and advertising activities to their prescription opioids, pending the outcome of the consultations and the implementation of regulations.

We support the views from your department that access to a broad range of safe, effective, and high-quality medicines is important to Canadians. Guided by our Code of Ethical Practices, our members are committed to ethical conduct in their interactions with healthcare professionals and with patients. We also agree that policy and practice should seek to minimize risks and harms, while ensuring that medicines are available to the patient who needs them. Innovative Medicines Canada is also preparing a detailed response to the *Notice of Intent*.

As you know, outside of this consultation process, Innovative Medicines Canada recently offered to facilitate a roundtable with our relevant members and Health Canada staff to discuss the role pharmaceutical innovation can play as part of the solution to the opioid crisis. We look forward to hearing back from departmental officials on this initiative.

Given the critical and urgent nature of this issue, please feel free to reach out to me directly, as needed, at [redacted]@imc-mnc.ca or 613-236- 0455 ext. [redacted]

Sincerely,

[redacted]

Pamela C. Fralick
President

cc: Simon Kennedy, Deputy Minister, Health Canada

Chris Halyk
President

19 Green Belt Drive
Toronto, ON M3C 1L9
1.800.387.8781 toll free 416.382.4888 tel
416.382.4889 fax

www.janssen.ca

janssen
PHARMACEUTICAL COMPANIES OF Johnson & Johnson

July 11, 2018 CH-GEN-139

Hon. Ginette Petitpas Taylor, P.C., M.P.
Minister of Health
Health Canada
Ottawa, ON K1A 0K9

Email: hcminister.ministresc@canada.ca

Dear Minister Petitpas Taylor,

I wish to acknowledge receipt of your letter on June 19, 2018, expressing Health Canada's concern about the appropriate use of opioids in Canada, and requesting the immediate and voluntary suspension of marketing and advertising of opioids to health care professionals.

Opioid abuse and addiction are serious public health issues. We are committed to patient safety and will continue to work with Health Canada to help ensure the safe and appropriate use of prescription opioid-containing medicines. As Health Canada acknowledged in its "*Notice of Intent to Restrict the Marketing and Advertising of Opioids*", "[p]rescription opioids can help Canadians who need them to manage pain", but it is important that these medications are used appropriately, under the care of a physician, and in accordance with their approved labelling that provides important information about the product's risks and benefits.

The safety and well-being of patients who use Janssen products is our highest priority. I can confirm Janssen Inc. ("Janssen") has not promoted any of its opioid brands in Canada since 2011.

Finding solutions to opioid abuse and addiction requires collaboration among many stakeholders. We support Health Canada's initiative to seek consultation from multiple stakeholders to find a suitable regulatory pathway that effectively balances the risks and benefits of prescription opioids with the need to ensure appropriate and safe prescribing of these medications to effectively treat Canadian patients suffering with pain.

Sincerely,

[redacted]

Chris Halyk
President
Janssen Inc.

John Helou
Président
Pfizer Canada inc.
C.P. 800
Pointe-Claire / Dorval (Québec) H9R 4V2
Tél. : 514 693 4563 Téléc. : 514 426 7084

Kirkland, July 13, 2018

The Honourable Ginette Petitpas-Taylor, P.C., M.P.
Minister of Health
70 Colombine Driveway, Tunney's Pasture
Ottawa, Ontario K1A 0K9

Via email: hcminister.ministresc@canada.ca

Dear Minister:

On behalf of Pfizer Canada Inc., thank you for your letter of June 19th regarding your intention to restrict the marketing and advertising of opioids and your request to immediately suspend those activities in Canada.

We recognize the seriousness of the opioids public health crisis and we commend the leadership that governments across the country are taking in order to address it. Pfizer Canada wants to assure you of our commitment to collaborate with Health Canada in this endeavor.

Regarding the marketing and advertising of our product portfolio, Pfizer Canada complies rigorously with all relevant laws, regulations, and Codes currently enforced in Canada under the *Foods and Drugs Act & Regulations*; the Pharmaceutical Advertising Advisory Board (PAAB) Code; Ad Standards; and Innovative Medicines Canada's *Code of Ethical Practices*.

Following the reception of your letter, we have carefully reviewed our current prescription and non-prescription portfolio. I can confirm that we do not engage in the marketing or advertising of any of our opioid containing products and Pfizer will continue to restrict such practices for these products. Our current commercial and medical activities for our opioid products are restricted to:

- Communications relating to pricing and availability of supply;
- Product Monographs available on Pfizer website: Pfizer.ca
- Listing of product availabilities on Pfizer website: Pfizerinjectables.ca;
- Listing of website name (Pfizerinjectables.ca) on Pfizer business cards;
- Reactive responses from Pfizer Medical Information in response to medical queries.

As we strongly believe in the importance of appropriate use of medicines and vaccines, we will continue to share helpful educational and scientific information on these products in compliance with the marketing authorizations received from Health Canada. As such, our medical affairs experts remain available to respond to questions by healthcare professionals and our products remain listed on our websites. We do not consider the foregoing activities to be marketing or advertising. Pfizer will, of course, rigorously adhere to any future restrictions the Government puts in place, by regulation or otherwise.

Pfizer has a rich history in the research and development of innovative non-opioid therapies for the management of pain. Advil, Celebrex and Lyrica are non-opioid analgesics that continue to help Canadians manage inflammatory pain (Advil & Celebrex) and pain associated with diabetic peripheral neuropathy, post-herpetic neuralgia and fibromyalgia (Lyrica).

Currently our development pipeline for pain includes a novel non-opioid injectable treatment that is based on a new mechanism of action. This investigational innovative medicine was granted Fast Track designation by the FDA in June 2017 for the treatment of chronic pain in patients with osteoarthritis and chronic low back pain. Pfizer Canada also partners with reputable organizations such as the Canadian Pain Society to accelerate the science in the field of pain by supporting independent, basic, biomedical, clinical and health sciences research.

I trust that you will find our reply to your satisfaction.

Yours sincerely,

[redacted]

John Helou
President, Pfizer Canada Inc.

C.C.: Vratislav Hadrava, MD, PhD, Country Medical Director & IM Medical Affairs Lead, Pfizer Canada Inc.

July 17, 2018

The Honourable Ginette Petitpas Taylor, P.C, M.P.
Minster of Health
House of Commons
Ottawa, Ontario K1A 0K9

Dear Minister:

Thank you for your letter dated June 19, 2018 requesting that the industry show leadership in helping to mitigate the effects of the current opioid crisis by immediately suspending marketing and advertising of opioids.

Like all Canadians, the Canadian Generic Pharmaceutical Association (CGPA) and its member companies and their employees are deeply concerned about this urgent public health issue. We support the Government of Canada's efforts to address the problem of opioid abuse and look forward to continuing to work with the federal, provincial and territorial governments, and all stakeholders, to better address this urgent problem.

Due to the nature of our business generic pharmaceutical manufacturers do not engage in the practice of advertising and / or marketing opioids, other narcotic products or any other prescription medicines to physicians and / or the public in Canada. As a point of clarification, it is generally the brand name pharmaceutical companies that engage in the practice of advertising and / or marketing to physicians on the advantages of their respective opioid products.

Generic pharmaceutical firms manufacture opioids and sell them directly to pharmacies or wholesalers. It is only when a patient receives an opioid prescription from their physician and brings it to the pharmacy that the pharmacist may choose to dispense a generic opioid in place of a brand opioid. The dispensing of a generic is based on demonstrated bioequivalence, as per Health Canada's 'Declaration of equivalence', with the brand name product. As such, CGPA's companies have no role whatsoever in the decision made by a physician or other health care professional in prescribing an opioid in the first place.

I can assure you that CGPA member companies will continue their current practice of not marketing or advertising opioids to physicians and / or the public in Canada as per the request in your June 19, 2018 letter.

We do wish to raise with you one matter that should be clarified in any legislative or regulatory approach to the marketing and advertising of opioids. As noted above, it is the pharmacist who has professional discretion to dispense a generic opioid in place of a prescribed brand name opioid. Similarly, private wholesalers or government and private payers have the discretion whether to purchase or pay for generic opioids. In exercising this discretion, the pharmacist, purchaser or payer must be satisfied that the generic opioid is bioequivalent to the brand name product.

.../2

Declarations of equivalence are issued by Health Canada. In notifying pharmacists, purchasers or payers of the availability of a generic opioid, CGPA companies are advising these bodies that the generic has been approved by Health Canada as bioequivalent to the brand name product and is available for ordering. In so advising these bodies, CGPA companies will provide product monographs. We do not believe that the intent of the Government in restricting the marketing and advertising of opioids is to prevent generic manufacturers from advising other bodies of Health Canada's declaration of bioequivalence or providing product monographs. Therefore, Health Canada should ensure that this matter is clear and that generic manufacturers can continue to advise other bodies of the bioequivalence and availability of their products with the brand name versions and continue to provide product monographs.

Based on the best information currently available, we believe that a key public health issue is the overprescribing of opioids. Member companies of CGPA, therefore, support efforts to educate physicians on appropriate prescribing and patient education on appropriate use and safe storage to prevent diversion. Focusing efforts towards proposals that address the root of the problem, such as better control and guidance at the prescriber and patient level, that would lead to a more complete long-term solution, while maintaining adequate access for Canadians that require pain medication.

An example of the latter is the Ontario Government's multifaceted narcotic strategy with their Narcotics Monitoring System. This monitoring system is designed to help monitor the prescribing and dispensing activity with the goal of reducing misuse, addiction, unlawful activities and deaths related to narcotic medications. The information collected will help promote appropriate prescribing, dispensing and use of narcotics and other controlled drugs.

CGPA and its member companies also note that the Government of Canada has introduced new regulations requiring Canadian Specific Opioid targeted Risk Management Plans (RMPs) for opioid medicines. These RMPs will "standardize and strengthen the rigor of the post-market surveillance of prescription opioids, allowing better quantification and characterization of the risks associated with opioid-related harms in Canadian patients and put in place targeted risk minimization activities to prevent or decrease prescription opioid-related harms in Canada."

From an industry perspective, it is important to note that Canada's Narcotic Control Regulations prescribe the conditions under which narcotics can be imported, exported and manipulated and to who they can be transferred and sold. Narcotics, such as opioids, are tightly controlled from importing, handling, storage, manufacturing, distribution, through to selling thus minimizing the potential for diversion.

Anyone handling narcotics must be issued a license by Health Canada, which:

- Must be renewed annually
- Specifies the permitted molecules
- Specifies permitted activities, such as manufacturing, packaging and distributing
- Specifies security levels required; dependent upon the molecule and its illicit value, as well as the location of the facility

Security requirements vary from Level 1 (lowest) to 11 (highest). The following are some examples of the controls required:

- Certain molecules may be stored in a locked cage but typically a cement block vault is required for storage; the latter must meet specific construction criteria.

- Only identified people have access to the locked area.
- Material is returned to the vault after every manipulation and stored there
- Inventory / weight control is performed after every operation; inventory discrepancies are investigated internally. Where notification to Health Canada is required, the reporting occurs within the timeframe specified by the Regulations relevant to substance.
- Controlled substances can only be transferred from one license holder to another license holder.
- Permits must be obtained from Health Canada to import and export controlled substances.
- Every licensed dealer is required to keep a record of:
    - the name and quantity of any narcotic received by the licensed dealer,
    - the name and address of the person who sold or provided it and the date on which it was received;
    - the name, quantity and form of any narcotic sold or provided by the licensed dealer,
    - the name and address of the person to whom it was sold or provided and the date on which it was sold or provided;
    - the name and quantity of any narcotic used in the making or assembling of a product or compound containing that narcotic,
    - the name and quantity of the product or compound made or assembled and the date on which the product or compound was placed in stock;
    - the name and quantity of any narcotic produced and the date on which it was placed in stock; and
    - the name and quantity of any narcotic in stock at the end of each month. (Narcotic Control Regulations)

CGPA is pleased to advise that, to date, our member companies report that there have been no instances of diversion of opioids while in the companies' control. Our companies are not aware of any subsequent diversion in the supply chain but note that that is not within companies' control.

CGPA and its member companies are actively trying to minimize the abuse and misuse of opioids within their sphere of control. Thank you, again, for your letter of June 19. Should you or your officials have any questions regarding any of the information contained in this letter please do not hesitate to contact me.

Sincerely, [redacted]

Jim Keon,
President

SANOFI

July 18, 2018

The Honourable Ginette Petitpas Taylor, P.C., M.P.
Minister of Health
Broke Claxton Building, Tunney's Pasture
Ottawa, On
K1A0K9
ginette.petitpasTaylor@parl.gc.ca

Re: June 19, 2018 letter regarding marketing/advertising activities on opioids

Dear Minister Petitpas Taylor:

This is to acknowledge your letter dated June 19, 2018 requiring manufacturers of opioids to suspend marketing and advertising activities on opioids to health care professionals. Sanofi-aventis Canada Inc. ("Sanofi") hereby confirms that we do not engage in any marketing/advertising activities for the prescription opioid products (listed below) for which Sanofi is the marketing authorization holder.

DIN	Brand Name	Generic name
02138018	Demerol	meperidine hcl
02049481	Novahistex DH	hydrocodone bitartrate & phenylephrine hcl
02049473	Novahistine DH	hydrocodone bitartrate & phenylephrine hcl
02137984	Talwin	pentazocine hcl
01916971	Tussionex	hydrocodone/phenyltoloxamine

Sanofi Canada recognizes that non-medical use, misuse, abuse and diversion of prescription medicines, including prescription opioids are a major public health crisis in this country. We are keen to continue working with all levels of government, with patients, health professionals and other stakeholders to find solutions to help mitigate the opioid crisis.

We trust that this is satisfactory. If you have any questions, please do not hesitate to contact Carrie Ku, Head of Regulatory Affairs, at [redacted]

Yours sincerely,

[redacted]

Niven Al-Khoury
President and Chief Executive Officer
[redacted]@sanofi.com

Mylan

85 Advance Road
Etobicoke, ON M8Z 2S6
Toll-Free 1.800.668.3174
Phone 416.236.2631
Fax 416.236.2940
Web www.mylan.ca

July 19, 2018

ECD Health Canada
DCHG Santé Canada
Rec'd Reçu JUL 27 2018
18-004578-218

The Honourable Ginette Petitpas Taylor, P.C., M.P.
Minister of Health
House of Commons
Ottawa, Ontario K1A 0K9

Dear Minister:

Like all Canadians, BGP Pharma ULC / Mylan Pharmaceuticals ULC ("Mylan") is deeply concerned about the opioid epidemic and for those affected by it. While Mylan plays a limited role in the supply of opioid containing medications – accounting for less than 1% of opioid-containing products sold in Canada in 2017 by volume[1] – Mylan fully supports the Government of Canada's efforts to address the national public health crisis.

Importantly, Mylan does not promote or advertise its opioid-containing products in Canada to healthcare professionals and intends to continue to refrain from such activities. Despite its limited role, Mylan is committed to doing its part to help in the fight against opioid addiction, abuse and misuse. We look forward to working with federal, provincial and territorial governments, as well as key stakeholders, to ensure a comprehensive response to this crisis.

Should you or your officials have any questions regarding any information contained in this letter, please do not hesitate to contact me.

Mylan remains committed to working with key stakeholders to continue to be a part of the long-term solution for this national health issue.

Sincerely,

[redacted]

for Barry Hayter
Country Manager
BGP Pharma ULC
Mylan Pharmaceuticals ULC

---

[1] IQVIA, Doses MAT December 2017.

abbvie

July 26, 2018

The Honourable Ginette Petitpas Taylor, PC, MP
Minister of Health
House of Commons
Ottawa, Ontario
Canada, K1A 0A6

SUBJECT: **Minister of Health Letter on Opioid Crisis dated June 19, 2018**
Response to Minister of Health Letter

Dear Minister Petitpas Taylor,

This letter is to confirm receipt of your letter addressed to Stéphane Lassignardie, General Manager, AbbVie, dated June 19, 2018 concerning the notice of Intent to Restrict the Marketing and Advertising of Opioids in Canada.

AbbVie is pleased that action will be taken on this important issue that has impacted so many Canadians. It is only when we come together as a full health care community that we can find innovative solutions like this.

AbbVie endorses the position taken by Innovative Medicine's Canada (IMC) on this issue, per the letter dated July 16, 2018 herein attached.

This letter confirms that at this time AbbVie Corporation does not market any opioid products in Canada. In addition, AbbVie does not currently have any opioid-products under development. Rather, AbbVie is developing innovative products that could be deemed as "opioid-sparing".

Corporation AbbVie / AbbVie Corporation
C.P. / P.O. Box 120 Pointe-Claire / Dorval
Pointe-Claire (QC) H9R 4N5
Tél. / Tel.: 514-906-9700
Téléc. / Fax: 514-906-9701
abbvie.ca

abbvie

Trusting you will find this information satisfactory. Should you need any additional information or clarifications, please contact me at [redacted] or via email at [redacted]@abbvie.com.

Sincerely,

[redacted]

Loretta Del Bosco
Director, Regulatory Affairs, Quality Assurance, Operations

Cc: Stephane Lassignardie, General Manager, Abbvie Corporation

INNOVATIVE MEDICINES CANADA | MÉDICAMENTS NOVATEURS CANADA

July 16, 2018

Mr. Ed Morgan
Director General, Policy, Planning and International Affairs Directorate
Health Products and Food Branch, Department of Health
2005A, Ottawa, Ontario, K1A 0K9

VIA E-MAIL: hc.ppiad-dppai.sc@canada.ca

Re: Notice of Intent to Restrict the Marketing and Advertising of Opioids

Dear Mr. Morgan:

Innovative Medicines Canada (IMC) is the national voice of Canada's innovative pharmaceutical industry. We advocate for policies that enable the discovery, development and commercialization of innovative medicines and vaccines that improve the lives of all Canadians. We support our members' commitment to being valued partners in the Canadian health and regulatory system.

IMC appreciates the opportunity to submit comments on the topic of opioid advertising in Canada. We support Health Canada's objectives regarding the public health crisis involving the rapid rise in rates of drug overdoses and death involving the misuse of illicit and prescription opioids. However, we caution of the potential overreach of this initiative which could negatively impact patients, health care professionals, manufacturers and distributors, provinces and territories, as well as health and patient organizations.

The Notice of Intent describes the restriction of both *advertising* and *marketing* of opioids. Advertising is a regulated activity that involves promotion of a product, while marketing is a broader term that includes all actions associated with selling a product. The proposal itself is limited to restricting *advertising*. For clarity, descriptions of this initiative should not include the term *marketing*, given the potential to encompass many other activities that should be outside the scope of this initiative.

Second, manufacturers should continue to be permitted to advertise products or features that reduce or prevent risks or harms associated with opioid abuse or misuse. The common and effective treatment of opioid use disorders is opioid agonist therapy such as buprenorphine or methadone. Further, products with abuse-deterrent or tamper-resistant properties may also reduce risks of misuse and abuse of opioids. The advertisement of such solutions is in the interest of Canadian public health authorities.

Our members invest in discovering new medicines and technologies. We believe that access to novel medicines provides significant benefits to Canada and Canadians. Pharmaceutical innovation can play a significant part of the solution to the opioid crisis, and should be part of any

policy to reduce prescription drug abuse. IMC believes that policies that encourage the development and adoption of abuse-deterrent products, opioid agonist therapies or other relevant technologies can be expected to encourage companies to bring other medicines to market in Canada. It is important for the manufacturers and distributors to continue the advertising of such products to ensure heath care professionals, patients, health and patient organizations as well as provinces and territories are aware of their benefits.

IMC acknowledges and welcomes the statement by the Minister of Health Ginette Petitpas Taylor in the News Release published on June 19, 2018 that referenced the "possibility of carefully tailored exemptions to allow the continued sharing of helpful education and scientific information". We hope that those exemptions will include opioids that reduce or prevent risks or harms associated with opioid abuse or misuse.

Furthermore, the Notice of Intent requests information and comments on the risk, benefit and impact of regular mandatory reporting by opioid manufacturers, to Health Canada and the public, of their marketing and advertising activities, objectives and budgets. Regular mandatory reporting is burdensome on biopharmaceutical companies and will add to the many other reporting requirements the industry must satisfy. In addition, through the pre-clearance of opioid-related materials included in the newly adopted Regulations on May 2, 2018, Health Canada would have received and pre-cleared those advertisements, which makes the mandatory regular reporting of such advertising activities redundant. Finally, while pre-clearing the advertising of opioids has already been included in the Regulations, requesting mandatory reporting of the objectives and budgets of the marketing and advertising activities is beyond the scope of the Regulations and confidential for each manufacturer.

While IMC supports the goals of the restriction of opioids advertising provided the exemptions referenced above are considered, IMC is of the view that the restriction of advertising activities should not go beyond this class of medicines. The various legislations and the guidance documents in place, the existence of the advertising preclearance agencies, as well as the specific codes of conduct that apply to member companies of various industry organizations provide sufficient safeguards to ensure patients are not subjected to "false, misleading and deceptive" advertisements. In addition, the value in the biopharmaceutical industry conveying education and scientific information about health products has been acknowledged by Health Canada and should not be undermined.

Lastly, IMC would like to briefly address the definition of "advertising" and examples of "marketing practices" referenced in the Notice of Intent. Advertising is defined under the *Food and Drug Act* as "any representation by any means for the purpose of promoting, directly and indirectly, the sale of any drug or device". The Notice of Intent provides examples of marketing practices including "...reimbursement for travel and hospitality expenses to attend-industry sponsored events, and gifts of meals, equipment...". While we acknowledge that there is a potential opportunity to provide comments on the definition of "advertising" given the upcoming consultation on the "Distinction between Advertising and Other Activities" policy, IMC maintains that such referenced examples cannot be considered advertising, are already incorporated and

managed under the IMC's Code of Ethical Practices, and therefore there is no need for additional Health Canada enforcement with respect to these matters.

IMC thanks Health Canada for the opportunity to submit these concerns, and would welcome the opportunity to further elaborate on these issues or answer any questions upon request.

Sincerely,

[redacted]

Declan Hamill
Vice President, Legal, Regulatory Affairs & Compliance

Petitpas Taylor, Minister / Ministre Ginette (HC/SC)

**From:**	Petitpas Taylor, Ginette - Riding 2 <[redacted]@parl.gc.ca> on behalf of Petitpas Taylor, Ginette - M.P. <Ginette.PetitpasTaylor@parl.gc.ca>
**Sent:**	2018-07-31 12:03 PM
**To:**	Petitpas Taylor, Minister / Ministre Ginette (HC/SC)
**Subject:**	FW: Allergan's Response to Minister's Jun 19 Letter re: Canada's Collective Response to Opioid Crisis
**Attachments:**	Petitpas Taylor Jul2018.pdf

From: Cho_Martin [mailto:[redacted]@Allergan.com]
Sent: July-31-18 12:04 PM
To: Petitpas Taylor, Ginette - M.P.
Cc: Ventin_Arima
Subject: Allergan's Response to Minister's Jun 19 Letter re: Canada's Collective Response to Opioid Crisis

Dear Honorable G. Petitpas Taylor

Please see attached Allergan's letter in response to your request for a collective response to the opioid crisis (hard copy mailed). We would be pleased to meet with you and your staff to provide an overview of Allergan and our portfolio of innovative products (pharmaceuticals, devices, biologic, surgical and regenerative medicines) and to discuss how we can collaboratively work together to help with the government's response to the Canadian opioid crisis.

If you have any questions, please contact me at the email/phone number below. I look forward to meeting you and your staff in the near future.

Thanks,
Martin Cho

ECD Health Canada
DCHG Santé Canada
Rec'd Reçu JUL 31 2018
18-004653-102

Martin Cho
Director, Market Access
Allergan Inc
500 - 85 Enterprise Blvd.
Markham, ON L6G 0B5
Office (905) 940-7152
Cell [redacted]
Fax (905) 940-1902
[redacted]@allergan.com
www.allergan.ca
Allergan

Please consider the environment before printing this email.

This e-mail, including any attachments, is meant only for the intended recipient of the transmission, and may be a confidential or privileged communication. If you received this e-mail in error, any review, use, dissemination,

distribution, or copying of this e-mail is strictly prohibited. Please notify us immediately of the error by return e-mail and please delete this message from your system. Thank you in advance for your cooperation.

Allergan

85 Enterprise Blvd, Suite 500, Markham, Ontario L6G 0B5 Telephone: (905) 940-1660 Facsimile: (905) 940-1902

July 31, 2018

The Honorable Ginette Petitpas Taylor, P.C., M.P.
Minister of Health
Brooke Claxton Building, Tunney's Pasture
Postal Locator: 0906C
Ottawa, ON K1A 0K9

Dear Honorable G. Petitpas Taylor

Re: Canada's Collective Response to the Opioid Crisis

On behalf of Allergan Inc., I am writing to respond to your letter dated June 19, 2018 seeking confirmation in support of Canada's collective response to the opioid crisis.

Allergan Inc. is a global pharmaceutical company focused on developing, manufacturing and commercializing branded pharmaceutical, device, biologic, surgical and regenerative medicine products for patients.

On behalf of Allergan in Canada, I can confirm that we do not sell or market any opioid containing products in Canada and we are supportive of the Canadian government's efforts to limit the harms of opioid medications. In that regard, Allergan markets drugs in several therapeutic areas where there is currently an unmet need for non-opioid options. As you know, physicians and patients are resorting to opioids to treat their non-cancer pain as other Health Canada approved options are not available due to funding decisions at the provincial level.

Allergan would be pleased to meet with you and your staff to introduce Allergan and discuss how we can collaboratively work together to help with the government's response to the Canadian opioid crisis.

I will follow up with your office shortly to seek a meeting. In the meantime, please contact me at [redacted] or at [redacted]@allergan.com if you have any questions or require additional information.

Kind Regards,

[redacted]

Arima Ventin, Executive Director
Market Access, Pricing, Government and Trade Relations
Allergan Inc.

Dear company,

The Government of Canada is deeply concerned about the opioid crisis which is affecting Canadians across all regions and from all segments of society. Although there are many factors that have led to this situation, the high level of opioids historically prescribed in Canada has certainly contributed to what is now a national public health crisis.

For this reason, on June 19, 2018, the Minister of Health called on industry to demonstrate leadership by immediately suspending the marketing and advertising of opioids. As part of this call out, a letter was sent to [company x]. As this request extends to all affiliates of this company, I have attached a copy of the letter for your reference and response.

I am writing to you today to confirm your support of Canada's collective response to the opioid crisis by immediately suspending any and all marketing and advertising of opioids to healthcare professionals.

As you know, the Government of Canada is committed to openness and transparency. As such, Health Canada will publish this letter and any response received. The Department looks forward to publicly recognizing those pharmaceutical companies that respond to this call to action.

I look forward to your response.

Sincerely,

Ed Morgan

Chère compagnie,

Le gouvernement du Canada est profondément préoccupé par la crise des opioïdes qui touche les Canadiennes et les Canadiens de toutes les régions et de tous les segments de la société. Bien que de nombreux facteurs aient mené à cette situation, le nombre historiquement élevé d'opioïdes prescrits au Canada a certainement contribué à ce qui est maintenant une crise nationale de santé publique.

C'est pourquoi, le 19 juin 2018, la ministre de la Santé a appelé l'industrie à faire preuve de leadership en suspendant immédiatement le marketing et la publicité sur les opioïdes. Dans le cadre de cet appel, une lettre a été envoyée à [companie x]. Comme cette demande s'adresse à toutes les entreprises affiliées à cette société, j'ai joint une copie de la lettre aux fins de consultation et de réponse.

Je vous écris aujourd'hui pour solliciter votre engagement à l'appui de la réponse collective du Canada à la crise des opioïdes en suspendant immédiatement toute commercialisation et toute publicité d'opioïdes auprès des professionnels de la santé.

Comme vous le savez, le gouvernement du Canada est déterminé à faire preuve d'ouverture et de transparence. À ce titre, Santé Canada publiera la présente lettre et toutes les réponses reçues. Le Ministère est impatient de reconnaître publiquement les sociétés pharmaceutiques qui répondent à cet appel à l'action.

J'attends avec impatience votre réponse.

Mes sincères salutations,

Ed Morgan

Dear company,

The Government of Canada is deeply concerned about the opioid crisis which is affecting Canadians across all regions and from all segments of society. Although there are many factors that have led to this situation, the high level of opioids historically prescribed in Canada has certainly contributed to what is now a national public health crisis.

For this reason, on June 19, 2018, the Minister of Health called on industry to demonstrate leadership by immediately suspending the marketing and advertising of opioids. As part of this call out, a letter was sent to [company X]. As this request extends to all affiliates of this company, I have attached a copy of the letter for your reference and response.

It is our understanding that you do not currently distribute opioids; however, should you do so in the future, we would seek your commitment to suspend any and all marketing and advertising of opioids to healthcare professionals in support of Canada's collective response to the opioid crisis.

As you know, the Government of Canada is committed to openness and transparency. As such, Health Canada will publish this letter and any response received. The Department looks forward to publicly recognizing those pharmaceutical companies that respond to this call to action.

I look forward to your response.

Sincerely,

Ed Morgan

Chère compagnie,

Le gouvernement du Canada est profondément préoccupé par la crise des opioïdes qui touche les Canadiennes et les Canadiens de toutes les régions et de tous les segments de la société. Bien que de nombreux facteurs aient mené à cette situation, le nombre historiquement élevé d'opioïdes prescrits au Canada a certainement contribué à ce qui est maintenant une crise nationale de santé publique.

C'est pourquoi, le 19 juin 2018, la ministre de la Santé a appelé l'industrie à faire preuve de leadership en suspendant immédiatement le marketing et la publicité sur les opioïdes. Dans le cadre de cet appel, une lettre a été envoyée à [compagnie x]. Comme cette demande s'adresse à toutes les entreprises affiliées à cette société, j'ai joint une copie de la lettre aux fins de consultation et de réponse.

Selon nos renseignements, vous ne distribuez pas d'opioïdes actuellement. Cependant, si ce fait venait à changer par la suite, nous vous demanderions de vous engager à interrompre tout marketing et toute publicité sur les opioïdes auprès des professionnels de la santé en appui à la réponse collective du Canada à la crise des opioïdes.

Comme vous le savez, le gouvernement du Canada est déterminé à faire preuve d'ouverture et de transparence. À ce titre, Santé Canada publiera la présente lettre et toutes les réponses reçues. Le Ministère est impatient de reconnaître publiquement les sociétés pharmaceutiques qui répondent à cet appel à l'action.

J'attends avec impatience votre réponse.

Mes sincères salutations,

Ed Morgan

Dear company,

The Government of Canada is deeply concerned about the opioid crisis which is affecting Canadians across all regions and from all segments of society. Although there are many factors that have led to this situation, the high level of opioids historically prescribed in Canada has certainly contributed to what is now a national public health crisis.

For this reason, on June 19, 2018, the Minister of Health called on industry to demonstrate leadership by immediately suspending the marketing and advertising of opioids. I am writing you today to confirm your support of Canada's collective response to the opioid crisis by immediately suspending any and all marketing and advertising of opioids to healthcare professionals.

As you know, the Government of Canada is committed to openness and transparency. As such, Health Canada will publish this letter and all responses received. The Department looks forward to publicly recognizing those pharmaceutical companies that respond to this call to action.

I look forward to your response.

Sincerely,

Ed Morgan

Chère compagnie,

Le gouvernement du Canada est profondément préoccupé par la crise des opioïdes qui touche les Canadiennes et les Canadiens de toutes les régions et de tous les segments de la société. Bien que de nombreux facteurs aient mené à cette situation, le nombre historiquement élevé d'opioïdes prescrits au Canada a certainement contribué à ce qui est maintenant une crise nationale de santé publique.

C'est pourquoi, le 19 juin 2018, la ministre de la Santé a appelé l'industrie à faire preuve de leadership en suspendant immédiatement le marketing et la publicité sur les opioïdes. Je vous écris aujourd'hui pour obtenir la confirmation que vous appuyez la réponse collective du Canada à la crise des opioïdes et suspendrez immédiatement tout marketing et toute publicité sur les opioïdes auprès des professionnels de la santé.

Comme vous le savez, le gouvernement du Canada est déterminé à faire preuve d'ouverture et de transparence. À ce titre, Santé Canada publiera la présente lettre et toutes les réponses reçues. Le Ministère est impatient de reconnaître publiquement les sociétés pharmaceutiques qui répondent à cet appel à l'action.

J'attends avec impatience votre réponse.

Mes sincères salutations,

Ed Morgan

Recipients of Minister of Health's Letter June 19, 2018	Destinataires de la lettre de la Ministre de la santé le 19 juin 2018
1. AA Pharma Inc.<br>2. AbbVie Corporation<br>3. Accord Healthcare Inc.<br>4. Adapt Pharma Canada Limited<br>5. Allergan Inc.<br>6. Apotex Inc.<br>7. Aralez Pharmaceuticals Canada Inc.<br>8. Aspen Pharmacare Canada Inc.<br>9. Astrazeneca Canada Inc.<br>10. Auro Pharma Inc.<br>11. Baxter Corporation<br>12. Biogen Canada Inc.<br>13. Biomed Pharma<br>14. Biosyent Pharma Inc.<br>15. Boehringer Ingelheim (Canada) Limited<br>16. Bristol-Myers Squibb Canada<br>17. Buzzz Pharmaceuticals Ltd. (Dublin)<br>18. Canada's Medical Device Technology Companies (Medec)<br>19. Canadian Generic Pharmaceuticals Association<br>20. Church & Dwight Canada Corporation<br>21. Cipher Pharmaceuticals Inc.<br>22. Collegium Pharmaceutical, Inc.<br>23. Dentsply Canada Limited<br>24. DRA Pharmedev Canada Inc.<br>25. Eli Lilly Canada Inc.<br>26. EMD Serono, A Division Of EMD Inc., Canada<br>27. Endo Ventures Inc.<br>28. ERFA Canada 2012 Inc.<br>29. Eisai Limited<br>30. Ethypharm<br>31. Euro-Pharm International Canada Inc.<br>32. Fresenius-Kabi Canada Limited<br>33. Generic Medical Partners Inc.<br>34. GlaxoSmithKline Inc.<br>35. Groupement Provincial de l'industrie du médicament (GPIM)<br>36. GW Pharmaceuticals<br>37. Hansamed Limited<br>38. HLS Therapeutics Inc.<br>39. Hoffmann-La Roche Limited<br>40. INDIVIOR Canada Ltd<br>41. Innovative Medicines Canada<br>42. Intrinsik Corp.	1. AA Pharma Inc.<br>2. Accord Healthcare Inc.<br>3. Adapt Pharma Canada Limited<br>4. Allergan Inc.<br>5. Apotex Inc.<br>6. Aralez Pharmaceuticals Canada Inc.<br>7. Aspen Pharmacare Canada Inc.<br>8. Association canadienne du médicament générique<br>9. AstraZeneca Canada Inc.<br>10. Auro Pharma Inc.<br>11. Biogen Canada Inc.<br>12. Biomed Pharma<br>13. BioSyent Pharma Inc.<br>14. Boehringer Ingelheim (Canada) Limitée<br>15. Bristol-Myers Squibb Canada<br>16. Buzzz Pharmaceuticals Ltd. (Dublin)<br>17. Church & Dwight Canada Corporation<br>18. Cipher Pharmaceuticals Inc.<br>19. Collegium Pharmaceutical, Inc.<br>20. Corporation AbbVie<br>21. Dentsply Canada Limited<br>22. DRA Pharmedev Canada Inc.<br>23. Eisai Co. Limited<br>24. Eli Lilly Canada Inc.<br>25. Emd Serono, une division d'EMD Canada Inc.<br>26. Endo Ventures Inc.<br>27. ERFA Canada 2012 Inc.<br>28. Ethypharm<br>29. EURO-PHARM International Canada inc.<br>30. Fresenius-Kabi Canada Limitée<br>31. Generic Medical Partners Inc.<br>32. GlaxoSmithKline Inc.<br>33. Groupement Provincial de l'industrie du médicament (GPIM)<br>34. GW Pharmaceuticals<br>35. HANSAmed Limited<br>36. HLS Therapeutics Inc.<br>37. Hoffmann-La Roche Limitée<br>38. INDIVIOR Canada Ltd.<br>39. Intrinsik Corp.<br>40. Ionis Pharmaceuticals, Inc.<br>41. JAMP Pharma Corporation<br>42. Janssen Inc.<br>43. Knight Therapeutics Inc.

43. Ionis Pharmaceuticals, Inc.<br>44. JAMP Pharma Corporation<br>45. Janssen Inc.<br>46. Knight Therapeutics Inc.<br>47. Laboratoire Atlas<br>48. Laboratoire Riva Inc.<br>49. Lundbeck Canada Inc.<br>50. Lupin Pharma Canada<br>51. Mallinckrodt Canada ULC<br>52. Mantra Pharma Inc.<br>53. Mapi Group<br>54. Marcan Pharmaceuticals Inc.<br>55. Merck Canada Inc.<br>56. Mint Pharmaceuticals Inc.<br>57. Mitsubishi Tanabe Pharmaceutical Development America, Inc.<br>58. Mylan Pharmaceuticals ULC (BGP Pharma ULC)<br>59. Novartis Pharmaceuticals Canada Inc.<br>60. Odan Laboratories Ltd.<br>61. Omega Laboratories Ltd.<br>62. Otsuka Canada Pharmaceutical Inc.<br>63. Paladin Labs Inc.<br>64. Peptigroupe Inc.<br>65. Phebra Pty Ltd<br>66. Pfizer Canada Inc.<br>67. Pharmapar Inc.<br>68. Pharmascience Inc.<br>69. Pharmazeutische Fabrik Montavit Ges M.B.H.<br>70. Pro Doc Ltée<br>71. Purdue Pharma<br>72. Ranbaxy Pharmaceuticals Canada Inc. a SUN PHARMA Company<br>73. Regulatory Solutions Inc.<br>74. Sandoz Canada Inc.<br>75. Sanis Health Inc.<br>76. Sanofi-Aventis Canada Inc.<br>77. Shire Pharma Canada ULC<br>78. Sivem Pharmaceuticals ULC<br>79. Sterimax Inc.<br>80. Sterinova Inc.<br>81. Sunovion Pharmaceuticals Canada Inc.<br>82. Taro Pharmaceuticals Inc.<br>83. Teligent<br>84. Teva Canada Limited.<br>85. Therapeutic Products, Inc.<br>86. UCB Canada Inc.	44. Laboratoire Atlas<br>45. Laboratoire Riva Inc.<br>46. Lundbeck Canada Inc.<br>47. Lupin Pharma Canada<br>48. Mallinckrodt Canada ULC<br>49. Mantra Pharma Inc.<br>50. Mapi Group<br>51. Marcan Pharmaceuticals Inc.<br>52. Médicaments novateurs Canada<br>53. Merck Canada Inc.<br>54. Mint Pharmaceuticals Inc.<br>55. Mitsubishi Tanabe Pharmaceutical Development America, Inc.<br>56. Mylan Pharmaceuticals ULC (BGP Pharma ULC)<br>57. Novartis Pharmaceuticals Canada Inc.<br>58. Odan Laboratories Ltd.<br>59. Omega Laboratories Ltd.<br>60. Otsuka Canada Pharmaceutical Inc.<br>61. Paladin Labs Inc.<br>62. Peptigroupe Inc.<br>63. Pfizer Canada Inc.<br>64. Pharmapar Inc.<br>65. Pharmascience Inc.<br>66. Pharmazeutische Fabrik Montavit Ges M.B.H.<br>67. Phebra Pty Ltd<br>68. Pro Doc Ltée<br>69. Purdue Pharma<br>70. Ranbaxy Pharmaceuticals Canada Inc., un compagnie de SUN PHARMA<br>71. Regulatory Solutions Inc.<br>72. Sandoz Canada Inc.<br>73. Sanis Health Inc. (membre du réseau Pharmaprix)<br>74. Sanofi-Aventis Canada Inc.<br>75. Shire Pharma Canada ULC<br>76. Sivem Pharmaceuticals ULC<br>77. Société Baxter<br>78. Les sociétés canadiennes de technologies médicales (MEDEC)<br>79. SteriMax Inc.<br>80. Sterinova Inc.<br>81. Sunovion Pharmaceuticals Canada Inc.<br>82. Taro Pharmaceuticals Inc.<br>83. Teligent<br>84. Teva Canada Limitée<br>85. Therapeutic Products Inc.

87. Valeant Canada LP<br>88. Xediton Pharmaceuticals Inc.	86. UCB Canada Inc.<br>87. Valeant Canada LP<br>88. Xediton Pharmaceuticals Inc.
**Recipients of Health Canada's Letter August 17, 2018**	**Destinataires de la lettre de Santé Canada le 17 août 2018**
Version A	Version A
1. Actavis Pharma Company<br>2. Pendopharm a Divisions of de Pharmascience Inc.<br>3. Pfizer Consumer Healthcare a Division of Pfizer Canada Inc.	1. Actavis Pharma Company<br>2. Pendopharm, une division de Pharmascience Inc.<br>3. Pfizer Consumer Healthcare, une division de Pfizer Canada Inc
Version B	Version B
1. Actavis Group PTC ehf.<br>2. Bard Pharmaceuticals (1990) Inc.<br>3. Hospira Healthcare Corporation<br>4. Confab Laboratories Inc.<br>5. Laboratoires Trianon Inc.<br>6. Patriot a Division of Janssen Inc.<br>7. Tanta Pharmaceuticals Inc<br>8. Viva Pharmaceutical Inc.	1. Actavis Group PTC ehf.<br>2. Bard Pharmaceuticals (1990) Inc.<br>3. Corporation Hospira Healthcare<br>4. Laboratoires Confab Inc.<br>5. Laboratoires Trianon Inc.<br>6. Patriot, une division de Janssen Inc<br>7. Tanta Pharmaceuticals Inc.<br>8. Viva Pharmaceuticals Inc.
Version C	Version C
1. ICU Medical Canada<br>2. Pharmetics (2011) Inc.<br>3. Vita Health Products Inc.	1. ICU Medical Canada<br>2. Pharmetics (2011) Inc.<br>3. Vita Health Products Inc.

Laboratoires
Confab
Laboratories inc.

M. Morgan,

Les Laboratoires Confab sont aux faits et aussi préoccupés par la crise des opioïdes qui touche les Canadiennes et les Canadiens de toutes les régions et de tous les segments de la société.

Nous sommes aussi au courant et supportons la décision du ministre de la Santé qui a suspendu le marketing et la publicité sur les opioïdes.

Vos renseignements sont à jours, Confab ne distribue pas des opioïdes actuellement. Nous nous engageons aussi à ne pas effectuer de campagne de marketing ni de publicité sur les opioïdes auprès des professionnels de la santé

Notez aussi que nous n'avons aucun projet en développement pour ce type de produit.

J'espère que ceci répond à vos questions.

Je vous prie d'agréer, Monsieur, l'expression de mes sentiments les meilleurs.

[redacted] 2018-08-20.

Lucie Tremblay
Executive Director, Quality Operations
Confab Laboratories Inc.

VITA
HEALTH PRODUCTS

150 Beghin Avenue
Winnipeg, Manitoba
R2J 3W2
Tel: 204/661-8386
Fax: 204/663-8386

September 4th, 2018

Ed Morgan
Director General
Policy, Planning and International Affairs Directorate
Health Canada

Dear Mr Morgan,

This letter is in response to the request I received from you by email on August 17th concerning the marketing and advertising of opioids in Canada.

Vita Health Products Inc. manufactures and distributes a small number of products containing 8 mg codeine phosphate that are exempted from prescription control under Section 36 of the Narcotic Control Regulations. We do not manufacture or distribute any other opiate-containing products.

I can confirm that Vita Health Products Inc. does not engage in the marketing or advertising of opioid products (either directly or indirectly) to health professionals.

Sincerely,

[redacted]

Hilary M Lloyd MRPharmS
Director of Regulatory Affairs
Vita Health Products Inc.

Copy:
Rachel Cahill, Senior Vice President, Finance, Vita Health Products Inc.
Carlson Teakle, Vice President, Sales and Marketing, Vita Health Products Inc.
Stephanie Haverstick, Vice President and Operating Officer, Vita Health Products Inc.

Hospira
A Pfizer Company

September 6, 2018

Ed Morgan
Director General
Policy, Planning and International Affairs Directorate
Édifice Graham Spry Building
Room/pièce 538 (AL: 2005A)
250, avenue Lanark Avenue
Ottawa, Ontario
K1A 0K9

Via email to: ed.morgan@canada.ca

Dear Mr. Morgan:

On behalf of Hospira Healthcare Corporation, a Pfizer Company, thank you for your letter of August 17th regarding the Minister of Health's intention to restrict the marketing and advertising of opioids and her request for our commitment to suspend those activities in Canada, should Hospira distribute opioids in future.

I can confirm that John Helou's letter of July 13th to Minister Petitpas-Taylor and its commitment covered the activities of all Pfizer businesses units, including Hospira Healthcare Corporation.

I trust that you will find this reply to your satisfaction.

Yours sincerely,

[redacted]

Beryl Chan, Director Regulatory Affairs
Hospira Healthcare Corporation, a Pfizer Company

C.C.:
John Helou, President, Pfizer Canada Inc.
Dr. Vratislav Hadrava, MD, PhD, Country Medical Director & IM Medical Affairs Lead, Pfizer Canada Inc.

Michèle Kay
Director, Regulatory Affairs
Tel: 905 507 7081
Cell: [redacted]
Fax: 905 507 7115
Email: [redacted]@pfizer.com

Pfizer Consumer Healthcare
450 - 55 Standish Court
Mississauga, Ontario L5R 4B2
Tel: 905 507 7000

September 6, 2018

Ed Morgan
Director General
Policy, Planning and International Affairs Directorate
Édifice Graham Spry Building
Room/pièce 538 (AL: 2005A)
250, avenue Lanark Avenue
Ottawa, Ontario
K1A 0K9

Via email to: ed.morgan@canada.ca

Dear Mr. Morgan:

On behalf of Pfizer Consumer Healthcare, a division of Pfizer Canada Inc., thank you for your letter of August 17th regarding the Minister of Health's intention to restrict the marketing and advertising of opioids and her request for our commitment to suspend those activities in Canada.

I can confirm that John Helou's letter of July 13th to Minister Petitpas-Taylor and its commitment covered the activities of all Pfizer business units, including Pfizer Consumer Healthcare.

I trust that you will find this reply to your satisfaction.

Yours sincerely,

[redacted]

Michèle Kay, Director Regulatory Affairs
Pfizer Consumer Healthcare, a division of Pfizer Canada Inc.

CC.:
John Helou, President, Pfizer Canada Inc.
Dr. Vratislav Hadrava, MD, PhD, Country Medical Director & IM Medical Affairs Lead, Pfizer Canada Inc.

teva

September 14, 2018

Ed Morgan
Director General
Policy, Planning and International Affairs Directorate
Health Products and Food Branch
Ottawa, Canada
K1A 0K9

Dear Mr. Morgan,

It was a great pleasure for our General Manager to meet with the Minister of Health in April as part of a delegation from the Canadian Generic Pharmaceutical Association (CGPA) to talk about opportunities to work with your government to improve the health of Canadians. Subsequent to integration with Actavis, Teva is one of Canada's largest pharmaceutical companies making innovative and generic medicines available to Canadian patients and we are proud to be part of this important industry in Canada.

Our GM has recently received a letter concerning the Health Ministry's intent to limit the marketing and advertising of opioids to health care professionals. We were specifically asked for industry to show leadership by suspending any marketing or advertising of opioids. On behalf of Teva, our GM has written a letter on June 26, 2018.

In response to e-mail I have received on August 17th, I am pleased to inform you that Teva/Actavis does not promote or advertise any opioid products to prescribers and we support the government's efforts to restrict the marketing of prescription opioids to physicians.

Our security and quality control processes are extremely vigilant and we support our supply chain partners in programs aimed at tackling diversion of opioids. For example, Teva's "Patch-for-Patch" pharmacy program ensures that patients can receive a refill only when a used patch is returned or can safely dispose old patches.

One of the often cited causes of the public health issues around opioid use is over-prescribing. It is important to point out that education on the appropriate prescribing of opioids is critical. We support efforts to educate physicians on appropriate prescribing and patient education about opioid use and the safe storage of all medicines. As members of the CGPA, we are aligned in encouraging Health Canada to focus on a strategy that involves physician and patient education.

We appreciate the efforts Minister of Health is taking to combat this public health crisis and find solutions to avoid further tragedy. We are committed to working with you to make sure that Canadians who live with pain have access to these important medicines while also ensuring that these products are not abused or misused.

Please let me know if there is any more we can do to help. Please note that Actavis Pharma Company has been now completely amalgamated with Teva Canada Limited.

Sincerely,

[redacted]

Mathi Mathivanan
Senior Director, Regulatory Affairs
Teva Canada Limited

Bristol-Myers Squibb Canada

2344, Alfred-Nobel, Bureau 300, Saint-Laurent (Québec) H4S 0A4
Tél.: 514-333-3200 Fax: 514-335-4102

Hon. Ginette Petitpas Taylor, P.C., M.P.
Minister of Health
Health Canada
Brooke Claxton Building, Tunney's Pasture
Ottawa ON
K1A 0K9

September 17th, 2018

Dear Minister,

We received your letter dated June 19th, 2018 that seeks our commitment to support Canada's collective response to the opioid crisis by immediately suspending all marketing and advertising of opioids to health care professionals.

Bristol Myers Squibb Canada does not currently manufacture, distribute, or sell opioids as part of its product portfolio. In the past, BMS did sell opioids, however, these were never marketed or promoted. We commend you on taking the necessary steps via public consultations to address this national crisis.

Sincerely,

[redacted]

Alan Reba
General Manager
Bristol-Myers Squibb Canada

pharma science

September 21, 2018

Mr. Ed Morgan, Director General
Policy, Planning and International Affairs Directorate
Graham Spry Building, Room 538 (AL: 2005A)
250, Lanark Avenue
Ottawa, Ontario, K1A OK9

RE: Advertising and Marketing of Opioids
Letters of June 19, 2018 and August 17, 2018

Dear Mr. Morgan,

Pharmascience Inc, hereby acknowledges receipt of the letters from the Minister of Health and yourself on June 19, 2018 and August 17, 2018, respectively. Pharmascience is a member of the Canadian Generic Manufacturer's Association (CGPA) and we make reference to, and endorse the content of, the CGPA response to the Minister's letter dated July 17, 2018.

As one of the few wholly-owned Canadian pharmaceutical companies, Pharmascience is deeply concerned with the ongoing crisis and have undertaken and/or are participating in several initiatives to combat the misuse of these products and to respond to unmet medical needs.

Pharmascience's generic pharmaceuticals are sold directly to pharmacies or wholesalers and we do not engage in any role whatsoever in the decision made by a physician or other health care professional in prescribing an opioid to a patient. Once the patient arrives at the pharmacy with a valid prescription, the pharmacist will decide on which product to dispense based on the requirements of the provincial governments and/or private payers.

Notwithstanding the above, we would like to reiterate the legitimate need for ongoing communication with healthcare professionals to inform them of new innovations in the treatment of pain and/or addiction (e.g., new products/formulations), new safety measures (e.g., Risk Management Plans) and any changes to risk/benefit profiles for existing products.

Pharmascience supports the efforts by the federal and provincial governments to curtail this urgent public health crisis and are committed to continue our collaboration with authorities to implement meaningful measures to address the medical needs of all Canadians.

Should there be any further information needed, please address these to Ms. Deirdre Cozier, Director, Global Regulatory Affairs.

Sincerely,

[redacted]

David W. Goodman, Ph.D.
Chief Executive Officer
Pharmascience Inc.

PHARMASCIENCE INC.
Head Office - 514.340.9800 - pharmascience.com
6111 Royalmount Avenue, Montreal, Quebec H4P 2T4

pharma science

September 21, 2018

Mr. Ed Morgan, Director General
Policy, Planning and International Affairs Directorate
Graham Spry Building, Room 538 (AL: 2005A)
250, Lanark Avenue
Ottawa, Ontario, K1A OK9

RE: Advertising and Marketing of Opioids
Letters of June 19' 2018 and August 17, 2018

Dear Mr. Morgan,

Pharmascience Inc, hereby acknowledges receipt of the letters from the Minister of Health and yourself on June 19, 2018 and August 17, 2018, respectively. Pharmascience is a member of the Canadian Generic Manufacturer's Association (CGPA) and we make reference to, and endorse the content of, the CGPA response to the Minister's letter dated July 17, 2018.

As one of the few wholly-owned Canadian pharmaceutical companies, Pharmascience is deeply concerned with the ongoing crisis and have undertaken and/or are participating in several initiatives to combat the misuse of these products and to respond to unmet medical needs.

Pharmascience's generic pharmaceuticals are sold directly to pharmacies or wholesalers and we do not engage in any role whatsoever in the decision made by a physician or other health care professional in prescribing an opioid to a patient. Once the patient arrives at the pharmacy with a valid prescription, the pharmacist will decide on which product to dispense based on the requirements of the provincial governments and/or private payers.

Notwithstanding the above, we would like to reiterate the legitimate need for ongoing communication with healthcare professionals to inform them of new innovations in the treatment of pain and/or addiction (e.g., new products/formulations), new safety measures (e.g., Risk Management Plans) and any changes to risk/benefit profiles for existing products.

Pharmascience supports the efforts by the federal and provincial governments to curtail this urgent public health crisis and are committed to continue our collaboration with authorities to implement meaningful measures to address the medical needs of all Canadians.

PHARMASCIENCE INC.
Head Office - 514.340.9800 - pharmascience.com
6111 Royalmount Avenue, Montreal, Quebec H4P 2T4

Should there be any further information needed, please address these to Ms. Deirdre Cozier, Director, Global Regulatory Affairs.

Sincerely,

[redacted]

David W. Goodman, Ph.D.
Chief Executive Officer
Pharmascience Inc.

PHARMASCIENCE INC.
Head Office - 514.340.9800 - pharmascience.com
6111 Royalmount Avenue, Montreal, Quebec H4P 2T4

September 21, 2018

Mr. Ed Morgan, Director General
Policy, Planning and International Affairs Directorate
Graham Spry Building, Room 538 (AL: 2005A)
250, Lanark Avenue
Ottawa, Ontario, K1A OK9

RE: Advertising and Marketing of Opioids
Letters of June 19' 2018 and August 17, 2018

Dear Mr. Morgan,

Pharmascience Inc, hereby acknowledges receipt of the letters from the Minister of Health and yourself on June 19, 2018 and August 17, 2018, respectively. Pharmascience is a member of the Canadian Generic Manufacturer's Association (CGPA) and we make reference to, and endorse the content of, the CGPA response to the Minister's letter dated July 17, 2018.

As one of the few wholly-owned Canadian pharmaceutical companies, Pharmascience is deeply concerned with the ongoing crisis and have undertaken and/or are participating in several initiatives to combat the misuse of these products and to respond to unmet medical needs.

Pharmascience's generic pharmaceuticals are sold directly to pharmacies or wholesalers and we do not engage in any role whatsoever in the decision made by a physician or other health care professional in prescribing an opioid to a patient. Once the patient arrives at the pharmacy with a valid prescription, the pharmacist will decide on which product to dispense based on the requirements of the provincial governments and/or private payers.

Notwithstanding the above, we would like to reiterate the legitimate need for ongoing communication with healthcare professionals to inform them of new innovations in the treatment of pain and/or addiction (e.g., new products/formulations), new safety measures (e.g., Risk Management Plans) and any changes to risk/benefit profiles for existing products.

Pharmascience supports the efforts by the federal and provincial governments to curtail this urgent public health crisis and are committed to continue our collaboration with authorities to implement meaningful measures to address the medical needs of all Canadians.

PHARMASCIENCE INC.
Head Office - 514.340.9800 - pharmascience.com
6111 Royalmount Avenue, Montreal, Quebec H4P 2T4

Should there be any further information needed, please address these to Ms. Deirdre Cozier, Director, Global Regulatory Affairs.

Sincerely,

[redacted]

David W. Goodman, Ph.D.
Chief Executive Officer
Pharmascience Inc.

PHARMASCIENCE INC.
Head Office - 514.340.9800 - pharmascience.com
6111 Royalmount Avenue, Montreal, Quebec H4P 2T4

Petitpas Taylor, Minister / Ministre Ginette (HC/SC)

**From:**	Petitpas Taylor, Ginette - Riding 2 <[redacted]@parl.gc.ca> on behalf of Petitpas Taylor, Ginette - M.P. <Ginette.PetitpasTaylor@parl.gc.ca>
**Sent:**	2018-09-24 8:19 AM
**To:**	Petitpas Taylor, Minister / Ministre Ginette (HC/SC)
**Subject:**	FW: Acknowledgement of Immediate Suspension of Marketing and Advertising of Opiods
**Attachments:**	Sterimax Inc. - Suspension of Opiod Marketing - 09.21.2018.pdf

---

From: Greg Ellis [mailto:[redacted]@Sterimaxinc.com]
Sent: September-21-18 5:07 PM
To: Petitpas Taylor, Ginette - M.P.
Cc: Ritesh Acharya; Fong Chan
Subject: Acknowledgement of Immediate Suspension of Marketing and Advertising of Opiods

Dear Honourable Minister,

On behalf of SteriMax Inc., I am attaching a letter of acknowledgement in response to your call to action related to the suspension of Marketing and Advertising of Opoids in Canada. I must apologize for our tardy response to your request. It was an unacceptable oversight on our behalf.

As the attached letter indicates, SteriMax has always acted with great responsibility and discretion in the supply of product within this therapeutic area. You can be assured that we will continue to do so.

Please accept this acknowledgement and contact me if you have additional questions or concerns in this regard.

Sincerely,

Gregory Ellis
President & COO

STERIMAX INC.
2770 Portland Drive
Oakville, Ontario
L6H 6R4
Office: (289) 881-0604
Mobile: [redacted]

[redacted]@sterimaxinc.com
www.sterimaxinc.com

ECD Health Canada
DCHG Santé Canada
Rec'd Reçu SEP 26 2018
[illegible]

NOTICE: This SteriMax Inc. e-mail message (including any file attachment) is intended only for the use of the individual or entity to which it is addressed, and may contain information that is privileged and/or confidential. If you are not the intended recipient, any dissemination, distribution or copying of this communication is strictly prohibited. If you have received this communication in error, please notify the sender immediately by reply e-mail or a collect telephone call and delete or destroy all copies of this message and any file attachment.

STERI*MAX*

2770 Portland Drive, Oakville, Ontario, Canada L6H 6R4
Tel.: 905-890-0661 800-881-3550 • Fax: 905-890-0508 877-546-7667 • Web: www.sterimaxinc.com

September 21, 2018

The Honourable Ginette Petitpas Taylor, P.C., MP
Minister of Health
House of Commons
Ottawa, Ontario
K1A 0A6
By email: Ginette.PetitpasTaylor@parl.gc.ca

re: Request for immediate voluntary suspension of marketing and advertising of opioids to healthcare professionals

To the Honourable Ginette Petitpas Taylor,

This letter is in response to your letter which called upon industry to immediately suspend marketing and advertising of opioids to healthcare professionals.

As a family-owned Canadian company, Steri*Max* Inc. ("Steri*Max*") is committed to maximizing the wellness of Canadians, and therefore we fully support this effort being made by the government to address the serious opioid crisis in Canada.

Steri*Max* confirms that we do not market or advertise our opioid products to prescribing physicians, and further, we do not list our opioid products on our company website, promotional materials or conference banners.

Our company's portfolio of products, including the opioids products, consists primarily of generic pharmaceuticals that are intended for use under the direct care and supervision of a health care professional in hospitals, pain clinics and home health care settings. We are committed to continuing to provide lower cost medications to Canadian patients with legitimate health needs. At the same time, we stand committed with Health Canada to ensure the proper and safe use of opioid medications and work towards solutions to decrease opioid addiction and misuse.

Sincerely, [redacted]

Gregory Ellis
President and Chief Operating Officer
Steri*Max* Inc.

The Honourable Ginette Petitpas Taylor, P.C., M.P.
Minister of Health

September 27, 2018

Re: Notice of Intent to Restrict the Marketing and Advertising of Opioids

Dear Mrs. Petitpas Taylor,

In response to your letter received on June 19, 2018, we would like to inform you that Omega Laboratories Ltd does not manufacture, commercialize or distribute opioids products.

Sincerely, [redacted]

Bruce W. Levins
President and General Manager
Tel.: 514-335-0310 ext. [redacted]
Email: [redacted]@omegalabs.ca

L'honorable Ginette Petitpas Taylor, P.C., M.P.
Ministre de la Santé

Le 27 septembre 2018

Re: Avis d'intention de restreindre le marketing et la publicité des opioïdes

Chère Mme Petitpas Taylor,

En réponse à votre lettre reçue le 19 juin 2018, nous tenons à vous informer que les Laboratoires Omega Limitées ne fabrique, ne commercialise ni ne distribue de produits opioïdes.

Cordialement, [redacted]

Bruce W. Levins
Président et Directeur Général
Tél. : 514-335-0310 poste [redacted]
Courriel: [redacted]@omegalabs.ca

OFFICE : 11177 HAMON, MONTRÉAL, (QUÉBEC) H3M 3E4 TÉL: (514) 335-0310 FAX : (514) 339-1407
LABORATORIES : 10850 HAMON, MONTRÉAL, (QUÉBEC) H3M 3E2

Stakeholder Meeting Report
Indivior Canada
October 11, 2018

<table>
<tr><td>Date: October 11, 2018</td><td>Meeting sheet completed by:
Policy, Planning and International Affairs Directorate</td></tr>
<tr><td>Subject :
Indivior Canada meeting with Health Canada</td><td>Stakeholder attendees:
Christine Lafave, Country Director, Indivior Canada
Dominique Gilbert, Director Market Access and Governmental Affairs
Zohra Douida, Head of Regulatory Affairs, Indivior Canada
Christine Mormont, Head of Medical Affairs, Indivior Canada
Sophie Deschenes, Director of Marketing, Indivior Canada
Philip Delistoyanov, Senior Associate, 3Sixty Public Affairs
Health Canada attendees:
Ed Morgan, Director General, Policy, Planning and International Affairs Directorate
Kirsten Mattison, Director, Controlled Substances Directorate
Amanda Moir, Director, Policy, Planning and International Affairs Directorate
Alain Musende, Manager, Marketed Health Products Directorate
Lissa Murseli, Manager, Policy, Planning and International Affairs Directorate
Jessica Faubert, Senior Policy Analyst, Policy, Planning and International Affairs Directorate</td></tr>
<tr><td colspan="2">■ Privacy notice was read and acknowledged

“We would like to remind you that this meeting is subject to disclosure as per the Health Products and Food Branch Policy on Open Decision-Making. Therefore, nothing shared during this meeting is considered confidential and personal information may be disclosed without your further consent.”</td></tr>
<tr><td colspan="2">Purpose:
To discuss Indivior’s perspectives on restricting opioid marketing and advertising to health care professionals as well as the opioid crisis in Canada.

No documents were shared by HC or stakeholders</td></tr>
<tr><td colspan="2">Notes:

A summary of key points is as follows:
Indivior provided background on the company’s history and activities conducted by their sales representatives.</td></tr>
</table>

- Indivior described company actions intended to increase knowledge of opioid use disorder and treatment of opioid use disorder, such as distributing guidelines, supporting networks that facilitate knowledge transfer between clinicians, and online educational training for health care professionals. Indivior described that there is a need for this information to be shared, and that the manufacturer plays an important role in disseminating product information and training to health professionals.
- Indivior reported that restrictions to marketing and advertising of opioids could limit physicians' knowledge, abilities and comfort prescribing opioid use disorder treatment. Indivior noted that it would be important for any restrictions on marketing to consider opioid use disorder treatments as distinct from opioids used to treat pain.
- Health Canada noted educational material altered or edited by industry could become promotional in nature, and therefore should be reviewed by the Pharmaceutical Advertising Advisory Board (PAAB).
- Indivior noted past experiences with pre-clearance via PAAB, noting that there should be some flexibilty in reviewing materials that reflects an understanding of the evolving terminology used to describe opioid use disorder.
- Indivior noted it would be important that physicians be educated on access and proper utilisation of new products to treat opioid use disorder.
- Health Canada mentioned consultations on proposed restrictions are ongoing and any new rules would take time to be put in place. Health Canada acknowledged the importance of educational material in supporting health care professionals' prescribing decisions, and noted that the intent of restrictions on the marketing and advertising of opioids is not to restrict access to treatment options, including for opioid use disorder, for people who need them.

660, boul. Industriel, Blainville QC J7C 3V4
La force d'être d'ici!

Laboratoire Riva Inc.
660 Boul. Industriel
Blainville, Québec, J7C 3V4
Tél.: 450-434-7482
Fax: 450-434-7781
E-mail: [redacted]@labriva.com

ECD Health Canada
DCHG Santé Canada
Rec'd
Reçu DEC 17 2018
#18-007871-292

Office of Submissions and Intellectual Property
DEC 03 2018
Bureau des présentations et de la propriété intellectuelle

Blainville, le 29 novembre 2018

Honorable Ginette Petitpas Taylor, C.P., députée
Ministre de la Santé
Santé Canada
Ottawa, ON K1A 0K9

Objet: **Réponse à la correspondance de Santé Canada demandant la suspension du marketing et de la publicité sur les opioïdes à l'intention des professionnels de la santé**

Madame la Ministre,

Suite à votre correspondance du 19 juin 2018 demandant la suspension du marketing et de la publicité sur les opioïdes, nous souhaitons par la présente vous confirmer que Laboratoire Riva ne fait pas de marketing ni de publicité sur les opioïdes auprès des professionnels de la santé. En tant que fabriquant générique, Laboratoire Riva n'influence pas les pratiques de prescription d'opioïdes.

Laboratoire Riva s'engage à collaborer et supporter les mesures mises en place par Santé Canada dans le cadre de son champ d'activités.

Je vous prie d'agréer, Madame, l'expression de mes sentiments les meilleurs.

LABORATOIRE RIVA INC.

[redacted]

Olivier St-Denis
Président

Health Canada - Health Products and Food Branch
Bilateral Meeting Program

Agenda Item Summary – Item # 4	
**Association:** Canadian Generic Pharmaceutical Association (CGPA)	**Meeting Date: Tuesday, December 4, 2018**
**Marketing & Advertising of Opioids**<br><br>**Opioid Marketing and Advertising**<br><br>**Time Required: 20 minutes**	**Check one:**<br>☐ For decision<br>☐ For direction<br>☒ For discussion<br>☐ For information<br>☐ For update
**SECTION 1 – To be completed by organization tabling the agenda item**	

Agenda Item Sponsor: CGPA

CGPA would like to receive an update on Health Canada's proposal to restrict the marketing and advertising of opioids as per the published Notice of Intent and correspondence from the Minister of Health.

SECTION 2 – To be completed by organization responding to the agenda item

Health Canada Attendees:

Amanda Moir
Director, Strategic Horizontal Policy Division
Policy, Planning and International Affairs Directorate

Lissa Murseli
Manager, Strategic Horizontal Policy Division
Policy, Planning and International Affairs Directorate

CGPA Attendees: Duane Terrill, (Apotex, Co-chair), Sandra D'Agostino-Ferlisi (Apotex), Swamy Subramanian (Apotex), Joanne Manley (Mylan), Gaetano Gallo (Odan Laboratories), Marie-Eve Latendresse (Omega Laboratories), Deirdre Cozier (Pharmascience), Sonia Gallo (Sandoz Canada), Fong Chan (Sterimax), Ritesh Archarya (Sterimax), Lul Ogba-Ghebriel (Taro), Mathi Mathivanan (Teva), Alpa Jani (Teva), Anne Wilkie (CGPA), Jim Keon (CGPA), and Jody Cox (CGPA)

Status Update:

On June 19, 2018, the Minister of Health announced measures to address the marketing and advertising of opioids to health care professionals, including a public consultation on the Government's intention to restrict marketing and advertising to health care professionals.

Canadians were invited to submit comments on the Notice of Intent to Restrict the Marketing and Advertising of Opioids from June 19 to July 18, 2018. Over 40 submissions were received

from a range of stakeholders, including health care professionals, patients, academics, and the pharmaceutical industry.

Feedback from these consultations is informing how to proceed with further restrictions on opioid advertising and marketing. Health Canada will publish a summary of responses received on the Notice of Intent to restrict opioid marketing and advertising aimed at health care professionals.

Health Canada is also reviewing evidence on the benefits and harms of past and current marketing practices, and is continuing to consult with stakeholders and experts to inform further restrictions on opioid marketing and advertising.

Additionally, in June, the Minister called on opioid manufacturers and distributors to immediately cease opioid marketing activities to health care practitioners, on a voluntary basis. Health Canada encourages all companies that manufacture or distribute opioids in Canada to respond to this request. A summary of companies that received a letter and the correspondence received by Health Canada is available at https://www.canada.ca/en/health-canada/services/substance-use/problematic-prescription-drug-use/opioids/responding-canada-opioid-crisis/industry-response.html

Furthermore, Health Canada has created a dedicated marketing compliance and enforcement team to proactively monitor advertising and to enforce the existing rules around improper advertising.

Petitpas Taylor, Minister / Ministre Ginette (HC/SC)

**From:**	Shiam Pasupathy <[redacted]@aapharma.ca>
**Sent:**	2018-12-21 8:08 AM
**To:**	Petitpas Taylor, Minister / Ministre Ginette (HC/SC)
**Cc:**	Geoff Johnson
**Subject:**	RE: Minister of Health Letter to Pharmaceutical Industry: Marketing and Advertising of Opioids
**Attachments:**	Letter from AA Pharma.pdf

Dear Minister,

Attached is the response from AA Pharma regarding the letter we had received on June 19th 2018 about Marketing and Advertising of Opioids.

Should you have any questions please contact us.

Regards,

ECD Health Canada
DCHG Santé Canada
Rec'd Reçu JAN 10 2019
#19-000236-743

Shiam Pasupathy
General Manager
AA Pharma Inc.
1165 Creditstone Road
Unit #1
Vaughan, Ontario
L4K 4N7
Phone: [redacted]
Fax: 905-669-9567
Email: [redacted]@aapharma.ca

From: Petitpas Taylor, Minister / Ministre Ginette (HC/SC) [mailto:hcminister.ministresc@canada.ca]
Sent: June-19-18 11:18 AM
To: Shiam Pasupathy
Subject: Minister of Health Letter to Pharmaceutical Industry: Marketing and Advertising of Opioids

Please find attached correspondence from the Minister of Health Canada.

Thank you / Merci
Health Canada - Santé Canada

AA pharma

• 1165 Creditstone Road Unit #1, Vaughan ON, L4K 4N7 • Tel: 905-669-0528 • Fax: 905-669-9569

December 21, 2018

The Honourable Ginette Petitpas Taylor, P.C, M.P. Minster of Health
House of Commons Ottawa,
Ontario K1A OK9

Dear Minister:

Thank you for your letter dated June 19, 2018 requesting that the industry show leadership in helping to mitigate the effects of the current opioid crisis by immediately suspending marketing and advertising of opioids.

AA Pharma, like all Canadians, is concerned about this urgent public health crisis. AA Pharma supports the Government of Canada's efforts to address the problem of opioid abuse and looks forward to continuing to work with all stakeholders to better address this issue.

As a manufacturer of legacy pharmaceuticals, AA Pharma does not engage in the practice of advertising and / or marketing our opioids, or other narcotic products to physicians and/ or the public in Canada.

AA Pharma manufactures generic Tramadol (Ultram) and generic Butorphanol (Stado), selling them directly to Canadian Wholesalers. It is only when a patient receives an opioid prescription from their physician and brings it to the pharmacy that the pharmacist may choose to dispense a generic opioid, in place of a brand opioid. The dispensing of a generic is based on demonstrated bioequivalence, as per Health Canada's 'Declaration of Equivalence', with the brand name product. As such, AA Pharma has no role in the decision made by a physician or other health care professional in prescribing an opioid, or more specifically, AA Tramadol /or Butorphanol.

As per your request, in your June 19, 2018 letter, AA Pharma will continue our current practice of not marketing or advertising opioids to Canadian physicians and / or the public, however, AA Pharma will continue to notify pharmacists, purchasers, or payers of the availability of a generic Tramadol or Butorphanol.

In so advising these bodies, AA Pharma will provide product monographs upon request. AA Pharma does not believe that the intent of the Government in restricting the marketing and advertising of opioids is to prevent AA Pharma from advising other bodies of the products availability, it's price, or of Health Canada's declaration of bioequivalence,as specified in their respective product monographs.

(AA) pharma

• 1165 Creditstone Road Unit #1, Vaughan ON, L4K 4N7 • Tel: 905-669-0528 • Fax: 905-669-9569

As you know the Government of Canada has introduced new regulations requiring Canadian Specific Opioid targeted Risk Management Plans (RMPs) for opioid medicines. These RMPs are designed to "standardize and strengthen the rigor of the post-market surveillance of prescription opioids, allowing better quantification and characterization of the risks associated with opioid-related harms in Canadian patients. In addition, this plan will put in place targeted risk minimization activities to prevent or decrease prescription opioid-related harms in Canada".

AA Pharma will be joining the CGPA's RMP (Risk Management Plan), and both Tramadol and Butrophonal will be monitored within the plan. AA Pharma is actively involved in the planning and development of the program, and will be in compliance with the Terms and Conditions set out by Health Canada in their letter dated, September 17th, 2018.

Should you have any questions or concerns, please feel free to contact me.

Most sincerely,

[redacted]

Shiam Pasuapthy
General Manager
AA Pharma Inc.

CC: Geoff Johnson
Director, Commercial Operations
AA Pharma Inc.

From: DeSerres, Jean
Sent: 2019-02-01 1:36 PM
To: Marshall, Andrew
Cc: Morgan, Ed (HC/SC) ; Kropp, Rhonda (HC/SC)

Subject: FW: Marketing and Advertising of Opioids / Le marketing et la publicité sur les opioïdes

Hello I'm putting Andrew in the look because Ciara is no longer with Paladin and Andrew is covering for EVL.
Regards
Jean

From: DeSerres, Jean
Sent: 1 février 2019 10:08
To: 'Morgan, Ed (HC/SC)' ; Walsh, Ciara
Cc: Kropp, Rhonda (HC/SC)
Subject: RE: Marketing and Advertising of Opioids / Le marketing et la publicité sur les opioïdes

Thanks,
Anyway, we have stopped all advertisement and marketin activities with opiates.
Jean

Jean De Serres, MD, M.Sc, MBA
Vice-President Scientific Affairs and Operations/ Vice-Président Affaires Scientifiques et Opérations
Laboratoires Paladin | Paladin Labs Inc.
100 Boulevard Alexis Nihon, Bureau 600 | Montréal, QC H4M2P2
[redacted] / cell : [redacted]

Le présent courriel peut contenir des renseignements protégés et confidentiels à l'intention du destinataire indiqué ci-dessus. Si vous n'êtes pas le destinataire ou un responsable de la transmission de ce message, nous vous informons que toute divulgation, copie, distribution ou toute autre utilisation de ces informations, est interdite. Si vous avez reçu ce courriel par erreur, nous vous prions de nous en avertir immédiatement afin que Paladin puisse procéder à la juste transmission du message. Nous vous remercions aussi, d'effacer le courriel de votre système par la suite. Merci.
This e-mail transmission may contain confidential or legally privileged information that is intended only for the individual(s) or entity(ies) named in the e-mail address. If you are not the intended recipient or an agent responsible for delivering it to the intended recipient, you are hereby notified that any disclosure, copying, distribution, or reliance upon the contents of this e-mail is strictly prohibited. If you have received this e-mail transmission in error, please notify the sender immediately, so that Paladin can arrange for proper delivery, and then please delete the message from your system. Thank you.

From: Morgan, Ed (HC/SC)
Sent: 31 janvier 2019 16:10
To: Walsh, Ciara
Cc: Kropp, Rhonda (HC/SC) ; DeSerres, Jean

Subject: [EXTERNAL] Marketing and Advertising of Opioids / Le marketing et la publicité sur les opioïdes

---

Please find enclosed correspondence regarding the marketing and advertising of opioids.

Vous trouverez ci-joint une correspondance concernant le marketing et la publicité sur les opioïdes.

Ed Morgan
Director General
Policy, Planning and International Affairs Directorate

Dear Company X,

Further to the letter of June 19, 2018, from the Honourable Ginette Petitpas Taylor, Minister of Health, calling on industry to voluntarily cease the marketing and advertising of opioids to healthcare professionals, I am seeking confirmation of your support of Canada's collective response to the opioid crisis by immediately suspending any and all marketing and advertising of opioids to healthcare professionals.

In cases where you continue to share product materials with health care professionals, please be reminded that, as part of the recent terms and conditions imposed on specific opioid products under the authority of section C.01.014.21 of the *Food and Drug Regulations*, all product materials must be submitted to an advertising preclearance agency recognized by Health Canada for review and preclearance.

As part of the Government of Canada's commitment to openness and transparency, Health Canada publishes a table of all correspondence and meetings with stakeholders related to the restriction of marketing and advertising of opioids. Health Canada has made available on our website the Minister's letter, a list of recipient firms and all responses received. As such, this letter and any subsequent response will also be made available to the public. The Department will continue to publicly recognize the companies that respond to the Minister's request to suspend marketing and advertising to health care professionals.

The Government of Canada remains deeply concerned about the opioid crisis, which is affecting Canadians across all regions, and from all segments of society. I encourage you to provide a response by February 15, 2019.

Sincerely,

Ed Morgan
Director General, Policy Planning and International Affairs Directorate

Cc: Rhonda Kropp, Director General, Marketed Health Products Directorate

Chère compagnie,

La présente fait suite à la lettre du 19 juin 2018 de l'Honorable Ginette Petitpas Taylor, ministre de la Santé, dans laquelle elle appelait l'industrie à cesser volontairement le marketing et de publicité sur les opioïdes auprès des professionnels de la santé. Je souhaite obtenir la confirmation de votre engagement à la réponse collective du Canada à la crise des opioïdes, en suspendant immédiatement toute activité de marketing et de publicité sur les opioïdes auprès des professionnels de la santé.

Dans les cas où vous continuez à partager des documents sur ces produits aux professionnels de la santé, sachez que, conforment aux récents conditions imposées sur des produits opioïdes en vertu de l'article C.01.014.21 du *Règlement sur les aliments et drogues*, tout document sur ces produits doit être présenté à un organisme de préapprobation de la publicité reconnu par Santé Canada afin d'être examiné et préautorisé.

Dans le cadre de l'engagement envers l'ouverture et la transparence du gouvernement du Canada, Santé Canada publie un tableau de toute la correspondance et de toutes les réunions avec les intervenants concernant la restriction du marketing et de la publicité sur les opioïdes. Santé Canada a publié sur son site Web la lettre de la ministre, une liste des entreprises visées et toutes les réponses reçues. Par conséquent, la présente lettre et toute réponse subséquente seront également rendues publiques. Le Ministère continuera à reconnaître publiquement les entreprises qui ont répondu à l'appel de la ministre de suspendre le marketing et le publicité sur les opioïdes auprès des professionnels de la santé.

Le gouvernement du Canada reste profondément préoccupé par la crise des opioïdes qui touche les Canadiennes et les Canadiens de toutes les régions et de tous les segments de la société. Je vous invite à répondre à la présente lettre d'ici le 15 février 2019.

Mes sincères salutations,

Ed Morgan
Directeur général, Direction des politiques, de la planification et des affaires internationales

c.c. : Rhonda Kropp, Directrice générale, Direction des produits de santé commercialisés

From: Paladugu, Sudheer
Sent: 2019-01-31 5:00 PM
To: Morgan, Ed (HC/SC)
Cc: Kropp, Rhonda (HC/SC) ; Aggarwal, Atul

Subject: FW: Marketing and Advertising of Opioids / Le marketing et la publicité sur les opioïdes

Good afternoon,
Further to your office letter (as attached), we would like to confirm that Marcan Pharmaceuticals Inc., is not marketing or promoting or advertising any opiod products.

We would like to bring to your notice that our products are dispensed and sold to wholesalers and or direct to pharmacy as per the purchase orders from respective vendors.

Should you have any questions or need any information, please do not hesitate to contact us.

Regards,

SUDHEER PALADUGU I Sr. VICE PRESIDENT –TECHNICAL
E: [redacted]@marcanpharma.com **I T**: 613.228.2600 x[redacted] **I F**: 613.224.0444 **I C**: [redacted]
A: 2 Gurdwara Rd. Suite #112, Ottawa, ON, K2E 1A2 **I W**: www.marcanpharma.com

---

From: Morgan, Ed (HC/SC) [mailto:ed.morgan@canada.ca]
Sent: January 31, 2019 3:32 PM
To: Aggarwal, Atul
Cc: Kropp, Rhonda (HC/SC); Paladugu, Sudheer
Subject: Marketing and Advertising of Opioids / Le marketing et la publicité sur les opioïdes

Please find enclosed correspondence regarding the marketing and advertising of opioids.

Vous trouverez ci-joint une correspondance concernant le marketing et la publicité sur les opioïdes.

Ed Morgan
Director General
Policy, Planning and International Affairs Directorate

Notice & Disclaimer - This email and any files transmitted with it contain Proprietary, privileged and confidential information and/or information protected by intellectual property rights and is only for the use of the intended recipient of this message. If you are not the intended recipient, please delete or destroy this and all copies of this message along with the attachments immediately. You are hereby notified and directed that (1) if you are not the named and intended addressee you shall not disseminate, distribute or copy this e-mail, and (2) any offer for product/service shall be subject to a final evaluation of relevant patent status. Company cannot guarantee that e-mail communications are secure or error-free, as information could be intercepted, corrupted, amended, lost, destroyed, arrive late or incomplete, or may contain viruses. Company does not accept responsibility for any loss or damage arising from the use of this email or attachments.

Recipients of Health Canada's Letter January 31, 2019/ Destinataires de la lettre de Santé Canada le 31 janvier 2019
1. Apotex Inc.<br>2. Auro Pharma Inc.<br>3. Church & Dwight Canada Corporation<br>4. Cipher Pharmaceuticals Inc.<br>5. Endo Ventures Inc.<br>6. ICU Medical Canada<br>7. JAMP Pharma Corporation<br>8. Laboratoire Atlas<br>9. Mallinckrodt Canada ULC<br>10. Marcan Pharmaceuticals Inc.<br>11. Pharmapar Inc.<br>12. Sanis Health Inc. (Part of Shopper's Drug Mart)<br>13. Sivem Pharmaceuticals ULC<br>14. Taro Pharmaceuticals Inc.<br>15. Valeant Canada LP

CHURCH & DWIGHT CANADA CORP.

February 4th, 2019

Marketed Health Products Directorate
A.L 1912C
Ottawa, Ontario
K1A0K9

Re: Marketing and advertisement of Opioids to Healthcare Professionals

---

Dear Mr. Morgan,

Further to your letter dated January 31st, 2019, we are pleased to provide some clarification.

We are confirming that Church & Dwight Canada Corp. does not sell or advertise any of its controlled substance to Healthcare professionals. Also, please note that both of our opioid products, Atasol 15 and Atasol 30 will be discontinued as of the following dates:

Medicinal Ingredient	Product Name	DIN	Discontinuation Date	Drug Shortage Website Posting	Run Out Date
Codeine Phosphate	Atasol 15	00293504	Dec 31, 2019	July 2019	Jan, 2020
	Atasol 30	00293512	May 31, 2019	October 22, 2018	April 29, 2019

This information was also sent to Health Canada on November 13th, 2018 and January 15th, 2019 in response to the terms and conditions imposed on specific opioid products. In light of this information, we were granted to not send the Canadian Specific Opioid Targeted Risk Management Plan (CSO-tRMP) and therefore no need to submit information related to these products to a preclearance agency advertisement.

Please, feel free to contact the undersigned if you have any questions.

Sincerely,

[redacted]

Elaine Moreau
Director Quality and Regulatory Affairs
Church & Dwight Canada Corporation
[redacted]@churchdwight.com
+ 1(438) 844-0090

CC: Michael Read

Pharmapar
1565, boul. Lionel-Boulet
Varennes, QC J3X 1P7

February 4, 2019

Ed Morgan
Director General, Policy Planning
and International Affairs Directorate
Health Canada

Dear Sir,

We are pleased to respond to your letter of January 31, 2019.

We respectfully submit that other than providing a copy of the authorized Product Monograph to the practitioners upon their request, I hereby confirm that Pharmapar Inc. does not provide any information or conduct any marketing or promotional activities for opioid products. Further, the authorized Product Monograph is a document which is publicly available on Health Canada website (drug product database).

Pharmapar Inc. fully supports Health Canada's initiative in response to the opioid crisis.

Sincerely,

[redacted]

Marc Beaudoin
CEO, Pharmapar Inc.

Copies to:
Rhonda Kropp, Director General, Marketed to Health Products Directorate
Barry Poole, Director Quality Control and Regulatory Affairs, Pharmapar Inc.

info@pharmapar.ca | 1-866-831-2003

-------- Original message --------
From: "Ruth Moses (SNS)"
Date: 2019-02-05 1:38 PM (GMT-05:00)
To: "Morgan, Ed (HC/SC)"
Cc: "Kropp, Rhonda (HC/SC)" , "Chris Potter (SDM)"

Subject: RE: Marketing and Advertising of Opioids / Le marketing et la publicité sur les opioïdes

Dear Mr. Morgan,

Please accept this letter as Sanis Health's acknowledgement of the correspondence received January 31, 2019 and our commitment to fully support the efforts to address Canada's opioid crisis. Sanis Health does not currently participate in any advertising or marketing to healthcare professionals. Any activities that may be undertaken in the future, will fully comply with the terms and conditions on specific opioid products under authority of section C.01.014.21 of the *Food and Drug Regulations*.

Regards,

Ruth Moses
Director, Sanis Regulatory Affairs and Quality Assurance

243 Consumers Road, Toronto, Ontario M2J 4W8
C: [redacted] ______________

This email message is confidential, may be legally privileged and is intended for the exclusive use of the addressee. If you received this message in error or are not the intended recipient, you should destroy the email message and any attachments or copies, and you are prohibited from retaining, distributing, disclosing or using any information contained. Please inform us of the delivery error by return email. Thank you for your cooperation.

From: Morgan, Ed (HC/SC) <ed.morgan@canada.ca>
Sent: Thursday, January 31, 2019 3:27 PM
To: Ruth Moses (SNS) [redacted]@sanis.com>
Cc: Kropp, Rhonda (HC/SC) <rhonda.kropp@canada.ca>; Chris Potter (SDM) [redacted]@shoppersdrugmart.ca>
Subject: [EXT] Marketing and Advertising of Opioids / Le marketing et la publicité sur les opioïdes

<u>CAUTION:</u> External email. Do not click links or open attachments unless you recognize the sender and know the content is safe.

Please find enclosed correspondence regarding the marketing and advertising of opioids.

Vous trouverez ci-joint une correspondance concernant le marketing et la publicité sur les opioïdes.

Ed Morgan
Director General
Policy, Planning and International Affairs Directorate

Sivem Pharmaceuticals

FEB 18 2019

BY COURIER

February 11, 2019

Health Canada
Health Products and Food Branch
Policy, Planning and International Affairs Directorate Graham Spry Building
Address Locator: 2005A
Ottawa, Ontario
K1A 0K9

Attention: Ed Morgan, Director General

Subject: **SIVEM Pharmaceuticals ULC--Call on the Pharmaceutical Industry to Voluntarily Suspend Marketing and Advertising of Opioids**

Dear Mr. Morgan,

This letter is in response to the correspondence dated January 31, 2019 sent to the attention of Paula Keays and related to the subject matter stated above.

SIVEM Pharmaceuticals ULC's ("SIVEM") current generic pharmaceutical product portfolio includes one molecule which is categorized as an opioid: Tramadol-Acetaminophen.

We wish to confirm that as it pertains to such molecule, SIVEM does not engage in marketing or advertising to healthcare professionals. Such molecule does continue to be included as part of SIVEM's overall product portfolio, and as such continues to be available to those health care professionals who, based on their professional judgement, consider that this molecule best meets the needs of their patient.

We thank you for your many ongoing efforts on this matter.

Sincerely,

[redacted]

Josie Romanelli
VP, Legal Affairs

CC: Paula Keays, President SIVEM Pharmaceuticals ULC

Laboratoire Atlas Inc.

9600, boul. des Sciences, Montréal, Québec, Canada, H1J 3B6. Tel : 514-254-7188 Fax : 514-254-3006

Montréal, le 12 février 2019

M. Ed Morgan, Directeur général
Direction des politiques, de la planification et des affaires internationales
Graham Spry Building, room 538 (AL :2005A)
250, Lanark Avenue
Ottawa, Ontario, K1A OK9

M. Morgan,

Par la présente, nous accusons réception de votre lettre du 31 janvier dernier. Nous sommes en accord avec les politiques de Santé Canada visant à enrayer la crise des opioïdes. En conséquence, nous nous engageons à ne pas partager de matériel faisant la promotion des opioïdes avec les professionnels de la santé canadiens.

En espérant le tout conforme,

Veuillez recevoir nos salutations sincères,

[redacted]

Mario Ostiguy [redacted]
Président
Laboratoire Atlas Inc.

icumedical
human connections

Commercial in Confidence

February 13, 2019

Office of Submissions and Intellectual Property
Therapeutic Products Directorate, Health Canada
101 Tunney's Pasture Driveway, Tunney's Pasture
Address Locator: 0201A1
Ottawa, Ontario, K1A 0K9

Subject: **Response to Correspondence Regarding the Marketing and Advertising of Opioids**
Morphine Sulfate Injection USP 1 mg/mL DIN 00636908
Morphine Sulfate Injection USP 5 mg/mL DIN 00649619
Regulatory Activity Type: UD-PV

Reference: **eCTD identifier: e209955; Sequence: 0009**

Dear Sir or Madam:

In response to the Correspondence Letter dated January 31, 2019 regarding the marketing and advertising of opioids, ICU Medical commits to suspend all marketing and advertising of opioids to healthcare professionals. Any product materials shared with healthcare professionals in the future will be submitted to an advertising preclearance agency recognized by Health Canada for review and preclearance.

We trust this is satisfactory. However, please do not hesitate to contact the undersigned using the contact information below should you have any questions.

Sincerely,

[redacted] 12-Feb-2019

Nicole Griffith [redacted]
Director, Global Regulatory Affairs
Phone: [redacted]
Fax: (514) 745-4388
Email: [redacted]@icumed.com

APOTEX
Innovating for
patient affordability

Jeff Watson
President & Chief Executive Officer
Apotex Inc.
150 Signet Dr.
Toronto, ON, M9L 1T9
Tel: 416-749-9026 ext. [redacted]
Email: [redacted]@apotex.com

February 13th, 2019

BY EMAIL, FOLLOWED BY COURIER

Ed Morgan
Director General, Policy Planning and International Affairs Directorate
Health Products and Food Branch
Graham Spry Building
Address Locator: 2005A
Ottawa, ON, K1A 0K9
E-mail: hc.ppiad-dppai.sc@canada.ca

Dear Mr. Morgan,

Apotex Inc. ("**Apotex**") wishes to thank you for your letter dated January 31, 2019. As a proud Canadian company, Apotex is deeply concerned about the opioid crisis in Canada. We support the Government's commitment to address this national public health crisis.

Apotex is cooperating with Health Canada's requirement to submit risk management plans. Further, Apotex confirms that it does not share product materials with doctors.

In your letter, you recall the Honourable Ginette Petitpas Taylor, Minister of Health's call on industry to voluntarily cease the marketing and advertising of opioids to health care professionals. Please note that due to the nature of Apotex's generic pharmaceutical business, Apotex does not engage in the practice of marketing and advertising opioids to doctors or the general public.

As a member of the Canadian Generic Pharmaceutical Association, Apotex endorses the position taken in the CGPA's letter to the Minister dated July 17, 2018 (enclosed). In particular, Apotex urges you to consider the following points made by the CGPA about:

(i) the differences between the business conducted by generic pharmaceutical companies (such as Apotex) and brand pharmaceutical companies;
(ii) the fact that generic pharmaceutical manufacturers (such as Apotex) do not play a role in influencing the prescribing decisions for opioid products;

(iii) the need to advise relevant stakeholders of the approval of a bioequivalent generic product by Health Canada and the availability of generic products; and
(iv) the measures in place for the control of narcotics.

We thank you once again for your letter and for the opportunity to submit this response.

Sincerely,
APOTEX INC.
[redacted]

Jeff Watson
President & Chief Executive Officer, Apotex Inc.

Enclosed: *CGPA Letter dated July 17, 2018*

cc:
The Honourable Ginette Petitpas Taylor, P.C., M.P.
Minister of Health
House of Commons
Ottawa, ON, K1A 0A6
Email: Ginette.PetitpasTaylor@parl.gc.ca

Rhonda Kropp
Director General, Marketed Health Products Directorate
Health Products and Food Branch
Health Canada
Address Locator 1912C
Ottawa, ON, K1A 0K9
E-mail: hc.mhpd-dpsc.sc@canada.ca

JAMP PHARMA
CORPORATION

VOTRE PARTENAIRE DE CONFIANCE
YOUR MOST TRUSTED PARTNER
jamppharma.com

Le jeudi 14 février 2019

Ed Morgan
Directeur général
Direction des politiques, de la planification et des affaires internationales
Édifice Graham Spry Building
250, avenue Lanark
Ottawa, Ontario
K1A 0K9

Cher M. Morgan,

Je vous écris aujourd'hui en réponse à votre lettre du 31 janvier 2019, ainsi que celle de la Ministre de la Santé du 19 juin 2018 sollicitant l'appui de l'industrie pharmaceutique afin de supporter les efforts du gouvernement canadien pour faire face à la crise des opioïdes qui sévit présentement au pays.

JAMP Pharma étant principalement une entreprise qui œuvre dans la distribution de médicaments génériques, nous ne faisons pas de promotion, marketing ou publicité sur les opioïdes auprès des professionnels de la santé.

En tant que fière entreprise canadienne, vous pouvez compter sur la collaboration de JAMP Pharma pour supporter les efforts du gouvernement canadien. Par votre entremise, je profite de cette occasion pour offrir mes sympathies et toute ma compassion à nos concitoyens et concitoyennes qui subissent les effets liés à la problématique des opioïdes.

Très cordialement,

[redacted]

Louis Pilon
Président propriétaire
Corporation JAMP Pharma

1310, rue Nobel, Boucherville Québec J4B 5H3
T 450 449-1236 | F 450 449-4326 | 1 866 399-9091

Health Canada Santé Canada

Canada

Restricting Marketing and Advertising of Opioids: Progress to Date and Views on Future Actions

May 14-15, 2019

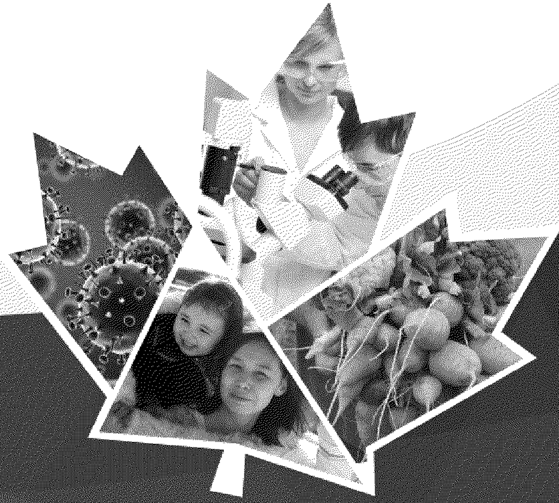

YOUR HEALTH AND SAFETY... OUR PRIORITY.

Purpose

- Provide an update on Health Canada's actions to date

- Seek views and feedback on:
  - opioid marketing and advertising practices, its influence on prescribing, and impact on patients
  - measures in place to guide health professionals' interactions with industry
  - ways to increase transparency in the marketing and promotion of opioids

Background

- More than 10,300 opioid-related deaths in Canada between January 2016 and September 2018.

- Prescription opioids have contributed to the current crisis.
  - Canadians are the second highest users per capita of prescription opioids in the world, and rates of opioid prescribing and opioid-related hospital visits and deaths have been increasing.

- Industry's marketing of opioids to health professionals can unduly influence prescribing practices.
  - Evidence suggests the information provided by industry to health care practitioners is less likely to include the harms compared to the benefits of a drug.
  - Industry's marketing and advertising can unduly influence physician prescribing, contributing to increased sales and availability of prescription opioids.
  - Physicians are often unaware of the impacts these marketing techniques have on their drug product knowledge which can lead to more frequent, expensive and poorer quality prescribing.

What We've Heard Through Consultations to Date

- Consultations included:
  - June 2018 Notice of Intent: 42 submissions received from industry, health professionals and organizations, and patients
  - September 2018 Opioid Symposium: Consultation session with over 200 participants in attendance with wide range of affiliations and experiences

- Stakeholders are supportive of restricting opioid marketing and advertising:
  - Some expressed a need for marketing and advertising restrictions for all prescription drugs
  - Others noted increased transparency of marketing and advertising of opioids is needed

- Stakeholders noted that any restrictions should:
  - Continue to allow access to educational/factual information
  - Ensure patient access to treatments for pain and opioid use disorder
  - Avoid increasing stigma associated with opioid use

Federal actions to address pharmaceutical industry marketing of opioids

Calling on industry to voluntarily cease opioid promotion

Imposing additional restrictions on marketing

Increasing openness and transparency

Calling on industry to voluntarily cease opioid promotion

- In June 2018, the Minister of Health called on opioid manufacturers and distributors to voluntarily cease opioid marketing activities to health care professionals in Canada until new restrictions are in place.
  - Health Canada's website indicates the status of companies that have responded

Imposing additional restrictions on marketing

- Opioids cannot be advertised directly to the public.
  - However, advertising to health care practitioners is permitted provided it is done in a manner that is not false or misleading.
- In October 2018, terms and conditions were added requiring that market authorization holders provide Health Canada with “Canadian Specific Opioid targeted Risk Management Plans”. As part of this requirement, all opioid advertisements must be pre-cleared through a recognized advertising preclearance agency to ensure they:
  - Present balanced information on benefits and risks; and,
  - Align with the Product Monograph

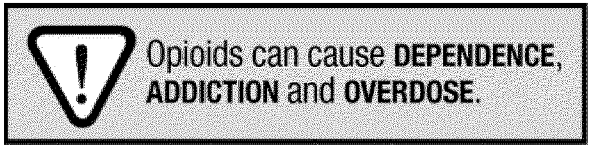

- Also, a warning sticker and a patient information handout must be provided with prescription opioids dispensed to Canadians at pharmacies or in doctors’ offices.
- In June 2019, additional Terms and Conditions on industry marketing and advertising of prescription opioid products to health care professionals will come into force.
- Advertisements for opioid products
  - Will be restricted to only statements, presented *verbatim*, that have been authorized by Health Canada in the Product Monograph
  - Must convey the benefits and risks in a balanced way

Increasing openness and transparency

- Health Canada has undertaken a number of actions to increase openness and transparency of actions relating to opioid marketing and advertising practices:

  - Records of relevant meetings and correspondence with stakeholders are made available to the public through a dedicated website.

  - New website launched on March 11, 2019 – the Stop Illegal Marketing of Drugs and Devices online platform that:
    - Aims to educate health care professionals on the rules governing the advertising of Canadian health products, including opioids
    - Makes it easy to file a complaint with Health Canada about promotional activities that may not be compliant with advertising rules.

Increasing openness and transparency

Help stop illegal marketing of drugs and devices

YOU REPORT • YOU PROTECT

As health experts, you work hard to help and protect your patients. Don't let your prescriptions be influenced by illegal marketing practices. By reporting this type of marketing, you can help Health Canada in our efforts to stop illegal marketing.

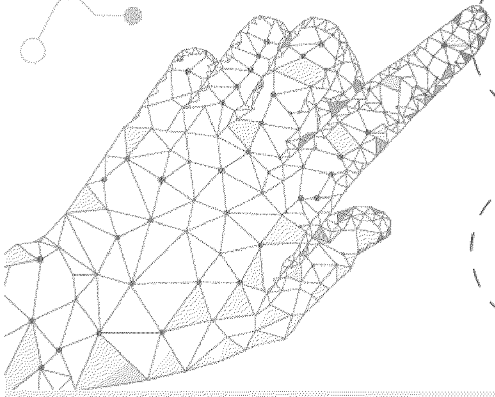

LEARN about Illegal Marketing of Drugs and Devices in Canada at canada.ca/drug-device-marketing

REPORT illegal marketing practices at drug-device-marketing@canada.ca

Pub.: 180815

Increasing openness and transparency

- Many stakeholder have roles to play in increasing transparency of industry marketing, and Health Canada is consulting on ways to increase transparency efforts with:
  - **Provinces/Territories:** Authority over practice of medicine
  - **Health Practitioners and Regulatory Bodies:** Guidelines for interactions with industry and disclosing conflicts of interest
  - **Industry:** Voluntary disclosure initiative, but low industry participation
  - **Patients/Public:** wary of inappropriate industry influence, but rely on products for treatment

Discussion

Opioid marketing and advertising practices

1. What is the nature of health professionals' interactions with industry and receipt – and influence of – of opioid marketing?
2. Have members ever complained to you about industry marketing and advertising practices?
3. How effective are measures in place to guide interactions between industry and health professionals?
4. What, in your view, are the impacts of marketing practices on prescribing practices? On patients?
5. What other actions would be most effective in mitigating potential undue industry influence?

Views on increasing transparency

6. What are your organization/member views on increasing transparency of industry interactions with health care professionals?
7. What measures could support increased transparency?
8. What role could your organization play?

Next Steps

- Information from this meeting will inform potential federal action related to the marketing and advertising of opioids and to increasing transparency of opioid marketing practices.

- This meeting will be listed on Health Canada's Meetings and Correspondence on marketing and advertising of opioids webpage.
  - The presentation and a summary of the meeting will be publicly available on request.

Annex A: Examples of marketing and advertising activities

Activities

- ✓ Print material and information shared at conferences (e.g. ads in medical journals, promotional messages)
- ✓ Design of continuing education courses or materials
- ✓ Proactive sales rep visits to distribute information and materials, including drug samples
- ✓ Industry funding of speakers, conferences, and events, gifts, meals

Target Audience

All health professionals, including:

- ✓ Family physicians
- ✓ Surgeons
- ✓ Specialists (including pain)
- ✓ Registered nurses and nurse practitioners
- ✓ Dentists
- ✓ Pharmacists
- ✓ Medical students

Annex B: Health Canada's response to the opioid crisis

Major Federal Investments

- **Budget 2017** ($5B for mental health and addictions).
- **Budget 2018** ($150M for an Emergency Treatment Fund; $13M/5 years for public education on stigma).
- **Budget 2019** ($22.3M over 4 years to support naloxone access and community-based opioid overdose response training; $8.1M/ 5 years, with $1.0M ongoing, to support safe supply).

Health Canada Approach

Increasing access to treatment	Emergency Treatment Fund provided $150M to provinces and territories to improve access to evidence-based treatment.
Increasing harm reduction	Naloxone made available without a prescription; Good Samaritan Overdose Act.
Increasing education and awareness	Updated guidelines for opioid prescribing; Opioid warning sticker/ patient handout.
Reducing stigma and promoting compassion to reduce barriers	Launched national stigma public education campaign; Developed/shared stigmatizing language lexicon.
Increasing evidence	Established a Canadian Pain Task Force to improve the prevention and management of chronic pain in Canada.

Health Canada Santé Canada

Canada

Restriction du marketing et de la publicité des opioïdes : progrès réalisés à ce jour et points de vue sur les mesures à prendre

14-15 mai 2019

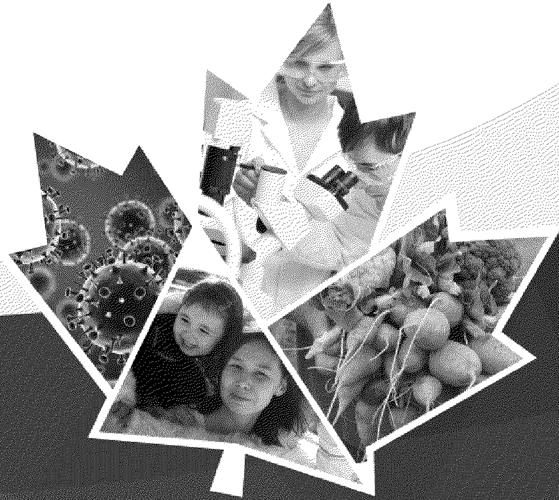

Objet

- Faire le point sur les mesures prises par Santé Canada à ce jour

- Obtenir des points de vue et des commentaires sur :
  - les pratiques de marketing et de publicité sur les opioïdes, leur influence sur la prescription et leur incidence sur les patients;
  - les mesures en place pour guider les interactions des professionnels de la santé avec l'industrie;
  - les façons d'accroître la transparence du marketing et de la promotion des opioïdes.

Renseignements généraux

- Plus de 10 300 décès liés aux opioïdes au Canada entre janvier 2016 et septembre 2018.

- Les opioïdes d'ordonnance ont contribué à la crise actuelle.
  - Les Canadiens sont les deuxièmes plus grands consommateurs d'opioïdes d'ordonnance par habitant au monde, et les taux de prescription d'opioïdes ainsi que le nombre de visites et de décès à l'hôpital liés aux opioïdes ont augmenté.

- Le marketing des opioïdes par l'industrie auprès des professionnels de la santé peut influencer indûment les pratiques de prescription.
  - Les preuves suggèrent que l'information fournie par l'industrie aux professionnels de la santé est moins susceptible d'inclure les effets nocifs en comparaison aux avantages d'un médicament.
  - Le marketing et la publicité de l'industrie peuvent influencer indûment la prescription par les médecins, ce qui contribue à l'augmentation des ventes et de la disponibilité des opioïdes d'ordonnance.
  - Souvent, les médecins ne sont pas conscients des répercussions de ces techniques de marketing sur leur connaissance des produits pharmaceutiques, ce qui peut mener à des ordonnances plus fréquentes, plus coûteuses et de moins bonne qualité.

Ce que nous avons entendu au cours des consultations jusqu'à maintenant

- Les consultations comprenaient :
  - l'avis d'intention de juin 2018 : 42 réponses reçues de l'industrie, des professionnels et organismes de la santé, et des patients;
  - le symposium sur les opioïdes de septembre 2018 : Séance de consultation avec plus de 200 participants provenant d'horizons divers.
- Les intervenants appuient la restriction du marketing et de la publicité des opioïdes :
  - Certains étaient d'avis qu'il est nécessaire de restreindre le marketing et la publicité pour tous les médicaments d'ordonnance;
  - D'autres ont mentionné le besoin d'augmenter la transparence dans le marketing et la publicité des opioïdes.
- Les intervenants ont fait remarquer que toute restriction devrait :
  - continuer de permettre l'accès à de l'information éducative/factuelle;
  - assurer aux patients l'accès à des traitements contre la douleur et pour les troubles liés à l'utilisation d'opioïdes;
  - éviter d'accroître la stigmatisation associée à la consommation d'opioïdes.

Actions fédérales pour répondre au marketing des opioïdes par l'industrie pharmaceutique.

Appel à l'industrie à cesser volontairement les activités promotionnelles

Imposer des restrictions additionnelles sur le marketing

Accroître l'ouverture et la transparence

Appel à l'industrie à cesser volontairement les activités promotionnelles

- En juin 2018, la ministre de la Santé a lancé un appel aux fabricants et aux distributeurs d'opioïdes à cesser volontairement les activités de marketing des opioïdes auprès des professionnels de la santé au Canada jusqu'à ce que de nouvelles restrictions soient mises en place.
  - Le site Web de Santé Canada indique le statut des entreprises qui ont répondu

Imposer des restrictions additionnelles sur le marketing

- Les opioïdes ne peuvent pas être annoncés directement au public.
  - Toutefois, la publicité destinée aux professionnels de la santé est permise à condition qu'elle ne soit pas fausse ou trompeuse.
- En octobre 2018, des conditions ont été ajoutées exigeant que les titulaires d'une autorisation de mise en marché donnent à Santé Canada leur « plans canadiens ciblant la gestion des risques spécifiques aux opioïdes ». Dans le cadre de cette exigence, toutes les publicités sur les opioïdes doivent recevoir l'autorisation au préalable d'un organisme de pré-approbation de la publicité reconnue afin de s'assurer qu'ils :
  - présentent des renseignements impartiaux sur les avantages et les risques;
  - respectent l'information de la monographie du produit.

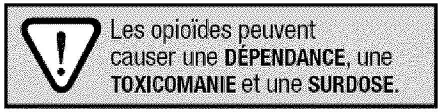

- De plus, un autocollant d'avertissement et une fiche d'information pour le patient doivent être fournis avec les opioïdes d'ordonnance distribués aux Canadiens dans les pharmacies ou dans les bureaux de médecins.
- En juin 2019, des conditions supplémentaires sur le marketing et la publicité des produits opioïdes d'ordonnance par l'industrie auprès des professionnels de la santé entreront en vigueur.
- La publicité pour les produits opioïdes :
  - sera limitée aux énoncés qui ont été autorisés par Santé Canada dans la monographie du produit, présentés *textuellement*;
  - doit présenter les avantages et les risques de façon impartiale.

Accroître l'ouverture et la transparence

- Santé Canada a pris un certain nombre de mesures pour accroître l'ouverture et la transparence des mesures relatives aux pratiques de marketing et de publicité liées aux opioïdes :

  - Les comptes rendus des réunions pertinentes et de la correspondance avec les intervenants sont mis à la disposition du public par l'entremise d'un site Web réservé à cette fin.

  - Un nouveau site Web lancé le 11 mars 2019 – la plateforme en ligne Mettons fin au marketing illégal des médicaments et des instruments médicaux :
    – vise à sensibiliser les professionnels de la santé aux règles régissant la publicité des produits de santé canadiens, y compris les opioïdes;
    – facilite le dépôt d'une plainte auprès de Santé Canada au sujet d'activités de promotion qui pourraient ne pas être conformes aux règles de publicité.

Accroître l'ouverture et la transparence

Contribuez à mettre un terme au marketing illégal des médicaments et des instruments médicaux

VOUS SIGNALEZ • VOUS PROTÉGEZ

En tant qu'expert de la santé, vous travaillez fort pour aider et protéger vos patients. Ne laissez pas les pratiques illégales de marketing influencer vos ordonnances. En reportant ce genre de marketing, vous pouvez aider Santé Canada dans nos efforts pour mettre un terme aux pratiques illégales de marketing.

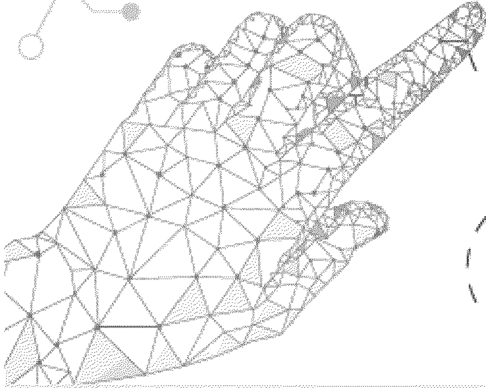

APPRENEZ au sujet du marketing illégal des médicaments et des instruments médicaux au Canada à l'adresse canada.ca/medicament-instrument-marketing

SIGNALEZ les pratiques de marketing illégales à medicament-instrument-marketing@canada.ca

Pub. : 180815

Accroître l'ouverture et la transparence

- De nombreux intervenants ont un rôle à jouer pour accroître la transparence du marketing de l'industrie, et Santé Canada mène des consultations sur les façons d'accroître la transparence :
  - **avec les provinces et les territoires :** Régissent la pratique de la médecine;
  - **les praticiens de la santé et les organismes de réglementation :** Lignes directrices sur les interactions avec l'industrie et divulgation des conflits d'intérêt;
  - **Industrie :** Initiative de divulgation volontaire, mais faible participation de l'industrie;
  - **Patients et grand public :** Se méfie de l'influence indue de l'industrie, mais se fie aux produits pour le traitement.

Discussion

Pratiques de marketing et de publicité sur les opioïdes

1. Quelle est la nature des interactions des professionnels de la santé avec l'industrie et la réception – et l'influence – du marketing des opioïdes?
2. Vos membres vous ont-ils déjà adressé des plaintes au sujet des pratiques de marketing et de publicité de l'industrie?
3. Les mesures mises en place pour guider les interactions entre l'industrie et les professionnels de la santé sont-elles efficaces?
4. À votre avis, quelles sont les répercussions des pratiques de marketing sur les pratiques de prescription? Sur les patients?
5. Quelles autres mesures seraient les plus efficaces pour atténuer l'influence indue potentielle de l'industrie?

Opinions sur l'accroissement de la transparence

6. Quels sont les points de vue de votre organisation ou de vos membres sur l'accroissement de la transparence des interactions entre l'industrie et les professionnels de la santé?
7. Quelles mesures pourraient favoriser une plus grande transparence?
8. Quel rôle votre organisation pourrait-elle jouer?

Prochaines étapes

- Les renseignements recueillis pendant cette réunion éclaireront les mesures fédérales potentielles liées au marketing et à la publicité des opioïdes et à l'accroissement de la transparence des pratiques de marketing des opioïdes.

- Cette réunion figurera sur la page Web Réunions et correspondance sur le marketing et la publicité sur les opioïdes de Santé Canada.
    - La présentation et un résumé de la réunion seront accessibles au public sur demande.

Annexe A : Exemples d’activités de marketing et de publicité

Activités

- ✓ Documents imprimés et information échangée lors de conférences (p. ex., annonces dans les journaux médicaux, messages promotionnels)
- ✓ Conception de cours ou de matériel de formation continue
- ✓ Visites proactives des représentants des ventes pour distribuer de l’information et du matériel, y compris des échantillons de médicaments
- ✓ Financement par l’industrie de conférenciers, de conférences et d’événements, de cadeaux et de repas

Public cible

Tous les professionnels de la santé, y compris :

- ✓ les médecins de famille;
- ✓ les chirurgiens;
- ✓ les spécialistes (y compris ceux qui traitent la douleur);
- ✓ les infirmières autorisées et les infirmières praticiennes;
- ✓ les dentistes :
- ✓ les pharmaciens;
- ✓ les étudiants en médecine.

Annexe B : Réponse de Santé Canada à la crise des opioïdes

Principaux investissements fédéraux

- **Budget de 2017** (5 G$ pour la santé mentale et les problèmes de dépendance).
- **Budget de 2018** (150 M$ pour un Fonds de traitement d'urgence; 13 M$ sur 5 ans pour l'éducation du public sur la stigmatisation).
- **Budget de 2019** (22,3 M$ sur quatre ans pour appuyer l'accès à la naloxone et la formation communautaire sur l'intervention en cas de surdose d'opioïdes; 8,1 M$/5 ans, avec 1,0 M$ en continu, pour appuyer l'approvisionnement sécuritaire).

Approche de Santé Canada

Accroître l'accès au traitement	Le Fonds d'urgence pour le traitement a versé 150 millions de dollars aux provinces et aux territoires pour améliorer l'accès à des traitements fondés sur des données probantes.
Accroître la réduction des effets nocifs	Offrir de la naloxone sans ordonnance; *Loi sur les bons samaritains secourant les victimes de surdose*.
Accroître l'éducation et la sensibilisation	Lignes directrices mises à jour pour la prescription d'opioïdes; Autocollant d'avertissement sur les opioïdes/fiche du patient.
Réduire la stigmatisation et promouvoir la compassion pour réduire les obstacles	Lancement d'une campagne nationale d'éducation publique sur la stigmatisation; Élaboration et diffusion d'un lexique sur les termes stigmatisants.
Amasser plus de données probantes	Création d'un groupe de travail canadien sur la douleur afin d'améliorer la prévention et la prise en charge de la douleur chronique au Canada.

Stakeholder Meeting Report
Health Care Professional Regulatory Bodies
And
Health Care Professional Associations
May 14 - 15, 2019

**Date:** May 14-15, 2019	**Meeting sheet completed by:**<br>Policy, Planning and International Affairs Directorate
**Subject:**<br>Restricting Marketing and Advertising of Opioids: Progress to Date and Views on Future Actions	**Stakeholder attendees:**<br>• Association of Faculties of Medicine of Canada<br>• Canadian Association of Schools of Nursing<br>• Canadian Centre on Substance Use and Addiction (CCSA)<br>• Canadian Dental Association<br>• Canadian Dental Regulatory Authorities Federation<br>• Canadian Federation of Medical Students<br>• Canadian Medical Association (CMA)<br>• Canadian Pharmacists Association (CPhA)<br>• Canadian Society of Addiction Medicine<br>• Canadian Society of Hospital Pharmacists<br>• College of Family Physicians of Canada<br>• Federation of Medical Regulatory Authorities of Canada (FMRAC)<br>• Nurse Practitioners Association of Canada<br>• Royal College of Physicians and Surgeons of Canada<br><br>**Government of Canada attendees:**<br>**Health Canada**<br>• Chief Medical Advisor for Health Canada and Senior Medical Advisor to Health Products and Food Branch<br>• Marketed Health Products Directorate<br>• Policy, Planning and International Affairs Directorate<br><br>**Public Health Agency of Canada**<br>• Chief Dental Officer of Canada

Purpose:

To provide an update on Health Canada's actions to date and to seek views and feedback from Health Care Professional Regulatory Bodies and Professional Associations on:

- Opioid marketing and advertising practices, its influence on prescribing, and impact on patients;
- Measures in place to guide health professionals' interactions with industry; and
- Ways to increase transparency in the marketing and promotion of opioids.

Notes:

A summary of key points is as follows:

- Health Canada presented an overview of the actions Health Canada has taken to address opioid marketing and advertising practices and to increase transparency and openness. These measures included a voluntary call to cease opioid marketing activities, further restrictions on the types of information that industry can share with health professionals, and ensuring the public has easy access to records of discussions between Health Canada and industry.

- Participants emphasized the importance of unbiased educational information to encourage appropriate prescribing. Educational programs and training are important, and a distinction should be made between activities that are promotional and those that support safe and appropriate prescribing.

- Participants noted that there is a need to address challenges in funding for continuing medical education (CME) for pain management using both pharmaceutical and non-pharmaceutical interventions. Respondents felt that funding for CME could contribute to the availability of unbiased education by reducing the need for industry-sponsored education.

- Participants stated the importance of increasing patient education options for pain management. Health Canada recognizes the impacts and challenges faced by Canadians and noted the establishment of the Canadian Pain Task Force. The Task Force is charged with providing advice to Health Canada regarding evidence and best practices for the prevention and management of chronic pain.

- Participants noted that physicians might have difficulty detecting when an advertisement is influencing their judgment, and/or lack knowledge on how to report concerns around inappropriate marketing or biased advertising. Participants sensed that more promotion and dissemination activities around the Stop Illegal Marketing of Drugs and Devices initiative would be valuable.

- Health Canada advised stakeholders on the existing guidelines on advertising and gave an overview of the procedure for reporting a complaint using the Stop Illegal Marketing of Drugs and Devices web-based platform when presented with a biased advertisement.

- Participants shared details regarding their organizations' current policies, guidelines and procedures in regard to interactions with industry. They also raised the importance of ensuring conflicts of interest are disclosed, both at conferences when speakers are sponsored by industry, and in educational materials.

Rapport de la réunion des intervenants
Organismes de réglementation des professionnels de la santé
et
Associations professionnelles des soins de santé
14 - 15 mai 2019

<table>
<tr><td>Date : 14 - 15 mai 2019</td><td>Feuille de réunion remplie par :
Direction des politiques, de la planification et des affaires internationales</td></tr>
<tr><td>Objet :
Restriction du marketing et de la publicité des opioïdes : progrès accomplis à ce jour et points de vue sur les mesures futures</td><td>Intervenants présents à la réunion :
Association des facultés de médecine du Canada
Association canadienne des écoles de sciences infirmières
Centre canadien sur les dépendances et l’usage de substances (CCDUS)
Association dentaire canadienne
Fédération canadienne des organismes de réglementation dentaire
Fédération des étudiants et des étudiantes en médecine du Canada
Association médicale canadienne (AMC)
Association des pharmaciens du Canada (APhC)
Société médicale canadienne sur l’addiction
Société canadienne des pharmaciens d’hôpitaux
Collège des médecins de famille du Canada
Fédération des ordres des médecins du Canada (FOMR)
Association des infirmières et infirmiers praticiens du Canada
Collège royal des médecins et chirurgiens du Canada
Participants du gouvernement du Canada :
Santé Canada
Conseillère médicale en chef pour Santé Canada et conseillère médicale principale de la Direction générale des produits de santé et des aliments
Direction des produits de santé commercialisés
Direction des politiques, de la planification et des affaires internationales
Agence de la santé publique du Canada
Dentiste en chef du Canada</td></tr>
<tr><td colspan="2">But :

Faire le point sur les mesures prises par Santé Canada à ce jour et solliciter les points de vue et les commentaires des organismes professionnels de réglementation des soins de santé et des associations professionnelles sur :
Les pratiques de marketing et de publicité des opioïdes, leur influence sur la</td></tr>
</table>

prescription et leur impact sur les patients;
- Les mesures en place pour guider les interactions des professionnels de la santé avec l'industrie;
- Les moyens d'accroître la transparence dans le marketing et la promotion des opioïdes.

Remarques :

Voici un résumé des principaux points :

- Santé Canada a présenté un aperçu des mesures prises pour traiter de la question des pratiques du marketing et de la publicité des opioïdes et pour accroître la transparence et l'ouverture. Ces mesures comprenaient un appel à cesser volontairement des activités de marketing des opioïdes, des restrictions supplémentaires quant aux types d'information que l'industrie peut communiquer aux professionnels de la santé et des mesures pour permettre au public d'accéder facilement aux comptes rendus des discussions entre Santé Canada et l'industrie.

- Les participants ont souligné l'importance d'une information éducative impartiale pour encourager l'adoption d'un comportement de prescription approprié. Les programmes éducatifs et la formation sont importants et une distinction devrait être faite entre les activités promotionnelles et celles qui permettent de prescrire les opioïdes de façon sûre et appropriée.

- Les participants ont souligné la nécessité de relever les défis liés au financement de la formation médicale continue (FMC) pour la gestion de la douleur au moyen d'interventions pharmaceutiques et non pharmaceutiques. Les répondants sont d'avis que le financement de la FMC pourrait contribuer à la disponibilité d'un enseignement impartial en réduisant le besoin d'un enseignement commandité par l'industrie.

- Les participants ont souligné l'importance d'accroître les possibilités d'éducation des patients concernant la gestion de la douleur. Santé Canada reconnaît les impacts et les défis auxquels les Canadiens font face et a fait remarquer la création du Groupe de travail canadien sur la douleur. Le groupe de travail est chargé de fournir des conseils à Santé Canada sur les données scientifiques et les meilleures pratiques concernant la prévention et la gestion de la douleur chronique.

- Les participants ont souligné que les médecins pouvaient avoir du mal à détecter l'influence d'une publicité sur leur jugement et/ou ne pas savoir comment signaler leurs préoccupations concernant un marketing inapproprié ou une publicité biaisée. Les participants estimaient qu'il serait utile qu'il y ait davantage d'activités de promotion et de diffusion dans le cadre de l'initiative Mettons fin au marketing illégal des médicaments et des instruments médicaux.

- Santé Canada a informé les intervenants des lignes directrices existantes concernant la publicité et leur a donné un aperçu de la procédure à suivre pour signaler une plainte à l'aide de la plateforme Web Mettons fin au marketing illégal des médicaments et des instruments médicaux lorsqu'une publicité biaisée leur est présentée.

- Les participants ont partagé des détails sur les politiques, lignes directrices et procédures en vigueur dans leurs organisations en ce qui concerne les interactions avec l'industrie. Ils ont également souligné l'importance de veiller à ce que les conflits d'intérêts soient divulgués, tant lors des conférences commanditées par l'industrie que dans le matériel pédagogique.

January 31, 2019

Ms. Ciara Walsh
Director, Regulatory Affairs
Endo Ventures Inc.

Dear Ms. Walsh,

Further to the letter of June 19, 2018, from the Honourable Ginette Petitpas Taylor, Minister of Health, calling on industry to voluntarily cease the marketing and advertising of opioids to healthcare professionals, I am seeking confirmation of your support of Canada's collective response to the opioid crisis by immediately suspending any and all marketing and advertising of opioids to healthcare professionals.

In cases where you continue to share product materials with health care professionals, please be reminded that, as part of the recent terms and conditions imposed on specific opioid products under the authority of section C.01.014.21 of the *Food and Drug Regulations*, all product materials must be submitted to an advertising preclearance agency recognized by Health Canada for review and preclearance.

As part of the Government of Canada's commitment to openness and transparency, Health Canada publishes a table of all correspondence and meetings with stakeholders related to the restriction of marketing and advertising of opioids. Health Canada has made available on our website the Minister's letter, a list of recipient firms and all responses received. As such, this letter and any subsequent response will also be made available to the public. The Department will continue to publicly recognize the companies that respond to the Minister's request to suspend marketing and advertising to health care professionals.

The Government of Canada remains deeply concerned about the opioid crisis, which is affecting Canadians across all regions, and from all segments of society. I encourage you to provide a response by February 15, 2019.

Sincerely,

[redacted]

Ed Morgan
Director General, Policy Planning and International Affairs Directorate

Cc: Rhonda Kropp, Director General, Marketed Health Products Directorate

Health Canada | Santé Canada
Health Products and Food Branch | Direction générale des produits de santé et des aliments

January 31, 2019

Mr. Atul Aggarwal
President and CEO
Marcan Pharmaceuticals Inc.

Dear Mr. Aggarwal,

Further to the letter of June 19, 2018, from the Honourable Ginette Petitpas Taylor, Minister of Health, calling on industry to voluntarily cease the marketing and advertising of opioids to healthcare professionals, I am seeking confirmation of your support of Canada's collective response to the opioid crisis by immediately suspending any and all marketing and advertising of opioids to healthcare professionals.

In cases where you continue to share product materials with health care professionals, please be reminded that, as part of the recent terms and conditions imposed on specific opioid products under the authority of section C.01.014.21 of the Food and Drug Regulations, all product materials must be submitted to an advertising preclearance agency recognized by Health Canada for review and preclearance.

As part of the Government of Canada's commitment to openness and transparency, Health Canada publishes a table of all correspondence and meetings with stakeholders related to the restriction of marketing and advertising of opioids. Health Canada has made available on our website the Minister's letter, a list of recipient firms and all responses received. As such, this letter and any subsequent response will also be made available to the public. The Department will continue to publicly recognize the companies that respond to the Minister's request to suspend marketing and advertising to health care professionals.

The Government of Canada remains deeply concerned about the opioid crisis, which is affecting Canadians across all regions, and from all segments of society. I encourage you to provide a response by February 15, 2019.

Sincerely,

[redacted]

Ed Morgan
Director General, Policy Planning and International Affairs Directorate

Cc: Rhonda Kropp, Director General, Marketed Health Products Directorate

Canada

Notice to Stakeholders: Further Restrictions to the Marketing and Advertising of Opioids

Health Canada is informing Market Authorization Holders (MAHs) of proposed enhanced Terms and Conditions (T&Cs) on Class B opioid products. This would serve to further restrict the marketing and advertising of opioids under the authority of section C.01.014.21 of the *Food and Drug Regulations*. Enhanced T&Cs will be finalized pending comments received from impacted stakeholders.

Background

Prescriptions written by health care professionals are a common source of opioids in Canada. Health care professionals receive information from a variety of sources to inform their prescribing decisions and advice to patients, including from the pharmaceutical industry. While there is value in the pharmaceutical industry conveying educational and scientific information about a health product, evidence suggests that the marketing and advertising of opioids has contributed to increased sales and availability of prescription opioids which may have contributed to Canada's opioid crisis.

In May 2018, regulations which permitted the Minister of Health to impose T&Cs on opioid products were introduced. In October 2018, through these T&Cs, Health Canada advised Market Authorization Holders (MAHs) that they must provide the Department with Canadian Specific Opioid targeted Risk Management Plans (CSO-tRMP) for their Class B opioid products. As part of this requirement, MAHs must submit for preclearance to an external advertising preclearance agency (APA) recognized by Health Canada all materials regarding opioid products they intend to provide to health care professionals. In carrying out their functions, the APAs will provide advice on whether the materials are consistent with the terms of the product's marketing authorization, and consider whether any statements are false or misleading.

Furthermore, in June 2018, Health Canada published a Notice of Intent to restrict the marketing and advertising of opioids. A summary of the feedback received from health care professionals and associations, patients, academics, and pharmaceutical industry representatives was published in December 2018. Many respondents were generally supportive of restrictions on marketing and advertising of opioids, and noted the importance of unbiased educational information to encourage appropriate prescribing.

Proposed Further Restrictions

Recognizing the urgency of the opioid crisis and the role that the marketing and advertising of opioids is playing, Health Canada is considering further restricting the marketing and advertising of opioids by imposing enhanced T&Cs for prescription opioid-containing products. **The enhanced T&Cs will restrict all promotional Class B opioid-related materials provided to health care professionals to only statements that have been authorized by Health Canada in**

the Product Monograph, such as the therapeutic claims, contraindications and side effects. Preclearance of advertising materials will continue to be required to help implement the latter restrictions. This approach will encourage appropriate prescribing by health care professionals who are provided with evidence-based, unbiased information that conveys the benefits and risks of such products in a balanced way.

Advertising of controlled drugs, including Class B opioids, must be consistent with the *Food & Drugs Act*, the *Controlled Drugs and Substances Act*, and associated regulations.

Next steps

Health Canada is communicating with MAHs and key stakeholders to provide an opportunity to submit comments on this approach. Health Canada plans to notify individual MAHs of the enhanced T&Cs by April 2019 with the expectation that all promotional materials developed as of June 2019 would be required to comply with the enhanced T&Cs.

Questions

Questions concerning the proposed additional restriction should be directed to MHPD_DPSC-Advertising_Reg_Publicite@hc-sc.gc.ca.

DRAFT

Prescription Opioids Risk Management Plan Tracker

As part of Health Canada's ongoing commitment to openness and transparency, the Department is publishing information regarding the status of risk management plans for prescription opioids.

Health Canada has identified the need for strengthened post-market oversight of opioids to help monitor and mitigate their risks. As one part of this initiative, the Department has mandated that manufacturers of Class B opioids submit risk management plans with a focus on opioid-related harms.[1]

A risk management plan is a living document developed by the sponsor or authorization holder of a drug. It consists of a set of activities and interventions designed to monitor, quantify, characterize and mitigate risks relating to the product. It may also include a means for assessing the effectiveness of any interventions.

How the Tracker Works

- The tracker highlights when manufacturers have submitted mandated opioid-specific RMPs to Health Canada; and the status of Health Canada's review of these documents. It also indicates situations in which manufacturers have not submitted mandated RMPs within required deadlines.
- Even if a manufacturer is listed on the tracker as not having submitted an RMP, it doesn't necessarily mean there is an immediate risk to the health of Canadians.
- Continued failure to comply with the Terms and Conditions is considered a contravention of section 21.7 of the *Food and Drugs Act* which could result in more serious compliance and enforcement action under that Act. Compliance and enforcement outcomes will be determined and listed in accordance with a risk-based approach, aligned with departmental policies, including compliance promotion activities.
- This tracker is updated regularly and was last updated on: January 21, 2019

[1] Link to CG2: http://gazette.gc.ca/rp-pr/p2/2018/2018-05-02/html/sor-dors77-eng.html

Active Ingredient	Brand Name (DINs)	Manufacturer	Deadline for RMP Submission	Date RMP received by Health Canada	Status of Health Canada's Review	Additional Comments
codeine (codeine monohydrate, codeine sulfate trihydrate)	CODEINE CONTIN 100MG CONTROLLED RELEASE TAB	Purdue Pharma	2019-01-15	2019-01-15	Ongoing	N/A
codeine (codeine monohydrate, codeine sulfate trihydrate)	CODEINE CONTIN 150MG CONTROLLED RELEASE TAB	Purdue Pharma	2019-01-15	2019-01-15	Ongoing	N/A
codeine (codeine monohydrate, codeine sulfate trihydrate)	CODEINE CONTIN 200MG CONTROLLED RELEASE TAB	Purdue Pharma	2019-01-15	2019-01-15	Ongoing	N/A
codeine (codeine monohydrate, codeine sulfate trihydrate)	CODEINE CONTIN 50MG CONTROLLED RELEASE TAB	Purdue Pharma	2019-01-15	2019-01-15	Ongoing	N/A
codeine phosphate	ACETAMINOPHEN ELIXIR WITH 8MG CODEINE PHOSPHATE SYRUP	JAMP Pharma Corporation	2019-01-15	019-01-16	Ongoing	N/A
codeine phosphate	CALMYLIN ACE	Teva Canada Limited	2019-01-15	2019-01-15	Ongoing	N/A
codeine phosphate	CALMYLIN	Teva Canada Limited	2019-03-12	2019-01-15	Ongoing	N/A

codeine phosphate	CALMYLIN PSE WITH CODEINE	Teva Canada Limited	2019-03-12	2019-01-15	Ongoing	N/A
codeine phosphate	CODEINE 30	Laboratoire Riva Inc.	2019-01-15	2019-01-14	Ongoing	N/A
codeine phosphate	CODEINE PHOSPHATE INJECTION USP	Sandoz Canada Incorporated	2019-01-15	2019-01-15	Ongoing	N/A
codeine phosphate	CODEINE PHOSPHATE SYRUP	Laboratoire Atlas Inc.	2019-01-15	Pending	Pending	N/A
codeine phosphate	DIMETANE EXPECTORANT C	Pfizer Consumer HealthCare a Division of Pfizer Canada Inc.	2019-01-15	2019-01-15	Ongoing	N/A
codeine phosphate	DIMETAPP-C	Pfizer Consumer HealthCare a Division of Pfizer Canada Inc.	2019-01-15	2019-01-15	Ongoing	N/A
codeine phosphate	FIORINAL-C ½	Aralez Pharmaceuticals Canada Inc.	2019-01-15	2019-01-14	Ongoing	N/A
codeine phosphate	FIORINAL-C ¼	Aralez Pharmaceuticals Canada Inc.	2019-01-15	2019-01-14	Ongoing	N/A
codeine phosphate	LINCTUS CODEINE BLANC	Laboratoire Atlas Inc.	2019-01-15	Pending	Pending	N/A

codeine phosphate	METHOXISAL-C ½	Teva Canada Limited	2019-01-15	2019-01-15	Ongoing	N/A
codeine phosphate	METHOXISAL-C ¼	Teva Canada Limited	2019-01-15	2019-01-15	Ongoing	N/A
codeine phosphate	pms-ACETAMINOPHEN WITH CODEINE ELIXIR	Pharmascience Inc.	2019-01-15	2019-01-15	Ongoing	N/A
codeine phosphate	PROCET-30	Pro Doc Limitée	2019-01-15	2019-01-15	Ongoing	N/A
codeine phosphate	RATIO-LENOLTEC NO 2	Teva Canada Limited	2019-01-15	2019-01-15	Ongoing	N/A
codeine phosphate	RATIO-LENOLTEC NO 3	Teva Canada Limited	2019-01-15	2019-01-15	Ongoing	N/A
codeine phosphate	RATIO-LENOLTEC NO 4	Teva Canada Limited	2019-01-15	2019-01-15	Ongoing	N/A
codeine phosphate	ROBAXISAL C ½	Pfizer Consumer HealthCare a Division of Pfizer Canada Inc.	2019-01-15	2019-01-15	Ongoing	N/A
codeine phosphate	ROBAXISAL C ¼	Pfizer Consumer HealthCare a Division of Pfizer Canada Inc.	2019-01-15	2019-01-15	Ongoing	N/A
codeine	ROBITUSSIN AC	Pfizer	2019-01-15	2019-01-15	Ongoing	N/A

phosphate		Consumer HealthCare a Division of Pfizer Canada Inc.				
codeine phosphate	TEVA-CODEINE	Teva Canada Limited	2019-01-15	2019-01-15	Ongoing	N/A
codeine phosphate	TEVA-COTRIDIN	Teva Canada Limited	2019-01-15	2019-01-15	Ongoing	N/A
codeine phosphate	TEVA-COTRIDIN EXPECTORANT	Teva Canada Limited	2019-01-15	2019-01-15	Ongoing	N/A
codeine phosphate	TEVA-EMTEC-30	Teva Canada Limited	2019-01-15	2019-01-15	Ongoing	N/A
codeine phosphate	TEVA-TECNAL C ½	Teva Canada Limited	2019-01-15	2019-01-15	Ongoing	N/A
codeine phosphate	TEVA-TECNAL C ¼	Teva Canada Limited	2019-01-15	2019-01-15	Ongoing	N/A
codeine phosphate	TRIANAL C½	Laboratoire Riva Inc.	2019-01-15	2019-01-14	Ongoing	N/A
codeine phosphate	TRIATEC-30	Laboratoire Riva Inc.	2019-01-15	2019-01-14	Ongoing	N/A
codeine phosphate	TYLENOL WITH CODEINE NO. 2 - TAB	Janssen Inc.	2019-01-15	2019-01-11	Ongoing	N/A
codeine phosphate	TYLENOL WITH CODEINE NO. 3 – TAB	Janssen Inc.	2019-01-15	2019-01-11	Ongoing	N/A
codeine phosphate	TYLENOL WITH CODEINE NO. 4	Janssen Inc.	2019-01-15	2019-01-11	Ongoing	N/A
codeine phosphate	CODEINE 15	Laboratoire Riva Inc.	2019-01-15	2019-01-14	Ongoing	N/A

fentanyl	FENTANYL PATCH	Pro Doc Limitée	2018-11-14	2018-11-15	Ongoing	N/A
fentanyl	PMS-FENTANYL MTX	Pharmascience Inc.	2018-11-14	2018-11-13	Ongoing	N/A
fentanyl	RAN-FENTANYL MATRIX PATCH	Ranbaxy Pharmaceuticals Canada Inc.	2018-11-14	2018-11-13	Ongoing	N/A
fentanyl	SANDOZ FENTANYL PATCH	Sandoz Canada Incorporated	2018-11-14	2018-11-15	Ongoing	N/A
fentanyl	TEVA-FENTANYL	Teva Canada Limited	2018-11-14	2018-11-14	Ongoing	N/A
fentanyl citrate	FENTANYL CITRATE INJECTION SDZ	Sandoz Canada Incorporated	2018-11-14	2018-11-15	Ongoing	N/A
fentanyl citrate	FENTANYL CITRATE INJECTION USP	Sandoz Canada Incorporated	2018-11-14	2018-11-15	Ongoing	N/A
fentanyl citrate	FENTANYL CITRATE INJECTION USP	Teligent OU	2018-11-14	2018-11-14	Ongoing	N/A
fentanyl citrate	FENTORA	Teva Canada Limited	2018-11-14	2018-11-14	Ongoing	N/A
hydrocodone	TUSSIONEX	Sanofi-Aventis Canada Inc.	2018-11-14	2018-11-13	Ongoing	N/A
hydrocodone bitartrate	DALMACOL	Laboratoire Riva Inc.	2018-11-14	2018-11-14	Ongoing	N/A
hydrocodone bitartrate	DIMETANE EXPECTORANT DC	Pfizer Consumer	2018-11-14	2018-11-13	Ongoing	N/A

		Healthcare, a Division of Pfizer Canada Inc.				
hydrocodone bitartrate	NOVAHISTEX DH	Sanofi-Aventis Canada Inc.	2018-11-14	2018-11-13	Ongoing	N/A
hydrocodone bitartrate	NOVAHISTINE DH	Sanofi-Aventis Canada Inc.	2018-11-14	2018-11-13	Ongoing	N/A
hydrocodone bitartrate	PDP-HYDROCODONE	Pendopharm Division of Pharmascience Inc.	2018-11-14	2018-11-13	Ongoing	N/A
hydromorphone hydrochloride	PMS HYDROMORPHONE SUPPOSITOIRE 3MG	Pharmascience Inc.	2018-11-14	2018-11-13	Ongoing	N/A
hydromorphone hydrochloride	HYDROMORPH CONTIN-CONTROLLED RELEASE CAP - 30MG	Purdue Pharma	2018-11-14	2018-11-14	Ongoing	N/A
hydromorphone hydrochloride	APO-HYDROMORPHONE	Apotex Inc.	2018-11-14	2018-11-15	Ongoing	N/A
hydromorphone hydrochloride	APO-HYDROMORPHONE CR	Apotex Inc.	2018-11-14	2018-11-15	Ongoing	N/A
hydromorphone hydrochloride	DILAUDID	Purdue Pharma	2018-11-14	2018-11-14	Ongoing	N/A
hydromorphone hydrochloride	DILAUDID HP	Purdue Pharma	2018-11-14	2018-11-14	Ongoing	N/A
hydromorphone	PMS	Pharmascience	2018-11-14	2018-11-13	Ongoing	N/A

hydrochloride	HYDROMORPHONE SIROP 1MG/ML	Inc.				
hydromorphone hydrochloride	PMS-HYDROMORPHONE TAB 1MG	Pharmascience Inc.	2018-11-14	2018-11-13	Ongoing	N/A
hydromorphone hydrochloride	PMS-HYDROMORPHONE TAB 2MG	Pharmascience Inc.	2018-11-14	2018-11-13	Ongoing	N/A
hydromorphone hydrochloride	PMS-HYDROMORPHONE TAB 4MG	Pharmascience Inc.	2018-11-14	2018-11-13	Ongoing	N/A
hydromorphone hydrochloride	PMS-HYDROMORPHONE TAB 8MG	Pharmascience Inc.	2018-11-14	2018-11-13	Ongoing	N/A
hydromorphone hydrochloride	HYDROMORPH CONTIN	Purdue Pharma	2018-11-14	2018-11-14	Ongoing	N/A
hydromorphone hydrochloride	HYDROMORPH CONTIN-CONTROLLED RELEASE CAP - 12MG	Purdue Pharma	2018-11-14	2018-11-14	Ongoing	N/A
hydromorphone hydrochloride	HYDROMORPH CONTIN-CONTROLLED RELEASE CAP - 18MG	Purdue Pharma	2018-11-14	2018-11-14	Ongoing	N/A
hydromorphone hydrochloride	HYDROMORPH CONTIN-CONTROLLED RELEASE CAP - 24MG	Purdue Pharma	2018-11-14	2018-11-14	Ongoing	N/A
hydromorphone hydrochloride	HYDROMORPH CONTIN-CONTROLLED RELEASE CAP - 3MG	Purdue Pharma	2018-11-14	2018-11-14	Ongoing	N/A
hydromorphone	HYDROMORPH	Purdue Pharma	2018-11-14	2018-11-14	Ongoing	N/A

hydrochloride	CONTIN-CONTROLLED RELEASE CAP - 6MG					
hydromorphone hydrochloride	HYDROMORPHONE HP 10	Sandoz Canada Incorporated	2018-11-14	2018-11-14	Ongoing	N/A
hydromorphone hydrochloride	HYDROMORPHONE HP 20	Sandoz Canada Incorporated	2018-11-14	2018-11-14	Ongoing	N/A
hydromorphone hydrochloride	HYDROMORPHONE HP 50	Sandoz Canada Incorporated	2018-11-14	2018-11-14	Ongoing	N/A
hydromorphone hydrochloride	HYDROMORPHONE HP FORTE NJECTION	Sandoz Canada Incorporated	2018-11-14	2018-11-14	Ongoing	N/A
hydromorphone hydrochloride	HYDROMORPHONE HYDROCHLORIDE INJECTION USP	Sandoz Canada Incorporated	2018-11-14	2018-11-14	Ongoing	N/A
hydromorphone hydrochloride	HYDROMORPHONE HYDROCHLORIDE INJECTION HP 50	Sterimax Inc.	2018-11-14	2018-11-19	Ongoing	N/A
hydromorphone hydrochloride	HYDROMORPHONE HYDROCHLORIDE INJECTION HP FORTE	Sterimax Inc.	2018-11-14	2018-11-19	Ongoing	N/A
hydromorphone hydrochloride	HYDROMORPHONE HYDROCHLORIDE INJECTION	Sterimax Inc.	2018-11-14	2018-11-19	Ongoing	N/A
hydromorphone hydrochloride	HYDROMORPHONE HYDROCHLORIDE INJECTION HP 10	Sterimax Inc.	2018-11-14	2018-11-19	Ongoing	N/A
hydromorphone hydrochloride	HYDROMORPHONE HYDROCHLORIDE	Pfizer Canada Inc.	2018-11-14	2018-11-13	Ongoing	N/A

	INJECTION USP					
hydromorphone hydrochloride	TEVA-HYDROMORPHONE	Teva Canada Limited	2018-11-14	2018-11-14	Ongoing	N/A
methadone hydrochloride	METADOL	Paladin Labs Inc.	2018-11-14	2018-11-13	Ongoing	N/A
methadone hydrochloride	METADOL-D	Paladin Labs Inc.	2018-11-14	2018-11-13	Ongoing	N/A
methadone hydrochloride	METHADOSE	Mallinckrodt Canada ULC	2018-11-14	2018-11-14	Ongoing	N/A
morphine hydrochloride	DOLORAL 1	Laboratoire Atlas Inc.	2018-11-14	2018-11-16	Ongoing	N/A
morphine hydrochloride	DOLORAL 5 SIROP 5MG/ML	Laboratoire Atlas Inc.	2018-11-14	2018-11-16	Ongoing	N/A
morphine sulfate	KADIAN	BGP Pharma ULC	2018-11-14	2018-11-14	Ongoing	N/A
morphine sulfate	M-EDIAT	Ethypharm Inc.	2018-11-14	2018-11-14	Ongoing	N/A
morphine sulfate	M-ESLON	Ethypharm Inc.	2018-11-14	2018-11-14	Ongoing	N/A
morphine sulfate	MORPHINE HP 50 - 50MG/ML USP	Sandoz Canada Incorporated	2018-11-14	2018-11-14	Ongoing	N/A
morphine sulfate	MORPHINE LP EPIDURAL	Sandoz Canada Incorporated	2018-11-14	2018-11-14	Ongoing	N/A
morphine sulfate	MORPHINE SR	Sanis Health Inc.	2018-11-14	2018-11-16	Ongoing	N/A

morphine sulfate	MS CONTIN SRT 100MG	Purdue Pharma	2018-11-14	2018-11-14	Ongoing	N/A
morphine sulfate	MS CONTIN SRT 15MG	Purdue Pharma	2018-11-14	2018-11-14	Ongoing	N/A
morphine sulfate	MS CONTIN SRT 200MG	Purdue Pharma	2018-11-14	2018-11-14	Ongoing	N/A
morphine sulfate	MS CONTIN SRT 30MG	Purdue Pharma	2018-11-14	2018-11-14	Ongoing	N/A
morphine sulfate	MS CONTIN SRT 60MG	Purdue Pharma	2018-11-14	2018-11-14	Ongoing	N/A
morphine sulfate	MORPHINE SULFATE INJ 10MG/ML USP	Sandoz Canada Incorporated	2018-11-14	2018-11-14	Ongoing	N/A
morphine sulfate morphine sulfate	MORPHINE SULFATE INJ 15MG/ML USP	Sandoz Canada Incorporated	2018-11-14	2018-11-14	Ongoing	N/A
morphine sulfate	MORPHINE SULFATE INJECTION USP	Sandoz Canada Incorporated	2018-11-14	2018-11-14	Ongoing	N/A
morphine sulfate	STATEX DPS 50MG/ML	Paladin Labs Inc.	2018-11-14	2018-11-13	Ongoing	N/A
morphine sulfate	STATEX DROPS 20MG/ML	Paladin Labs Inc.	2018-11-14	2018-11-13	Ongoing	N/A
morphine sulfate	STATEX SUPPOSITORIES 20MG	Paladin Labs Inc.	2018-11-14	2018-11-13	Ongoing	N/A
morphine	STATEX SUPPOSITORIES	Paladin Labs Inc.	2018-11-14	2018-11-13	Ongoing	N/A

sulfate	30MG					
morphine sulfate	STATEX SUPPOSITORIES 5MG	Paladin Labs Inc.	2018-11-14	2018-11-13	Ongoing	N/A
morphine sulfate	STATEX SYRUP 10MG/ML	Paladin Labs Inc.	2018-11-14	2018-11-13	Ongoing	N/A
morphine sulfate	STATEX SYRUP 1MG/ML	Paladin Labs Inc.	2018-11-14	2018-11-13	Ongoing	N/A
morphine sulfate	STATEX SYRUP 5MG/ML	Paladin Labs Inc.	2018-11-14	2018-11-13	Ongoing	N/A
morphine sulfate	STATEX TAB 10MG	Paladin Labs Inc.	2018-11-14	2018-11-13	Ongoing	N/A
morphine sulfate	STATEX TAB 5MG	Paladin Labs Inc.	2018-11-14	2018-11-13	Ongoing	N/A
morphine sulfate	STATEX TABLET 25MG	Paladin Labs Inc.	2018-11-14	2018-11-13	Ongoing	N/A
morphine sulfate	STATEX TABLETS 50MG	Paladin Labs Inc.	2018-11-14	2018-11-13	Ongoing	N/A
morphine sulfate	STATEX-SUPPOSITORIES 10MG	Paladin Labs Inc.	2018-11-14	2018-11-13	Ongoing	N/A
morphine sulfate	MORPHINE SULFATE INJECTION USP	ICU Medical Canada Inc.	2018-11-14	2018-11-13	Ongoing	N/A
morphine sulfate	MORPHINE SULFATE INJECTION BP	Sterimax Inc.	2018-11-14	2018-11-19	Ongoing	N/A
morphine sulfate	MORPHINE SULFATE INJECTION SDZ	Sandoz Canada Incorporated	2018-11-14	2018-11-14	Ongoing	N/A

morphine sulfate	MS.IR	Purdue Pharma	2018-11-14	2018-11-14	Ongoing	N/A
morphine sulfate	SANDOZ MORPHINE SR	Sandoz Canada Incorporated	2018-11-14	2018-11-14	Ongoing	N/A
morphine sulfate	TEVA-MORPHINE SR	Teva Canada Limited	2018-11-14	2018-11-14	Ongoing	N/A
normethadone hydrochloride	COPHYLAC	Valeant Canada LP/Valeant Canada S.E.C.	2018-11-14	2018-11-13	Ongoing	N/A
opium	SANDOZ OPIUM & BELLADONNA	Sandoz Canada Incorporated	2018-11-14	2018-11-14	Ongoing	N/A
oxycodone hydrochloride	ACT OXYCODONE CR	Actavis Pharma Company	2018-11-14	2018-11-14	Ongoing	N/A
oxycodone hydrochloride	APO-OXYCODONE/ACET	Apotex Inc.	2018-11-14	2018-11-16	Ongoing	N/A
oxycodone hydrochloride	APO-OXYCODONE CR	Apotex Inc.	2018-11-14	2018-11-15	Ongoing	N/A
oxycodone hydrochloride	OXY.IR	Purdue Pharma	2018-11-14	2018-11-13	Ongoing	N/A
oxycodone hydrochloride	OXYCODONE	Pro Doc Limitée	2018-11-14	2018-11-15	Ongoing	N/A
oxycodone hydrochloride	SUPEUDOL	Sandoz Canada Incorporated	2018-11-14	2018-11-14	Ongoing	N/A
oxycodone	SUPEUDOL 10	Sandoz Canada	2018-11-14	2018-11-14	Ongoing	N/A

hydrochloride		Incorporated				
oxycodone hydrochloride	SUPEUDOL 10	Sandoz Canada Incorporated	2018-11-14	2018-11-14	Ongoing	N/A
oxycodone hydrochloride	SUPEUDOL 20	Sandoz Canada Incorporated	2018-11-14	2018-11-14	Ongoing	N/A
oxycodone hydrochloride	SUPEUDOL 5	Sandoz Canada Incorporated	2018-11-14	2018-11-14	Ongoing	N/A
oxycodone hydrochloride	OXYCODONE-ACET	Pro Doc Limitée	2018-11-14	2018-11-15	Ongoing	N/A
oxycodone hydrochloride	OXYCODONE/ ACET	Sanis Health Inc.	2018-11-14	2018-11-16	Ongoing	N/A
oxycodone hydrochloride	OxyNEO	Purdue Pharma	2018-11-14	2018-11-13	Ongoing	N/A
oxycodone hydrochloride	PMS-OXYCODONE	Pharmascience Inc.	2018-11-14	2018-11-13	Ongoing	N/A
oxycodone hydrochloride	PMS-OXYCODONE CR	Pharmascience Inc.	2018-11-14	2018-11-13	Ongoing	N/A
oxycodone hydrochloride	RIVACOCET	Laboratoire Riva Inc.	2018-11-14	2018-11-14	Ongoing	N/A
oxycodone hydrochloride	SANDOZ OXYCODONE/ ACETAMINOPHEN	Sandoz Canada Incorporated	2018-11-14	2018-11-14	Ongoing	N/A
oxycodone hydrochloride	TARGIN	Purdue Pharma	2018-11-14	2018-11-14	Ongoing	N/A

oxycodone hydrochloride	TEVA-OXYCOCET	Teva Canada Limited	2018-11-14	2018-11-14	Ongoing	N/A
oxycodone hydrochloride	TEVA-OXYCODAN	Teva Canada Limited	2018-11-14	2018-11-14	Ongoing	N/A
tramadol hydrochloride	APO-TRAMADOL/ACET	Apotex Inc.	2019-01-15	2019-01-14	Ongoing	N/A
tramadol hydrochloride	AURO-TRAMADOL/ ACETAMINOPHEN	Auro Pharma Inc.	2019-01-15	2019-01-14	Ongoing	N/A
tramadol hydrochloride	AURO-TRAMADOL	Auro Pharma Inc.	2019-01-15	2019-01-14	Ongoing	N/A
tramadol hydrochloride	DURELA	Cipher Pharmaceuticals Inc.	2019-01-15	2019-01-15	Ongoing	N/A
tramadol hydrochloride	IPG-TRAMADOL/ ACET	Marcan Pharmaceuticals Inc.	2019-01-15	2019-01-14	Ongoing	N/A
tramadol hydrochloride	JAMP-ACET-TRAMADOL	JAMP Pharma Corporation	2019-01-15	2019-01-14	Ongoing	N/A
tramadol hydrochloride	AG-ACET-TRAMADOL	ANGITA PHARMA INC.	2019-01-15	2019-01-16	Ongoing	N/A
tramadol hydrochloride	MAR-TRAMADOL	Marcan Pharmaceuticals Inc.	2019-01-15	2019-01-14	Ongoing	N/A
tramadol hydrochloride	MAR-TRAMADOL/ ACET	Marcan Pharmaceuticals Inc.	2019-01-15	2019-01-14	Ongoing	N/A
tramadol hydrochloride	MINT-TRAMADOL/ ACET	Mint Pharmaceuticals	2019-01-15	Pending	Pending	N/A

		Inc				
tramadol hydrochloride	NRA-TRAMADOL/ACET	Nora Pharma Inc.	2019-01-15	Pending	Pending	N/A
tramadol hydrochloride	pms-TRAMADOL-ACET	Pharmascience Inc.	2019-01-15	2019-01-15	Ongoing	N/A
tramadol hydrochloride	PRIVA-TRAMADOL/ACET	Pharmapar Inc.	2019-01-15	2019-01-14	Ongoing	N/A
tramadol hydrochloride	RALIVIA	Valeant Canada LP/Valeant CANADA S.E.C.	2019-01-15	2019-01-14	Ongoing	N/A
tramadol hydrochloride	RAN-TRAMADOL/ACET	Ranbaxy Pharmaceuticals Canada Inc.	2019-01-15	2019-01-14	Ongoing	N/A
tramadol hydrochloride	TARO-TRAMADOL ER	Taro Pharmaceuticals Inc.	2019-01-15	2019-01-15	Ongoing	N/A
tramadol hydrochloride	TEVA-TRAMADOL/ ACETAMINOPHEN	Teva Canada Limited	2019-01-15	2019-01-15	Ongoing	N/A
tramadol hydrochloride	TRAMACET	Janssen Inc.	2019-01-15	2019-01-11	Ongoing	N/A
tramadol hydrochloride	TRAMADOL	AA Pharma Inc.	2019-01-15	2019-01-14	Ongoing	N/A
tramadol hydrochloride	TRAMADOL/ACET	Sanis Health Inc.	2019-01-15	2019-01-14	Ongoing	N/A
tramadol hydrochloride	TRAMADOL / ACETAMINOPHEN	Sivem Pharmaceuticals ULC	2019-01-15	Pending	Pending	N/A
tramadol hydrochloride	TRAMADOL-ACET	Pro Doc Limitée	2019-01-15	2019-01-15	Ongoing	N/A
tramadol hydrochloride	TRIDURAL	Paladin Labs Inc.	2019-01-15	2019-01-14	Ongoing	N/A
tramadol	ULTRAM	Janssen Inc.	2019-01-15	2019-01-11	Ongoing	N/A

hydrochloride						
tramadol hydrochloride	ZYTRAM XL	Purdue Pharma	2019-01-15	2019-01-15	Ongoing	N/A

N/A: Not applicable at this time

Prescription **Tracker Example**
The idea is that this could be posted as a link on the Compliance and Enforcement.

Prescription Opioids Risk Management Plan Tracker

As part of Health Canada's ongoing commitment to openness and transparency, the Department is publishing information regarding the status of risk management plans for prescription opioids.

The potential for harm associated with opioid drugs and the growing related public health crisis is the subject of increasing concern for Canadians and other levels of government in Canada. Health Canada has identified the need for strengthened post-market oversight of opioids to help monitor and mitigate their risks. As one part of this initiative, the Department has mandated that manufacturers of Class B opioids submit risk management plans with a focus on opioid-related harms.[1]

A risk management plan is a living document developed by the sponsor or authorization holder of a drug. It consists of a set of activities and interventions designed to monitor, quantify, characterize and mitigate risks relating to the product. It may also include a means for assessing the effectiveness of any interventions.

This tracker provides a snapshot of the submission and review of mandated risk management plans for prescription opioid products.

How the Tracker Works

- The tracker highlights when manufacturers have submitted mandated opioid-specific RMPs to Health Canada; and the status of Health Canada's review of these documents. It also indicates situations in which manufacturers have not submitted mandated RMPs within required deadlines.
- Even if a manufacturer is listed on the tracker as not having submitted an RMP, it doesn't necessarily mean there is an immediate risk to the health of Canadians.
- Continued failure to comply with the Terms and Conditions is considered a contravention of section 21.7 of the *Food and Drugs Act* which could result in more serious compliance and enforcement action under that Act. Compliance and

---

[1] Link to CG2: http://gazette.gc.ca/rp-pr/p2/2018/2018-05-02/html/sor-dors77-eng.html

enforcement outcomes will be determined and listed in accordance with a risk-based approach, aligned with departmental policies, including compliance promotion activities.

- Health Canada will continue to take action to manage risks identified to the health of Canadians.
- This tracker is updated regularly.
- Once an item is listed in the closed section, it indicates that the RMP was found to be satisfactory and while Health Canada may continue to update this document in collaboration with the manufacturer, there will be no more updates regarding this item.
- Last update:

Open Items

Active ingredient	Brand name (DINs)	Manufacturer	Deadline for RMP submission	Date RMP received by Health Canada	Status of Health Canada's review	Additional comments
						(hyperlink to any relevant posted summary of review/recommendations)

Closed Items

Active ingredient	Brand name (DINs)	Manufacturer	Deadline for RMP submission	Date RMP received by Health Canada	Status of Health Canada's review	Additional comments
						(hyperlink to any relevant posted summary of review/recommendations)

Dear Class B Opioid Market Authorization Holder,

The Government of Canada continues to take steps to address the opioid crisis. Health Canada is considering further restricting the marketing and advertising of opioids by proposing additional Terms and Conditions (Ts and Cs) for prescription opioid-containing products.

The proposed additional Ts and Cs serve to further restrict the marketing and advertising of opioids **to only statements that have been authorized by Health Canada in the Product Monograph (PM).**

Specifically, **only information contained in the PM would be included in all advertising materials for Class B opioids to health care professionals and must be presented *verbatim***. The advertising materials would also have to include the following elements in order to meet the fair balance requirements, stated *verbatim*:

- Indication:
  - The complete indication statement must appear at least once in the piece.

- Contraindication:
  - Each contraindication must be listed as a separate bullet.

- Most serious warnings and precautions:
  - The warnings and precautions which are emphasized in the Terms of Market Authorization (TMA; bolded, underlined, CAPLOCKED, etc.) must begin with a bolded topic.

- Other relevant warnings and precautions:
  - The rest of the warnings and precautions related to promoted indication(s) must appear as a bulleted list and may include the description.

- For more information:
  - A direct link to the TMA must be provided and the reader must be directed to seek more information about adverse events, drug interactions and dosing.

Preclearance of advertising materials will continue to be required to help implement the above proposed restrictions.

By way of this letter and the Notice to Stakeholders, Health Canada is informing affected Market Authorization Holders (MAHs) of their opportunity to provide comments on the proposed approach.

MAHs can provide their comments in writing by April 1, 2019, to the Marketed Health Products Directorate, Health Products and Food Branch, Address Locator: 1906B, Ottawa, Ontario, K1A 0K9, Department of Health. Comments can also be sent by email to MHPD_DPSC-Advertising_Reg_Publicite@hc-sc.gc.ca.

Health Canada plans to notify individual MAHs of the additional Ts and Cs by end of April 2019 with the expectation that **all new advertising materials developed as of June 2019 will be required to comply with the additional Ts and Cs.** Beginning in January 2020, previously existing advertising materials non-compliant with the additional Ts and Cs will no longer be permitted to be in circulation.

Thank you,

Rhonda Kropp, DG, MHPD

Notice to Stakeholders: Further Restrictions to the Marketing and Advertising of Opioids

Health Canada is seeking input from Market Authorization Holders (MAHs) on proposed additional Terms and Conditions (Ts and Cs) for Class B opioid products. Building on the Ts and Cs imposed in October 2018 by the Minister of Health, which included mandatory preclearance of advertising materials, the proposed additions would serve to further restrict the marketing and advertising of opioids under the authority of section C.01.014.21 of the *Food and Drug Regulations*.

Background

Prescriptions written by health care professionals are a common source of opioids in Canada. Health care professionals receive information from a variety of sources to inform their prescribing decisions and advice to patients, including from the pharmaceutical industry. While there is value in the pharmaceutical industry conveying educational and scientific information about a health product, evidence suggests that the marketing and advertising of opioids may be contributing to increased sales and availability of prescription opioids. It is also noteworthy that Canada has one of the highest prescription rates of opioids in the world.

In May 2018, regulations were introduced which gave the Minister of Health the authority to impose Ts and Cs on opioid products. In October 2018, through these Ts and Cs, Health Canada advised MAHs that they must provide Health Canada with Canadian Specific Opioid targeted Risk Management Plans (CSO-tRMP) for their Class B opioid products. As part of this requirement, MAHs must submit all materials regarding opioid products they intend to provide to health care professionals for preclearance to an external advertising preclearance agency (APA) recognized by Health Canada. The APA assesses whether the materials are consistent with the terms of the product's market authorization and determine whether any statements are false or misleading.

In June 2018, Health Canada published a Notice of Intent to restrict the marketing and advertising of opioids whereby it consulted on whether and how to proceed with the restriction on opioid marketing and advertising. A summary of the feedback received from health care professionals and associations, patients, academics, and pharmaceutical industry representatives was published in December 2018. Respondents were generally supportive of restrictions on marketing and advertising of opioids, and noted the importance of having access to unbiased educational information to support appropriate opioid prescribing.

Proposed Further Restrictions

Recognizing the current ongoing opioid crisis, Health Canada is considering further restricting the marketing and advertising of opioids by proposing additional Ts and Cs for prescription opioid-containing products. **The proposed additional Ts and Cs would restrict all advertising materials of Class B opioids provided to health care professionals to only statements that have been authorized by Health Canada in the Product Monograph (PM). Specifically, only information contained in the PM would be permitted in such advertising materials and would have to be presented *verbatim* while meeting fair balance requirements of benefits and risks.**

Importantly, the mandatory preclearance imposed through the October 2018 Ts and Cs would continue to apply. This proposed approach allows the pharmaceutical industry to convey educational and scientific information about a health product, while ensuring that information is evidence-based, unbiased, and accurate, and the benefits and risks of opioid products are conveyed in a balanced way.

Advertising of controlled substances, including Class B opioids, must be consistent with the *Food and Drugs Act*, the *Controlled Drugs and Substances Act*, and associated regulations.

Next steps

MAHs have until April 1, 2019 to submit comments in writing. Health Canada plans to notify individual MAHs of the additional Ts and Cs by end of April 2019 with the expectation that all advertising materials developed as of June 2019 would be required to comply with the new Ts and Cs.

Questions

Questions concerning the proposed additional restriction should be directed to MHPD_DPSC-Advertising_Reg_Publicite@hc-sc.gc.ca.

Health Canada | Santé Canada

Health Products and Food Branch | Direction générale des produits de santé et des aliments

Marketed Health Products Directorate
A.L. 1912C
OTTAWA, Ontario
K1A 0K9

File: 19-108953-521

Name
Title
Company name
Street address
CITY, Province
Postal code
Email:

Additional Terms and Conditions for Class B opioid(s)
Re: Brand-Name(s)

Dear Name:

This letter serves as a follow-up to the correspondence Health Canada sent you on March 11, 2019 proposing additional Terms and Conditions for Class B opioid products.

Though prescription opioids can help Canadians who need them to manage pain, over-prescription of opioids has contributed to Canada's current opioid crisis. In fact, Canada has one of the highest prescription rates of opioids in the world. The prescribing practices of health care professionals are informed by a variety of sources, including the pharmaceutical industry. While there is value in the pharmaceutical industry conveying educational and scientific information about a health product, evidence suggests that the marketing and advertising of opioids may be contributing to increased sales and availability of prescription opioids. There is also evidence to suggest that information on harm is less likely to be provided than information on health benefits. Given these concerns, the Government of Canada is taking additional steps to address the ongoing opioid crisis.

You will recall that, in June 2018, Heath Canada published its Notice of Intent (NOI) to Restrict the Marketing and Advertising of Opioids. Between August and November 2018, Health Canada sent manufacturers Terms and Conditions letters for Class B opioid products, which advised them that they must:

- provide Health Canada with Canadian Specific Opioid-targeted Risk Management Plans (CSO-tRMP) for their opioid products, including the requirement to

.../2

- submit all materials regarding opioid products they intend to provide to health care professionals for preclearance to an external advertising preclearance agency recognized by Health Canada.

In March 2019, Health Canada notified manufacturers of the proposal for additional Terms and Conditions to impose restrictions on the actual content of marketing and advertising of opioids. Health Canada has taken into consideration comments submitted by Market Authorization Holders (MAHs) when finalizing the additional requirements.

By way of this letter, and under authority of section C.01.014.21 of the *Food and Drug Regulations*[1], additional Terms and Conditions will be imposed as of June 1, 2019 on the following Drug Identification Number (DIN):

MEDICINAL INGREDIENT	BRAND NAME	DIN	STRENGTH	FORM	STATUS
X	X	X	X	X	X

1) Name of manufacturer must ensure that promotional materials disseminated to health care professionals contain only statements that have been authorized by Health Canada in the approved labelling[2], and that those statements be presented *verbatim*.

2) Name of manufacturer must also include the following elements in order for the promotional materials to achieve fair balance in terms of benefit / risk presentation,
    a. Indication:
        i. The complete indication statement must appear at least once in the piece.
    b. Contraindication:
        i. Each contraindication must be listed as a separate bullet.
    c. Most serious warnings and precautions:
        i. The warnings and precautions which are emphasized in the approved labelling[2] (bolded, underlined, CAPLOCKED, etc.) must begin with a bolded topic.
    d. Other relevant warnings and precautions:
        i. The rest of the warnings and precautions related to promoted indication(s) must appear as a bulleted list and may include the description.
    e. For more information:
        i. A direct link to the approved labelling[2] must be provided which would direct the reader to seek more information about adverse events, drug interactions and dosing.

-3-

[1] Regulations amending the *Food and Drug Regulations* (Opioids): http://gazette.gc.ca/rp-pr/p2/2018/2018-05-02/html/sor-dors77-eng.html
[2] Approved labelling includes the product monograph (PM), prescribing information (PI) and/or package label.

The additional Terms and Conditions do not apply to sell sheets and other communications which have the main purpose of providing information on product availability, changes in product appearance, supply disruptions and product discontinuation, provided they do not include therapeutic[3] or promotional messaging.

All new advertising materials developed as of June 1, 2019 will be required to comply with the additional Terms and Conditions. Beginning on January 1, 2020, previously existing advertising materials not in compliance with the additional Terms and Conditions will no longer be permitted to be in circulation.

Failure to comply with the additional Terms and Conditions set out above, as well as the original Terms and Conditions set out in our previous communication is a contravention of Section 21.7 of the *Food and Drugs Act.*[4] Compliance and enforcement measures may be taken if:

- statements included in advertising materials are not presented *verbatim* and/or do not meet the fair balance requirements, or
- Health Canada becomes aware of advertising that did not obtain preclearance and/or had not been submitted to Health Canada with proof of preclearance.

Thank you in advance for your cooperation in this matter.

Please confirm receipt of this letter by way of reply to MHPD_DPSC-Advertising_Reg_Publicite@hc-sc.gc.ca by May 15, 2019.

Sincerely,

Rhonda Kropp
Director General
Marketed Health Products Directorate

---

[3] A therapeutic message is one regarding the effectiveness and/or efficacy of a health product.
[4] *Food and Drugs Act*: http://laws-lois.justice.gc.ca/eng/acts/F-27/index.html

Health Canada / Santé Canada

Health Products and Food Branch / Direction générale des produits de santé et des aliments

Direction des produits de santé commercialisés
Indice de l'adresse 1912C
Ottawa (Ontario)
K1A 0K9

Dossier : 19-108953-521

Nom
Titre
Nom de l'entreprise
Adresse municipale
Ville (Province)
Code postal
Courriel :

Conditions supplémentaires sur un (des) opioïde(s) de catégorie B
Objet : Nom(s) de marque

Madame, Monsieur,

La présente lettre fait suite à la lettre que Santé Canada vous a envoyée le 11 mars 2019 proposant d'ajouter des conditions supplémentaires aux produits opioïdes de catégorie B.

Bien que les opioïdes d'ordonnance puissent aider les Canadiens qui en ont besoin à soulager des douleurs, la prescription excessive de ces produits a contribué à la crise des opioïdes que le Canada connaît actuellement. En effet, le Canada affiche l'un des taux de prescription d'opioïdes les plus élevés au monde. Les pratiques de prescription des professionnels de la santé sont éclairées par diverses sources, dont l'industrie pharmaceutique. Bien qu'il y ait un intérêt à ce que l'industrie pharmaceutique transmette des renseignements éducatifs et scientifiques sur un produit de santé, les données montrent que le marketing et la publicité des opioïdes peuvent contribuer à augmenter les ventes et la disponibilité des opioïdes sur ordonnance. Des données probantes indiquent également que les renseignements sur les méfaits sont moins susceptibles d'être communiqués que ceux sur les bienfaits pour la santé. Compte tenu de ces préoccupations, le gouvernement du Canada prend des mesures supplémentaires afin de s'attaquer à la crise des opioïdes.

Vous vous souviendrez qu'en juin 2018, Santé Canada a publié un Avis d'intention de restreindre le marketing et la publicité des opioïdes. Entre les mois d'août et de novembre 2018, Santé Canada a envoyé aux fabricants des lettres de conditions concernant les produits opioïdes de catégorie B, les informant qu'ils sont tenus de :

- fournir à Santé Canada des Plans canadiens ciblant la gestion des risques spécifiques

aux opioïdes (PCcGRSO) pour leurs produits opioïdes; y compris l'exigence de

- soumettre tout le matériel concernant les produits opioïdes qu'ils ont l'intention de fournir aux professionnels de la santé à un organisme externe de pré-approbation de la publicité reconnu par Santé Canada aux fins de pré-approbation.

En mars 2019, Santé Canada a informé les fabricants de la proposition d'ajouter des conditions supplémentaires qui imposeraient des restrictions sur le contenu même du marketing et de la publicité des opioïdes. Santé Canada a tenu compte des commentaires envoyés par les détenteurs d'une autorisation de mise sur le marché (DAMM) au moment de finaliser les exigences supplémentaires.

Par la présente lettre, et en vertu de l'article C.01.014.21 du *Règlement sur les aliments et drogues*[1], des conditions supplémentaires seront imposées à partir du 1 juin 2019 sur les numéros d'identification du médicament (DIN) suivants :

Ingrédient actif	Nom du produit	Numéro d'identification de médicament (DIN)	Concentration	Forme posologique	État actuel
X	X	X	X	X	X

1) Nom du fabricant doit s'assurer que le matériel promotionnel distribué aux professionnels de la santé ne contient que des énoncés qui ont été autorisés par Santé Canada dans l'étiquetage approuvé[2], et que ces énoncés sont présentés *textuellement*.

2) Nom du fabricant doit également inclure les éléments suivants pour que le matériel promotionnel affiche un juste équilibre en ce qui a trait à la présentation des risques et des bienfaits.
   a. Indication :
      i. La mention d'indication complète doit apparaître au moins une fois dans la pièce.
   b. Contre-indication :
      i. Chaque contre-indication doit être énumérée sous une puce distincte.
   c. Mises en garde et précautions les plus importantes :
      i. Les mises en garde et les précautions qui sont mises en évidence dans l'étiquetage approuvé[2] (en caractères gras, soulignés, EN LETTRES MAJUSCULES, etc.) doivent commencer par un énoncé en caractères gras.

[1] *Règlement modifiant le Règlement sur les aliments et drogues (opioïdes)* :
http://gazette.gc.ca/rp-pr/p2/2018/2018-05-02/html/sor-dors77-fra.html

[2] L'étiquetage approuvé comprend la monographie du produit, les renseignements d'ordonnance et/ou l'étiquette d'emballage.

d. Autres mises en garde et précautions pertinentes :
    i. Les autres mises en garde et précautions relatives aux indications faisant l'objet de la promotion doivent figurer dans une liste à puces et peuvent inclure la description.
e. Pour de plus amples renseignements :
    i. Un lien direct vers l'étiquetage[2] approuvé doit être fourni afin d'inviter le lecteur à obtenir plus de renseignements sur les effets indésirables, les interactions médicamenteuses et la posologie.

Les conditions supplémentaires ne s'appliquent pas aux fiches de vente et aux autres communications dont l'objectif principal est de fournir des renseignements sur la disponibilité du produit, les modifications à l'apparence du produit, les interruptions d'approvisionnement et l'abandon de la vente du produit, à condition qu'elles ne comprennent pas de messages thérapeutiques[3] ou promotionnels.

Tout nouveau matériel publicitaire élaboré à compter du 1er juin 2019 devra être conforme aux conditions supplémentaires. À partir du 1er janvier 2020, le matériel publicitaire existant non conforme aux conditions supplémentaires ne pourra plus être mis en circulation.

La non-conformité aux conditions supplémentaires énoncées ci-dessus, ainsi qu'aux conditions initiales énoncées dans notre précédente communication, constitue une infraction à l'article 21.7 de la *Loi sur les aliments et drogues*[4]. Des mesures de conformité et d'application de la loi peuvent être prises dans les cas suivants :

- les énoncés figurant dans le matériel publicitaire ne sont pas présentés *textuellement* et/ou ne satisfont pas aux exigences de juste équilibre; ou
- Santé Canada prend connaissance des publicités qui n'ont pas fait l'objet d'une pré-approbation et/ou qui ne lui ont pas été soumises avec des preuves de pré-approbation.

Je vous remercie d'avance de votre collaboration.

Veuillez accuser réception de la présente lettre en envoyant une réponse à MHPD DPSC-Advertising Reg Publicite@hc-sc.gc.ca d'ici le 15 mai 2019.

Cordialement,

Rhonda Kropp

[3] Un message thérapeutique est un message portant sur l'efficacité d'un produit de santé.
[4] *Loi sur les aliments et drogues* :
https://laws-lois.justice.gc.ca/fra/lois/F-27/index.html

Directrice générale
Direction des produits de santé commercialisés

Health Canada Santé Canada

News Release
For Immediate Release

Health Canada announces proposed additional restrictions on marketing of opioids
New measures will further limit advertising to health care professionals

March 11, 2019 Ottawa Health Canada

Canada continues to experience an opioid crisis, which is having devastating effects on the health and lives of many Canadians, their families and their communities across the country.

Today, the Honourable Ginette Petitpas Taylor, Minister of Health, announced that Health Canada is taking additional steps to further restrict the marketing and advertising of prescription opioids. Health Canada is proposing additional restrictions on the marketing and advertising of Class B opioid products provided to health care professionals. Class B opioids are equal to or stronger than morphine.

Under Health Canada's proposal, all promotional materials about Class B opioids provided to health care professionals, including print and electronic ads and pamphlets, must be limited to only statements that have been authorized by Health Canada in the Product Monograph. Statements would have to be presented verbatim, and convey the benefits and risks of opioids in a balanced way. Affected companies can comment on the additional restrictions before they are finalized in April 2019. The new requirements would take effect by June 2019.

The Minister also announced the launch of the Stop Illegal Marketing of Drugs and Devices online platform. This web-based platform aims to educate Canadians on the rules governing the advertising of Canadian health products. It also provides a quick and easy tool to file a complaint with Health Canada about promotional activities that may not be compliant with advertising rules.

Commented [DSM1]: Link to be provided, once available.

These measures build on already announced initiatives to address the pharmaceutical industry's opioid marketing and advertising practices. For example, as of October 2018, a warning sticker and a patient information handout must be provided with prescription opioids dispensed to Canadians at pharmacies or in doctors' offices. Also, an external advertising agency has to preclear all materials regarding opioid products that industry intends to provide to health care professionals.

Quotes

"I am deeply concerned by the ongoing opioid crisis, which is affecting Canadians from all walks of life. I recognize that advertising can influence the prescribing practices of health care professionals. Today's announcement will help us to ensure that health professionals are getting only factual information about these products, so that they can provide the best possible support to their patients."

The Honourable Ginette Petitpas Taylor
Minister of Health

Associated Links

Notice to Stakeholders
About opioids
Restricting the Marketing and Advertising of Opioids

Commented [DSM2]: Link to be provided, once available.

Canada

Health Canada Santé Canada

- 30 -

Contacts

Thierry Bélair
Office of Ginette Petitpas Taylor
Minister of Health
613-957-0200

Media Relations
Health Canada
613-957-2983
hc.media.sc@canada.ca

Public Inquiries:
613-957-2991
1-866-225-0709

Canada

Proposed content for web summary

What We Heard: Summary of Responses to the Notice of Intent to Restrict the Marketing and Advertising of Opioids

Draft: October 10, 2018

Introduction

Canadians are the second highest users per capita of prescription opioids in the world, and rates of opioid prescribing and opioid-related hospital visits and deaths have been increasing rapidly. Prescriptions written by health care professionals are a common source of opioids in Canada. Health care professionals receive information from a variety of sources to inform their prescribing decisions and advice to patients, including from the pharmaceutical industry.

Recognizing the influence industry marketing and advertising activities have on prescribing practices, Health Canada released a Notice of Intent (Notice) to restrict the marketing and advertising of opioids to health care professionals. The Notice was open for a 30 day consultation period from June 19, 2018 to July 18, 2018.

This What We Heard Report contains a summary of the feedback about Health Canada's Notice, reflecting the views of individuals and groups who chose to respond. As such, this summary cannot be accurately generalized to the wider Canadian population and does not necessarily reflect the Government of Canada's position.

Who provided feedback to Health Canada?

The Notice was open for comments from all interested stakeholders. In total, 42 responses were received, including from health care professionals, patients, academics, and the pharmaceutical industry. The breakdown of responses by stakeholder group is shown in Figure 1.

Figure 1: Responses to the Notice of Intent to restrict the marketing and advertising of opioids, by stakeholder group

Respondent Type	Total
**Health Care Professionals and Health Care Professional Associations**	21
**Patients and Patient Organizations**	11
**Industry and Industry Associations**	6
**Provincial Government Officials**	3
**Academia**	1*
**Total count**	42

*Note: This response was submitted on behalf of multiple individuals.

What did stakeholders say?

Summary of Key Themes

Respondents shared their views on experiences related to the **marketing and advertising** of drugs, the current system in place to review marketing and advertising material as well as impacts of drug marketing material on prescribing practices.

Respondents were supportive of federal action to address Canada's opioid crisis, and provided various suggestions for actions that could help reduce the harms related to opioids. Specifically related to the marketing and advertising of opioids, respondents were generally supportive of restrictions and noted the importance of **unbiased educational information** to encourage appropriate prescribing. Many respondents noted that the measures taken to restrict the advertising and marketing of opioids should not inadvertently limit the availability of evidence-based information.

Many respondents also underscored the importance of ensuring that individuals with acute or chronic pain, and those with opioid use disorder, continue to have **access to the treatments** they need.

Respondents provided suggestions for a **range of actions** to achieve better regulatory oversight of marketing and advertising of opioids, such as mandatory transparency and disclosure requirements. Additionally, some respondents noted that the factors contributing to the opioid crisis are complex, and therefore, actions are required beyond addressing the marketing and advertising of prescription opioids.

Summary of Feedback

Health Care Professionals and Health Care Professional Associations

Respondents highlighted that accurate, factual information and educational activities are essential for supporting health care professionals in their roles as they provide good quality patient care. Health professional associations support taking further action, though some respondents expressed concern that restrictions on marketing and advertising of opioids could lead to reduced access to opioids for pain treatment and opioid use disorder treatment. In summary:

Marketing and Advertising

- Reflecting on personal experience, some respondents described industry's information and sponsored events for health care professionals as biased and containing promotional content. Other respondents indicated that the information and learning opportunities provided by industry contained valuable information about effective pain management.
- Advertising that omits or de-emphasizes information on a drug's risks or adverse effects is potentially harmful.

Unbiased Educational Information

- Health care professionals have a role in minimizing industry's bias in continuing medical education by following existing standards, guidelines and codes of ethics related to their relationships with the pharmaceutical industry.
- Educational programs and training are important, and a distinction should be made between activities that are promotional and those that support safe and appropriate prescribing.
- Treatments for opioid use disorder are a pressing need, so restrictions to advertising and marketing should not reduce health care professionals' access to educational information and training in this area, reduce the development of new treatments, or prevent the pharmaceutical industry from notifying the public of their products.
- Some respondents indicated educational materials or programs developed or delivered by industry should be reviewed by a third party to ensure they are factual and accurate, while others responded that educational materials should not be developed by pharmaceutical manufacturers. The materials should communicate both benefits and risks, and include details that help in decision-making, such as non-opioid treatments available, guidelines for use, efficacy of treatment and cost effectiveness.
- There is a need to address challenges in funding for continuing medical education for pain management using both pharmaceutical and non-pharmaceutical options.

Access to Treatments

- Restricting marketing of opioids could have negative impacts for patients living with pain if they do not have access to the most appropriate treatments for pain management, due to less information being shared with health care professionals about the risks of drug interactions, new technologies and new drugs.

Commented [PS1]: I find this section repetitive versus points in the previous section.either make this more specific, or remove.

Commented [MG2]: Bullet reworded to highlight the respondents' comments about *access* to treatments.

Range of Actions

- Illegal opioids are a large contributing factor to the opioid crisis. Therefore, efforts to restrict marketing and advertising of prescription opioids may have limited effectiveness in addressing the current crisis.
- There is a need to restrict promotional activities related to opioids, as well as for all prescription and non-prescription drugs. There should be strict penalties for non-compliance, including criminal charges if appropriate.
- Restrictions should apply to all forms of industry communication including verbal, written, electronic, social media communications and personal visits from sales representatives to health care professionals.
- Restrictions could extend to advocacy groups that receive industry funding.
- The current, voluntary pre-clearance system for drug advertising by agencies such as the Pharmaceutical Advertising Advisory Board needs to be more robust and systematic and should define opioid-specific advertising criteria.

- There is support for greater transparency, disclosure and mandatory reporting of marketing activities, funding and incentives provided by pharmaceutical companies to health care professionals, associations and patient groups.
- Physicians should be prohibited from receiving industry payments, including for providing educational lectures to other health care professionals. Health care professionals should be compensated for their time as speakers, but the amount of monetary compensation could be limited.
- Industry could be permitted to fund educational programs if the activity was certified by a regulatory body. Similarly, any industry funding to organizations should be used for unbiased learning events.
- New opioid products could be approved with conditions that would, for example, require additional studies to verify clinical benefits and identify gaps in knowledge.

Patients and Patient Organizations

The majority of patient organization respondents expressed concerns with the intent to restrict the marketing and advertising of opioids, due to potential unintended consequences and impacts on patients. Individual members of the public, including people who live with pain, shared a range of comments that largely focused on the importance of individuals having access to opioids to manage pain. In summary, they indicated:

Marketing and Advertising

- The advertising and marketing of opioids is part of a larger, more complex problem.
- Restrictions to activities such as journal advertisements would be unlikely to have an impact on prescribing practices.

Unbiased Educational Information

- Educational programs and materials for prescribers to support appropriate prescribing through education on pain management, use of alternative therapies, and new and evolving evidence, are important.
- The pharmaceutical industry plays an important role in supporting continuing medical education and sufficient oversight is in place.
- Educational events sponsored by industry provide valuable information to practicing clinicians.

Access to Treatments

- Restrictions on marketing could lead to reductions in prescribing or dosages, create access problems for those in pain, and further stigmatize those who live with pain and prescribers who prescribe opioids for pain control.

Commented [PS3]: I am very surprised by this comment. Does ORT agree?

Commented [MG4]: This is an accurate reflection of what a patient group has said. The bullet reflects what we heard and doesn't necessarily reflect HC's view on industry's role. Recommend to keep the bullet.

- It can be challenging to access prescription opioids to manage pain. Reducing access to prescription opioids would leave patients with fewer options for pain management, would negatively affect their quality of life, and may lead people to use illegal drugs.

Range of Actions

- This initiative should restrict the marketing and advertising of all drugs, and not just opioids. While the opioid crisis is a complex and multifaceted issue, illegal opioids are the principal driver, rather than the marketing of prescription opioids.
- The federal government taking immediate action on drug marketing is supported. The importance of hearing from people who are immediately affected by opioid use when developing potential restrictions should inform this action.

Industry and Industry Associations

Respondents from the pharmaceutical industry expressed concerns with restrictions that might impact their ability to provide materials to health care professionals to support the safe use of products, and reiterated the importance of industry in continuing to provide such information. Other industry respondents highlighted the potential benefits of regular mandatory reporting on the marketing and advertising of opioids. In summary:

Marketing and Advertising

- The pharmaceutical industry raised concerns with the premise of the proposal, suggesting it is simplistic to frame interactions between industry and health care professionals as marketing, indicating mandatory reporting of advertising activities would be redundant, and noting that many identified activities should not be considered advertising.

Unbiased Educational Information

- Being able to disseminate non-promotional information to health care professionals related to appropriate prescribing and the safe use of prescription medications is important.
- Health care professionals' access to educational programs and materials is fundamental. Evidence-based, independently accredited, continuing health education programs are essential to improving clinical care and patient outcomes.

Access to Treatments

- Any restrictions should take into account potential impacts and/or unintended consequences on patients with chronic pain and those living with opioid use disorder. In particular, products or information that aim to reduce or prevent risks or harms associated with problematic opioid use warrant particular consideration when setting restrictions. Some respondents believe that recent measures requiring risk management plans for opioids is beneficial, while questions were raised as to whether regulations are needed.

Range of Actions

- Other respondents noted that addressing the opioid crisis has economic and social gains, and transparency measures such as mandatory reporting on the marketing and advertising of opioids would enable investors to hold companies accountable.

Provincial Government Officials

Respondents in this group provided various comments about potential restrictions on the marketing and advertising of opioids. In summary:

Unbiased Educational Information

- Opioid prescribing decisions are not solely influenced by industry marketing.
- It is important for marketing materials to contain an equal balance of information on the potential harms and benefits of opioids.
- Any restrictions should still allow for information, education and training for health care professionals related to appropriate opioid use, as well as the distribution of clinical research and educational materials. This can help encourage evidence-based treatment decisions by health care professionals which can include opioid and non-opioid treatments.

Access to Treatments

- Restrictions must not affect access for individuals seeking pain management.

Range of Actions

- Measures to improve appropriate prescribing will assist in addressing the crisis.
- Increased transparency of the marketing and advertising of opioids is important. Regulatory bodies should be engaged to enact appropriate policies to encourage compliance.

Academia

The single submission represented 11 academics from various academic institutions in Canada, the United States and Australia. This submission strongly supported restricting the marketing and advertising of opioid products, while also emphasizing that the effects of drug promotion in general are not limited to opioids. In summary, they noted that:

Marketing and Advertising

- The current opioid crisis can, in part, be attributed to the pharmaceutical industry's marketing practices due to their considerable resources for promotion, and promotional materials often offering poor quality information.
- Physicians are exposed to drug promotion in multiple ways, noting that evidence suggests that reliance on information from pharmaceutical companies is associated with less appropriate prescribing.

Range of Actions

- Improved regulation of drug promotion is needed. Further, the Pharmaceutical Advertising Advisory Board, which reviews and pre-clears drug advertising, is not sufficiently independent from the influence of the pharmaceutical industry and a new, independent regulatory agency should be established.
- Specific to opioids, there should be increased oversight of promotion by Health Canada, restrictions to advertisements, and requirements for companies to develop plans to support appropriate prescribing. Materials to health care professionals should include advice on the de-prescribing of products.

Closing Remarks

We thank all respondents for providing comments on this proposal.

The feedback received will inform next steps for federal action including policy development and potential restrictions. We are continuing to engage stakeholders and subject matter experts throughout the fall of 2018.

For more information

Visit our Marketing and Advertising of Opioids page for updates on our work.

Contenu proposé pour le résumé sur le Web

Ce que nous avons entendu – Résumé des réponses à l'Avis d'intention de restreindre le marketing et la publicité des opioïdes

Ébauche – 10 octobre 2018

Introduction

Les Canadiens sont au deuxième rang mondial des plus grands consommateurs par habitant d'opioïdes d'ordonnance. De plus, les taux de prescription d'opioïdes ainsi que de séjours à l'hôpital et de décès attribuables à la consommation de ces produits ont augmenté rapidement. Les ordonnances remplies par des professionnels de la santé sont une source courante d'opioïdes au Canada. Les professionnels de la santé reçoivent de l'information de diverses sources, y compris de l'industrie pharmaceutique, dans le but d'orienter leurs décisions en matière de prescriptions ainsi que les conseils qu'ils donnent aux patients.

Reconnaissant l'influence du marketing et de la publicité de l'industrie sur les pratiques en matière de prescription, Santé Canada a publié un Avis d'intention (Avis) pour restreindre le marketing et la publicité sur les opioïdes auprès des professionnels de la santé. L'Avis a fait l'objet de commentaires pendant une période de consultation de 30 jours, du 19 juin 2018 au 18 juillet 2018.

Le présent rapport « Ce que nous avons entendu » contient un résumé des commentaires sur l'Avis de Santé Canada, reflétant les opinions de personnes et de groupes qui ont choisi d'y répondre. À ce titre, le présent résumé ne peut pas être rigoureusement généralisé pour englober la population canadienne dans son ensemble et ne reflète pas nécessairement la position du gouvernement du Canada.

Qui a fourni des commentaires à Santé Canada?

Tous les intervenants concernés avaient la possibilité d'émettre des commentaires sur l'Avis. Au total, 42 réponses ont été reçues, y compris de professionnels de la santé, de patients, d'universitaires et de l'industrie pharmaceutique. La ventilation des réponses par groupe d'intervenants est illustrée à la figure 1.

Figure 1 : Réponses à l'Avis d'intention de restreindre le marketing et la publicité sur les opioïdes par groupe d'intervenants

Type de répondant	Total
**Professionnels de la santé et associations de professionnels de la santé**	21
**Patients et organisations de patients**	11
**Intervenants et associations sectoriels**	6
**Représentants de gouvernements provinciaux**	3
**Universitaires**	1*
**Nombre total**	42

*Remarque : Cette réponse a été soumise au nom de plusieurs individus.

Qu'ont dit les intervenants?

Résumés des principaux thèmes

Les répondants se sont exprimés sur leurs expériences du **marketing et de la publicité** portant sur les médicaments, sur le système actuellement en place pour examiner le matériel de marketing et de publicité, ainsi que sur les incidences du matériel de marketing de médicaments sur les pratiques de prescription.

Les répondants étaient favorables aux mesures du gouvernement fédéral pour s'attaquer à la crise des opioïdes et ont proposé diverses mesures qui pourraient permettre de réduire les méfaits liés aux opioïdes. En ce qui a trait plus précisément au marketing et à la publicité sur les opioïdes, les répondants étaient généralement favorables à l'application de restrictions additionnelles et ont souligné l'importance d'une **information éducative non biaisée** afin de favoriser des pratiques de prescription appropriées. De nombreux répondants ont relevé que les mesures prises pour restreindre la publicité et le marketing sur les opioïdes ne devraient pas par mégarde limiter la disponibilité de renseignements fondés sur des données probantes.

De nombreux répondants ont également souligné l'importance d'assurer que toute restriction additionnelle n'aura aucune incidence négative sur les personnes souffrant de douleurs aiguës ou chroniques, et sur celles atteintes de troubles liés à l'utilisation d'opioïdes, qui souhaitent obtenir un **accès aux traitements** dont elles ont besoin.

Les répondants ont offert des suggestions sur un **éventail de mesures**, telles que des exigences en matière de transparence et de divulgation obligatoire, afin d'assurer une meilleure surveillance réglementaire du marketing et de la publicité sur les opioïdes. En outre, certains répondants ont souligné qu'étant donné la complexité des facteurs contribuant à la crise des opioïdes, les mesures requises vont au-delà de la question du marketing et de la publicité sur les opioïdes d'ordonnance

Résumé des commentaires

Professionnels de la santé et associations de professionnels de la santé

Les répondants ont souligné que des renseignements et des activités éducatives fiables et factuels sont essentiels pour appuyer les professionnels de la santé dans leurs rôles en tant que prestataires de soins de bonne qualité aux patients. Les associations de professionnels de la santé ont appuyé la prise d'autres mesures, quoique certains répondants aient exprimé l'inquiétude que le fait de restreindre le marketing et la publicité sur les opioïdes puisse limiter l'accès aux opioïdes pour le traitement de la douleur et des troubles liés à l'utilisation d'opioïdes. Voici un résumé de leurs propos :

Marketing et publicité

- En s'inspirant de leur expérience personnelle, certains répondants ont décrit les informations et les activités commanditées offertes par l'industrie et destinées aux professionnels de la santé comme biaisées et renfermant du contenu promotionnel. D'autres répondants ont indiqué que l'information et les possibilités éducatives offertes par l'industrie contenaient de précieux renseignements sur la gestion efficace de la douleur.
- Les publicités qui omettent ou banalisent les renseignements sur les risques ou effets indésirables d'un médicament sont potentiellement nuisibles.

Information éducative non biaisée

- Les professionnels de la santé ont un rôle à jouer pour réduire au minimum l'incidence potentielle du biais introduit par l'industrie dans la formation médicale continue en observant les normes, lignes directrices et codes d'éthique existants pour ce qui touche les relations qu'ils entretiennent avec l'industrie pharmaceutique.
- Les programmes éducatifs et la formation sont importants et il faut distinguer entre les activités promotionnelles et celles qui appuient des pratiques de prescription sécuritaires et appropriées.
- Les traitements des troublés liés à l'utilisation d'opioïdes constituent un besoin pressant, et les restrictions sur la publicité et le marketing ne devraient donc pas restreindre l'accès des professionnels de la santé à des informations éducatives et à des formations en la matière, freiner le développement de nouveaux traitements ou empêcher l'industrie pharmaceutique de sensibiliser le public à ses produits.
- Certains répondants ont indiqué que les matériels et programmes éducatifs élaborés ou offerts par l'industrie devraient être examinés par un tiers pour assurer qu'ils sont factuels et fiables, alors que d'autres ont signifié que les matériels éducatifs ne devraient pas être élaborés par des fabricants pharmaceutiques. Les matériels devraient communiquer à la fois les avantages et les risques, et inclure des détails pouvant aider à la prise de décisions, tels que les traitements disponibles sans l'apport d'opioïdes, les lignes directrices sur l'utilisation, l'efficacité du traitement et sa rentabilité.
- Il est nécessaire de s'attaquer aux problèmes du financement de la formation médicale continue sur la gestion de la douleur au moyen d'options pharmaceutiques et non pharmaceutiques.

Accès aux traitements

- L'industrie offre des renseignements et de l'enseignement bénéfiques sur les risques liés aux interactions médicamenteuses, aux nouvelles technologies et aux nouveaux médicaments. Restreindre le marketing d'opioïdes pourrait avoir une incidence négative sur la communication d'informations aux professionnels de la santé et entre ceux-ci. Par la suite, cela pourrait être défavorable aux patients souffrant de douleurs s'ils n'ont pas accès aux traitements les plus adéquats pour la gestion de la douleur.

Éventail de mesures

- Les opioïdes illégaux sont un important facteur contribuant à la crise des opioïdes. Les efforts visant à restreindre le marketing et la publicité portant sur les opioïdes d'ordonnance pourraient avoir une efficacité limitée pour régler la crise actuelle.
- Il est nécessaire de restreindre les activités promotionnelles liées aux opioïdes, ainsi qu'à tous les médicaments d'ordonnance et sans ordonnance. Il devrait y avoir des sanctions strictes en cas de non-conformité, y compris le cas échéant des accusations criminelles.
- Les restrictions devraient s'appliquer à toutes les formes de communication de l'industrie, notamment les communications verbales, écrites, électroniques et sociomédiatiques, ainsi que les visites en personne de représentants commerciaux auprès des professionnels de la santé. Les restrictions pourraient s'étendre aux groupes de défense qui reçoivent un financement de l'industrie.
- Le système volontaire actuel de préautorisation pour la publicité sur les médicaments mis en place par des organismes tels que le Conseil consultatif de publicité pharmaceutique doit être plus structuré et systématique et devrait définir des critères de publicité propres aux opioïdes.
- Il y a un appui pour accroître la transparence, la divulgation et la déclaration obligatoire à l'égard des activités de marketing, du financement et des incitatifs des sociétés pharmaceutiques visant les professionnels de la santé, les associations et les groupes de patients.
- Il devrait être interdit aux médecins de toucher une rémunération de l'industrie, notamment pour offrir des conférences éducatives à d'autres professionnels de la santé. Les professionnels de la santé devraient être rémunérés pour le temps passé comme conférencier, mais le montant de la rémunération devrait être limité.
- On pourrait permettre à l'industrie de financer des programmes éducatifs si l'activité a été homologuée par un organisme de réglementation. De la même façon, tout financement de l'industrie visant des organisations devrait servir à des activités d'apprentissage non biaisées.
- De nouveaux produits opioïdes pourraient être approuvés et assortis de conditions qui exigeraient, par exemple, des études additionnelles pour vérifier les bienfaits cliniques et cerner les lacunes dans les connaissances.

Patients et organisations de patients

La majorité des organisations de patients ont exprimé des inquiétudes quant à l'intention de restreindre le marketing et la publicité sur les opioïdes, en raison des conséquences et des incidences involontaires sur les patients. Des membres du grand public, y compris des personnes souffrant de douleurs, ont communiqué un éventail de commentaires qui portaient principalement sur l'importance pour les individus d'avoir accès aux opioïdes pour gérer la douleur. Voici un résumé de leurs propos :

Marketing et publicité

- La publicité et le marketing sur les opioïdes font partie d'un problème plus large et plus complexe.
- Les restrictions sur des activités telles que les publicités dans les revues n'auraient probablement pas d'incidence sur les pratiques de prescription.

Information éducative non biaisée

- Les programmes et matériels éducatifs à l'intention des prescripteurs en vue d'appuyer des pratiques de prescription appropriées, par la formation sur la gestion de la douleur, le recours à des thérapies non conventionnelles et la prise en compte de nouvelles données probantes en évolution, jouent un rôle important.
- L'industrie pharmaceutique a un rôle important à jouer dans le soutien à la formation médicale continue et dans la mise en place d'une surveillance suffisante.
- Les activités éducatives commanditées par l'industrie offrent de précieux renseignements aux cliniciens en exercice.

Accès aux traitements

- Des restrictions sur le marketing pourraient entraîner des réductions dans le nombre global de prescriptions ou dans les doses prescrites, créer des problèmes d'accès pour ceux qui souffrent de douleurs et stigmatiser encore davantage ceux qui souffrent de douleurs et ceux qui prescrivent des opioïdes pour maîtriser celles-ci.
- Il peut être difficile de se procurer des opioïdes d'ordonnance pour gérer la douleur. Réduire l'accès aux opioïdes d'ordonnance limiterait les options des patients pour gérer la douleur, aurait une incidence négative sur la qualité de vie de ces derniers et pourrait mener certaines personnes à se procurer des drogues illégales.

Éventail de mesures

- Cette initiative devrait restreindre le marketing et la publicité sur tous les médicaments, et pas seulement sur les opioïdes. Bien que la crise des opioïdes soit une question complexe et multidimensionnelle, le principal facteur réside dans les opioïdes illégaux plutôt que dans le marketing des opioïdes d'ordonnance.
- Il y a un soutien pour la prise de mesures immédiates par le gouvernement fédéral en matière de marketing sur les médicaments. Il importe d'entendre les personnes immédiatement touchées par l'utilisation des opioïdes en vue d'orienter l'élaboration de restrictions éventuelles.

Intervenants et associations sectoriels

Les répondants de l'industrie pharmaceutique ont exprimé des inquiétudes quant aux restrictions qui pourraient limiter leur capacité d'offrir des matériels aux professionnels de la santé visant à appuyer une utilisation sécuritaire des produits, et ont insisté sur l'importance de laisser l'industrie continuer à

offrir une telle information. D'autres répondants de l'industrie ont souligné les avantages éventuels d'une déclaration obligatoire régulière des activités de marketing et de publicité sur les opioïdes. Voici un résumé de leurs propos :

Marketing et publicité

- L'industrie pharmaceutique a soulevé des inquiétudes quant à la prémisse de la proposition, laissant entendre qu'il est simpliste de dépeindre les interactions entre l'industrie et les professionnels de la santé comme du marketing, indiquant que la déclaration obligatoire d'activités de publicité serait superflue, et soulignant que de nombreuses activités relevées ne devraient pas être considérées comme de la publicité.

Information éducative non biaisée

- Il est important de pouvoir diffuser des informations non promotionnelles auprès des professionnels de la santé relativement à la prescription appropriée et à l'utilisation sécuritaire de médicaments d'ordonnance.
- L'accès des professionnels de la santé aux programmes et matériels éducatifs est fondamental. Les programmes de formation continue en matière de santé fondés sur des données probantes et accrédités de façon indépendante sont essentiels à l'amélioration des soins cliniques et de l'état de santé des patients.

Accès aux traitements

- Toute restriction devrait prendre en compte les incidences éventuelles et les conséquences non voulues sur les patients qui souffrent de douleurs chroniques et sur ceux atteints par des troubles liés à l'utilisation d'opioïdes. En particulier, les produits ou l'information visant à réduire ou à prévenir les risques ou les préjudices associés à la consommation problématique d'opioïdes méritent une attention particulière lors de l'instauration de restrictions. Certains répondants croient que des mesures récentes exigeant des plans de gestion des risques visant les opioïdes sont utiles, alors que d'autres ont contesté la raison d'être de règlements.

Éventail de mesures

- D'autres répondants ont souligné que la prise en compte de la crise des opioïdes permet de faire des gains économiques et sociaux, et que des mesures de transparence telles que la déclaration obligatoire du marketing et de la publicité sur les opioïdes permettraient aux investisseurs de responsabiliser les entreprises.

Représentants de gouvernements provinciaux

Les répondants dans ce groupe ont offert divers commentaires sur les restrictions éventuelles touchant le marketing et la publicité sur les opioïdes. Voici un résumé de leurs propos :

Information éducative non biaisée

- Les décisions de prescrire des opioïdes ne sont pas influencées uniquement par le marketing de l'industrie.
- Il importe que les matériels de marketing fassent la part égale aux préjudices et aux bienfaits éventuels des opioïdes.
- Toute restriction devrait quand même permettre d'offrir de l'information, de l'enseignement et de la formation aux professionnels de la santé relativement à la consommation appropriée d'opioïdes, ainsi que de distribuer des résultats de recherches cliniques et des matériels éducatifs. Cela peut encourager les professionnels de la santé à prendre des décisions fondées sur des données probantes quant au traitement à offrir, lequel peut comprendre des médicaments avec et sans opioïdes.

Accès aux traitements

- Les restrictions ne doivent pas avoir d'incidence sur l'accès des personnes qui souhaitent gérer la douleur.

Éventail de mesures

- Des mesures pour améliorer les pratiques de prescription appropriées aideront à contrer la crise.
- Une transparence accrue autour du marketing et de la publicité sur les opioïdes est importante. Les organismes de réglementation devraient être mobilisés pour exécuter des politiques appropriées dans le but de favoriser la conformité.

Universitaires

L'unique soumission représentait 11 universitaires de divers établissements universitaires au Canada, aux États-Unis et en Australie. Cette soumission était fortement en faveur de restreindre le marketing et la publicité sur les produits opioïdes, tout en insistant sur le fait que les effets de la promotion des médicaments en général ne se limitent pas aux opioïdes. Voici un résumé de leurs propos :

Marketing et publicités

- La crise des opioïdes actuelle peut en partie être attribuée aux pratiques de marketing de l'industrie pharmaceutique, en raison des ressources considérables de cette dernière en matière de promotion, et à la qualité souvent mauvaise de l'information communiquée dans les matériels promotionnels.
- Les médecins sont exposés à de multiples méthodes de promotion des médicaments, et on souligne que les preuves laissent entendre que le recours à l'information provenant de sociétés pharmaceutiques est associé à des pratiques de prescription moins appropriées.

Éventail de mesures

- Une réglementation améliorée de la promotion des médicaments est requise. De plus, le Conseil consultatif de publicité pharmaceutique, qui examine et préautorise la publicité sur les médicaments, n'est pas suffisamment dégagé de l'influence de l'industrie pharmaceutique, et un nouvel organisme de réglementation indépendant devrait être établi.
- Plus particulièrement par rapport aux opioïdes, il devrait y avoir une surveillance accrue par Santé Canada de la promotion effectuée par l'industrie, des restrictions sur les publicités, ainsi que des exigences pour obliger les entreprises à élaborer des plans de soutien aux pratiques de prescription appropriées. Les matériels destinés aux professionnels de la santé devraient inclure des conseils sur la déprescription de produits.

Mot de la fin

Nous remercions tous les répondants de leurs commentaires sur cette proposition.

Les commentaires reçus éclaireront les prochaines étapes qu'entreprendra le gouvernement fédéral, notamment l'élaboration de politiques et la mise en place de restrictions éventuelles. Nous poursuivrons nos entretiens avec les intervenants et les experts en la matière tout au long de l'automne 2018.

Pour en savoir davantage

Consultez la page sur le marketing et la publicité visant les opioïdes pour des mises à jour sur nos travaux.

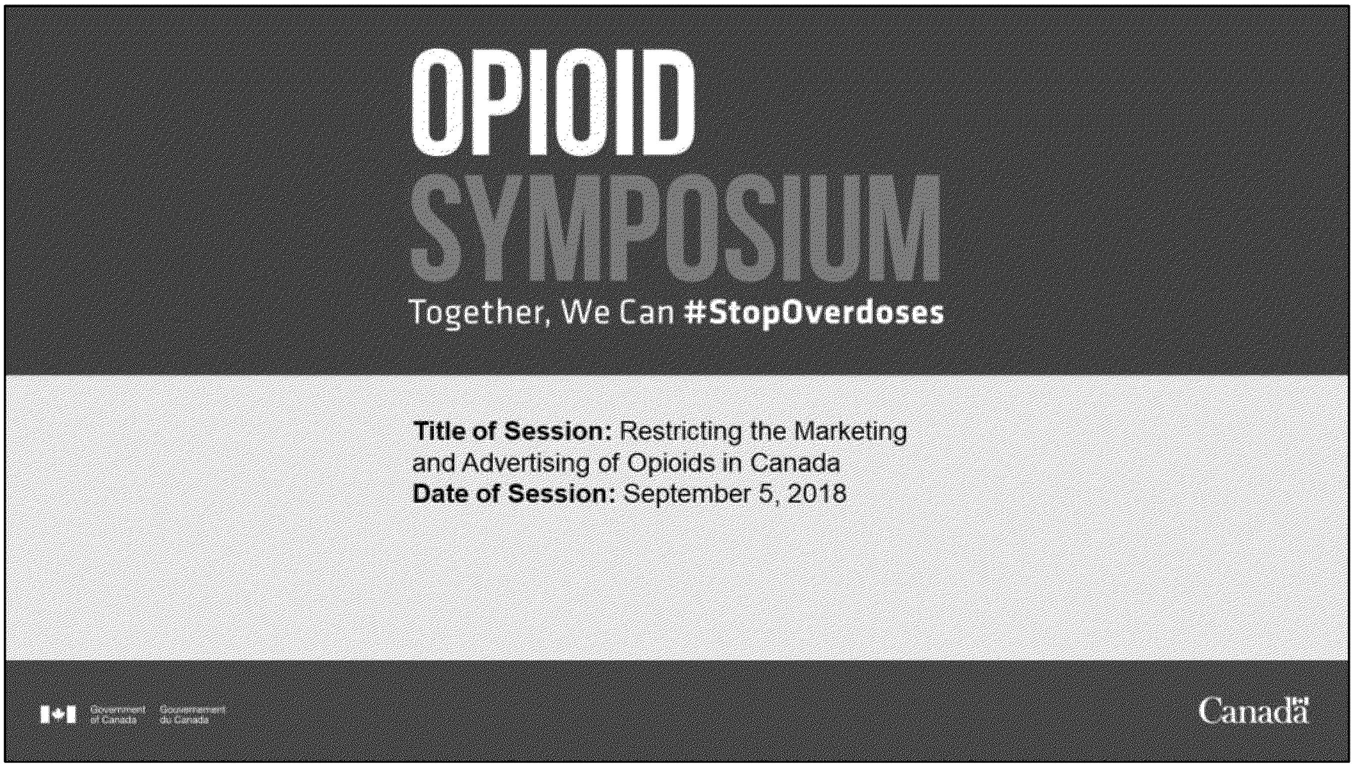

- Thank you Rita.
- Good afternoon everyone, my name is Ed Morgan and I am the Director General of the Policy, Planning, and International Affairs Directorate at Health Canada.
- It's a pleasure to be here today.
- We have heard a lot during the Symposium about the impact of problematic opioid use on the lives of individuals, families and communities.
- This afternoon, I want to talk to you about one aspect of the Government of Canada's actions to address the opioid crisis – restricting the marketing and advertising of opioids.

Purpose

To update stakeholders on Health Canada's actions to date, and to consult on further restricting the marketing and advertising of opioids

- Today, we want to get your feedback potential restrictions to opioid marketing and advertising aimed at health professionals.
- We want to hear from you how these potential restrictions could impact different groups.
- But before we get into that, I would like to provide you with an update on what Health Canada is doing to address the pharmaceutical industry's marketing and advertising of opioids.

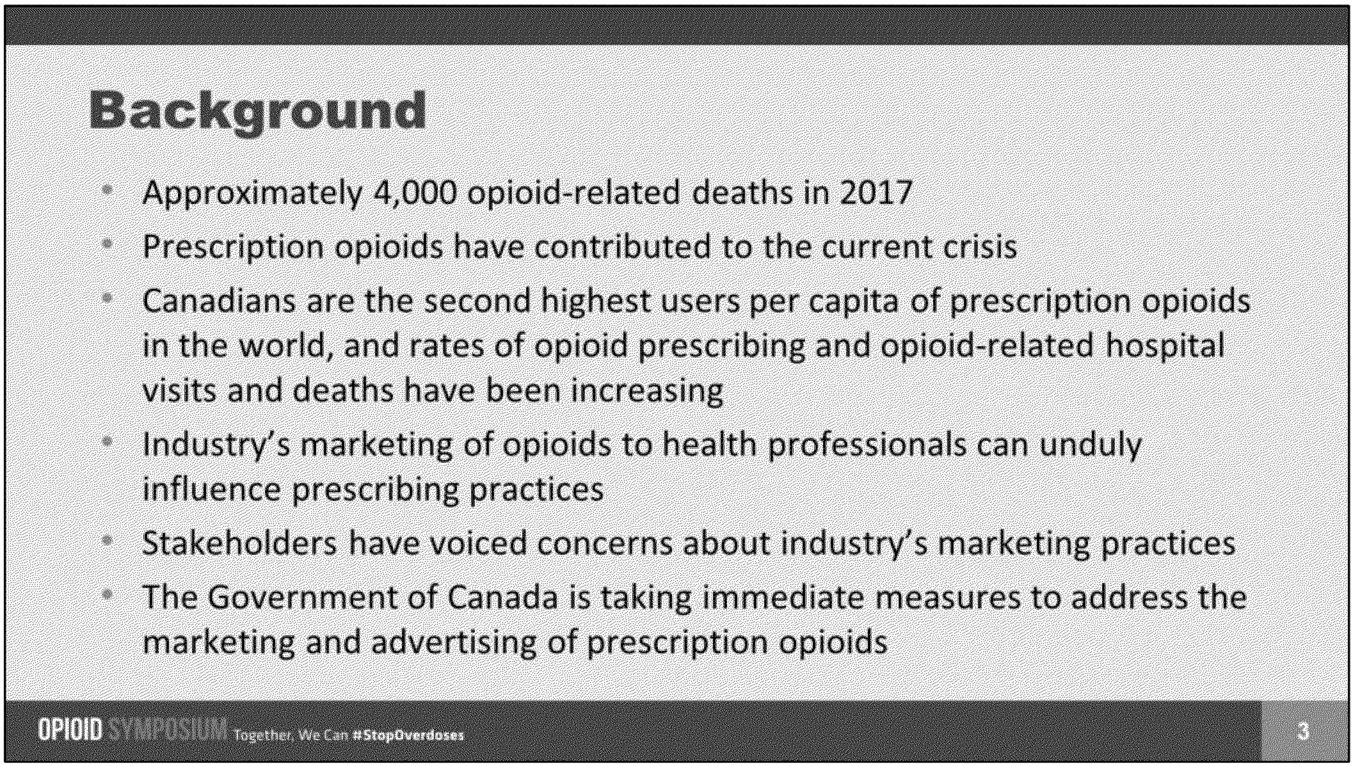

- As I am sure you are aware, the opioid crisis is Canada's most significant public health crisis in recent history, and it continues to worsen.
    - In 2017, there were approximately 4,000 opioid-related deaths, up from approximately 2,800 in 2016.

- Opioid medications are intended to help people who are experiencing serious pain, and we know that for some Canadians, prescription opioids are medications that are effective in managing their pain.

- While there is value in the pharmaceutical industry conveying educational and scientific information about a health product, we know that industry marketing practices can unduly influence health professionals and lead to over-prescribing of opioids.

- We have heard from many of you that something needs to be done to address inappropriate marketing and advertising of opioids. Some stakeholders have clearly indicated the existing measures are not enough.

- The Government of Canada is taking action to address the issue.

- Let me be clear, we definitely recognize the need for continued access to these medications. While we are proposing to restrict the **marketing and**

advertising of opioids, these actions are not intended to restrict access to medications for those who need them.

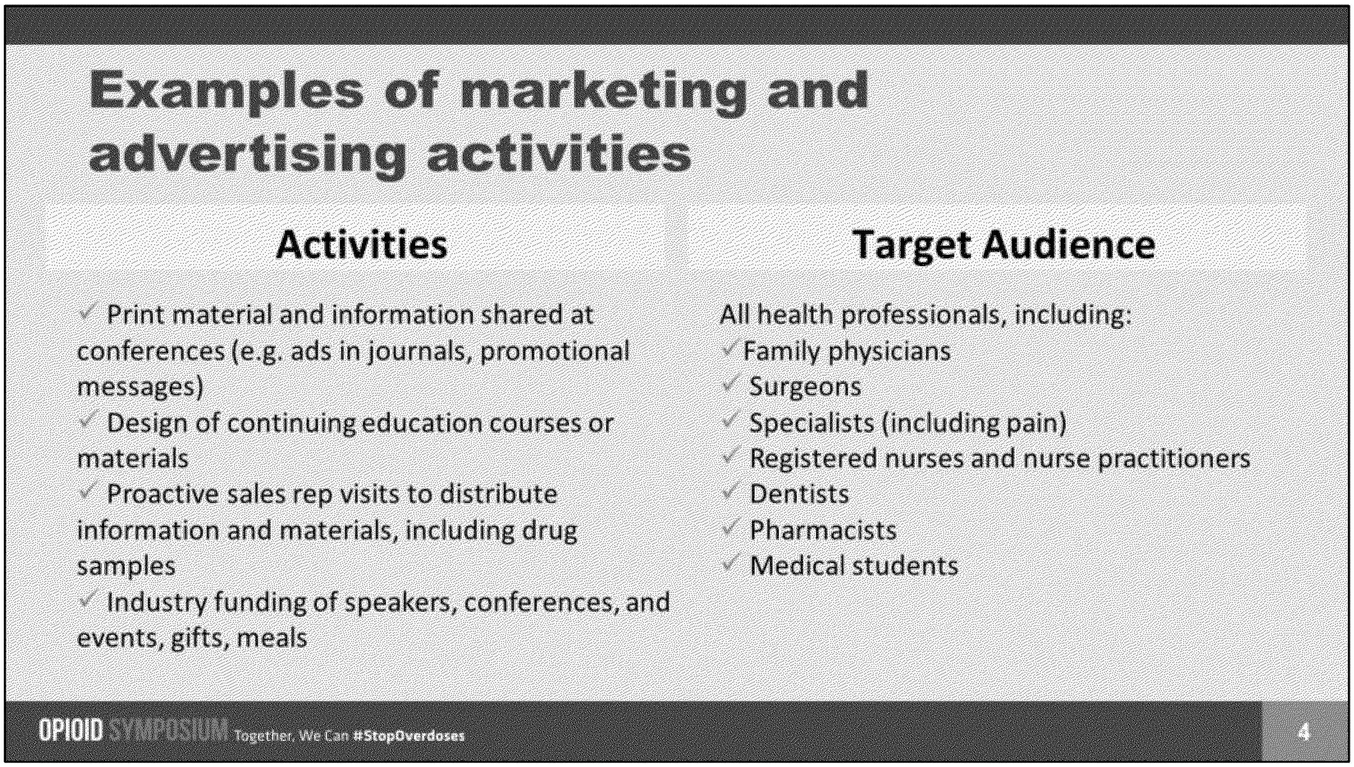

- When we talk about marketing and advertising in this context, we are talking about a broad range of activities initiated by the pharmaceutical industry and targeted at health professionals.
- These activities can be direct or indirect, including things like print advertisements in journals, visits to doctors from industry sales representatives and payments to speakers at conferences.
- The promotional messaging embedded in these activities can unduly influence prescribing practices.

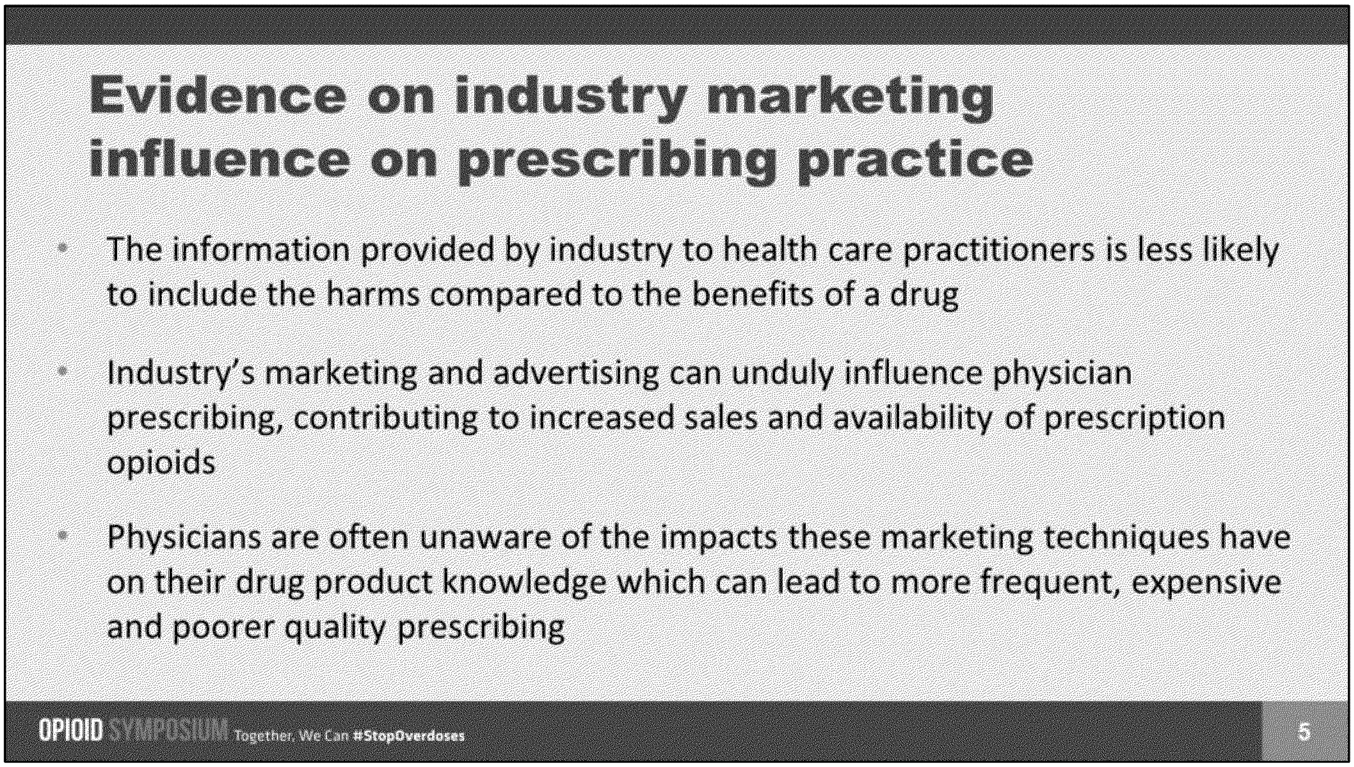

- Evidence indicates that materials shared with health care practitioners by industry are often biased.
- For example, information provided to health care professionals by pharmaceutical industry representatives often emphasize the benefits of drugs while downplaying the potential harms.
- In addition, we know that this marketing can influence physician prescribing, lead to more prescriptions, increased costs, and lower prescribing quality.
- Additionally, physicians, like any of us, are often unaware of the extent to which marketing can impact their own behaviour, which limits their ability to recognize and mitigate that influence when prescribing.

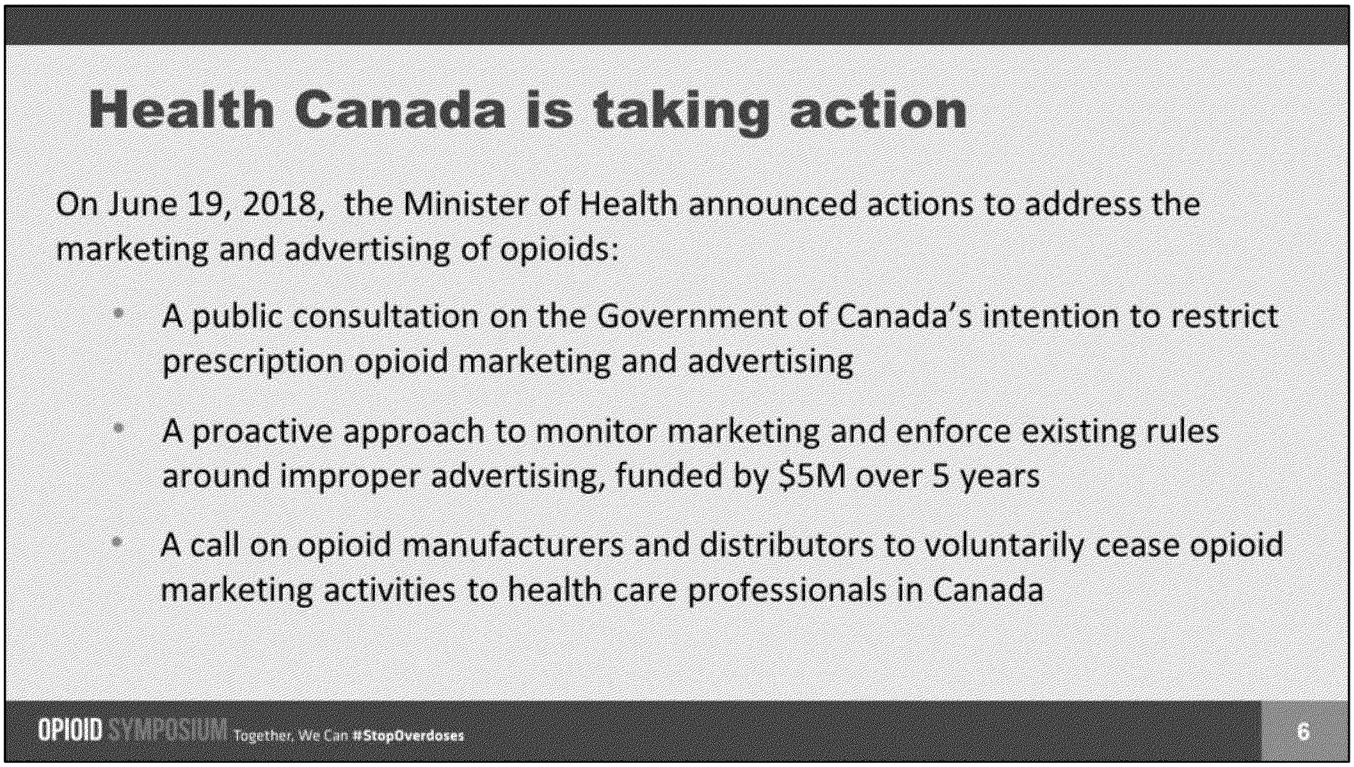

- For these reasons, the Minister of Health announced on June 19th federal government actions to further restrict the advertising and marketing of opioids in Canada.

- Health Canada launched a formal consultation to hear from you, and all stakeholders, about what those restrictions on marketing could look like and the impact they could have.

- The Minister of Health also called on opioid manufacturers and distributors in Canada to immediately stop the marketing and advertising of opioids, on a voluntary basis.

- Some companies have responded to this letter and, as announced by the Minister today, those responses have been made available to the public on our website.

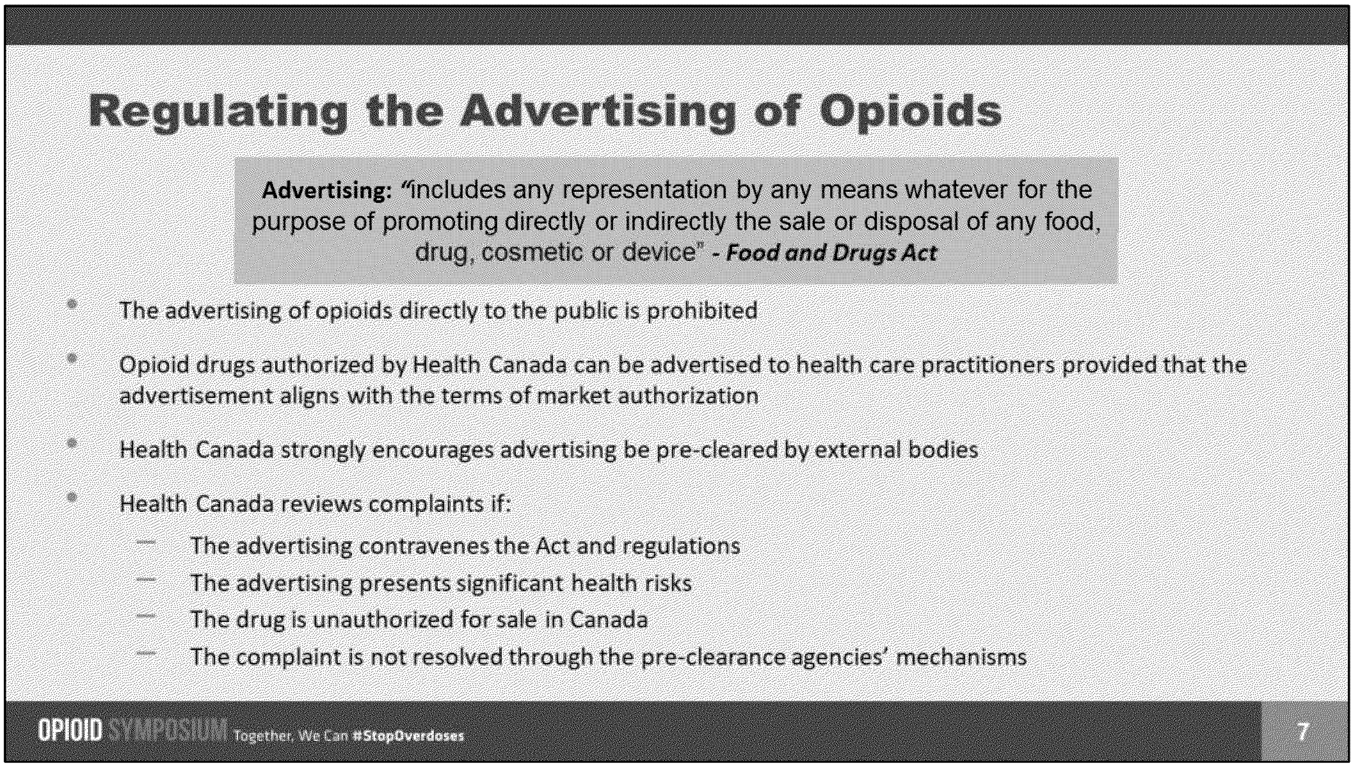

- These actions complement existing rules for advertising opioids in Canada.
- Under current rules, the pharmaceutical industry is not allowed to advertise opioids to the general public. Opioids can be advertised only to health care professionals.
- Advertisements need to be aligned with the terms of the opioid's approval from Health Canada. For example, this means a drug cannot be advertised to treat a condition other than what it was approved to treat.
- Health Canada strongly encourages industry advertising be pre-cleared by external bodies.
- Health Canada also investigates complaints if advertising activities pose significant health risks or if advertisements contravene the law.

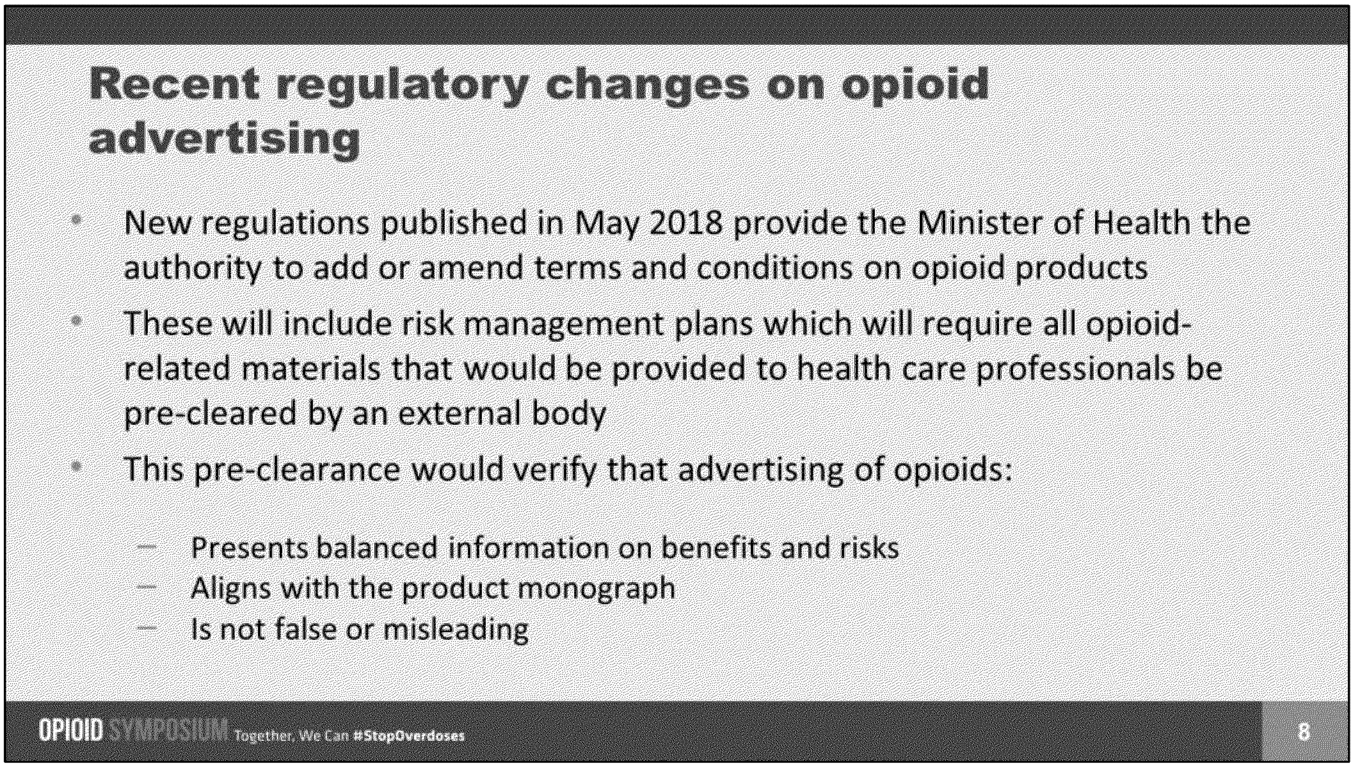

- In addition, new regulations that were published in May of this year introduce terms and conditions on opioid products.
- This will require opioid manufacturers to submit risk management plans to Health Canada.
- These risk management plans will **require** that opioid-related materials be pre-cleared by an external body.
- In the case of materials directed to health care professionals, this pre-clearance is conducted by the Pharmaceutical Advertising Advisory Board.
- Pre-clearance ensures that advertising is aligned with the product monograph.
- The product monograph is a document that is approved by Health Canada and includes information on the ingredients a drug can contain, the directions for the use of the drug, the conditions for which it can be used, and possible side effects or risks.
- Pre-clearance also ensures that advertising portrays a balance of the benefits and risks of the drug, and that there are no false or misleading

claims.

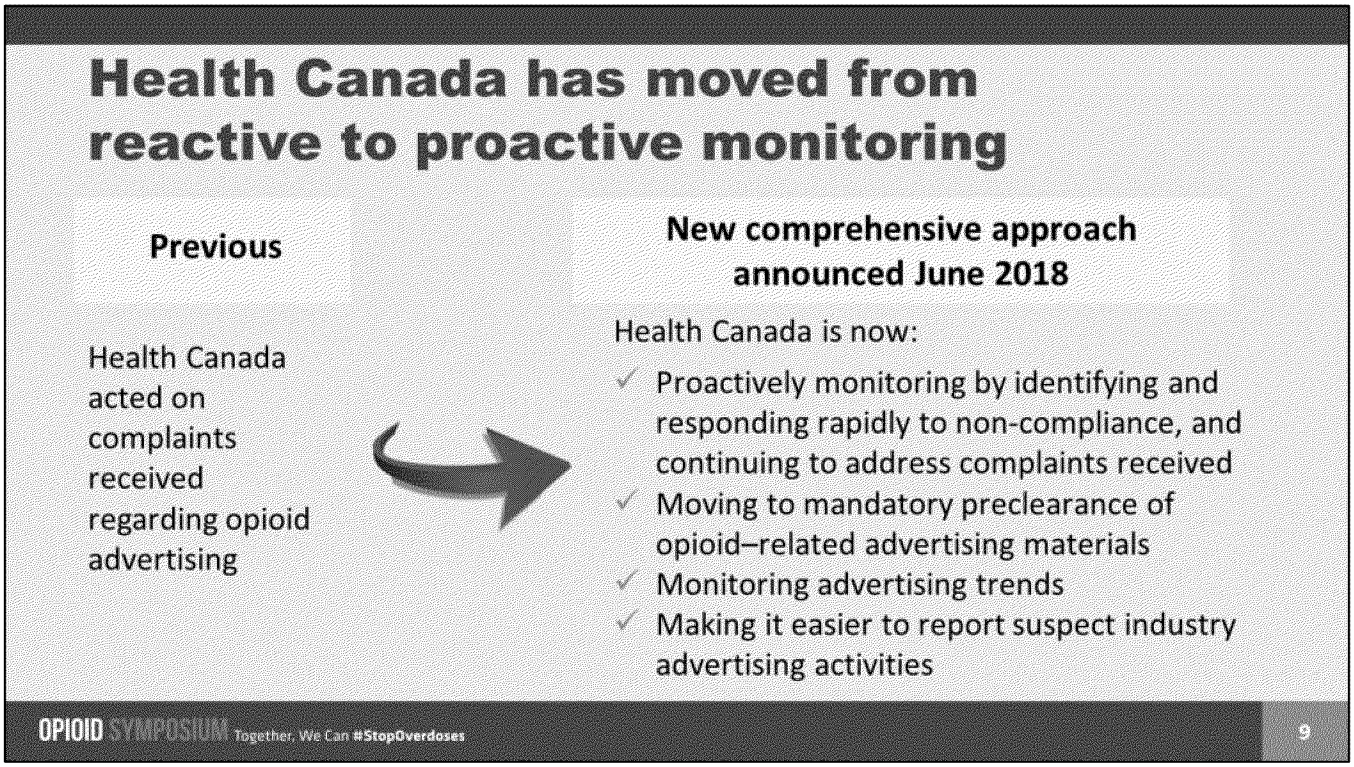

- At the same time, we are moving to a proactive approach to processing advertising complaints.

- Proactive monitoring will help identify and address high-risk cases of misleading advertising, with the aim of minimizing the potential health risks to Canadians.

- Health Canada will conduct outreach activities to better educate healthcare professionals on advertising regulations, assist them in identifying non-compliant advertising, and make it easier for them to report inappropriate marketing to Health Canada.

- Health Canada will also be taking stronger action when we find non-compliant advertising.

- This could include site visits to opioid manufacturers, seizing advertising materials, issuing a public communication, or recommending criminal charges, if appropriate.

- This approach is backed by an investment of $5 million in operating resources over five years.

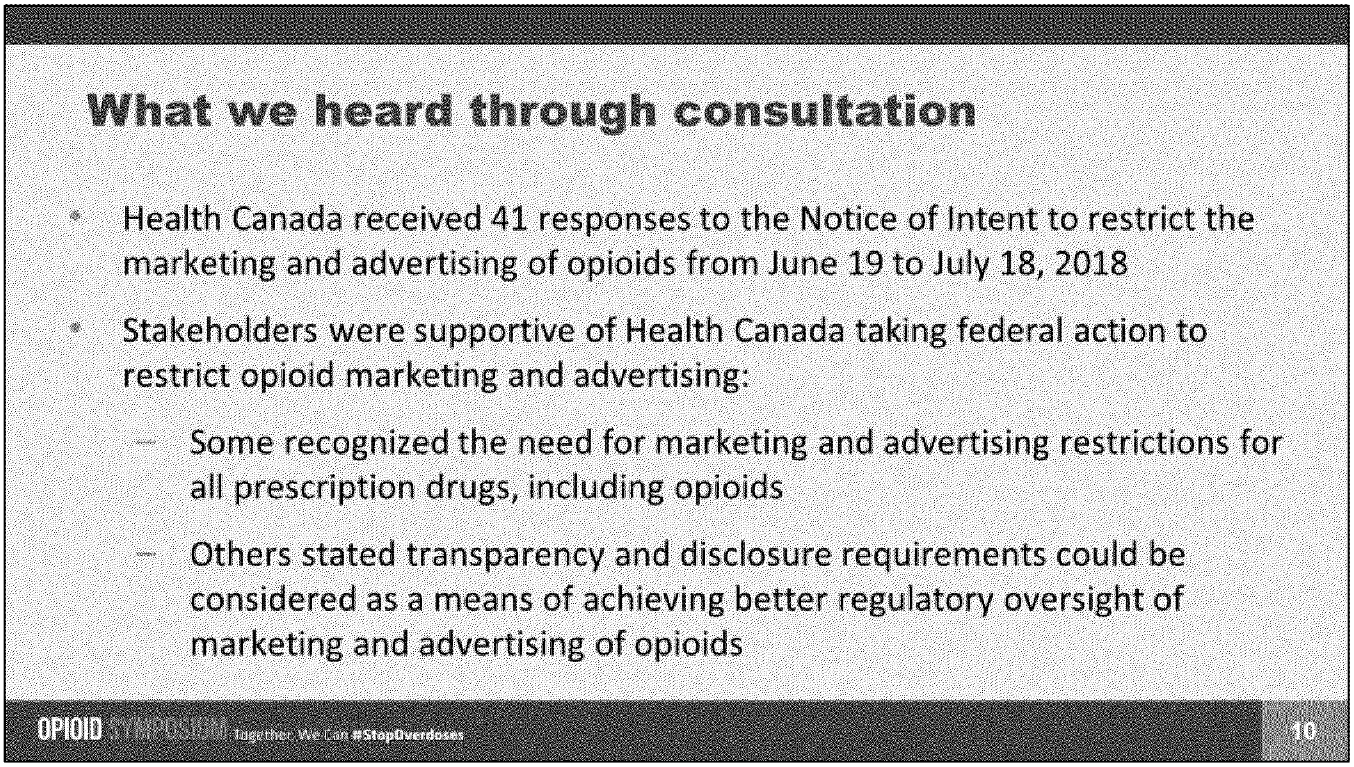

- As mentioned previously, in June and July, we held a public consultation to hear from the public and stakeholders about the Government of Canada's intention to further restrict the marketing and advertising of opioids.
- We received feedback from over 40 organizations, companies, and individuals as part of these consultations. In general, stakeholders were supportive of Health Canada taking action to restrict the marketing and advertising of opioids.
- Some respondents suggested there was a need for marketing and advertising restrictions for opioids and **all** prescription drugs.
- Others suggested that transparency and disclosure requirements could be considered.

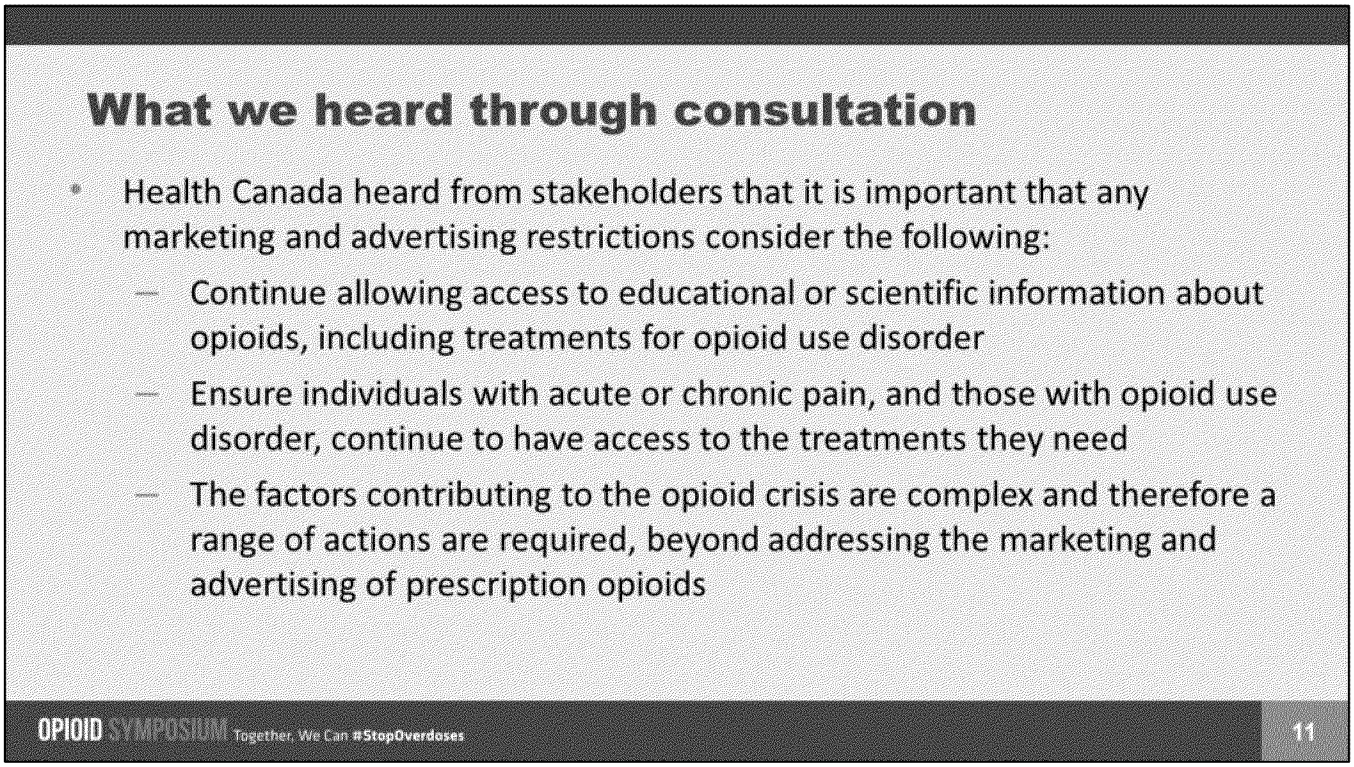

- Stakeholders noted that **educational or scientific information about opioids has a key purpose** and should therefore still be permitted.

- Some stakeholders noted that information sharing on opioid use disorder treatment is especially important.

- Health Canada understands the importance of educational material in supporting health care professionals' prescribing decisions.

- In addition, many stakeholders underscored the importance of ensuring that individuals with acute or chronic pain, and those with opioid use disorder, continue to have access to the treatments they need.

- Let me be clear, the intent of restrictions on the marketing and advertising of opioids to health professionals is not to restrict access to treatment options for people who need them.

- Finally, the variety of comments we received during this consultation

underscores that the factors contributing to the opioid crisis are complex.

- For example, some stakeholders stated that the majority of harms in the opioid crisis are caused by illegal drugs, rather than prescription opioids.

- The restriction of marketing and advertising activities is just one part of the government of Canada's comprehensive opioid response. We absolutely agree that a range of actions are required to address the crisis.

- Although the severity of the harms in the current opioid crisis can be attributed to illegal drugs, prescription opioids have also been a contributing factor. For instance, evidence indicates that 52% of 2015-16 opioid-related hospitalizations in Manitoba and Ontario and 34% in British Columbia occurred in people with an active opioid prescription.

- Additionally, a recent study indicated that more than 75% of the 2016 opioid overdose deaths in Ontario were attributed to patients who had an active opioid prescription at one point between 2013 and 2016.

- We can confidently say that we have heard you and are considering all these comments as we move forward.

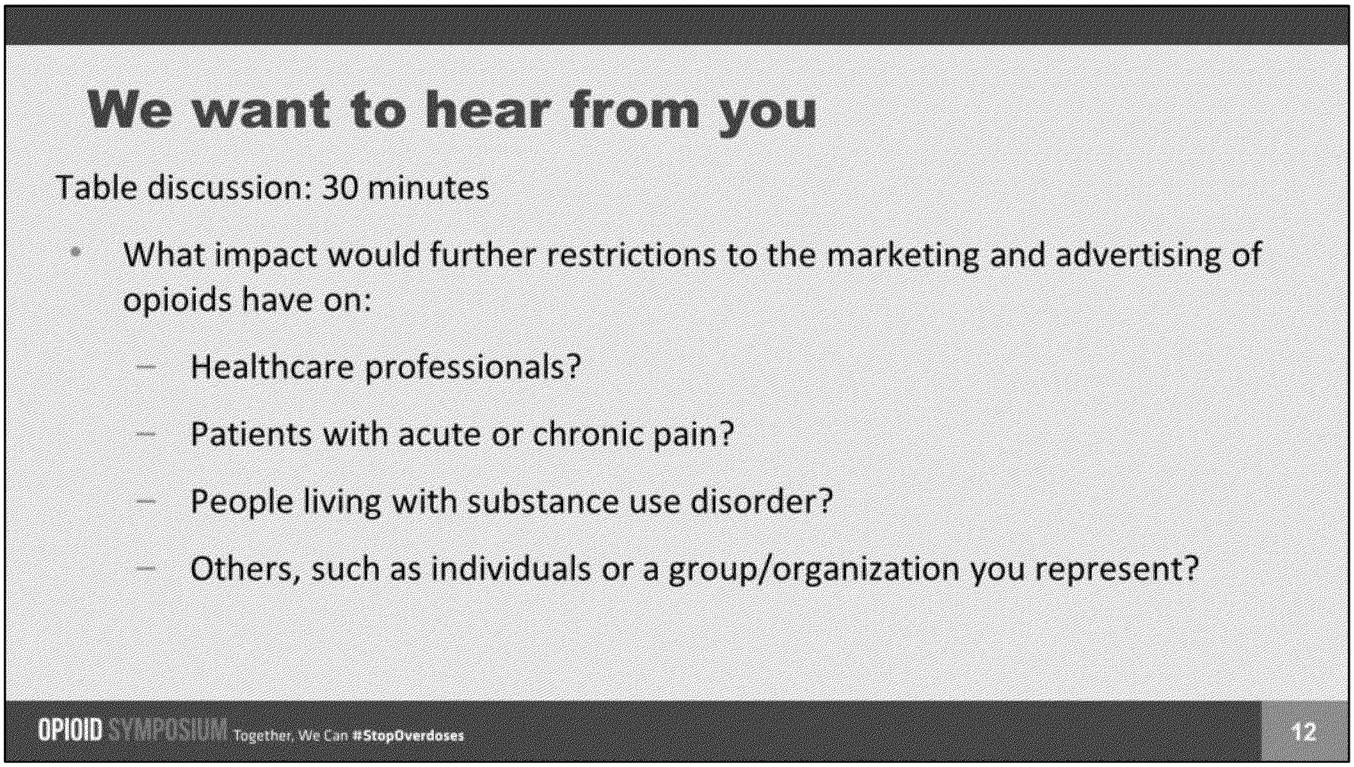

- We are committed to hearing from Canadians to help shape how Health Canada moves forward to address this issue, so today we want to hear from you.

- We would like you to spend the next half hour talking in the groups at your table.

- We would like you to consider the following questions: What do you think would be the impact of these possible restrictions? Is there anything that we missed?

- For example, how would potential restrictions to marketing and advertising impact:

  - Health care professionals?
  - Patients with acute and chronic pain?
  - People living with substance use disorder?
  - Others?

- You can think about both positive and negative impacts that could arise. The goals is to really tease out the potential impacts on these groups.

- Please be sure to identify someone at your table who will be responsible for recording the conversation on the worksheet, which looks like this *[hold up worksheet as example].*

- It is important that you write down all the ideas from your group discussion.

- We will be using the worksheets to support our record of the discussion and most importantly, to inform our work going forward.

- After 30 minutes of discussion at your tables, there will be an opportunity to share one key comment with the room. It could be the theme that your group finds most important, or the one potential impact that you, as a group, feel is most surprising or overlooked.

- If you run out of space on the worksheet, or have other comments you would like to provide to us, you can write them on the back of your sheet.

- In addition, if you have any questions about the worksheet, you can ask Health Canada staff who will be circulating in the room.

- For those of you who are participating in the web cast, we invite you to share your comments on these questions via email (go to next slide for email address).

Reporting back

- Please limit comments to one key theme per table
- Written comments will be collected at the end of the session
- Additional comments can be sent to: hc.ppiad-dppai.sc@canada.ca **by September 21**

- It sounds like there was some great discussion at your tables. We would love to hear a bit about the ideas you were discussing.
- To start, we ask that you limit your comments to one point per table, to ensure that we can hear from as many people as possible
- If you would like to share a comment, please raise your hand and Health Canada staff will come to your table with a microphone.
- With that, I will turn it over to the floor, what are some of the most interesting points that came out of your discussion?

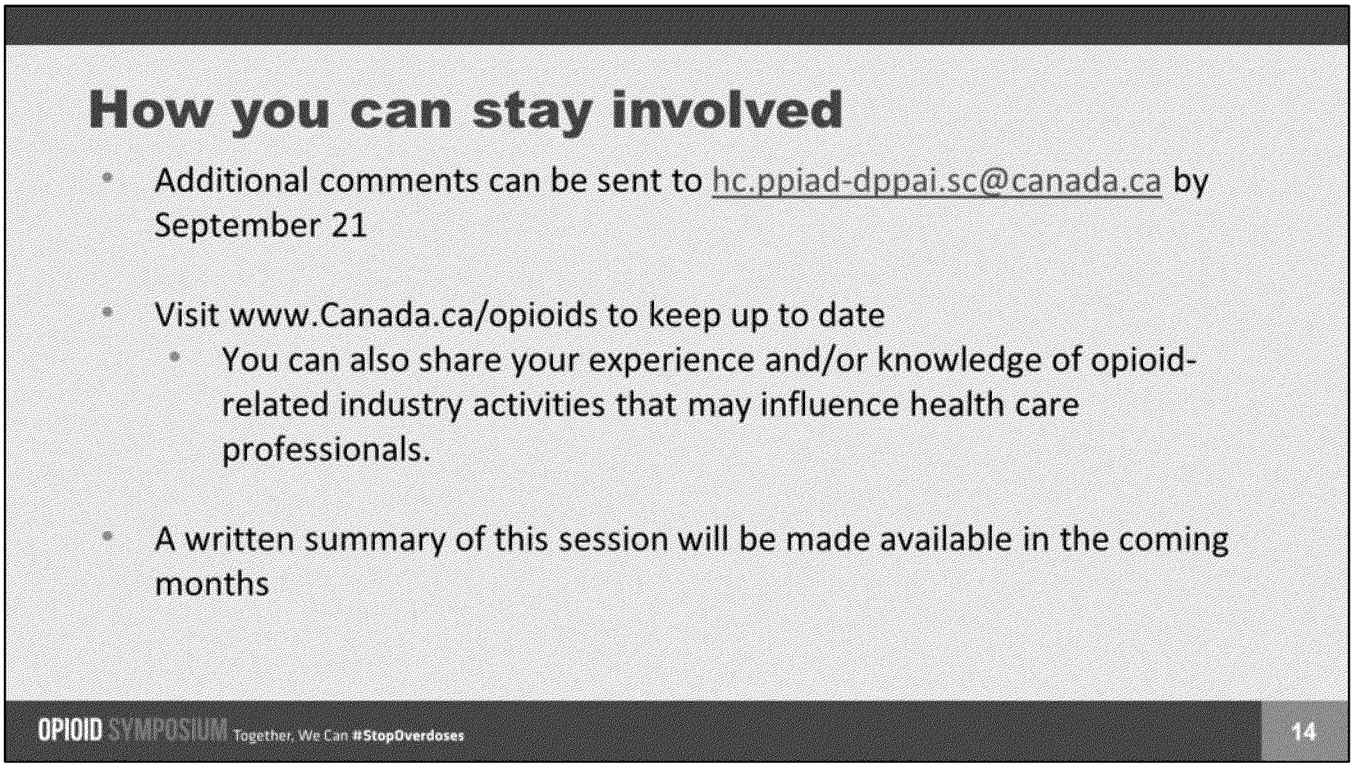

- That is all the time we have for reporting back. Thank you for sharing your input with us.

- I think your comments reflect the level of complexity involved in addressing this issue and its importance.

- For those tables who did not have a chance to share a comment, rest assured that we will review all your comments and will take them into consideration as we determine how to move forward.

- Health Canada staff will be circulating to collect the worksheets to ensure that we have a record of your table discussions.

- I also want to note that you can share additional thoughts that were not captured on your worksheet by sending them to our email inbox over the next two weeks. Our email address is on the screen in blue.

- You may like to write it down, or you can grab one of the contact cards that we have left on your tables, which look like this *[hold up contact card as example]* .

- As we move forward with federal action on this issue, you can keep up to

date by checking our webpage: canada.ca/opioids.

- In addition, if you are a healthcare professional who would like to share your experiences with Health Canada, regarding industry’s marketing of opioids, you can do so via the website.

- Lastly, a written summary of the feedback received during this session will be made available in the coming months.

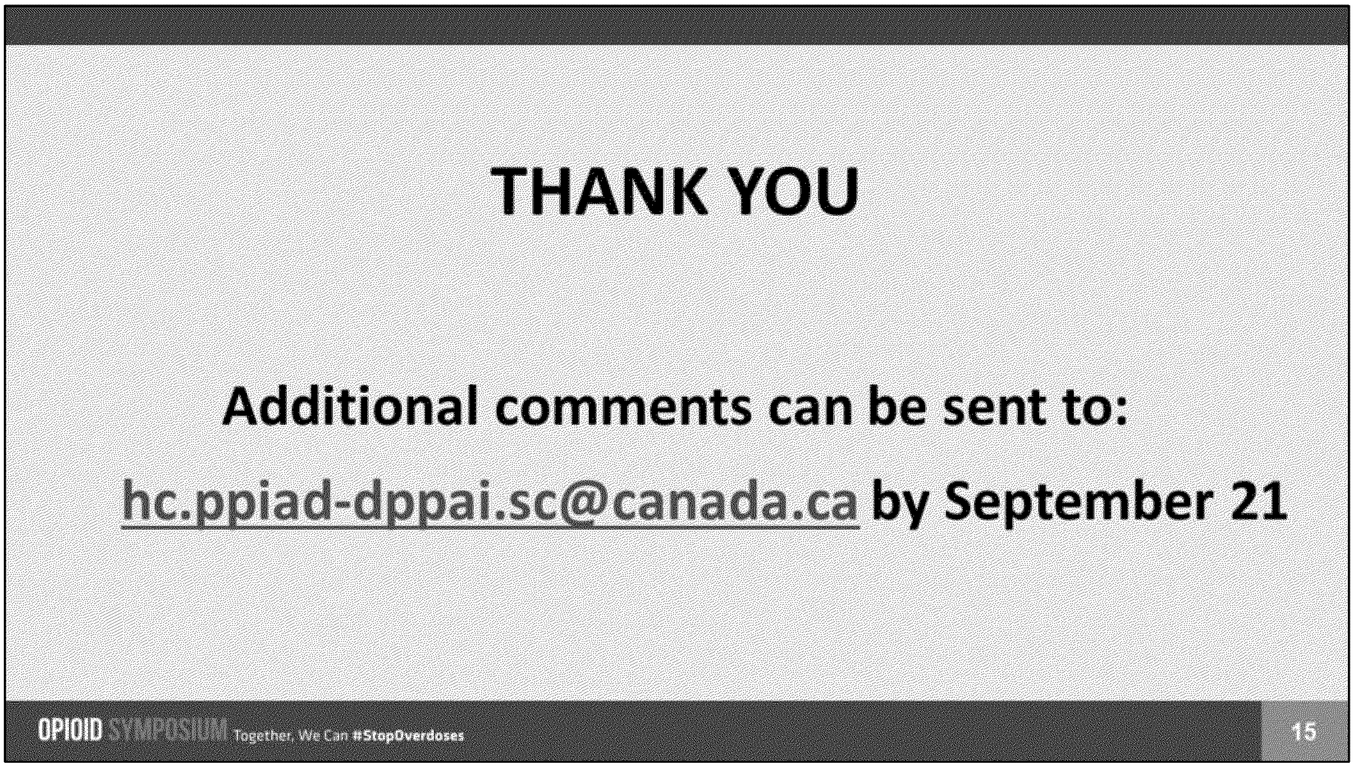

- Thank you again for the insightful discussion today, and for the valuable input that you provided.
- I will now hand the microphone over to my colleague, Kristen Mattison, who will be moderating the session on Canadian Drugs and Substances Strategy.

References

National report: Apparent opioid-related deaths in Canada. Published 2018. Available at https://www.canada.ca/en/public-health/services/publications/healthy-living/national-report-apparent-opioid-related-deaths-released-june-2018.html

International Narcotic Control Board. Narcotic Drugs Technical report: Estimated World Requirements for 2017 – Statistics for 2015. 2017.

Canadian Institute for Health Information - Pan-Canadian Trends in the Prescribing of Opioids, 2012 to 2016. Published in 2017; available at: https://www.cihi.ca/sites/default/files/document/pan-canadian-trends-opioid-prescribing-2017-en-web.pdf

Opioid crisis having "significant" impact on Canada's health care system (2018); available at: https://www.cihi.ca/en/opioid-crisis-having-significant-impact-on-canadas-health-care-system

Dhalla, I. et al. 2009. Prescribing of opioid analgesics and related mortality before and after the introduction of long-acting oxycodone. CMAJ. 181(12).

Lexchin, J et al. "The danger of imperfect regulation: Oxycontin use in the United States and Canada." International Journal of Risk & Safety in Medicine, 23, 2011.

Van Zee, A. 2009. The promotion and marketing of oxycontin: commercial triumph, public health tragedy. Am J. Public Health. 99

United States General Accounting Office. December 2003. Report to Congressional Requesters. Prescription Drugs: OxyContin Abuse and Diversion and Efforts to Address the Problem. GAO-04-110.

Mintzes, B. et al. "Pharmaceutical Sales Representatives and Patient Safety: A Comparative Prospective Study of Information Quality in Canada, France and the United States." Journal of General Internal Medicine, 28 (10) October 2013.

Robertson, C. et al. 2012. Effect of Financial Relationships on the Behaviours of Health Care Professionals: A Review of the Evidence. Journal of Law, Medicine & Ethics

Sah, S. and Fugh-Berman, A. Physicians under the Influence: Social Psychology and Industry Marketing Strategies. Journal of Law, Medicine & Ethics. Fall 2013

Wazana, A. "Physicians and the Pharmaceutical Industry: Is a Gift Ever Just a Gift? JAMA 283, no 3 (2000).

Hadland, S. et al. 2018. Association of Pharmaceutical Industry Marketing of Opioid Products to Physicians with Subsequent Opioid Prescribing. JAMA.

Hadland, S. et al. 2017. Industry Payments to Physicians for Opioid Products, 2013-2015. AJPH. Vol 107 (9).

Spurling, G. et al. 2010. "Information from Pharmaceutical Companies and the Quality, Quantity, and Cost of Physicians' Prescribing: A Systematic Review." PLOS Medicine 7.

OPIOID SYMPOSIUM: RESTRICTING THE MARKETING AND ADVERTISING OF OPIOIDS IN CANADA

Before we begin, who is at your table? Please circle all that apply

People with lived and living experience	Health Professional Organizations	First Nations, Inuit, and Indigenous Groups	Substance Use Experts/Academics
First Responders	Pain Perspective Organizations	Provincial government representatives	Other (please specify):
Law Enforcement	Drug Policy Advocates	Federal government representatives	

What impact would further restrictions to the marketing and advertising of opioids have on:

Healthcare professionals	Patients with acute or chronic pain
People living with substance use disorder	Others, such as individuals or a group/organization you represent (please specify)

SYMPOSIUM SUR LES OPIOIDES: RESTREINDRE DAVANTAGE LE MARKETING ET LA PUBLICITÉ SUR LES OPIOÏDES

Avant de commencer, qui est à votre table? Veuillez encercler tout ce qui s'applique.

Les personnes ayant vécu ou vivant présentement une expérience pertinente	Ordres professionnels de la santé	Groupes autochtones et inuits	Universitaires et experts en toxicomanie
Intervenants de première ligne	Groupes de défense de la personne aux prises avec des douleurs	Représentants du gouvernement provincial	Autre (veuillez préciser):
Forces de l'ordre	Défenseurs de politiques en matière de drogues	Représentants du gouvernement fédéral	

Quel serait l'impact de nouvelles restrictions sur le marketing et la publicité sur les opioïdes sur :

Les professionnels de la santé?

Les patients souffrant de douleurs chroniques ou aiguës?

Les personnes atteintes de troubles liés à la consommation d'opioïdes?

Les autres, comme les personnes ou un groupe/une organisation que vous représentez?

Remarks for
the Honourable Ginette Petitpas Taylor
Minister of Health

Opioid Symposium

September 5, 2018

Toronto, ON

1240 words (approx. 10 minutes)

Check against delivery

[*Good morning / good afternoon*] everyone.

Thank you for joining me.

It's wonderful to be back in Toronto.

I would like to begin by acknowledging that the land on which we gather is the traditional territory of the Ojibway, the Anishnabe and the Mississaugas of the New Credit.

And I'd like to acknowledge Rita Notarandrea from the Canadian Centre on Substance Use and Addiction and [*name(s) here*] from the Centre for Addiction and Mental Health.

Commented [LE1]: For...?

I would also like to thank The Globe and Mail and La Presse (TBC) for their support. Special thanks to the Globe and Mail for the use of this state of the art facility. Thank you to our four supporters in organizing this Symposium.

Commented [LE2]: Sponsors?

As we start the next two days, I think it's important to recognize that we are all here because we ~~all~~ share a commitment to facing the serious challenge of the opioid

crisis and we all want to ~~save~~ improve the lives of those who are affected by it.

Statistics

As you know, the statistics are shocking. The numbers show that almost 4,000 lives were lost to opioid-related overdoses in 2017.

The news is not good.

Addressing the opioid crisis is a top priority for this government. It is certainly a top priority for me, for many reasons.

Lives have been lost, there's huge cost to communities, devastating impact on families and friends ... it is a national tragedy.

A tragedy that plays out from coast to coast to coast.

It affects our First Nation communities, it affects new Canadians. It affects folks in cities, in rural areas, in suburbs. It affects the young, the old … men, women, and our children.

[*Minister can relate experience as a social worker, or from her visit to Vancouver's East Side in 2017*.]

Yet there is ~~some~~ hope in all this and we can see it in the room today. This crisis has brought together individuals with lived and living experience, professional groups, the scientific community, medical agencies … different levels of government. All dedicated to addressing this national health crisis.

This is where we are now … it's "all hands on deck."

And I believe that our diversity of background and views will help ensure a comprehensive, compassionate and collaborative approach.

Commented [AM3]: Later there are three pillars referenced whre "collaborative" is replaced with "evidence-based". We should be consistent thoughout. Recommend changing collaborative here to evidence-based.

As I said earlier, we need to consider all aspects of the opioid crisis.

We need to look at the foundations of the problem and find a long-term approach as part of an overall drugs and substance strategy.

We need to listen to Canadians with lived and living experience and people living with chronic pain. And we need to learn from them.

Government of Canada Actions

Over the last year, I have had the privilege to meet with many of you around the room to hear your perspectives, collaborate and learn from you.

In particular, I have been moved by your stories and your experiences.

[*Minister could discuss roundtables she hosted with people with lived and living experience, including the one the night before Symposium to learn from people living in chronic pain – what she heard (TBC)*.]

We need to hear your voice. We need to hear what the opioid crisis has done to families and to communities.

Because finding a strategy that is compassionate, comprehensive and evidence-based, will require us to collaborate and consider all sides, and all angles of this issue.

In September 2017, as my first engagement on this issue as Minister of Health, I committed to:

- removing regulatory barriers to treatment for substance use disorder;
- encouraging innovative ideas to address the opioid crisis;
- renewing the Canadian Drugs and Substances Strategy; and
- reducing stigma.

In June 2018, I added to these commitments by signaling our Government's intent to further restrict the marketing and advertising of opioids to health care professionals, including asking the pharmaceutical industry to ~~be more open and transparent with~~ immediately cease marketing and advertising of opioids ~~medications~~ to health care professionals, on a voluntary basis.

Today, as we gather here to discuss new areas of collaboration, I am pleased to announce the new progress that has been made on these commitments.

As part of our continued efforts to hold industry to account for their opioid marketing efforts onactivities to -opioid medications to health care professionals, I am pleased to publish the letter I sent tomy voluntary call to action to industry members, as well as -and the 23 responses from industry to date.

Commented [LM4]: HPFB can insert/verify the number at the time of the speech. As of August 7, it's 23 responses.

-The response has been encouraging but we continue to call on industry to do more.

This is part of our ongoing efforts to improve openness and transparency. Moving forward, Health Canada will continue to update Canadians on additional responses received.

The response has been encouraging but we continue to push call on industry to do more.

Making this correspondence available to the public is part of our ongoing commitment to openness and transparency. As part of this commitment, to be open and transparent tSo, this fall, Health Canada will also release a report that will summarizinge what we heard from the Notice of Intent to restrict the marketing and advertising of opioids, which was open for comment from June 19 to July 18.

on this issue and explore regulatory changes to address this issue.

Feedback received during this consultation is helping to We received 40 responses from a wide range of stakeholders, which will inform Health Canada's work to further restrict the marketing and advertising of opioids.

The plenary session tomorrow is another opportunity for you to share your thoughts and help shape how Health Canada moves forward. to help inform this work.

We have also taken an additional step to removing unnecessary regulatory barriers to treatment for substance use disorder.

We heard from our stakeholders, many of whom are in the room today, that nurses are often the main point of contact

for Canadians who need treatment for substance use disorder.

We need to equip them to treat patients where they are.

As of today, nurses can provide opioid medication for treatment purposes outside of a hospital setting.

This will remove a barrier to treatment by allowing patients to stay in their communities instead of travelling to hospitals that can be far away.

We will continue to identify regulatory barriers that can be removed, including the possibility of providing social workers with the same exemptions as nurses.

That is not all.

Our government is also investing in projects and funding focused on prevention and response efforts.

For example, we are allocating $3.4 million for three new innovative opioid-related projects supported through Health Canada's Substance Use and Addiction Program.

Commented [MG5]: Verify number of SUAP projects

These projects will:

- provide peer training on naloxone use
- build capacity for treating opioid addiction
- provide overdose prevention and harm reduction training for paramedics, and
- adapt a tele-health model for opioid replacement therapy delivery in remote areas.

As announced in June, through the Canadian Institutes of Health Research, we will be providing up to $1.5 million to support the work of up to 15 research projects. These will evaluate interventions and practices that have been put in place to address the opioid crisis.

We have also allocated $2.8 million to support innovative opioid research.

Commented [BL6]: CIHR – as per the comments in the other document. I'm confused as to what we're announcing. Could we please make this clear?

While we are focused on reversing the trends of the opioid crisis, we also recognize that the problematic use of substances is a systemic problem in Canadian society.

For example, based on the data tracked by CCSA, we know that the cost of alcohol on our health care and justice system was $14.7 million dollars in 2014.

We have also heard from provinces, such as Manitoba on the rise of incidents involving methamphetamines.

That is why our drugs and substances strategy must focus beyond the opioid crisis, while still addressing the immediate needs.

Today, we are launching a public online consultation to consider next steps and new ideas for the *Canadian Drugs and Substances Strategy*.

Commented [AM7]: Session on this is on Day 2 so this should perhaps read "Tomorrow"

Your feedback will help inform our strategy on drugs and substances, including alcohol. I encourage all Canadians to participate and I look forward to hearing the feedback from the concurrent session tomorrow.

Commented [AM8]: I believe this is a plenary session, not a concurrent session

As we look towards the future, we also need to continue to develop innovative technologies that will help further reduce the harms of substance use.

As part of these efforts, we are also launching the Drug Checking Technology Challenge.

The end goal, which includes a prize of approximately $1 million, is to develop a technology that will allow people such as:

- those who use drugs;
- those who use supervised consumption sites; and
- law enforcement

to determine what is in the drugs so that they have the information available to make the choice that is right for them.

Conclusion

We know that opioid use disorder is complex and destructive, and its impact can be devastating. All too often, we see lives and families shattered by it.

And as I said earlier, no one is immune. This crisis affects people of all ages, backgrounds, professions.

That’s why we need to put our heads together: complex challenges require complex solutions.

This Symposium offers an opportunity to work together on an approach to this public health crisis.

An approach that is comprehensive, collaborative and compassionate.

Commented [AM9]: Same as previous comment – consistency in what pillars are quoted

That means we need to think about, and carefully consider all the resources we have available to us.

We need to work together … all levels of government, stakeholders, people with lived and living experience, and communities.

We need to understand the opioid crisis is a public health crisis. It is not about criminal behavior or moral failing.

People are dying.

If we’re going to help, if we’re going to make a difference … we’re going to have to come together on this.

I know we can do it.

And I am looking forward to listening to you during the next two days, here and online, and continuinge to working with you in the future

Thank you all for your commitment, for joining me here today and a special thank you to all the participants with lived and living experience who are generously shared sharing their personal stories so that we can all learn and benefit from your experience.

DRAFT: December 20 2018

Summary of the Stakeholder Consultation Session at the Opioid Symposium: Further Restricting the Marketing and Advertising of Opioids

Sept 5, 2018

Draft: December 20, 2018

DRAFT

Introduction

Prescriptions written by healthcare practitioners are a common way to access opioids in Canada. Health care practitioners receive information from a variety of sources to inform their prescribing decisions and advice to patients, including from the pharmaceutical industry. Recognizing the urgency of the opioid crisis, and the role that the marketing and advertising of opioids may play in the prescribing of opioids, Health Canada is proposing to further restrict the marketing and advertising of opioids.

Health Canada held a consultation session at the Opioid Symposium on September 5, 2018 to gather feedback from stakeholders on Health Canada's intention to further restrict marketing and advertising of opioids to health care professionals. This session was in addition to the broader consultation Health Canada launched in June 2018 through a Notice of Intent and subsequent publication of a report summarizing the comments Health Canada received.

The following summary of the stakeholder consultation session at the Opioid Symposium in Toronto on September 5, 2018 reflects the views of individuals and groups who attended the Opioid Symposium and chose to participate in the consultation session. As such, this summary cannot be accurately generalized to the wider Canadian population and does not necessarily reflect the Government of Canada's position.

A What We Heard report to summarize the full Opioid Symposium is also available.

Commented [LM1]: Insert web link to broader symposium WWH, when posted

Who provided feedback to Health Canada?

There were close to 200 participants in attendance at the Opioid Symposium, by invitation only. Participants included people with lived and living experience related to opioid use; substance use experts; researchers and academia; law enforcement; first responders; Indigenous groups; drug policy advocates; health care practitioners; health professional organizations; regulatory bodies; health professional education institutions; pain perspective organizations; and representatives of federal, provincial, territorial, and municipal governments.

In addition, approximately 1600 individuals observing the Opioid Symposium via webcast were invited to submit comments to Health Canada via e-mail.

The session on further restricting the marketing and advertising of opioids was a plenary session open to all participants, including those participating virtually.

What were stakeholders asked?

Following a presentation in which Health Canada provided background on opioid marketing and advertising, participants at the Opioid Symposium were asked to work in small groups to answer the question:

What impact could further restrictions to the marketing and advertising of opioids have on: health professionals; patients with acute or chronic pain; people living with substance use disorder; and others, such as individuals or a group/organization you represent?

What did stakeholders say?

With respect to further marketing and advertising restrictions, participants at the Opioid Symposium provided feedback focused on four key themes:

1. **Education for health care professionals and patients** is essential to provide **accurate and up to date information** on opioids.
2. **Access to treatment** should **not be limited** for people who need it
3. **Scope of restrictions** could extend **beyond opioid** medications.
4. **Stigma** could be reduced by **engaging individuals** living with pain and/or opioid use disorder.

1. Education for health care professionals and patients

Participants highlighted that restrictions on marketing and advertising could limit health care professionals' **access to information** about emerging opioid therapies. Participants noted that restrictions could have the consequence of further reducing the information available about opioid use disorder treatments. There was consensus that regardless of the source of the information, there is a strong need for evidence-based information that is up to date on the risks and benefits of opioid products.

Some participants felt that restrictions could increase prescriber awareness of the risks associated with opioids and encourage prescribing decisions based on evidence.

Participants also indicated that restrictions should not limit patients' access to information regarding opioid treatments. It was suggested that public educational information written in plain language could assist patients and prescribers to safely transition to alternative treatments.

2. Access to treatment

Some participants expressed concern that restrictions on opioid advertising could result in prescribers being reluctant to offer opioid treatments, which would **limit patient access** to opioids, including for the treatment of opioid use disorder. They highlighted that without access to opioids, individuals may resort to using illegal drugs, increasing the chance of overdose or death.

On the other hand, restrictions on opioid marketing and advertising could lead to greater awareness of non-pharmaceutical treatment options, including physiotherapy, massage therapy, acupuncture, chiropractic care, and cognitive behavioral therapy. Participants noted these treatments may not be widely accessible since they are not covered by provincial and territorial health care plans. Some participants suggested that non-pharmaceutical treatment programs could be funded by governments or a tax on industry for pharmaceutical patents.

3. Scope of Restrictions

Several participants noted that marketing and advertising of prescription drugs is an issue that extends **beyond opioids**. Others suggested that robust enforcement of existing rules, including the use of criminal sanctions, is necessary.

4. Stigma

Several participants expressed concern that restrictions could **further stigmatize** individuals living with pain or opioid use disorder, by increasing negative perceptions associated with the use of opioid medications. Some participants noted that decriminalizing illegal drugs could help reduce stigma. Participants underscored the importance of considering patients' perspectives when developing policies that aim to reduce problematic opioid use.

Next Steps

We thank all participants who contributed to this consultation session. The feedback received will inform next steps for federal action. We will continue to engage stakeholders and subject matter experts in early 2019.

Visit our Marketing and Advertising of Opioids page for updates on our work and information about other consultation activities.

DRAFT

Résumé de la séance de consultation des intervenants lors du Symposium sur les opioïdes : Restreindre le marketing et la publicité sur les opioïdes

5 septembre 2018

Version provisoire : 20 décembre 2018

Introduction

Les ordonnances écrites par des professionnels de la santé sont un moyen courant d'obtenir des opioïdes au Canada. Les professionnels de la santé reçoivent de l'information de diverses sources, y compris de l'industrie pharmaceutique, dans le but d'orienter leurs décisions en matière de prescriptions ainsi que les conseils qu'ils donnent aux patients. Reconnaissant l'urgence de la crise des opioïdes et le rôle que peuvent jouer le marketing et la publicité en ce qui touche la prescription des opioïdes, Santé Canada propose de restreindre davantage le marketing et la publicité sur les opioïdes.

Le 5 septembre 2018, Santé Canada a tenu une séance de consultation à l'occasion du Symposium sur les opioïdes afin de recueillir les commentaires des intervenants sur l'intention de Santé Canada de restreindre davantage le marketing et la publicité sur les opioïdes auprès des professionnels de la santé. Cette séance s'ajoutait à une consultation plus vaste de Santé Canada lancée en juin 2018 par l'intermédiaire d'un Avis d'intention et la publication subséquente d'un rapport résumant les commentaires reçus par Santé Canada.

Le résumé qui suit de la séance de consultation des intervenants lors du Symposium sur les opioïdes qui a eu lieu à Toronto le 5 septembre 2018 reflète les points de vue des personnes et des groupes qui ont assisté au Symposium et ont choisi de participer à la séance de consultation. À ce titre, le présent résumé ne peut pas être rigoureusement généralisé pour englober la population canadienne dans son ensemble et ne reflète pas nécessairement la position du gouvernement du Canada.

Un rapport « Ce que nous avons entendu » résumant l'ensemble du Symposium sur les opioïdes est également disponible.

Commented [LM1]: Insert web link to broader symposium WWH, when posted

Qui a fourni des commentaires à Santé Canada?

Il y avait près de 200 participants sur invitation seulement au Symposium sur les opioïdes. Les participants comprenaient des personnes ayant vécu ou vivant une expérience liée à la consommation d'opioïdes; des experts dans le domaine; des chercheurs et des universitaires; des membres des forces de l'ordre; des premiers répondants; des groupes autochtones; des défenseurs des politiques sur les drogues; des professionnels de la santé; des organismes de professionnels de la santé; des organismes de réglementation; des établissements d'éducation des professionnels de la santé; des organismes de soutien pour les personnes qui vivent avec une douleur chronique; et des représentants des gouvernements fédéral, provinciaux et territoriaux ainsi que des administrations municipales.

De plus, environ 1 600 personnes assistant au Symposium sur les opioïdes par webdiffusion ont été invitées à faire part de leurs commentaires à Santé Canada par courriel.

La séance sur la restriction du marketing et de la publicité sur les opioïdes était une séance plénière ouverte à tous les participants, y compris les participants en ligne.

Qu'est-ce qui a été demandé aux intervenants?

À la suite d'une présentation contextuelle de Santé Canada sur le marketing et la publicité visant les opioïdes, il a été demandé aux participants du Symposium sur les opioïdes de travailler en petits groupes pour répondre à la question suivante :

Quel serait l'impact de nouvelles restrictions sur le marketing et la publicité sur les opioïdes sur : les professionnels de la santé; les patients souffrant de douleurs chroniques ou aiguës; les personnes atteintes de troubles liés à la consommation d'opioïdes; et les autres, comme les personnes ou un groupe/une organisation que vous représentez?

Qu'ont dit les intervenants?

À l'égard des nouvelles restrictions sur le marketing et la publicité sur les opioïdes, les participants au Symposium sur les opioïdes ont fourni des commentaires axés sur quatre thèmes principaux :

1. **L'éducation des professionnels de la santé et des patients** est essentielle pour fournir **des renseignements exacts et à jour** sur les opioïdes.
2. **L'accès au traitement** ne devrait **pas être limité** pour les personnes qui en ont besoin.
3. **La portée des restrictions** pourrait être élargie **au-delà des opioïdes**.
4. **La stigmatisation** pourrait être réduite en **mobilisant les personnes** qui vivent avec une douleur chronique ou des troubles de consommation d'opioïdes.

1. Éducation des professionnels de la santé et des patients

Les participants ont souligné que les restrictions sur le marketing et la publicité pourraient limiter **l'accès des professionnels de la santé à l'information** sur les nouveaux traitements aux opioïdes. Les participants ont indiqué que les restrictions pourraient réduire encore davantage la quantité de renseignements disponibles au sujet des traitements des troubles liés à la consommation d'opioïdes. Un consensus s'est dégagé sur le besoin absolu de disposer de renseignements à jour fondés sur des données probantes, peu importe leur source, au sujet des risques et des avantages liés aux produits opioïdes.

Certains participants estimaient que les restrictions pourraient accroître la sensibilisation des prescripteurs aux risques liés aux opioïdes et encourager les décisions fondées sur des données probantes en ce qui concerne les prescriptions.

De plus, les participants ont indiqué que les restrictions ne devraient pas limiter l'accès des patients à l'information sur les traitements opioïdes. Il a été suggéré de rédiger en langage clair des messages pour

l'éducation du public en vue spécialement d'aider les patients et les prescripteurs à passer de façon sécuritaire à des traitements alternatifs.

2. Accès aux traitements

Certains participants ont exprimé l'inquiétude que les restrictions sur la publicité relative aux opioïdes pourraient entraîner une réticence de la part des prescripteurs à offrir des traitements aux opioïdes, ce qui pourrait **limiter l'accès des patients** aux opioïdes, y compris pour le traitement des troubles liés à la consommation d'opioïdes. Ils ont souligné que sans l'accès aux opioïdes, certaines personnes pourraient recourir à des drogues illégales, ce qui augmenterait les risques de surdose ou de décès.

D'un autre côté, les restrictions visant le marketing et la publicité sur les opioïdes pourraient permettre de faire connaître davantage d'autres options de traitement non pharmaceutique, comme la physiothérapie, la massothérapie, l'acupuncture, la chiropratique et la thérapie cognitive du comportement. Les participants ont mentionné que ces traitements pourraient ne pas être facilement accessibles puisqu'ils ne sont pas couverts par les régimes de soins de santé provinciaux et territoriaux. Certains participants ont suggéré que les programmes de traitement non pharmaceutiques soient financés par les gouvernements ou par une taxe sur l'industrie pour les brevets de médicaments.

3. Portée des restrictions

Plusieurs participants ont indiqué que le marketing et la publicité sur les médicaments d'ordonnance constituent un problème qui s'étend **au-delà des opioïdes**. D'autres ont souligné la nécessité d'une application stricte des règles existantes, notamment le recours aux sanctions criminelles.

4. Stigmatisation

Plusieurs participants ont exprimé l'inquiétude que les restrictions puissent **accroître la stigmatisation** des personnes qui vivent avec une douleur chronique ou des troubles liés à la consommation d'opioïdes, en augmentant les perceptions négatives relatives à la consommation d'opioïdes. Certains participants ont indiqué que la décriminalisation des drogues illégales pourrait aider à réduire la stigmatisation. Les participants ont souligné l'importance de tenir compte du point de vue des patients dans l'élaboration de politiques visant à réduire la consommation problématique des opioïdes.

Prochaines étapes

Nous remercions tous les participants qui ont contribué à cette séance de consultation. Les commentaires reçus éclaireront les prochaines étapes qu'entreprendra le gouvernement fédéral. Nous poursuivrons nos entretiens avec les intervenants et les experts en matière au début de 2019.

Consultez la page sur le marketing et la publicité visant les opioïdes pour des mises à jour sur nos travaux et des renseignements sur d'autres activités de consultation.

OPIOID SYMPOSIUM
Together, We Can
#StopOverdoses

SYMPOSIUM SUR LES OPIOIDES
Ensemble nous pouvons mettre
#FinAuxSurdoses

You can provide additional comments on **restricting the marketing and advertising of opioids** to:

Vous pouvez soumettre d'autres commentaires sur la possibilité de **restreindre le marketing et la publicité sur les opioïdes** à :

hc.ppiad-dppai.sc@canada.ca

by September 21

d'ici le 21 septembre

OPIOID SYMPOSIUM
Together, We Can
#StopOverdoses

SYMPOSIUM SUR LES OPIOIDES
Ensemble nous pouvons mettre
#FinAuxSurdoses

You can provide additional comments on **restricting the marketing and advertising of opioids** to:

Vous pouvez soumettre d'autres commentaires sur la possibilité de **restreindre le marketing et la publicité sur les opioïdes** à :

hc.ppiad-dppai.sc@canada.ca

by September 21

d'ici le 21 septembre

OPIOID SYMPOSIUM
Together, We Can
#StopOverdoses

SYMPOSIUM SUR LES OPIOIDES
Ensemble nous pouvons mettre
#FinAuxSurdoses

You can provide additional comments on **restricting the marketing and advertising of opioids** to:

Vous pouvez soumettre d'autres commentaires sur la possibilité de **restreindre le marketing et la publicité sur les opioïdes** à :

hc.ppiad-dppai.sc@canada.ca

by September 21

d'ici le 21 septembre

OPIOID SYMPOSIUM
Together, We Can
#StopOverdoses

SYMPOSIUM SUR LES OPIOIDES
Ensemble nous pouvons mettre
#FinAuxSurdoses

You can provide additional comments on **restricting the marketing and advertising of opioids** to:

Vous pouvez soumettre d'autres commentaires sur la possibilité de **restreindre le marketing et la publicité sur les opioïdes** à :

hc.ppiad-dppai.sc@canada.ca

by September 21

d'ici le 21 septembre

Communication Services Request (Part I)

Web Publishing - Graphic Design - Print - Video - Social Media

Complete all applicable sections in Part I. Part II will be completed in consultation with the Communications and Public Affairs Branch.

Number

A GENERAL INFORMATION

Contact name: J. Mark Smith
Catherine Kay

Branch/Region: HPFB

Directorate: MHPD

Target release date: 03/05/2019

Strategic Communications Advisor: David S Martin

Project title: **Notice to Stakeholders: Further Restrictions to the Marketing and Advertising of Opioids**

Request to:
- [x] Create new content or product
- [ ] Update/reprint existing content or product (note: web content changes under 3 paragraphs do not require a form, contact your Digital Communications Advisor)

Service required: (check all that apply)

- [x] Web Publishing - Internet (complete **Section D**)
- [ ] Print (complete **Section F**)
- [x] Social Media (complete **Section H**)
- [ ] Graphic Design (complete **Section E**)
- [ ] Video (complete **Section G**)

Intranet Publishing refer to *my*Source

B PROJECT DETAILS

What is the objective of the target groups?

Health Canada is informing Market Authorization Holders (MAHs) of proposed enhanced Terms and Conditions (T&Cs) on Class B opioid products. This would serve to further restrict the marketing and advertising of opioids under the authority of section C.01.014.21 of the Food and Drug Regulations

Identify primary and secondary audiences?

Industry

Is there a communications plan available? [x] No [ ] Yes, supply a copy.

Are there any partners associated with this project? [x] No [ ] Yes, specify:

Strategic priority (choose one)

- [ ] Ministerial or CPHO announcement
- [ ] TBS requirement
- [ ] New requirements for service delivery
- [ ] Ministerial or CPHO priority
- [ ] Legislation or guidelines
- [ ] Consultation
- [ ] Departmental/Agency
- [ ] Marketing campaign/public outreach
- [x] Other, specify:

Opioids crisis

Indicate date and time, if applicable:

C DESCRIPTION

Type of product - check all that apply

- [ ] Brochure/Pamphlet
- [ ] Flyer
- [ ] Poster
- [ ] Video/animation
- [ ] Web form, accessible fillable
- [ ] Consultation document
- [ ] Guidance document
- [ ] Promotional items
- [ ] Web banner
- [ ] Display/pull-up banner
- [ ] Infobites
- [ ] Report
- [ ] Web button
- [ ] E-Bulletin
- [ ] Infographic
- [ ] Social media post
- [x] Web content (HTML)
- [ ] Fact sheet
- [ ] Postcard
- [ ] Summary document
- [ ] Web thumbnail
- [ ] Other, specify:

Health Canada and the Public Health Agency of Canada
Santé Canada et l'Agence de la santé publique du Canada
Canada

Communication Services Request (Part I)

Number

Web Publishing - Graphic Design - Print - Video - Social Media

Complete all applicable sections in Part I. Part II will be completed in consultation with the Communications and Public Affairs Branch.

How often will the content or product be updated (e.g., updated annually, data released quarterly, etc.)?

N/A

Note: Client are responsible for retaining original submitted content.

D WEB PUBLISHING

Format

- [x] HTML
- [ ] HTML and downloadable format → [ ] PDF [ ] txt [ ] xls [ ] Other, specify:
- [ ] Video

What is the approximate length of the source document(s) (e.g., total page count in Microsoft Word or Excel)?

Product Name	English	French	Other	
Notice to Stakeholders: Further Restrictions to the Marketing and Advertising of Opioids	2	2		+

Does similar content or a previous version of this content already exist online? [x] No [ ] Yes

If yes, can older information be removed? [ ] No [ ] Yes

When will you update and/or remove this content?

Update: Removal:

H SOCIAL MEDIA

What channels would you like to publish to:

	Health theme	Ministerial	CPHO
Twitter	[x]	[x]	[ ]
Facebook	[ ]		
YouTube	[ ]		

	Health Canada	Public Health Agency of Canada
LinkedIn	[x]	[ ]

What is the proposed frequency of the posts?

▶ To be completed by Strategic Communications

Identify the risk(s) of not providing the product(s) or service(s) requested?

What other opportunities are there to enhance the reach of the product(s) or service(s) to target group?

- [x] This request is consistent with the current communications approach for this project.
- [ ] The content will impact the Department or Agency's relationship with other government departments, stakeholders, or other levels of government.
- [ ] The topic received media coverage in the last 12 months.

Comments

Post, as requested.

Communication Services Request (Part I)

Web Publishing - Graphic Design - Print - Video - Social Media

Complete all applicable sections in Part I. Part II will be completed in consultation with the Communications and Public Affairs Branch.

Number

PROJECT SUPPORT

Name: David Martin | Digital signature: [redacted] | Date: 02/19/2019

▶ Program Director

Name: | Digital signature: | Date:

Communication Services Request (Part I)

Number

Web Publishing - Graphic Design - Print - Video - Social Media

Complete all applicable sections in Part I. Part II will be completed in consultation with the Communications and Public Affairs Branch.

A GENERAL INFORMATION

Contact name: J. Mark Smith
Catherine Kay

Branch/Region: HPFB

Directorate: MHPD

Target release date: 02/28/2019

Strategic Communications Advisor: David S Martin, Elizabeth Keeping

Project title: **Opioids Transparency**

Request to:
- [x] Create new content or product
- [ ] Update/reprint existing content or product (note: web content changes under 3 paragraphs do not require a form, contact your Digital Communications Advisor)

Service required: (check all that apply)

- [x] Web Publishing - Internet (complete **Section D**)
- [ ] Graphic Design (complete **Section E**)
- [ ] Print (complete **Section F**)
- [ ] Video (complete **Section G**)
- [x] Social Media (complete **Section H**)

Intranet Publishing refer to *my*Source

B PROJECT DETAILS

What is the objective of the target groups?

To encourage the compliance of companies who are required to submit risk management plans (RMPs) (requests made, receipt of RMP, status of review, etc.)

To meet the information needs of the target groups by practicing transparency on this high-profile public health file.

Identify primary and secondary audiences?

Industry, Public

Is there a communications plan available? [x] No [ ] Yes, supply a copy.

Are there any partners associated with this project? [ ] No [x] Yes, specify: ROEB. TPD. PPIAD

Strategic priority (choose one)

- [ ] Ministerial or CPHO announcement
- [ ] Ministerial or CPHO priority
- [ ] Departmental/Agency
- [ ] TBS requirement
- [ ] Legislation or guidelines
- [ ] Marketing campaign/public outreach
- [ ] New requirements for service delivery
- [ ] Consultation
- [x] Other, specify:
  Opioids Crisis

Indicate date and time, if applicable:

C DESCRIPTION

Type of product - check all that apply

- [ ] Brochure/Pamphlet
- [ ] Consultation document
- [ ] Display/pull-up banner
- [ ] E-Bulletin
- [ ] Fact sheet
- [ ] Flyer
- [ ] Guidance document
- [ ] Infobites
- [ ] Infographic
- [ ] Postcard
- [ ] Poster
- [ ] Promotional items
- [ ] Report
- [x] Social media post
- [ ] Summary document
- [ ] Video/animation
- [ ] Web banner
- [ ] Web button
- [x] Web content (HTML)
- [ ] Web thumbnail
- [ ] Web form, accessible fillable
- [ ] Other, specify:

Health Canada and the Public Health Agency of Canada | Santé Canada et l'Agence de la santé publique du Canada

Canada

Communication Services Request (Part I)

Web Publishing - Graphic Design - Print - Video - Social Media

Complete all applicable sections in Part I. Part II will be completed in consultation with the Communications and Public Affairs Branch.

Number

How often will the content or product be updated (e.g., updated annually, data released quarterly, etc.)?

Quarterly

Note: Client are responsible for retaining original submitted content.

D WEB PUBLISHING

Format

- [x] HTML
- [ ] HTML and downloadable format → [ ] PDF [ ] txt [ ] xls [ ] Other, specify:
- [ ] Video

What is the approximate length of the source document(s) (e.g., total page count in Microsoft Word or Excel)?

Product Name	English	French	Other	
Compliance Table	17			+
Possible cross-links to the DHPR				+
Landing page TBC	2			+
				+

Does similar content or a previous version of this content already exist online? [x] No [ ] Yes

If yes, can older information be removed? [x] No [ ] Yes

When will you update and/or remove this content?

Update: Removal:

H SOCIAL MEDIA

What channels would you like to publish to:

	Health theme	Ministerial	CPHO
Twitter	[x]	[x]	[ ]
Facebook	[ ]		
YouTube	[ ]		

	Health Canada	Public Health Agency of Canada
LinkedIn	[x]	[ ]

What is the proposed frequency of the posts?

▶ To be completed by Strategic Communications

Identify the risk(s) of not providing the product(s) or service(s) requested?

What other opportunities are there to enhance the reach of the product(s) or service(s) to target group?

- [ ] This request is consistent with the current communications approach for this project.
- [x] The content will impact the Department or Agency's relationship with other government departments, stakeholders, or other levels of government.

Communication Services Request (Part I)

Web Publishing - Graphic Design - Print - Video - Social Media

Complete all applicable sections in Part I. Part II will be completed in consultation with the Communications and Public Affairs Branch.

Number

PROJECT SUPPORT

☐ The topic received media coverage in the last 12 months.

Comments

Name: Aldege Bellefeuille | Digital signature: [redacted] | Date: 02/19/2019

▶ Program Director

Name: Katherine Soltys | Digital signature: [redacted] | Date: 02/19/2019
